# Supplementary material for: Atomically precise ultrasmall copper cluster for room-temperature highly regioselective dehydrogenative coupling
Source: Nat Commun. 2023 Oct 28;14:6877. doi: 10.1038/s41467-023-42688-3 (PMC10613312; doi:10.1038/s41467-023-42688-3)
Supplement: Supplementary file 1 — Supplementary information [file 41467_2023_42688_MOESM1_ESM.pdf]

Supplementary information

Atomically Precise Ultrasmall Copper Cluster for Room-Temperature Highly Regioselective Dehydrogenative Coupling

---

In the format provided by the authors and unedited

Supplementary Information (SI) for

Atomically Precise Ultrasmall Copper Cluster for Room-Temperature Highly Regioselective Dehydrogenative Coupling

Teng Jia[1], Yi-Xin Li[1], Xiao-Hong Ma[1], Miao-Miao Zhang[1], Xi-Yan Dong[1,2], Jie Ai[1] & Shuang-Quan Zang[1]*

[1]Henan Key Laboratory of Crystalline Molecular Functional Materials, Henan International Joint Laboratory of Tumor Theranostcal Cluster Materials, Green Catalysis Center, and College of Chemistry, Zhengzhou University, Zhengzhou, P. R. China.

[2]College of Chemistry and Chemical Engineering, Henan Polytechnic University, Jiaozuo, P. R. China.

Correspondence to: zangsqzg@zzu.edu.cn.

Contents

General Methods and Materials 3
Synthetic Procedures 4
Characterization of $Cu_3NC^{(NHC)}$, $Cu_3NC^{(BINAP)}$ and $Cu_3NC^{(Pz)}$ 6
General Procedure for $Cu_3NC^{(NHC)}$-Catalyzed the A^3 Coupling Reaction and the Redox-A^3 Coupling Reaction 19
Investigation on Lowest Catalyst Loading of the $Cu_3NC^{(NHC)}$ for both Organic Transformations 24
Catalytic Mechanism Studies by a series of Control Experiments 25
Catalytic Mechanism Studies by Density Functional Theory (DFT) Calculations 27
The Preliminary Kinetic Studies of the A^3 Coupling Reaction and Redox-A^3 Coupling Reaction 29
Recyclability of the $Cu_3NC^{(NHC)}$ Catalyzed A^3 Coupling Reaction and Redox-A^3 Coupling Reaction 31
Tables of Crystal Data and Structure Refinements 32
Compound Characterization of A^3 Coupling Reaction 33
Compound Characterization of Redox-A^3 Coupling Reaction 59
^{1}H, ^{13}C and ^{19}F NMR Spectra of New Compounds 66
References. 157

General Methods and Materials

All the reactions were carried out under ambient atmosphere (air) conditions unless otherwise noted. All commercial reagents and solvents were obtained from the commercial provider and used without further purification. ^{1}H NMR and ^{13}C NMR spectra were recorded on Bruker 600 MHz spectrometers. Chemical shifts were reported relative to internal tetramethylsilane (δ 0.00 ppm), CD_3CN (δ 1.94 ppm) or $CDCl_3$ (δ 7.26 ppm) for ^{1}H NMR and $CDCl_3$ (δ 77.0 ppm), CD_3CN (δ 118.3 ppm) for ^{13}C NMR. Flash column chromatography was performed on 300-400 mesh silica gel.

Powder X-ray diffraction (PXRD) patterns of $Cu_3NC^{(NHC)}$, $Cu_3NC^{(BINAP)}$ and $Cu_3NC^{(Pz)}$ were recorded on a Rigaku B/Max-RB X-ray diffractometer with Cu-Kα radiation (λ = 1.5418 Å) in air at room temperature. Electrospray ionization mass spectrometry (ESI–MS) of the clusters were recorded on an AB Sciex X500R Q-TOF spectrometer. UV–vis absorption spectra were obtained by means of a Hitachi UH4150 UV–visible spectrophotometer.

Single crystal analysis. Single-crystal X-ray diffraction measurements of $Cu_3NC^{(NHC)}$ and $Cu_3NC^{(Pz)}$ were performed on a Rigaku XtaLAB Pro diffractometer with Cu-Kα radiation (λ = 1.5418 Å) at 200 K. Data collection and reduction were performed by the program CrysAlisPro[(1)]. The crystal structures were solved with direct methods (*SHELXS*)[(2)] and refined by full-matrix least squares on F^2 using *OLEX2*[(3)], which utilizes the *SHELXL*-2015 module[(4)]. All non-hydrogen atoms were refined anisotropically. Hydrogen atoms were placed in calculated positions refined using idealized geometries and assigned fixed isotropic displacement parameters. Structure refinement was handled with different strategies according to the electron density distribution. The imposed restraints in least-squares refinement of each structure were noted in the corresponding CIF files. Detailed information about the X-ray crystal data, intensity collection procedure and refinement results for all cluster compounds is summarized in Supplementary Table 3 and Supplementary Table 4.

Materials

Three-component coupling of aldehydes, alkynes, and amines (including the A^3 coupling reaction and the redox-A^3 reaction) via C–H activation was carried out under the atmosphere of dried and purified N_2 using standard Schlenk. Toluene and THF were dried over sodium/benzophenone and distilled under nitrogen prior to use. DCM was dried over calcium hydride and distilled under nitrogen prior to use. Ethanol and methanol was dried over Iodine/magnesium strips and distilled under nitrogen prior to use. The other dry solvent including 1,4-dioxane, acetonitrile and DCE were directly purchased from Energy Chemical. BINAP, 3,5-diphenyl-pyrazole, aldehydes, alkynes and amines were purchased from Bidepharm, Energy Chemical and Heowns without further purification. The Vulcan XC-72 carbon black was purchased from Macklin.

The ^{t}BuSCu was prepared according to the reported literatures[(5)].

Synthetic Procedures

General Procedure for Synthetic Carbene Ligand of $Cu_3NC^{(NHC)}$

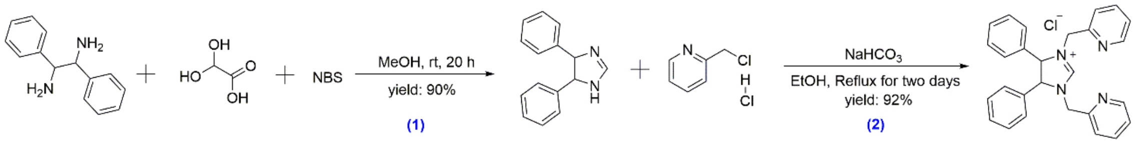

Supplementary Figure 1. The route of synthesizing NHC ligand.

Synthesis of 4,5-diphenyl-4,5-dihydro-1H-imidazole (1)[(6)]

Under N_2 atmosphere, 1,2-diphenylethylenediamine (3.0 g, 14.1 mmol), dry MeOH (100.0 mL) and formylformic acid (1.5 g, 16.3 mmol) were added into the dry round-bottom flask in turn. The reaction mixture was stirred at room temperature for 4 h. Then NBS (3.0 g, 18.54 mmol) was added into the stirred mixture of the round-bottom flask and the reaction mixture was stirred at room temperature for 20 h. The reaction was monitored by TLC. When 1,2-diphenylethylenediamine was consumed, the reaction was quenched by adding sat. $Na_2S_2O_5$ (aq.), then concentrated. The residue was added to 5.0% NaOH (aq.) and was extracted by EA. The organic phase was dried by Na_2SO_4, then concentrated. The crude product was then purified by column chromatography to give the 4,5-diphenyl-4,5-dihydro-1H-imidazole as white solid with overall isolated yield 90%. ^{1}H NMR (600 MHz, $CDCl_3$) δ 7.36 – 7.32 (m, 4H), 7.29 (t, J = 7.3 Hz, 2H), 7.26 – 7.23 (m, 4H), 4.70 (s, 2H).

Synthesis of 4,5-diphenyl-1,3-bis(pyridin-2-ylmethyl)-4,5-dihydro-1H-imidazol-3-ium chloride (2)[(7)]

Under N_2 atmosphere, 4,5-diphenyl-4,5-dihydro-1H-imidazole (1.1 g, 5.0 mmol), $NaHCO_3$ (1.3 g, 15.0 mmol), dry EtOH (50.0 mL) and 2-(Chloromethyl)pyridine hydrochloride (1.7 g, 10.3 mmol) were added into the dry round-bottom flask in turn. The reaction mixture was stirred and refluxed at 80 ^oC by oil bath for 2 days. The reaction was monitored by TLC. When 4,5-diphenyl-4,5-dihydro-1H-imidazole was consumed, the reaction was shut down to cool to ambient temperature, then concentrated. The crude product was then purified by column chromatography to give the 4,5-diphenyl-1,3-bis(pyridin-2-ylmethyl)-4,5-dihydro-1H-imidazol-3-ium chloride as white solid with overall isolated yield 92%. ^{1}H NMR (600 MHz, CD_3CN) δ 9.44 (s, 1H), 8.69 (d, J = 4.3 Hz, 2H), 7.75 (td, J = 7.7, 1.7 Hz, 2H), 7.45 – 7.39 (m, 6H), 7.36 (dd, J = 7.1, 5.1 Hz, 2H), 7.29 (dd, J = 7.7, 1.6 Hz, 4H), 7.23 (d, J = 7.7 Hz, 2H), 5.09 (d, J = 16.0 Hz, 2H), 5.02 (s, 2H), 4.39 (d, J = 16.0 Hz, 2H). ^{13}C NMR (151 MHz, CD_3CN) δ 161.5, 153.9, 150.7, 138.4, 136.4, 130.8, 130.4, 129.0, 124.6, 124.0, 74.0, 51.5.

General Procedure for Synthesis of $Cu_3NC^{(NHC)}$, $Cu_3NC^{(BINAP)}$ and $Cu_3NC^{(Pz)}$

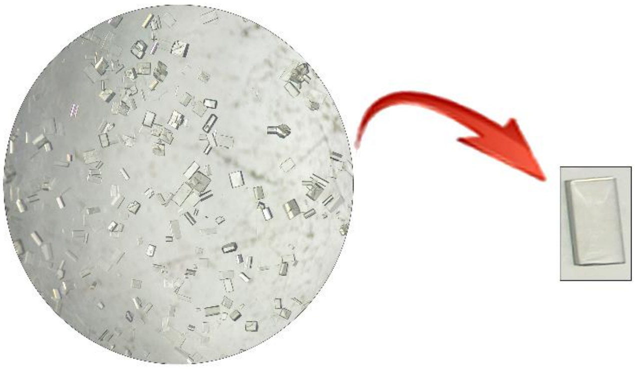

Supplementary Figure 2. Photographs of the $Cu_3NC^{(NHC)}$ single crystals.

Synthesis of $Cu_3NC^{(NHC)}$: Under N_2 atmosphere, 4,5-diphenyl-1,3-bis(pyridin-2-ylmethyl)-4,5-dihydro-1H-imidazol-3-ium chloride (440.2 mg, 1.0 mmol) was dissolved in 20 mL of H_2O, to which 20 mL of H_2O solution containing KPF_6 (404.8 mg, 2.2 mmol) was added under vigorous stirring at room temperature. The suspension was centrifuged and the white solid was collected. The white solid was dissolved in 30.0 mL of MeCN. Overabundant Cu power (320 mg, 5.0 mmol) was then added to the stirred mixture. The reaction mixture was stirred at room temperature for 24 h. After filtration and concentration, diethyl ether was added to obtain the crude product. The crude product was dissolved in acetonitrile and the resulting solution was diffused with diethyl ether in vapor phase to obtain colorless crystals of **$Cu_3NC^{(NHC)}$** (Yield: 67.8%, calculated based on NHC ligand) for 5 days at room temperature. ^1H NMR (600 MHz, CD_3CN) δ 8.48 (d, J = 4.3 Hz, 6H), 7.99 (t, J = 7.4 Hz, 6H), 7.63 – 7.56 (m, 6H), 7.51 – 7.28 (m, 30H), 7.23 (d, J = 7.5 Hz, 6H), 4.93 (s, 6H), 4.17 (d, J = 15.6 Hz, 6H), 3.50 (d, J = 15.7 Hz, 6H). ^{13}C NMR (151 MHz, CD_3CN) δ 154.2, 152.3, 141.2, 132.2, 131.1, 130.4, 126.4, 126.1, 72.0, 53.0.

Synthesis of $Cu_3NC^{(BINAP)}$(8): BINAP (0.045 g, 0.07 mmol) and ^{t}BuSCu (0.025 g, 0.16 mmol) were well mixed in 15 mL acetonitrile/toluene (volume ratio 2:1) at room temperature. The mixture was treated under ultrasonic conditions until a clear solution was obtained. Then 250 μLCS_2 was added. The solution quickly turned to tawny when exposed to air. The tawny solution was allowed to evaporate slowly in darkness at room temperature. After approximately 5 days, yellow block crystals of **$Cu_3NC^{(BINAP)}$** were obtained in a yield of 49.0% (calculated based on BINAP). ^1H NMR (400 MHz, DMSO) δ 7.98 (s, 12H), 7.59 (d, J = 8.1 Hz, 12H), 7.44 – 7.29 (m, 20H), 7.25 (t, J = 7.5 Hz, 5H), 7.15 (dd, J = 15.0, 7.1 Hz, 12H), 6.73 (dd, J = 34.8, 27.5 Hz, 23H), 6.45 (s, 12H), 1.10 (s, 9H).

Synthesis of $Cu_3NC^{(Pz)}$(9): $Cu(CH_3CN)_4PF_6$ (358 mg, 0.96 mmol) and 3,5-diphenyl-pyrazole (212 mg, 0.96 mmol) were dissolved in 5 mL acetone. The mixture was stirred to provide a clear, slightly green solution. The NEt_3 (0.16 mL, 116 mg, 1.15 mmol) was added to stirred solution by dropwise and formed a white precipitate, which was stirred for 30 min, then filtered, washed with acetone and vacuum-dried. Finally, the product

was dissolved in DCM-Et_2O solution and colorless crystals of **$Cu_3NC^{(Pz)}$** were obtained in a yield of 80 % (218 mg). ^{1}H NMR (600 MHz, $CDCl_3$) δ 7.69 (d, *J* = 7.5 Hz, 12H), 7.20 (t, *J* = 7.3 Hz, 8H), 7.06 (t, *J* = 7.5 Hz, 12H), 6.76 (s, 4H). ^{13}C NMR (151 MHz, $CDCl_3$) δ 155.1, 132.6, 128.6, 128.0, 126.6, 102.2.

General Procedure for Synthesis of Deuterated Phenylacetylene

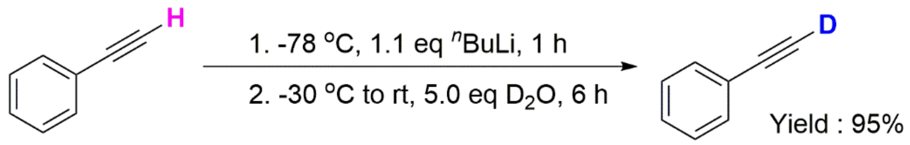

Supplementary Figure 3. The route of synthesizing deuterated phenylacetylene

Under nitrogen atmosphere, ^{n}BuLi (1.60 M in hexane, 0.65 mL, 1.1 mmol) was added dropwise to a solution of phenylacetylene (102.1 mg, 1.00 mmol) in 25 mL dry THF at –78 °C. The mixture was stirred for 1 hour then allowed to warm to –30 °C and stirred for another 2 h before the addition of D_2O (90.0 mg, 5.0 mmol) in one portion. The resulting mixture was allowed to warm to room temperature gradually and stirred for 6 h. Removal of the volatiles under reduced pressure gave pale-yellow residue that was then purified by column chromatography to give pale-yellow oil with overall isolated yield: 95%.

Characterization of $Cu_3NC^{(NHC)}$, $Cu_3NC^{(BINAP)}$ and $Cu_3NC^{(Pz)}$

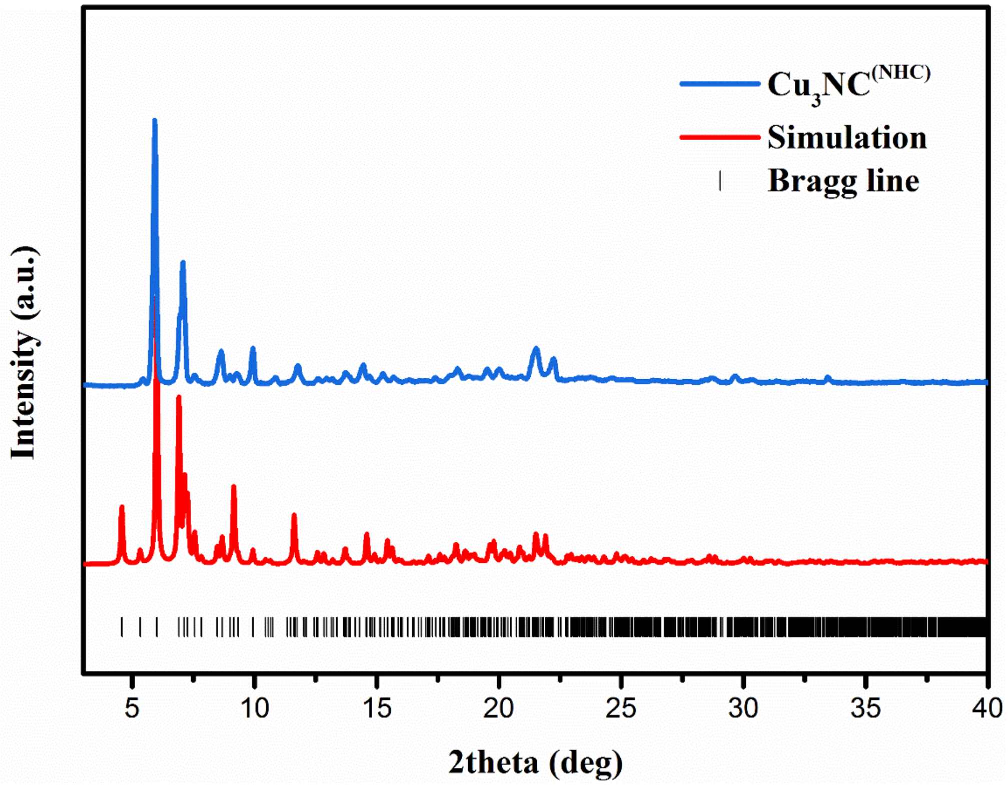

Supplementary Figure 4. PXRD patterns of $Cu_3NC^{(NHC)}$.

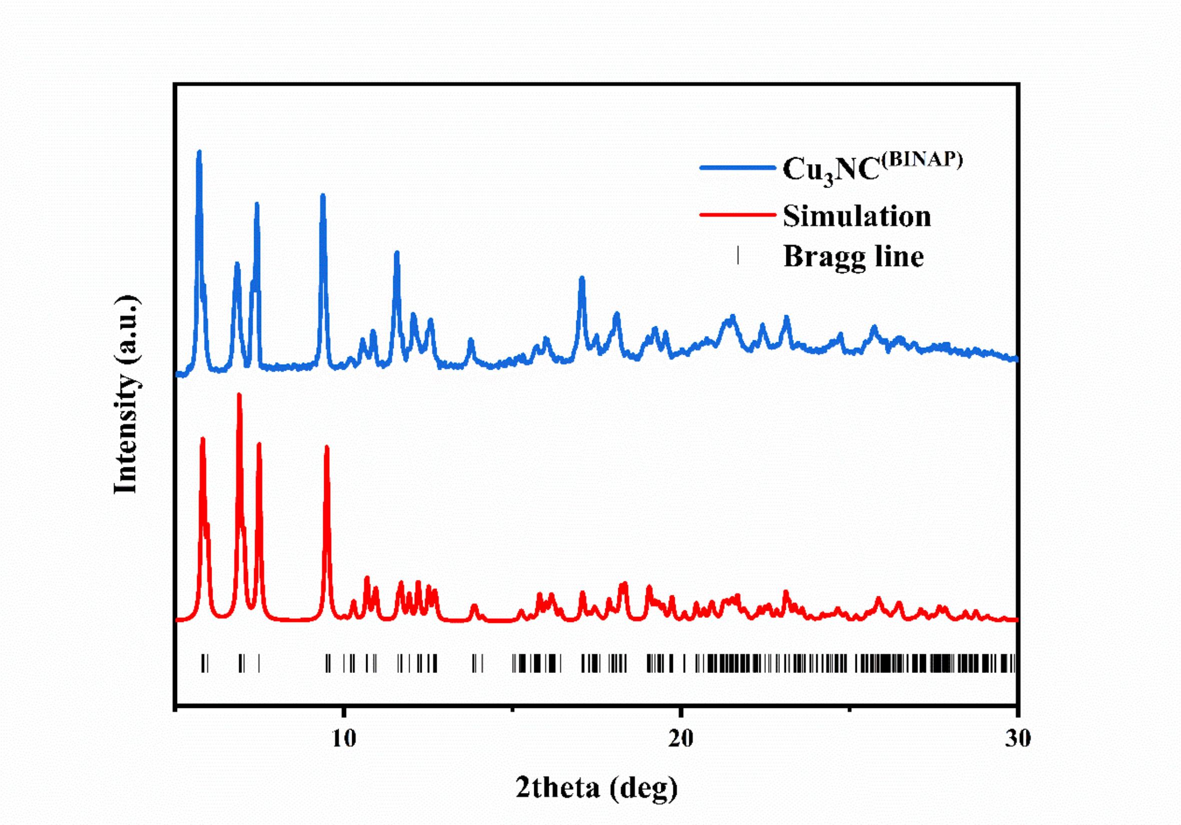

Supplementary Figure 5. PXRD patterns of $Cu_3NC^{(BINAP)}$.

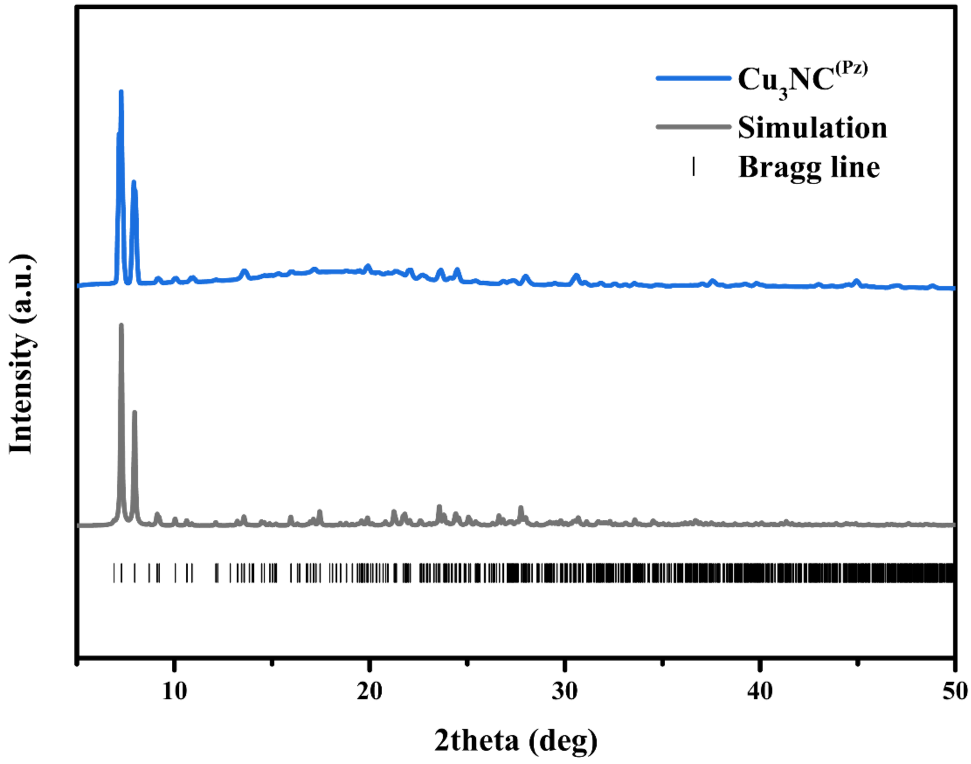

Supplementary Figure 6. PXRD patterns of $Cu_3NC^{(Pz)}$.

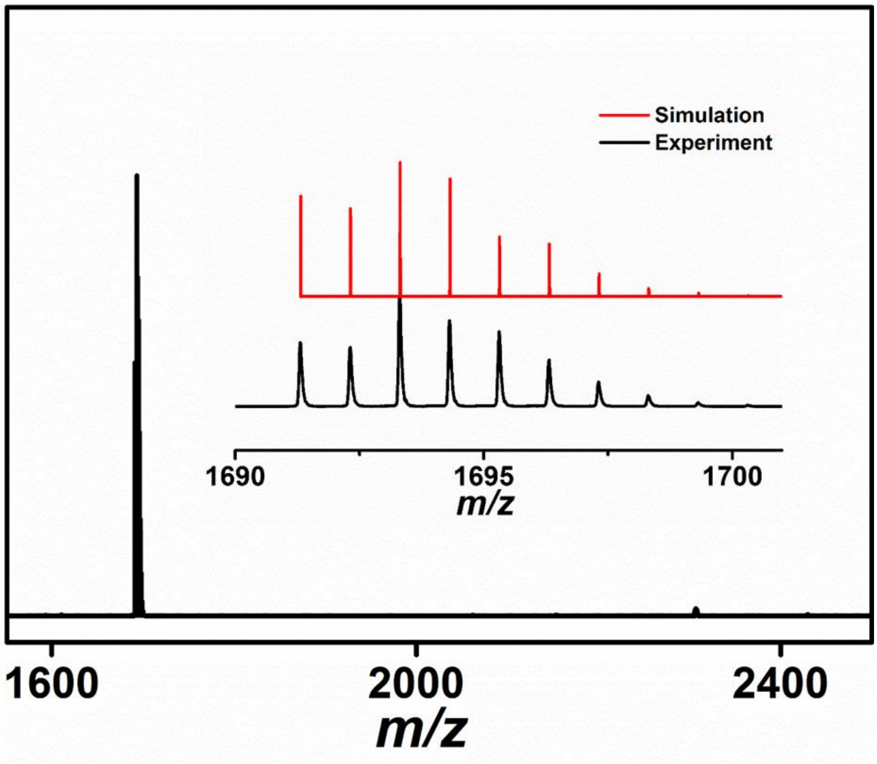

Supplementary Figure 7. ESI–MS spectrum of the $Cu_3NC^{(NHC)}$.

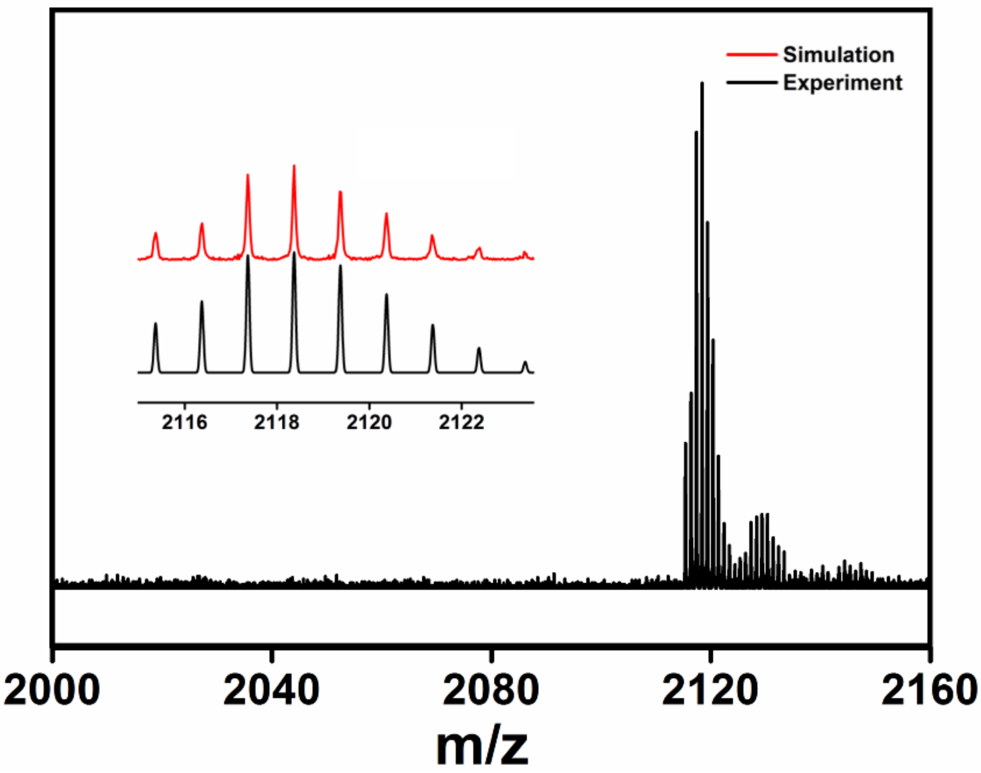

Supplementary Figure 8. ESI–MS spectrum of the $Cu_3NC^{(BINAP)}$.

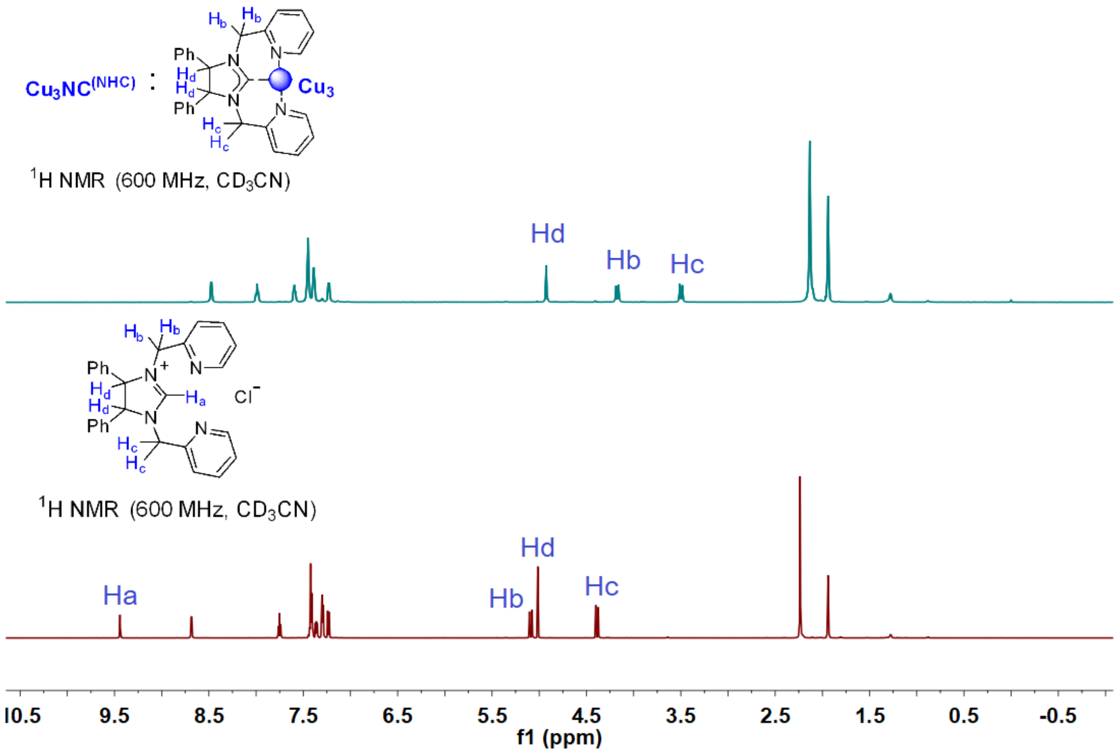

Supplementary Figure 9. ^{1}H NMR spectra of $Cu_3NC^{(NHC)}$ and NHC ligand in MeCN-d_3.

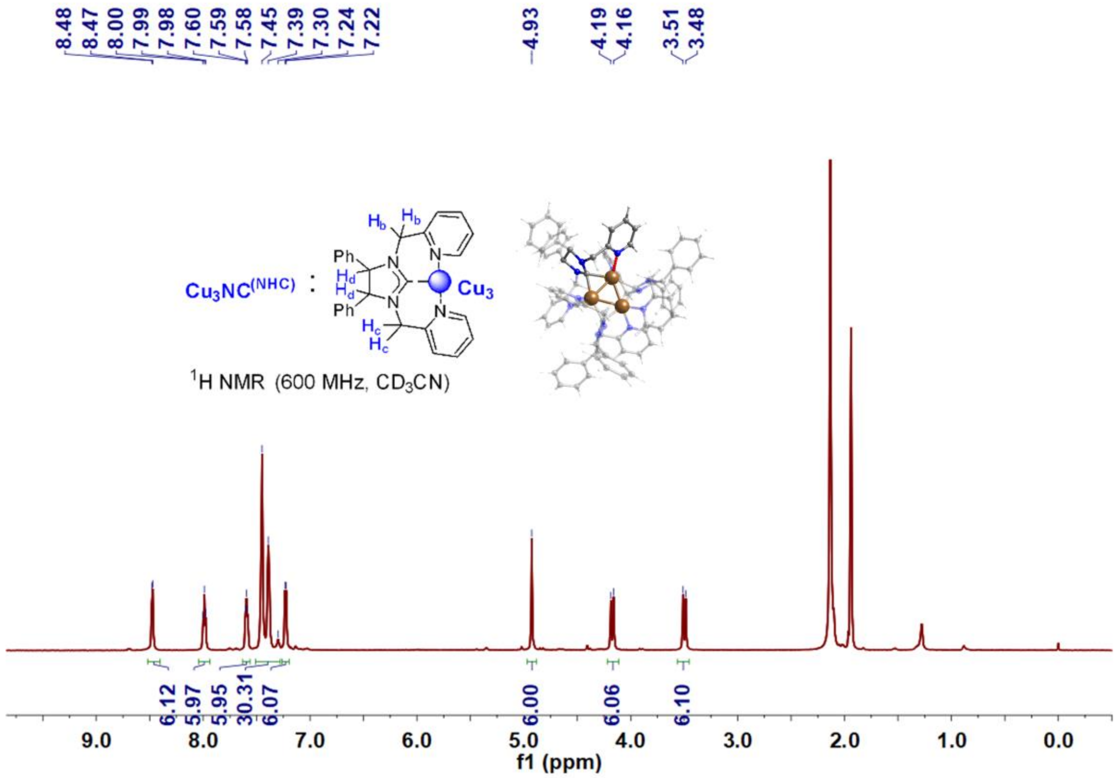

Supplementary Figure 10. ^{1}H NMR spectrum of $Cu_3NC^{(NHC)}$ in MeCN-d_3, Inset: the structures of $Cu_3NC^{(NHC)}$ (CCDC: 2268919).

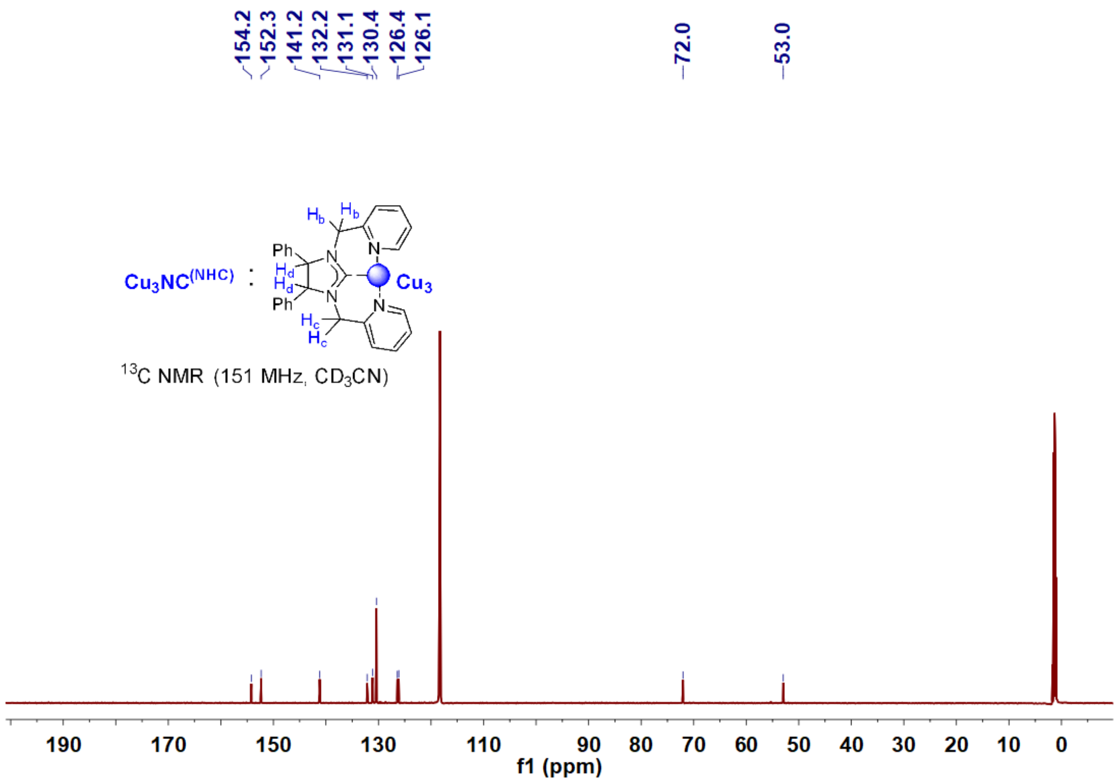

Supplementary Figure 11. ^{13}C NMR spectrum of $Cu_3NC^{(NHC)}$ in MeCN-d_3.

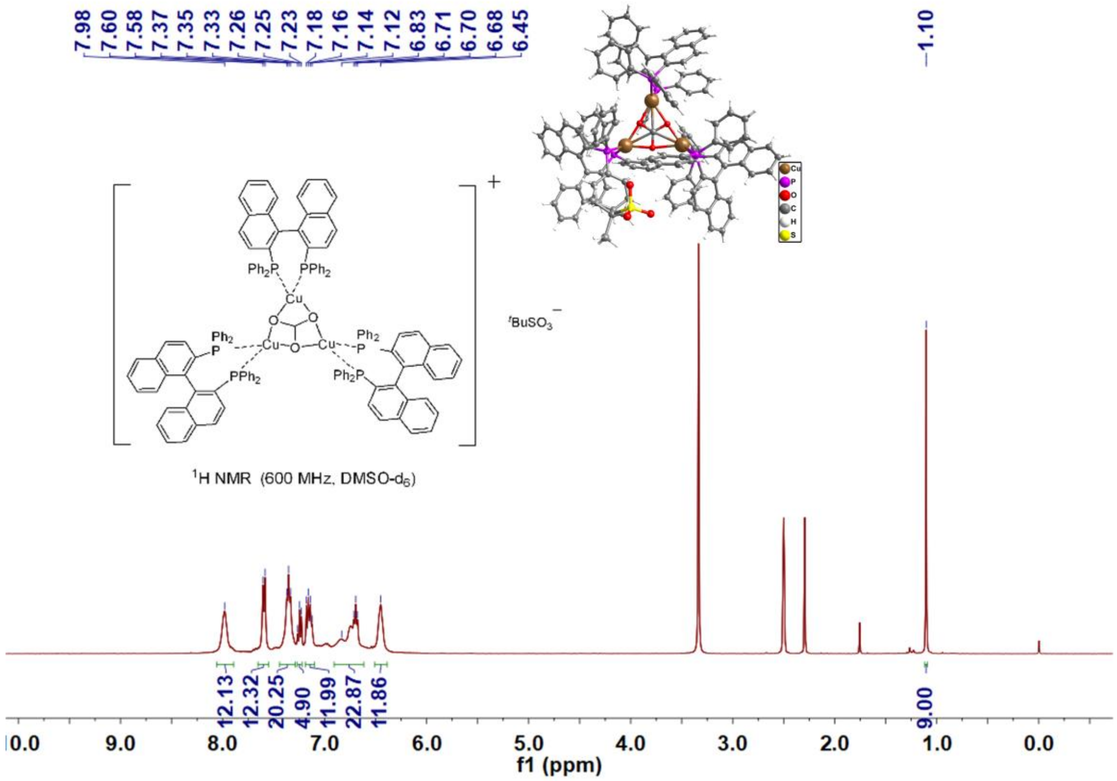

Supplementary Figure 12. ^{1}H NMR spectrum of $Cu_3NC^{(BINAP)}$ in DMSO-d_6, Inset: the structures of $Cu_3NC^{(BINAP)}$.

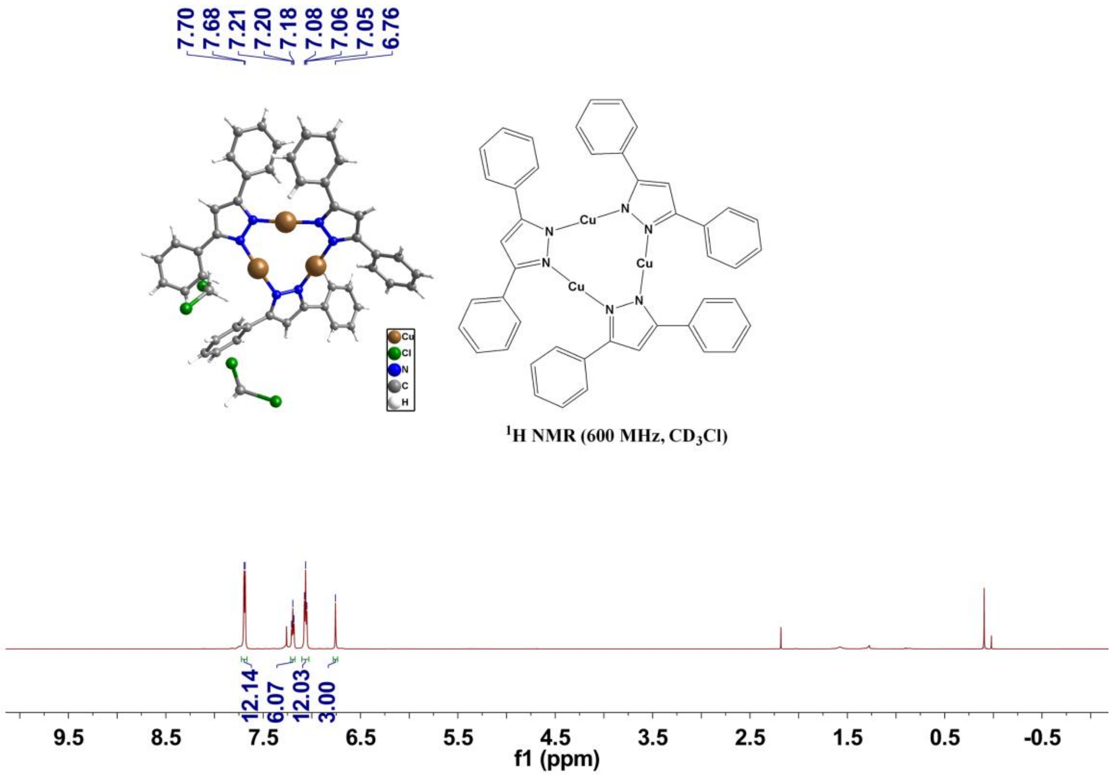

Supplementary Figure 13. ^{1}H NMR spectrum of $Cu_3NC^{(Pz)}$ in CD_3Cl, Inset: the structures of $Cu_3NC^{(Pz)}$ (CCDC: 2268920).

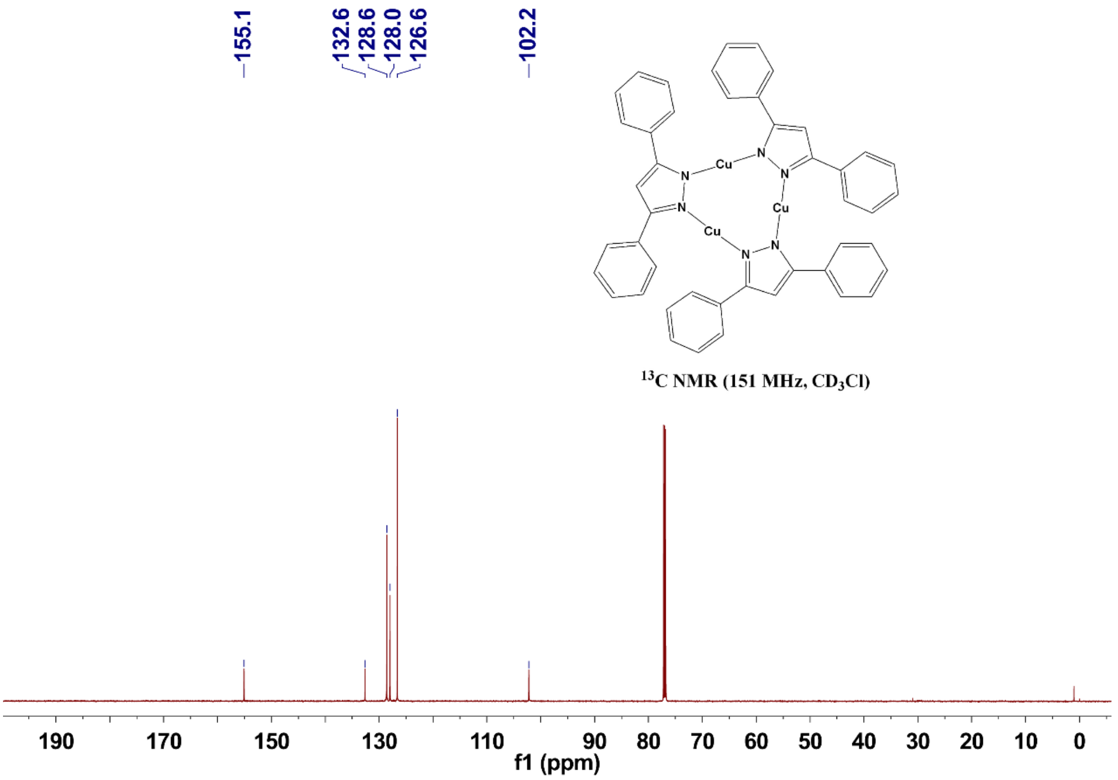

Supplementary Figure 14. ^{13}C NMR spectrum of $Cu_3NC^{(Pz)}$ in CD_3Cl.

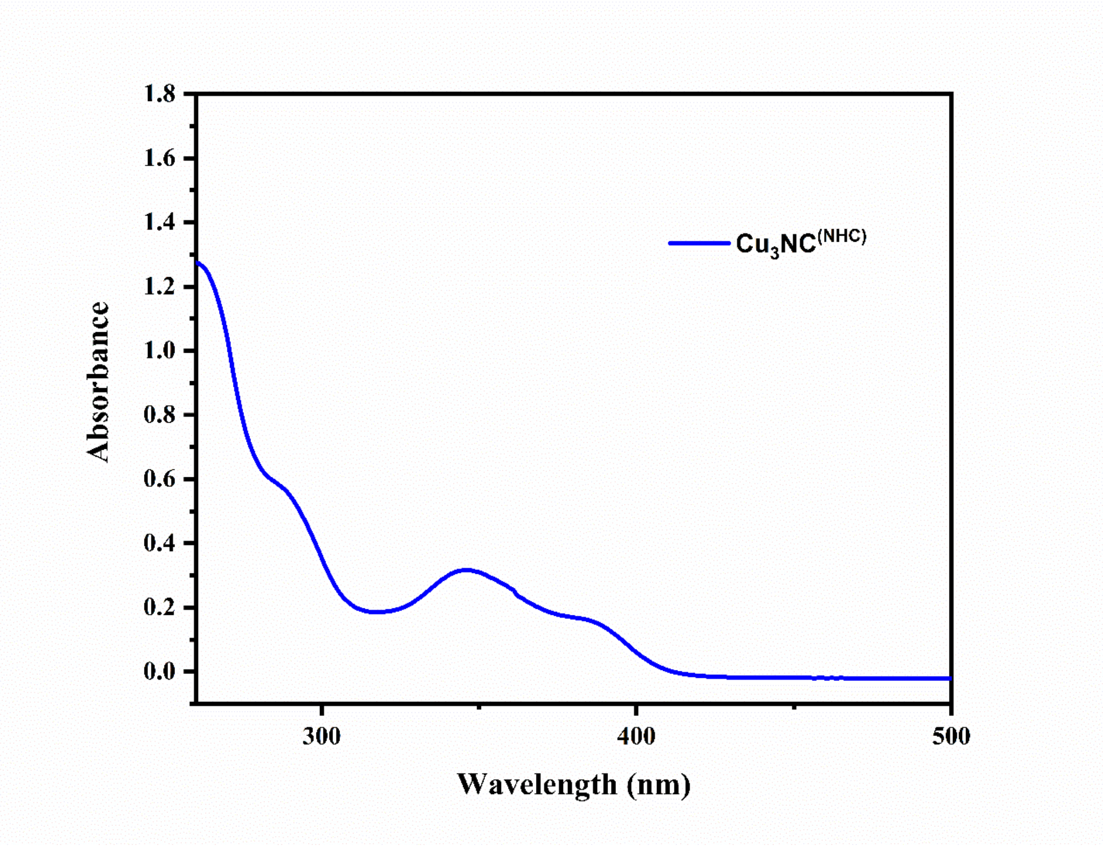

Supplementary Figure 15. UV–vis spectrum of $Cu_3NC^{(NHC)}$ in DCM.

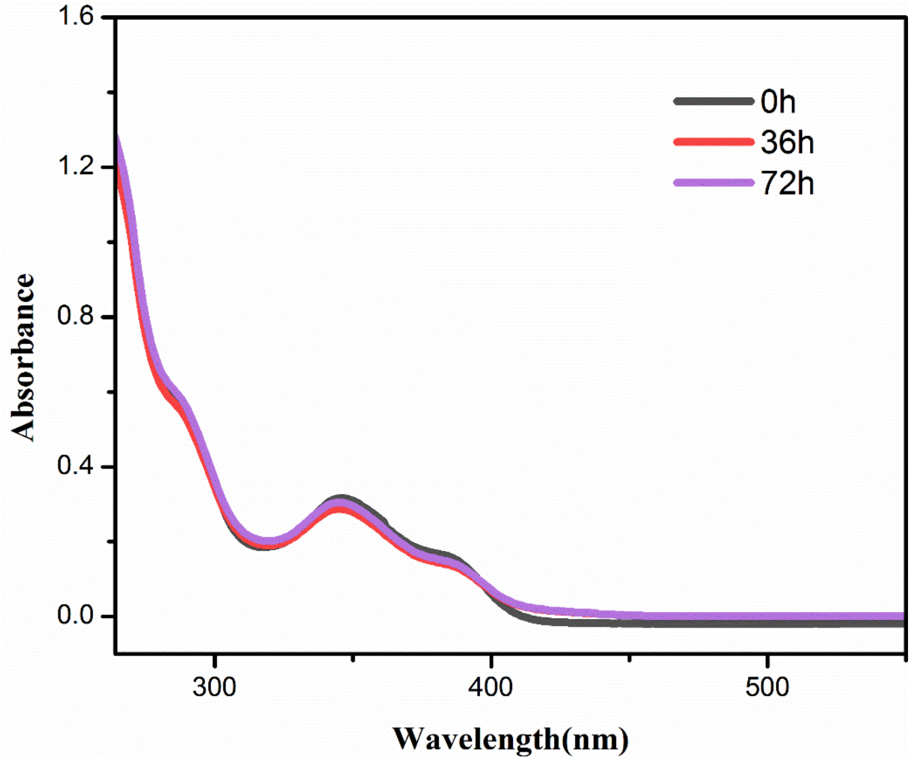

Supplementary Figure 16. UV–vis spectra tracking of $Cu_3NC^{(NHC)}$ in DCM (0-72 h).

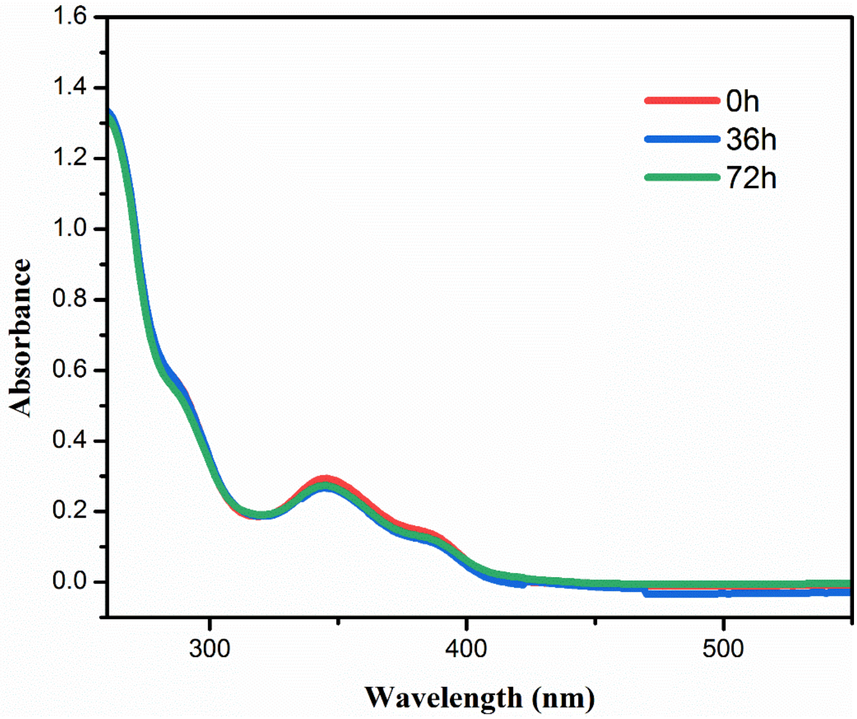

Supplementary Figure 17. UV–vis spectra tracking of $Cu_3NC^{(NHC)}$-$HNBn_2$ (1:3) in DCM (0-72 h).

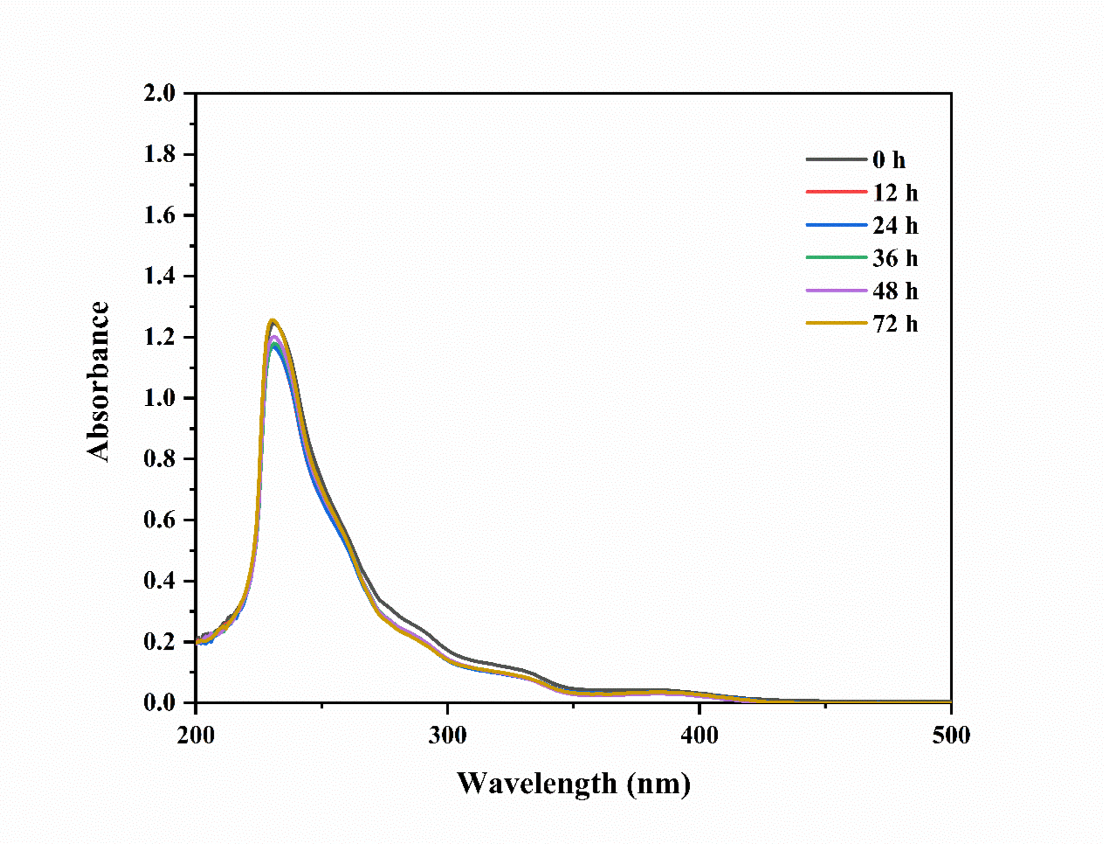

Supplementary Figure 18. UV–vis spectra tracking of $Cu_3NC^{(BINAP)}$ in DCM (0-72 h).

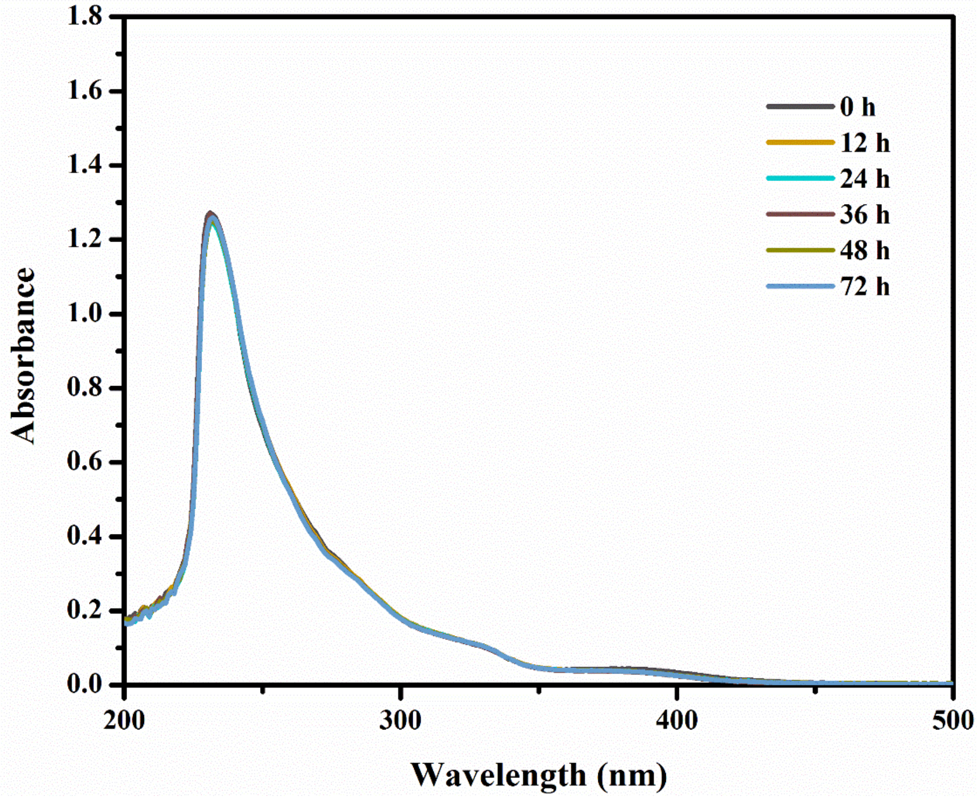

Supplementary Figure 19. UV–vis spectra tracking of $Cu_3NC^{(BINAP)}$-$HNBn_2$ (1:3) in DCM (0-72 h).

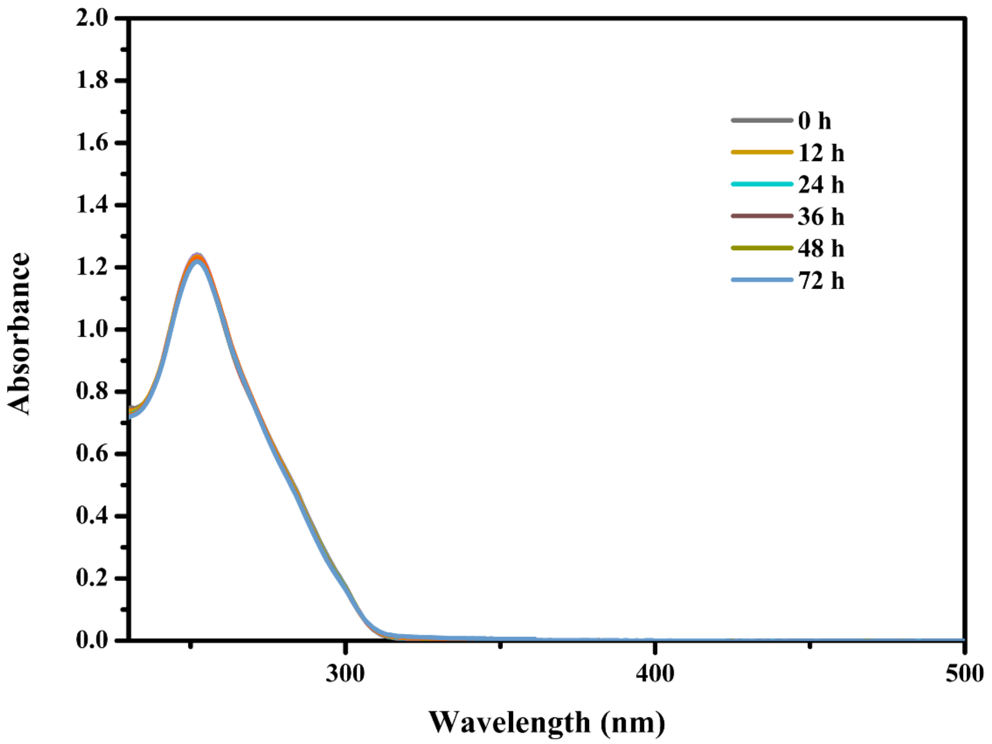

Supplementary Figure 20. UV–vis spectra tracking of $Cu_3NC^{(Pz)}$ in DCM (0-72 h).

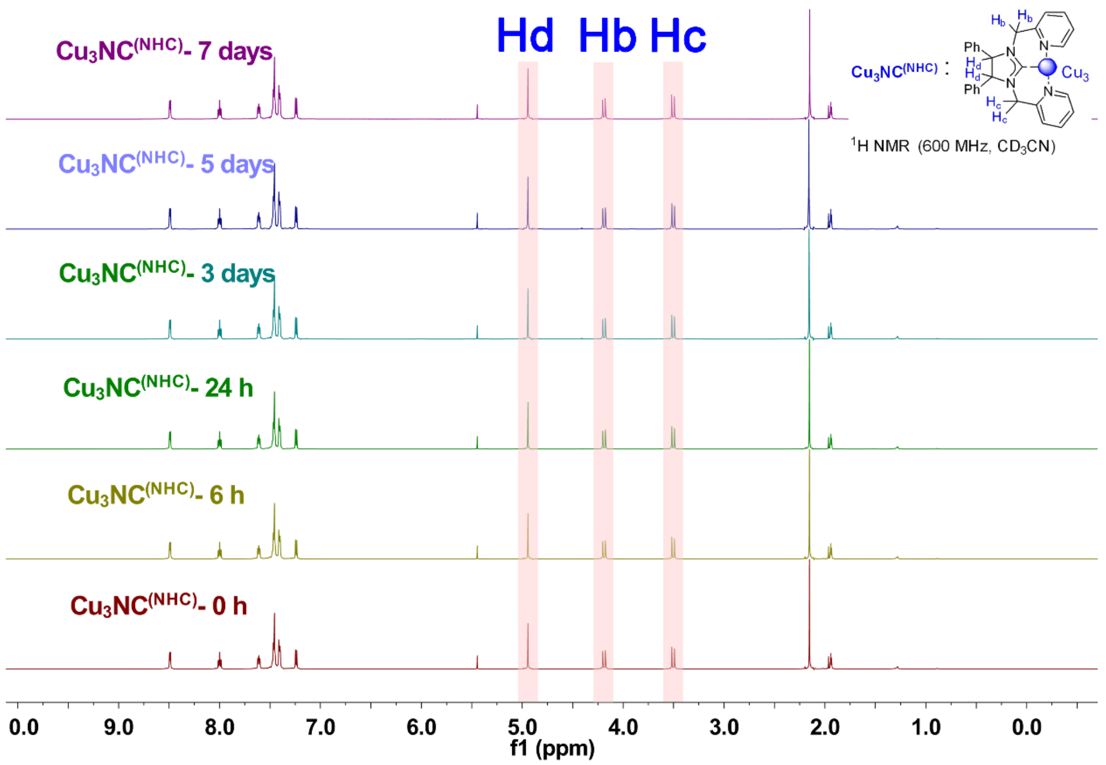

Supplementary Figure 21. ^{1}H NMR spectra tracking of $Cu_3NC^{(NHC)}$ in MeCN-d_3 (0-7 days).

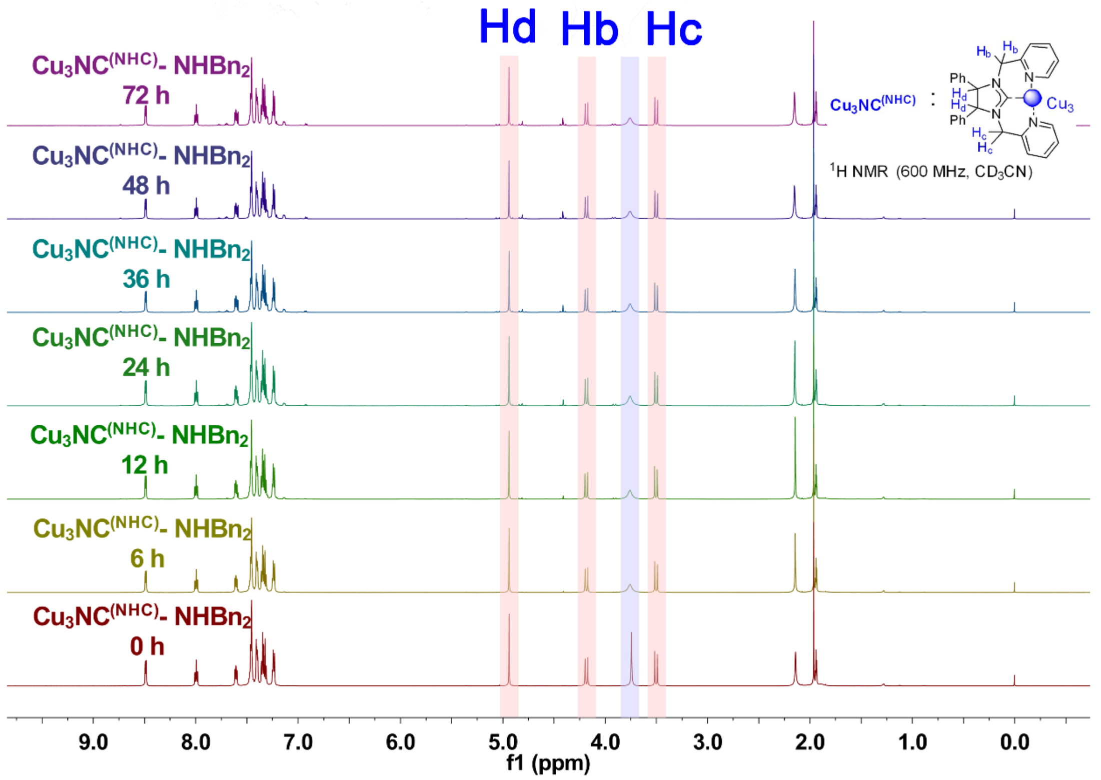

Supplementary Figure 22. ^{1}H NMR spectra tracking of $Cu_3NC^{(NHC)}$-$HNBn_2$ (1:3) in MeCN-d_3 (0-72 h).

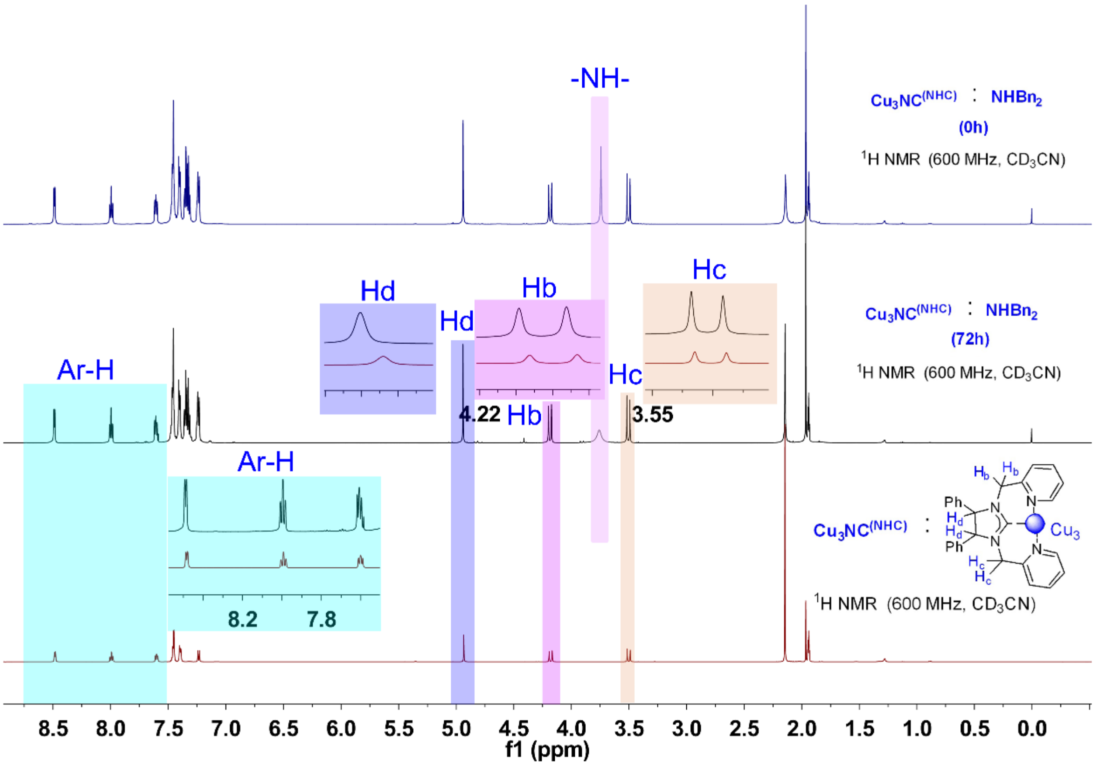

Supplementary Figure 23. ^{1}H NMR spectra tracking of $Cu_3NC^{(NHC)}$-$HNBn_2$ (1:3) in MeCN-d_3 (0-72 h).

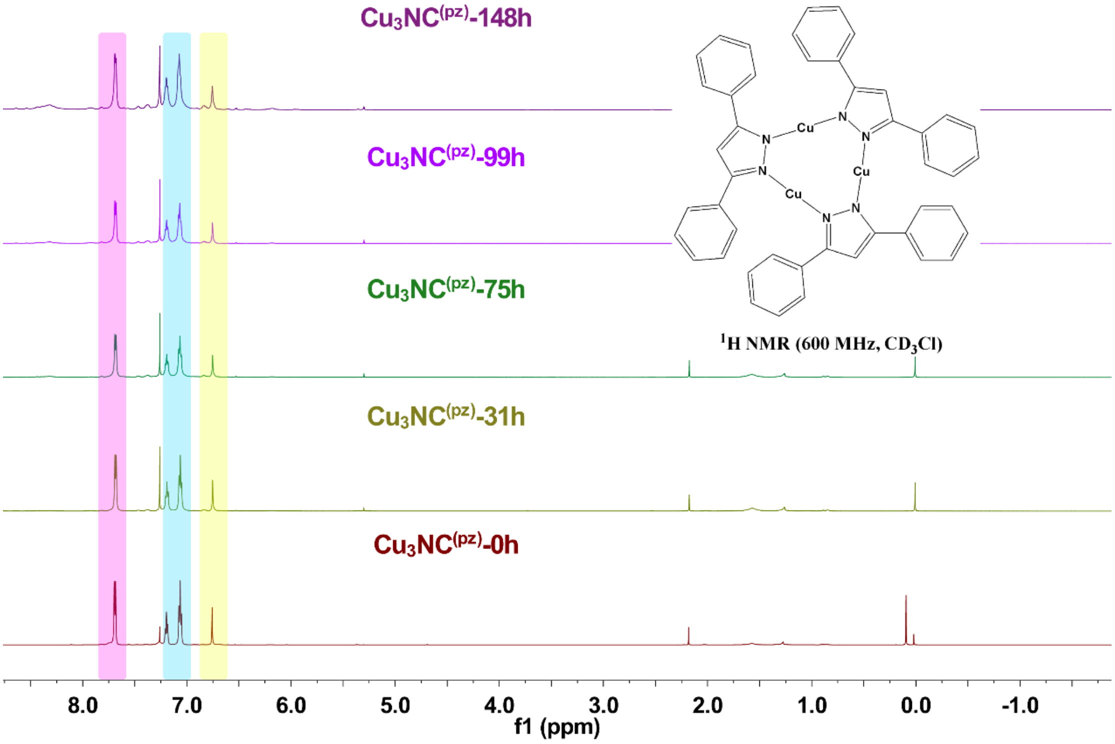

Supplementary Figure 24. ^{1}H NMR spectra tracking of $Cu_3NC^{(Pz)}$ in CD_3Cl (0-148 h).

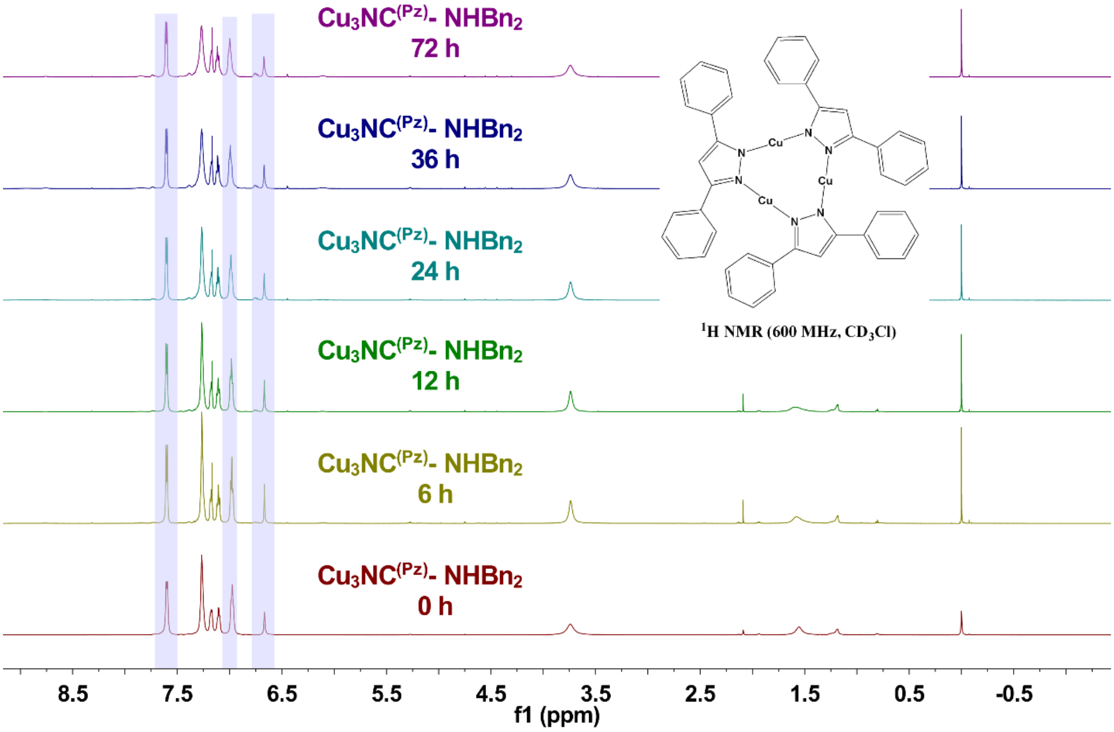

Supplementary Figure 25. ^{1}H NMR spectra tracking of $Cu_3NC^{(NHC)}$-$HNBn_2$ (1:3) in CD_3Cl (0-72 h).

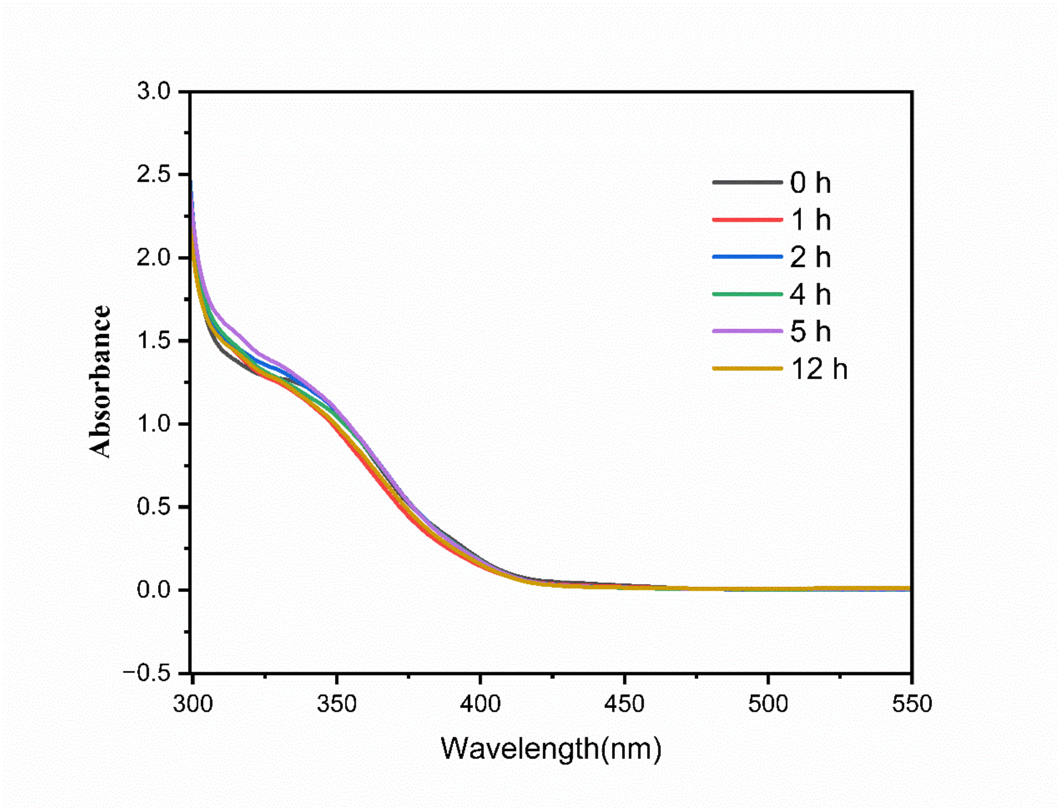

Supplementary Figure 26. *In situ* UV–vis spectra tracking of the $Cu_3NC^{(NHC)}$ catalyzed A^3 coupling reaction for 12 h in DCM.

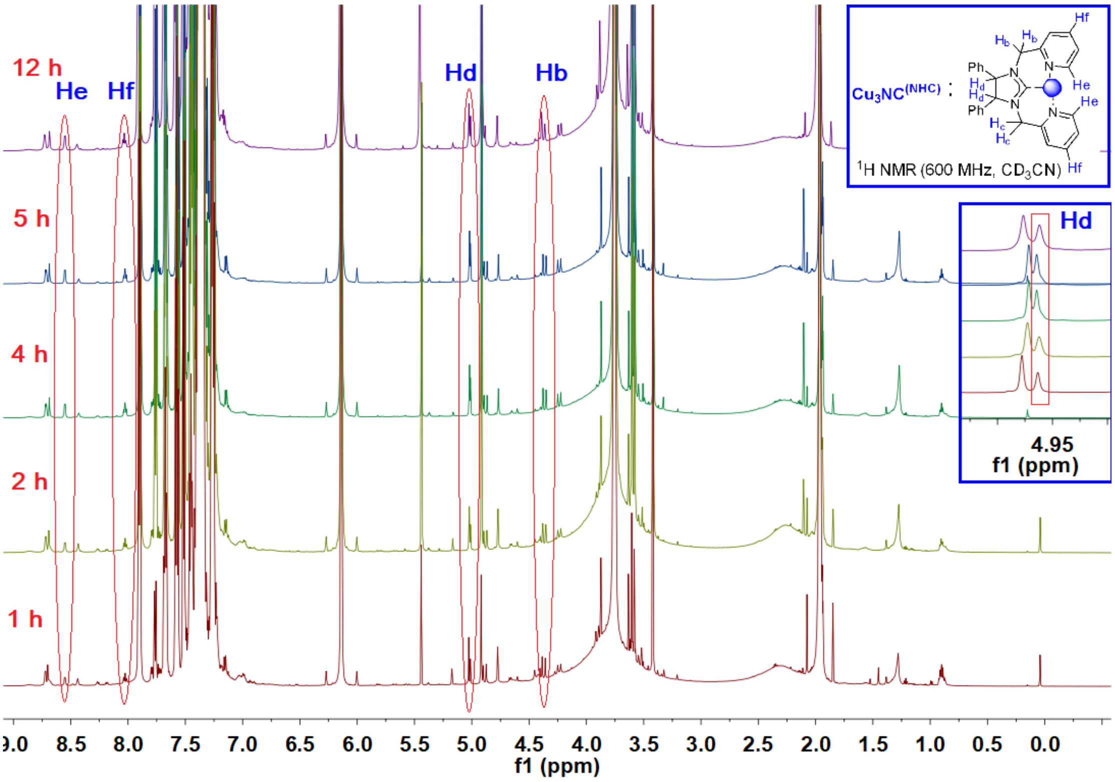

Supplementary Figure 27. *In situ* ^{1}H NMR spectra tracking of the $Cu_3NC^{(NHC)}$ catalyzed A^3 coupling reaction for 12 h in DCM.

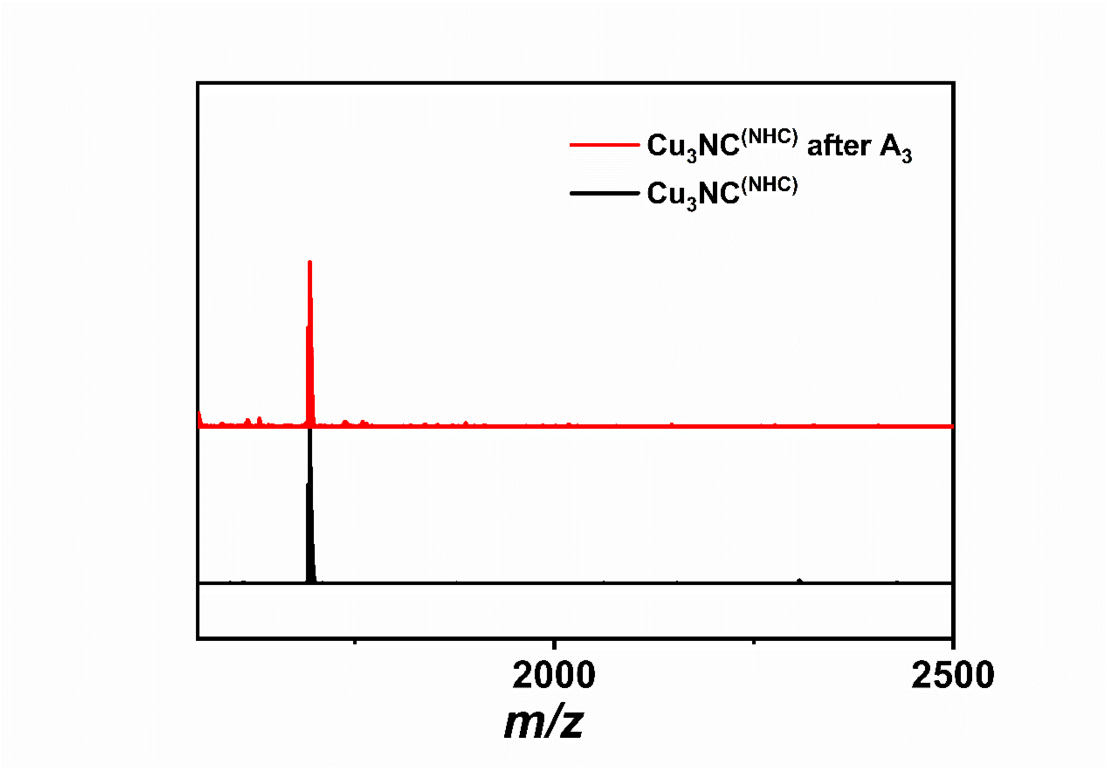

Supplementary Figure 28. ESI–MS spectra of the $Cu_3NC^{(NHC)}$ dissolved in DMF/DCM solution and measured in the positive mode after catalytic A^3 coupling reaction.

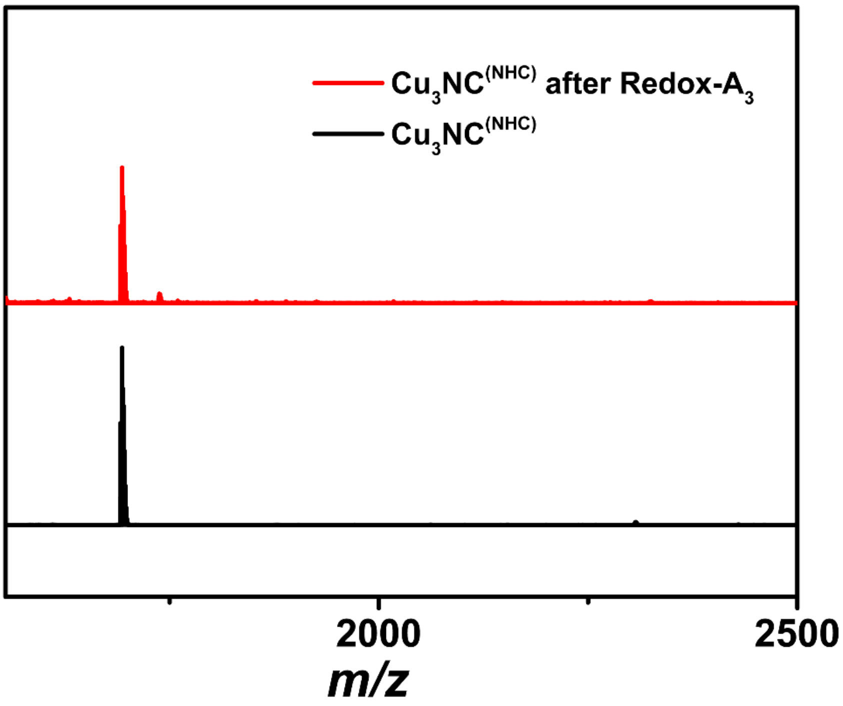

Supplementary Figure 29. ESI–MS spectra of the Cu$_3$NC$^{(NHC)}$ dissolved in DMF/DCM solution and measured in the positive mode after catalytic redox-A^3 coupling reaction.

General Procedure for Cu$_3$NC$^{(NHC)}$-Catalyzed the A^3 Coupling Reaction and the Redox-A^3 Coupling Reaction

General Procedure for Cu Catalysts-Catalyzed the A^3 Coupling Reaction (Method A).

Under N_2 atmosphere, Cu catalysts (2.5 mol%-5.0 mol%), activated MS 4Å sieves (600 mg) and dry DCM (1.5 mL) were added into the dry Schlenk tube. The reaction mixture was stirred at room temperature for 5 minutes. Aldehydes 1 (0.2 mmol, 1.0 eq) and alkynes 2 (0.2 mmol, 1.0 eq) were then added to the stirred mixture. The reaction mixture was stirred at room temperature for 5 minutes. Amines 3 (0.3 mmol, 1.5 eq) and dry DCM (0.5 mL, 0.1 M) were added into the stirred mixture of the Schlenk tube. The reaction was stirred at room temperature for 5 h-12 h or 24 h. The reactions were monitored by TLC. When aldehydes 1 and alkynes 2 were consumed, the reactions were quenched and concentrated. The crude products were then purified by column chromatography to give the target molecules.

General Procedure for Cu$_3$NC$^{(NHC)}$-Catalyzed the A^3 Coupling Reaction (Method B).

Under N_2 atmosphere, the catalyst of Cu$_3$NC$^{(NHC)}$ (2.5 mol%), activated MS 4Å sieves (600 mg) and dry DCM (1.5 mL) were added into the dry Schlenk tube. The reaction mixture was stirred at room temperature for 5 minutes. Aldehydes 1 (0.2 mmol, 1.0 eq)

and alkynes 2 (0.2 mmol, 1.0 eq) were then added to the stirred mixture. The reaction mixture was stirred at room temperature for 5 minutes. Amines 3 (0.3 mmol, 1.5 eq) and dry DCM (0.5 mL, 0.1 M) were added into the stirred mixture of the Schlenk tube. The reactions were stirred at room temperature for 12 h. The reactions were monitored by TLC. When aldehydes 1 and alkynes 2 were consumed, the reactions were quenched and concentrated. The crude products were then purified by column chromatography to give the target molecules.

General Procedure for $Cu_3NC^{(NHC)}$-Catalyzed the A_3 Coupling Reaction (Method C).

Under N_2 atmosphere, the catalyst of $Cu_3NC^{(NHC)}$ (2.5 mol%), activated MS 4Å sieves (600 mg) and dry DCM (1.5 mL) were added into the dry Schlenk tube. The reaction mixture was stirred at room temperature for 5 minutes. Aldehydes 1 (0.1 mmol or 0.2mmol) and alkynes 2 (0.2 mmol or 0.1mmol) were then added to the stirred mixture. The reaction mixture was stirred at room temperature for 5 minutes. Amines 3 (0.3 mmol) and dry DCM (0.5 mL, 0.1 M) were added into the stirred mixture of the Schlenk tube. The reactions were stirred at room temperature for 12 h. The reactions were monitored by TLC. When aldehydes 1 and alkynes 2 were consumed, the reactions were quenched and concentrated. The crude products were then purified by column chromatography to give the target molecules.

General Procedure for Cu Catalysts-Catalyzed the Redox-A^3 Coupling Reaction (Method D).

Under N_2 atmosphere, Cu catalysts (2.5 mol%-0.25 mol%), activated MS 4Å sieves (600 mg) and dry solvents or mixture solvents (1.5 mL) were added into the dry Schlenk tube. The reaction mixture was stirred at room temperature for 5 minutes. Aldehydes 1 (0.2 mmol, 1.0 eq) and alkynes 2 (0.2 mmol, 1.0 eq) were then added to the stirred mixture. The reaction mixture was stirred at room temperature for 5 minutes. Amines 3 (0.3 mmol, 1.5 eq) and dry solvents or mixture solvents (0.5 mL, 0.1 M) were added into the stirred mixture of the Schlenk tube. The reactions were stirred at room temperature (or 50 °C by heating block) for 6 h-48 h. The reactions were monitored by TLC. When aldehyde 1 and alkynes 2 were consumed, the reactions were quenched and concentrated. The crude products were then purified by column chromatography to give the target molecules.

General Procedure for $Cu_3NC^{(NHC)}$-Catalyzed the Redox-A^3 Coupling Reaction (Method E).

Under N_2 atmosphere, the catalyst of $Cu_3NC^{(NHC)}$ (0.25 mol%), activated MS 4Å sieves (600 mg) and dry DCM (1.5 mL) were added into the dry Schlenk tube. The reaction mixture was stirred at room temperature for 5 minutes. Aldehydes 1 (0.2 mmol, 1.0 eq) and alkynes 2 (0.2 mmol, 1.0 eq) were then added to the stirred mixture. The reaction mixture was stirred at room temperature for 5 minutes. 1,2,3,4-tetrahydro-2-isoquinolines 3 (0.3 mmol, 1.5 eq) and dry DCM (0.5 mL, 0.1 M) were added into the

stirred mixture of the Schlenk tube. The reactions were stirred at room temperature for 40 h. The reactions were monitored by TLC. When aldehydes 1 and alkynes 2 were consumed, the reactions were quenched and concentrated. The crude products were then purified by column chromatography to give the target molecules.

General Procedure for $Cu_3NC^{(NHC)}$-Catalyzed the A^3 Coupling Reaction in 2 mmol scale.

Under N_2 atmosphere, the catalyst of $Cu_3NC^{(NHC)}$ (46 mg, 1.2 mol%), activated MS 4Å sieves (5.0 g) and dry DCM (15.0 mL) were added into the dry Schlenk tube. The reaction mixture was stirred at room temperature for 5 minutes. Benzaldehyde 1 (213 mg, 2.0 mmol, 1.0 eq) and phenylacetylene 2 (205.0 mg, 2.0 mmol, 1.0 eq) were then added to the stirred mixture. The reaction mixture was stirred at room temperature for 5 minutes. Dibenzylamine 3 (592.0 mg, 3.0 mmol, 1.5 eq) and dry DCM (5.0 mL, 0.1 M) were added into the stirred mixture of Schlenk tube. The reaction was stirred at room temperature for 12 h. The reaction was monitored by TLC. When benzaldehyde 1 and phenylacetylene 2 were consumed, the reaction was quenched and concentrated. The crude product was then purified by column chromatography to give the N, N-dibenzyl-1,3-diphenylprop-2-yn-1-amine (**4a**) as colorless oil with overall isolated yield: 95% (735.7 mg).

General Procedure for $Cu_3NC^{(NHC)}$-catalyzed the Redox-A^3 Coupling Reaction in 2 mmol scale

Under N_2 atmosphere, the catalyst of $Cu_3NC^{(NHC)}$ (9.2 mg, 0.25 mol%), activated MS 4Å sieves (5.0 g) and dry DCM (15.0 mL) were added into the dry Schlenk tube. The reaction mixture was stirred at room temperature for 5 minutes. Benzaldehyde 1 (213 mg, 2.0 mmol, 1.0 eq) and phenylacetylene 2 (205.0 mg, 2.0 mmol, 1.0 eq) were then added to the stirred mixture. The reaction mixture was stirred at room temperature for 5 minutes. 1,2,3,4-Tetrahydro-2-isoquinoline 3 (400.0 mg, 3.0 mmol, 1.5 eq) and dry DCM (5.0 mL, 0.1 M) were added into the stirred mixture of Schlenk tube. The reaction was stirred at room temperature for 40 h. The reaction was monitored by TLC. When benzaldehyde 1 and phenylacetylene 2 were consumed, the reaction was quenched and concentrated. The crude product was then purified by column chromatography (PE-EA, v/v 10/1) to give the 2-benzyl-1-(phenylethynyl)-1,2,3,4-tetrahydroisoquinoline (**5a**) as pale-yellow oil with overall isolated yield: 96% (620.5 mg).

Recycling Procedure for $Cu_3NC^{(NHC)}$-Catalyzed the A^3 Coupling Reaction.

Under N_2 atmosphere, the catalyst of $Cu_3NC^{(NHC)}$ (2.5 mol%), activated MS 4Å sieves (600 mg) and dry DCM (1.5 mL) were added into the dry Schlenk tube. The reaction mixture was stirred at room temperature for 5 minutes. Aldehydes 1 (0.2 mmol, 1.0 eq) and alkynes 2 (0.2 mmol, 1.0 eq) were then added to the stirred mixture. The reaction mixture was stirred at room temperature for 5 minutes. Amines 3 (0.3 mmol, 1.5 eq) and dry DCM (0.5 mL, 0.1 M) were added into the stirred mixture of Schlenk tube. The reactions were stirred at room temperature for 12 h. Conversions and yields were

determined by ^{1}H NMR using 1,3,5-trimethoxybenzene as an internal standard. Then the reactants [aldehydes 1 (0.2 mmol, 1.0 eq), alkynes 2 (0.2 mmol, 1.0 eq) and amines 3 (0.3 mmol, 1.5 eq)] were injected to the reaction system five consecutive times, subsequently. Every conversion and yield were determined by ^{1}H NMR using 1,3,5-trimethoxybenzene as an internal standard.

Recycling Procedure for $Cu_3NC^{(NHC)}$-Catalyzed the Redox-A^3 Coupling Reaction.

Under N_2 atmosphere, the catalyst of $Cu_3NC^{(NHC)}$ (0.25 mol%), activated MS 4Å sieves (600 mg) and dry DCM (1.5 mL) were added into the dry Schlenk tube. The reaction mixture was stirred at room temperature for 5 minutes. Aldehydes 1 (0.2 mmol, 1.0 eq) and alkynes 2 (0.2 mmol, 1.0 eq) were then added to the stirred mixture. The reaction mixture was stirred at room temperature for 5 minutes. The 1,2,3,4-Tetrahydro-2-isoquinolines 3 (0.3 mmol, 1.5 eq) and dry DCM (0.5 mL, 0.1 M) were added into the stirred mixture of the Schlenk tube. The reactions were stirred at room temperature for 40 h. Conversions and yields were determined by ^{1}H NMR using 1,3,5-trimethoxybenzene as an internal standard. Then the reactants [aldehydes 1 (0.2 mmol, 1.0 eq), alkynes 2 (0.2 mmol, 1.0 eq) and amines 3 (0.3 mmol, 1.5 eq)] were injected to the reaction system two consecutive times, subsequently. Every conversion and yield were determined by ^{1}H NMR using 1,3,5-trimethoxybenzene as an internal standard.

Recycling Procedure for $Cu_3NC^{(NHC)}$/Activated Carbon-Catalyzed the A^3 Coupling Reaction.

Under N_2 atmosphere, the $Cu_3NC^{(NHC)}$ (9.2 mg) were dissolved in 2 mL dried DCM, which was added dropwise into 5 mL dried DCM solution of 100 mg activated carbon (Vulcan XC-72 carbon black) under vigorous stirring. After stirring for 6 h at room temperature, the solution was centrifuged at 8000 rpm for 5 minutes, and the activated carbon was washed with dried DCM three times and then dried in vacuum.

Under N_2 atmosphere, the $Cu_3NC^{(NHC)}$/XC-72 (2.5 mol%), activated MS 4Å sieves (600 mg) and dry DCM (1.5 mL) were added into the dry Schlenk tube. The reaction mixture was stirred at room temperature for 5 minutes. Aldehydes 1 (0.2 mmol, 1.0 eq) and alkyne 2 (0.2 mmol, 1.0 eq) were then added to the stirred mixture. The reaction mixture was stirred at room temperature for 5 minutes. Amine 3 (0.3 mmol, 1.5 eq) and dry DCM (0.5 mL, 0.1 M) were added into the stirred mixture of the Schlenk tube. The reaction was stirred at room temperature for 12 h. The activated MS 4Å sieves were taken out and the reaction mixture was centrifuged at 8000 rpm for 5 minutes and purified to give the target molecule. The $Cu_3NC^{(NHC)}$/XC-72 was washed with hexane three times then dried in vacuum and used again in the next round of recycling. Conversion and yield were determined by ^{1}H NMR using 1,3,5-trimethoxybenzene as an internal standard.

The $Cu_3NC^{(NHC)}$/XC-72 as a catalyst was tested to catalyze the A^3 coupling reaction under optimized conditions, affording the desired typical propargylamine product with excellent yields (98%) for the first time. The activated MS 4Å sieves were taken out

and the reaction mixture was centrifuged at 8000 rpm for 5 minutes and purified to give the target molecule. The $Cu_3NC^{(NHC)}$/XC-72 was washed with hexane three times then dried in vacuum and used again in the next round of recycling. The result indicates that the $Cu_3NC^{(NHC)}$/XC-72 catalyzed the A^3 coupling reaction under the optimized conditions, affording propargylamine product with poor yields (35%) for the second time. To investigate the origin of the decrease in A^3 coupling reaction yield, the reaction process was monitored by the *in situ* ^1H NMR spectrum that the $Cu_3NC^{(NHC)}$/XC-72 as a catalyst, for the first time, catalyzed the A^3 coupling reaction under the optimized conditions. The characteristic peaks of $Cu_3NC^{(NHC)}$ were found in the *in situ* ^1H NMR spectrum, indicating that $Cu_3NC^{(NHC)}$ is very easy to separate from activated carbon to lead to the decrease in A^3 coupling reaction yield on the second time.

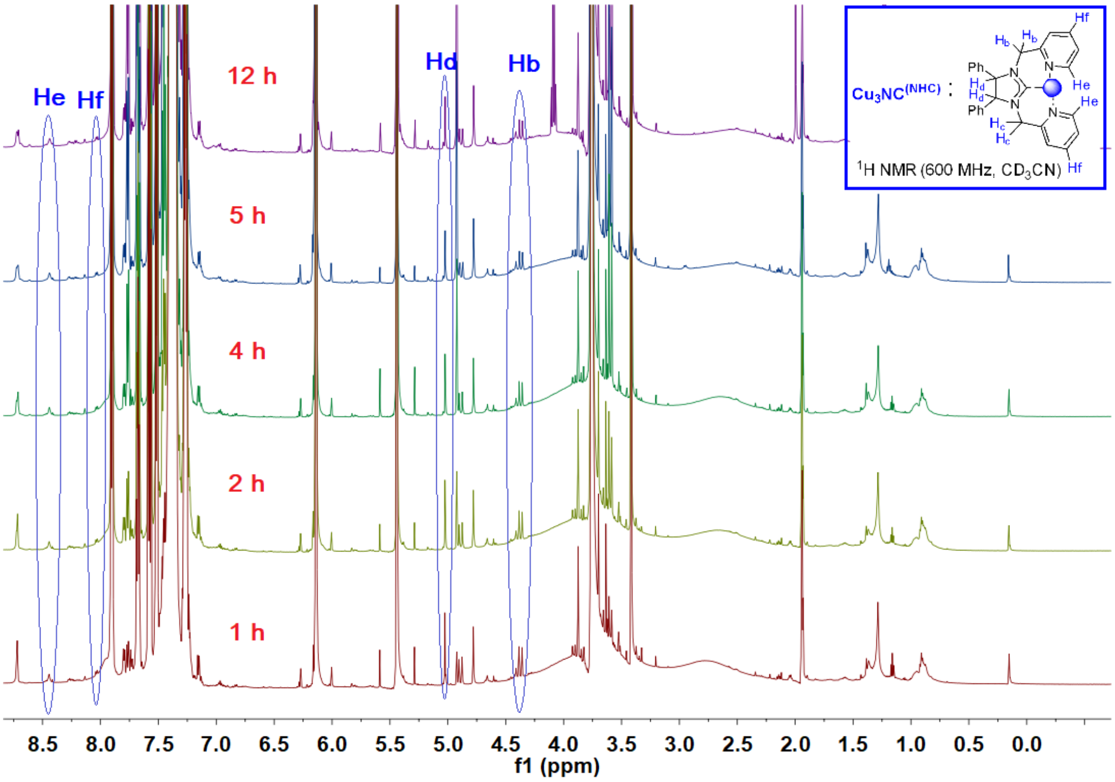

Supplementary Figure 30. *In situ* ^1H NMR spectra tracking of the $Cu_3NC^{(NHC)}$/XC-72 catalyzed A^3 coupling reaction for 12 h in DCM, for the first time.

Investigation on Lowest Catalyst Loading of the $Cu_3NC^{(NHC)}$ for both Organic Transformations

Supplementary Table 1. Optimization of catalytic performance and investigation on lowest catalyst loading of the $Cu_3NC^{(NHC)}$ for the A^3 coupling reaction of benzaldehyde, phenylacetylene and dibenzylamine under the identical conditions[a].

A^3 coupling reaction

Entry	Time	[Cat.]	Yield[c]	TON[d]
1	12 h	2.5 mol%	99%	39.6
2	12 h	0.25 mol%	49%	196
3[b]	12 h	0.025 mol%	13%	520
4[b]	60 h	0.025 mol%	56%	2240
5[b]	120 h	0.025 mol%	75%	3000
6[b]	240 h	0.025 mol%	95%	3800

[a]General reaction conditions: 1 (0.2 mmol, 1.0 equiv.), 2 (0.2 mmol, 1.0 equiv.), 3 (0.3 mmol, 1.5 equiv.), [Cu] catal., activated MS 4Å sieves (600 mg) in dry solvent (2.0 mL) under N_2 at room temperature. [b]General reaction conditions: 1 (2.0 mmol, 1.0 equiv.), 2 (2.0 mmol, 1.0 equiv.), 3 (2.0 mmol, 1.0 equiv.), [Cu] catal. (5×10^{-4} mmol), activated MS 4Å sieves in dry solvent under N_2 at room temperature. [c]Yield was determined by ^{1}H NMR using 1,3,5-trimethoxybenzene as an internal standard. [d]TON = (Moles of reactants) × (Yield %) / (Moles of catalysts).

Supplementary Table 2. Optimization of catalytic performance and investigation on lowest catalyst loading of the $Cu_3NC^{(NHC)}$ for the redox-A^3 coupling reaction of benzaldehyde, phenylacetylene and 1,2,3,4-tetrahydro-2-isoquinoline under the identical conditions[a].

Redox-A^3 coupling reaction

Entry	Time	[Cat.]	Yield[c]	TON[d]
1	40 h	0.25 mol%	98%	392
2[b]	40 h	0.025 mol%	30%	1200
3[b]	120 h	0.025 mol%	46%	1840

[a]General reaction conditions: 1 (0.2 mmol, 1.0 equiv.), 2 (0.2 mmol, 1.0 equiv.), 3 (0.3 mmol, 1.5 equiv.), [Cu] catal., activated MS 4Å sieves (600 mg) in dry solvent (2.0 mL) under N_2 at room temperature. [b]General reaction conditions: 1 (2.0 mmol, 1.0 equiv.), 2 (2.0 mmol, 1.0 equiv.), 3 (2.0 mmol, 1.0 equiv.), [Cu] catal. (5×10^{-4} mmol), activated MS 4Å sieves in dry solvent under N_2 at room temperature. [c]Yield was determined by ^{1}H NMR using 1,3,5-trimethoxybenzene as an internal standard. [d]TON = (Moles of reactants) × (Yield %) / (Moles of catalysts).

Catalytic Mechanism Studies by a series of Control Experiments

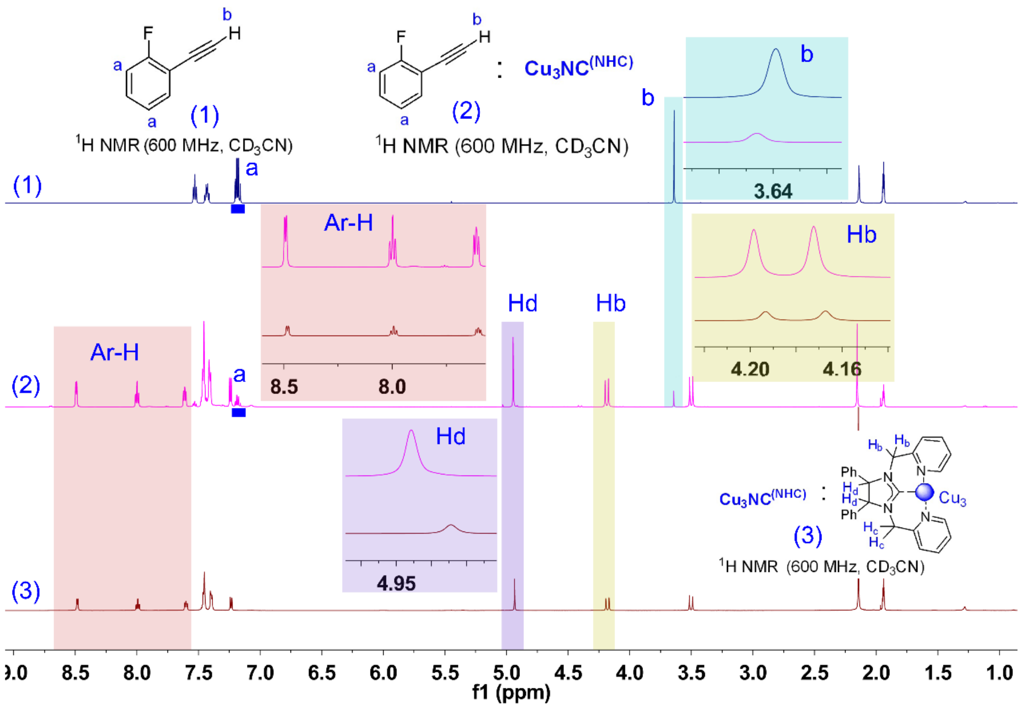

Supplementary Figure 31. ^{1}H NMR spectra of 2-fluorophenylacetylene (top), a mixture of the Cu$_3$NC$^{(NHC)}$ and 2-fluorophenylacetylene (middle) and the Cu$_3$NC$^{(NHC)}$ (bottom) in CD$_3$CN.

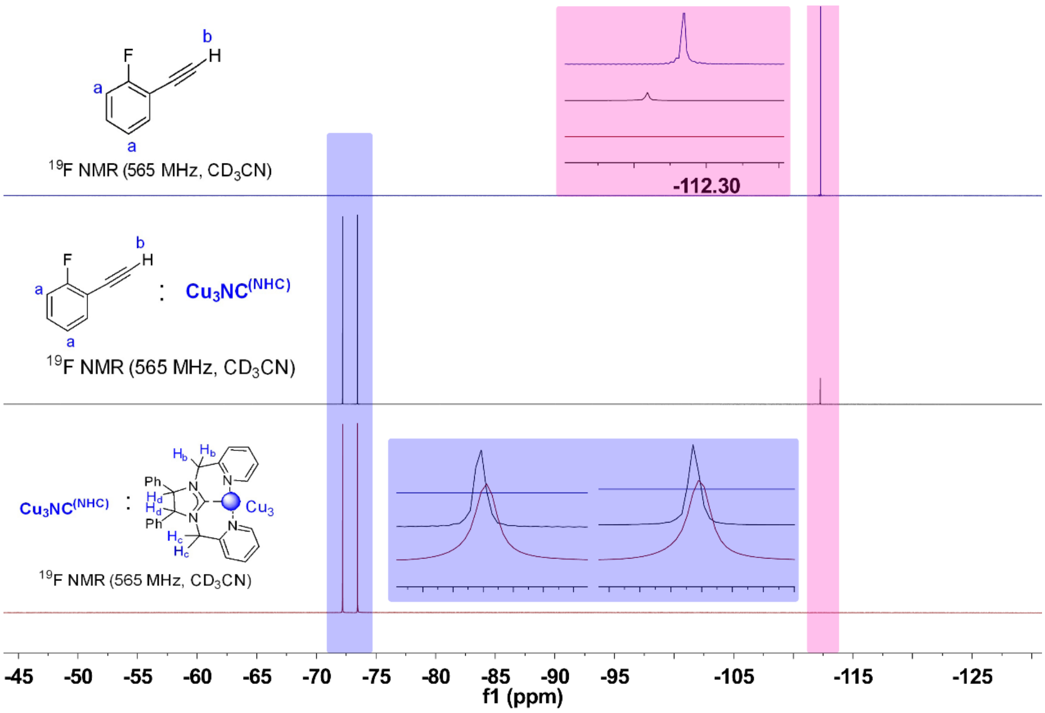

Supplementary Figure 32. ^{19}F NMR spectra of 2-fluorophenylacetylene (top), a mixture of the Cu$_3$NC$^{(NHC)}$ and 2-fluorophenylacetylene (middle) and the Cu$_3$NC$^{(NHC)}$ (bottom) in CD$_3$CN.

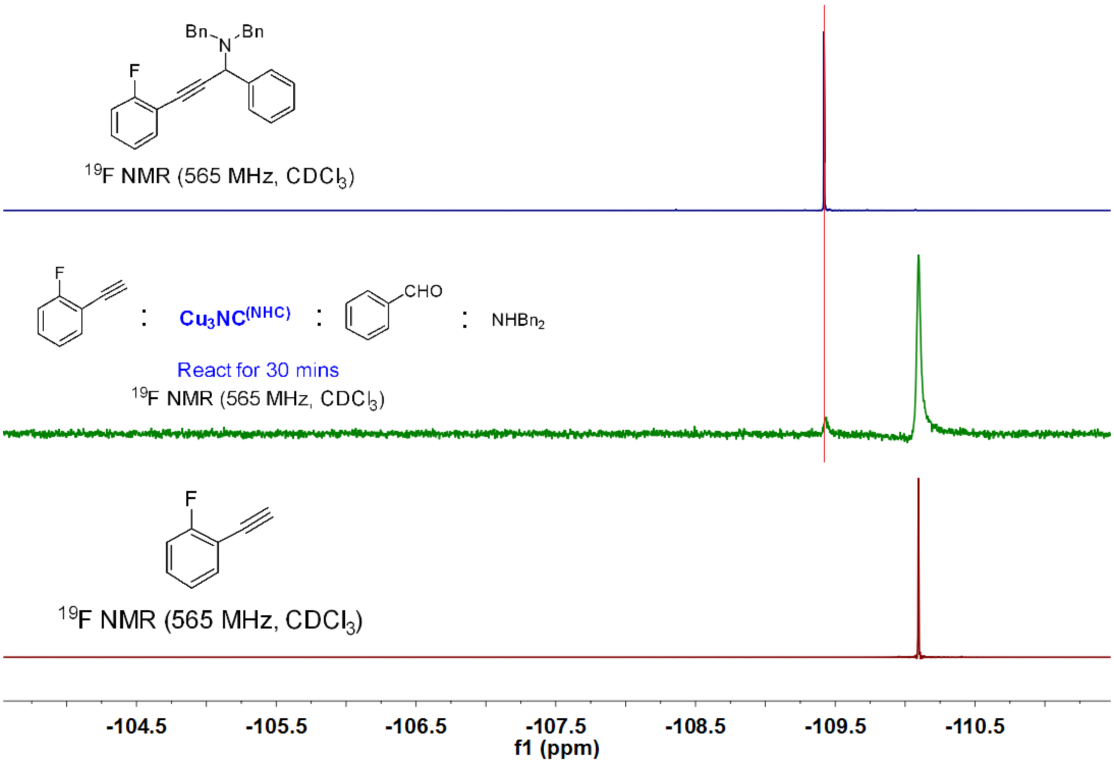

Supplementary Figure 33. ^{19}F NMR spectra of N, N-dibenzyl-3-(2-fluorophenyl)-1-phenylprop-2-yn-1-amine (4ad) (top), the Cu3NC$^{(NHC)}$ catalyzed A^3 coupling reaction for 30 mins (middle) and 2-fluorophenylacetylene (bottom) in CDCl3.

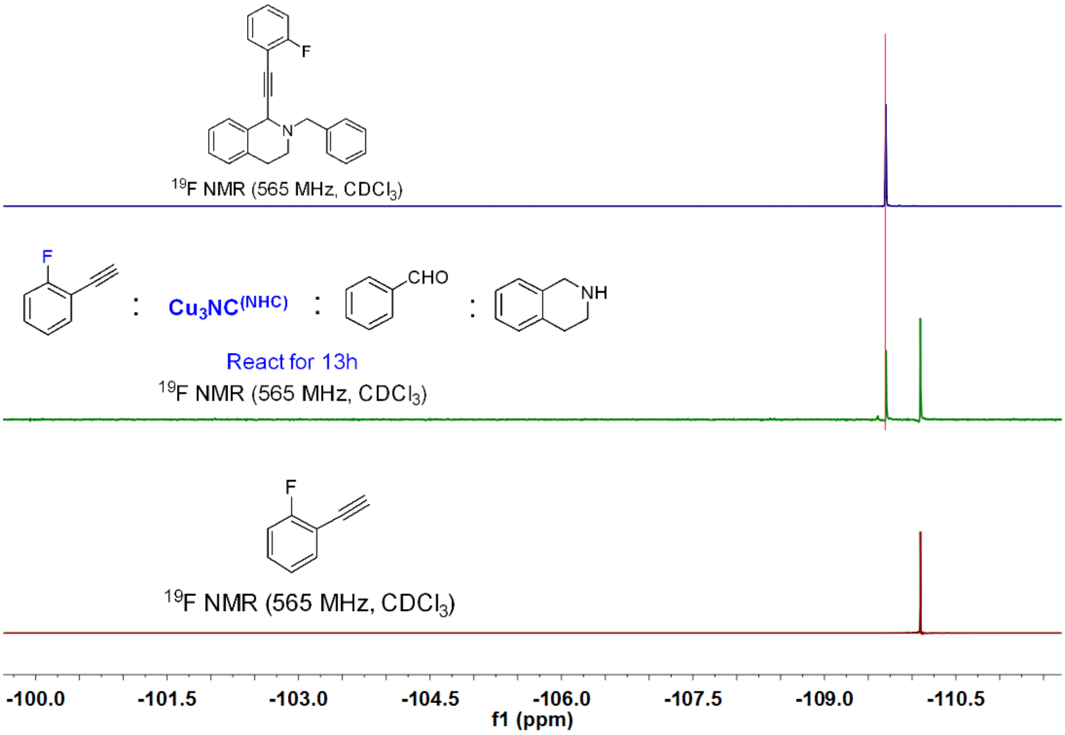

Supplementary Figure 34. ^{19}F NMR spectra of 2-benzyl-1-((2-fluorophenyl) ethynyl)-1,2,3,4-tetrahydroisoquinoline (5k) (top), the Cu3NC$^{(NHC)}$ catalyzed Redox-A^3 coupling reaction for 13 hour (middle) and 2-fluorophenylacetylene (bottom) in CDCl3.

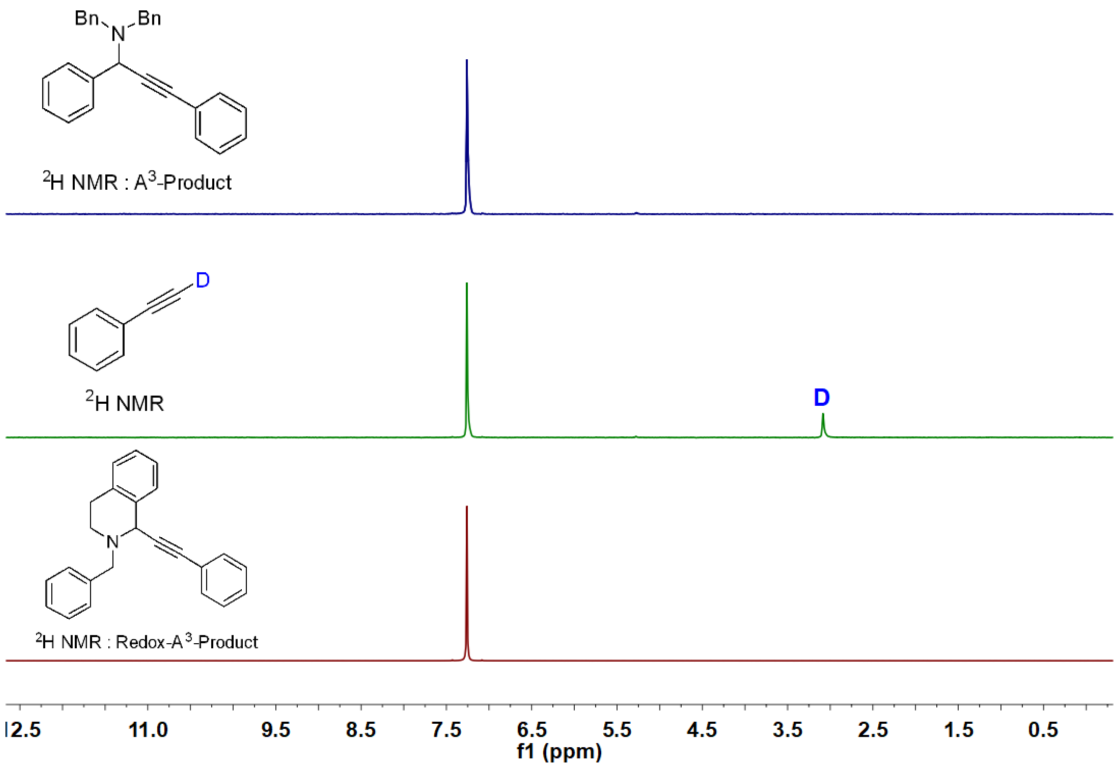

Supplementary Figure 35. ^{2}H NMR spectra of N, N-dibenzyl-1,3-diphenylprop-2-yn-1-amine (**4a**) (top), deuterated phenylacetylene (middle) and 2-benzyl-1-(phenylethynyl)-1,2,3,4-tetrahydroisoquinoline (**5a**) (bottom) in $CDCl_3$.

Catalytic Mechanism Studies by Density Functional Theory (DFT) Calculations

DFT calculations were conducted using the Gaussian 16 package, specifically at the B3LYP-D3 level of theory, where dispersion interactions were considered[(10-15)]. For carbon, oxygen, hydrogen, and nitrogen atoms, the 6-31G* basis set was employed, while the 6-311++G** basis set was utilized for copper[(16,17)]. To account for solvation effects, single-point calculations were performed using the solvation model based on density (SMD) in dichloromethane[(18)]. Vibrational frequency calculations were conducted to verify that a transition state exhibits only one imaginary frequency and a local minimum displays no imaginary frequency. Transition states connecting the relevant minima were further investigated through intrinsic reaction coordinate (IRC) calculations[(19-21)]. Corrections of -2.6 (or 2.6) kcal/mol were applied for transformations involving the conversion of two molecules to one (or one molecule to two) to obtain relative Gibbs energies at 298 K. This approach has been used in numerous previous studies to minimize the overestimation of entropy contributions[(22-30)].

According to the energetic span model, it is depicted that schematic illustration of the energy profile given the A^3 coupling reaction in Figure 6, which includes the energy profile for an additional catalytic cycle (THIQs as the amine substrate). The TS_{6-7} and Int7 represent rate-determining transition state and intermediate, respectively. We could also view the first Int7 to the second TS_{6-7} as a catalytic cycle from the Supplementary Figure 32. Therefore, the overall barrier could be calculated by $\Delta G_1 + \Delta G_2$. And we got

that ΔG_1 = 27.6 kcal mol^{-1} and ΔG_2 =5.8 kcal mol^{-1} from the Figure 6.

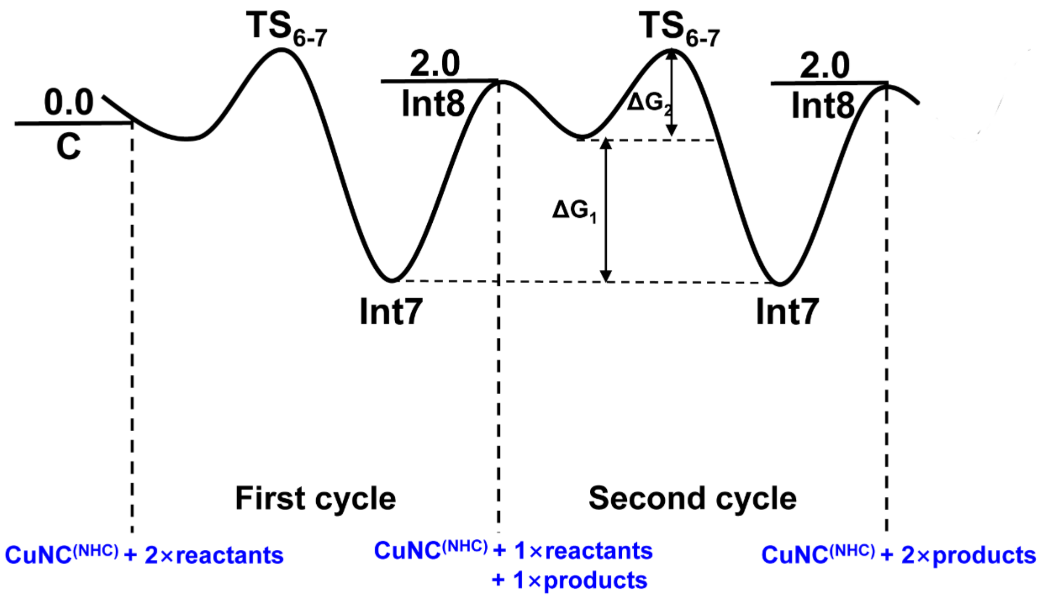

Supplementary Figure 36. Schematic illustration of the energy profile given the $Cu_3NC^{(NHC)}$ catalyzed A^3 coupling reaction in Figure 6, which includes the energy profile for an additional catalytic cycle (THIQs as the amine substrate).

According to the energetic span model, it is depicted that schematic illustration of the energy profile given the redox-A^3 coupling reaction in Figure 6, including the energy profile for an additional catalytic cycle (THIQs as the amine substrate). The $TS_{9\text{-}10}$ and Int9 represent rate-determining transition state and intermediate, respectively. We could also view the first $TS_{9\text{-}10}$ to the second Int9 as a catalytic cycle from the Supplementary Figure 33. Therefore, the overall barrier could be calculated by ΔG_3 +ΔG_4. And we obtained that ΔG_3 = 19.8 kcal mol^{-1} and ΔG_4 =6.7 kcal mol^{-1} from the Figure 6.

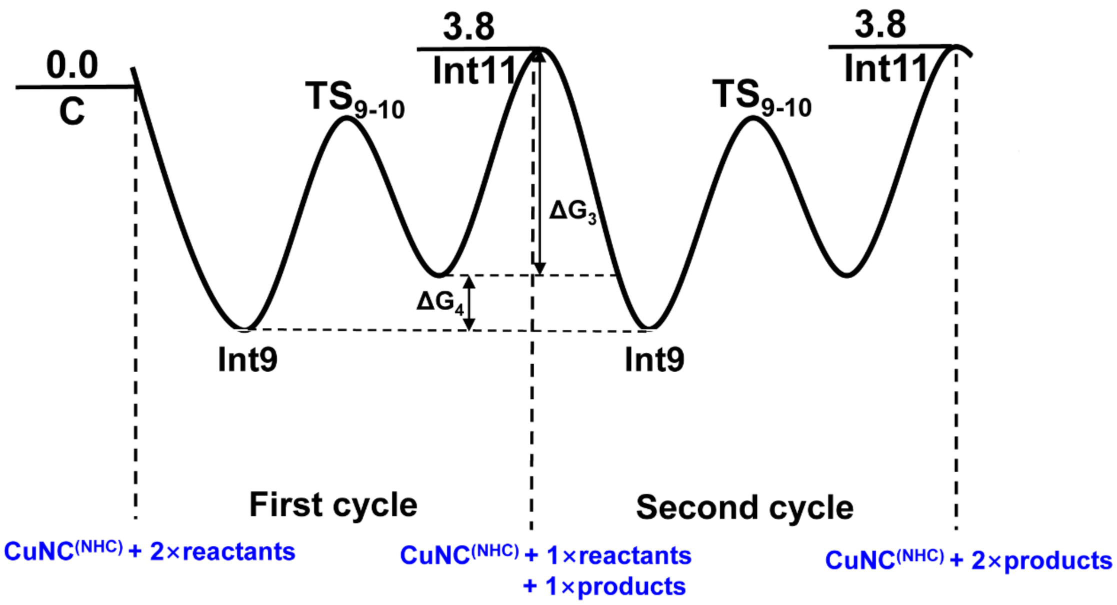

Supplementary Figure 37. Schematic illustration of the energy profile given the $Cu_3NC^{(NHC)}$ catalyzed redox-A^3 coupling reaction in Figure 6, which includes the energy profile for an additional catalytic cycle (THIQs as the amine substrate).

The Preliminary Kinetic Studies of the A^3 Coupling Reaction and Redox-A^3 Coupling Reaction

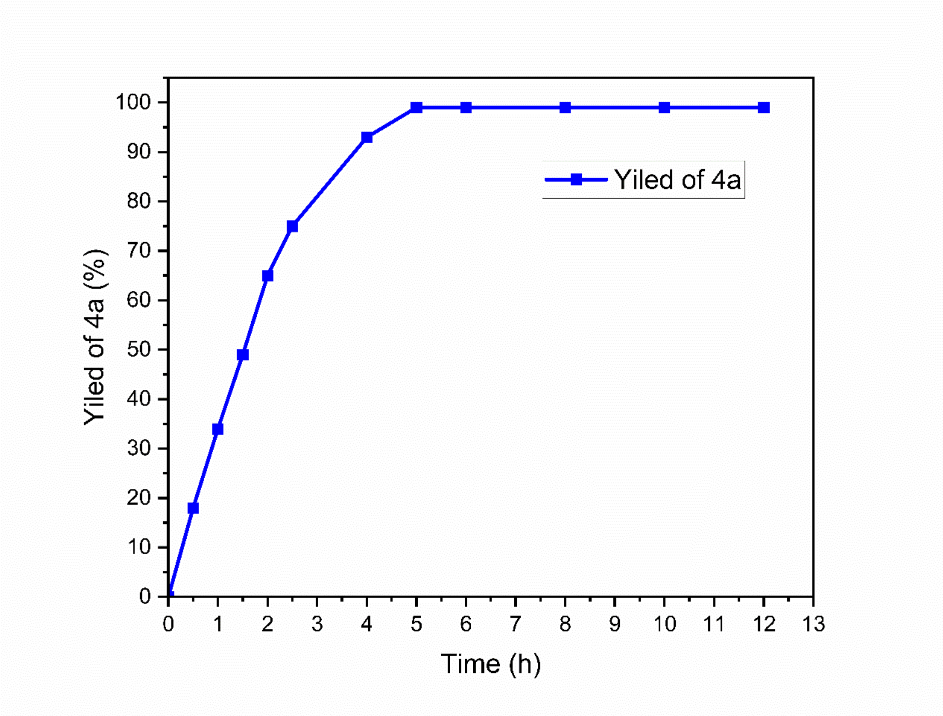

Supplementary Figure 38. Catalytic performances of 2.5 mol% $Cu_3NC^{(NHC)}$ in A^3 coupling reaction of phenylacetylene at room temperature.

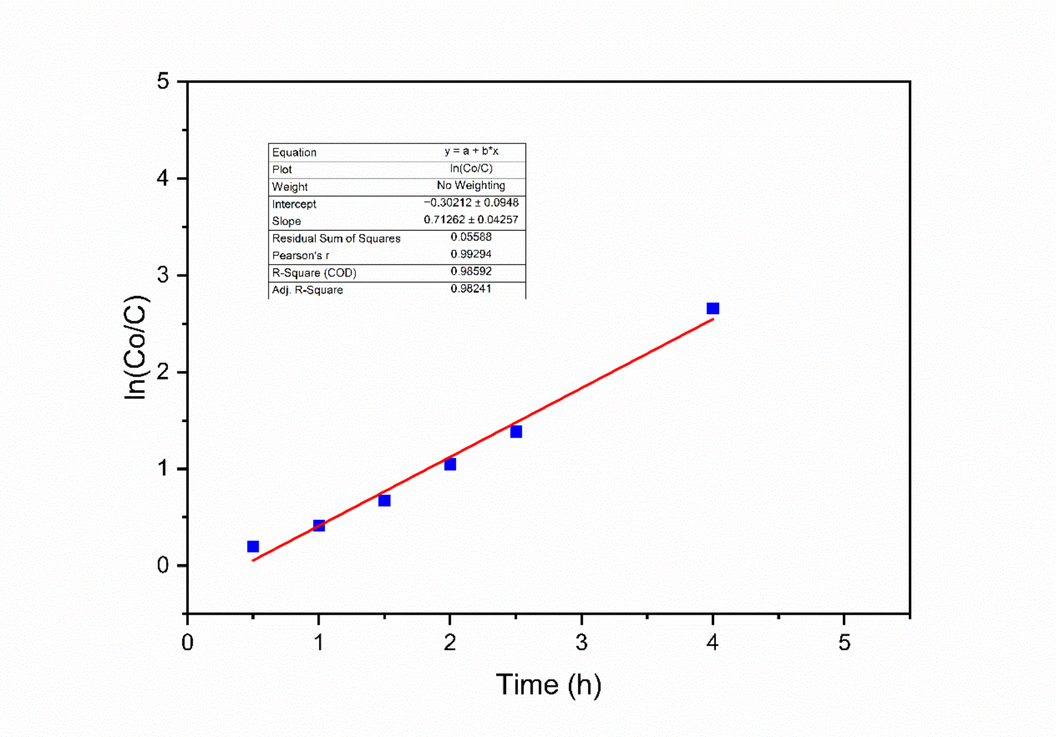

Supplementary Figure 39. Kinetic curve ($\ln(C_0/C)$ vs. t) of phenylacetylene A^3 coupling reaction by using 2.5 mol% $Cu_3NC^{(NHC)}$ as catalyst. C_0 and C are the phenylacetylene concentrations before the reaction and at a reaction time of t, respectively.

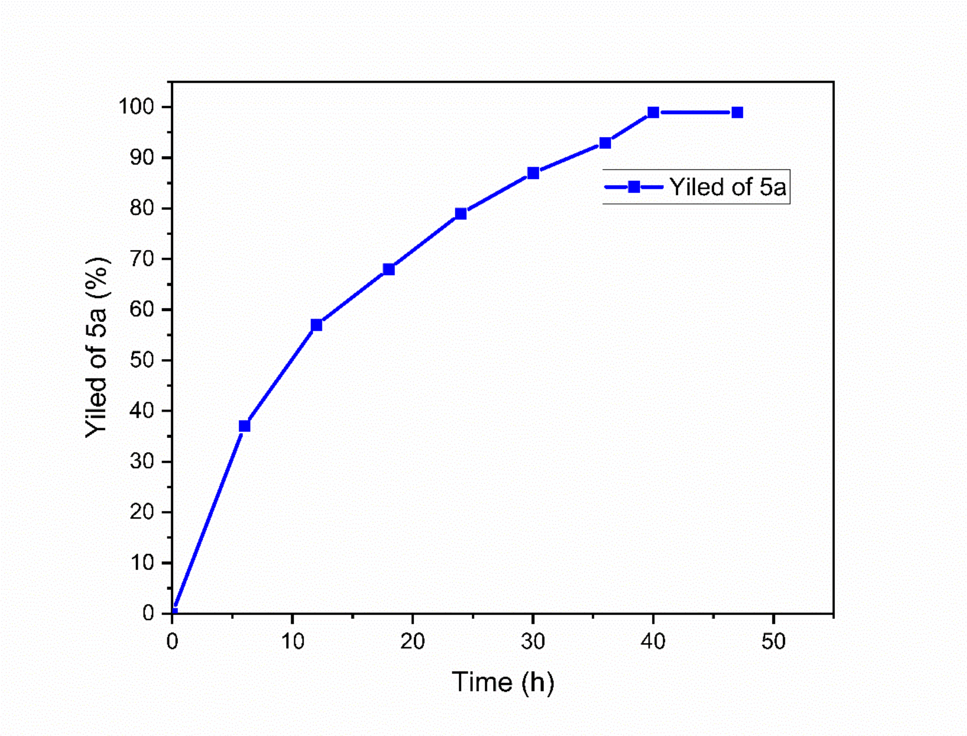

Supplementary Figure 40. Catalytic performances of 0.25 mol% $Cu_3NC^{(NHC)}$ in redox-A^3 coupling reaction of phenylacetylene at room temperature.

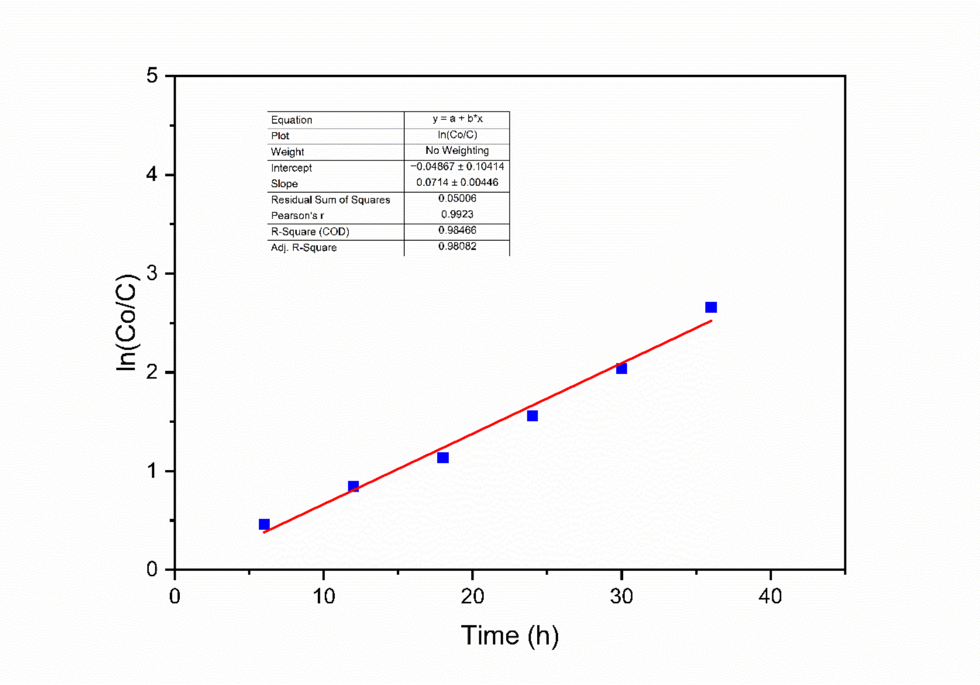

Supplementary Figure 41. Kinetic curve (ln(C_0/C) vs. t) of phenylacetylene redox-A^3 coupling reaction by using 0.25 mol% $Cu_3NC^{(NHC)}$ as catalyst. C_0 and C are the phenylacetylene concentrations before the reaction and at a reaction time of t, respectively.

Recyclability of the $Cu_3NC^{(NHC)}$ Catalyzed A^3 Coupling Reaction and Redox-A^3 Coupling Reaction

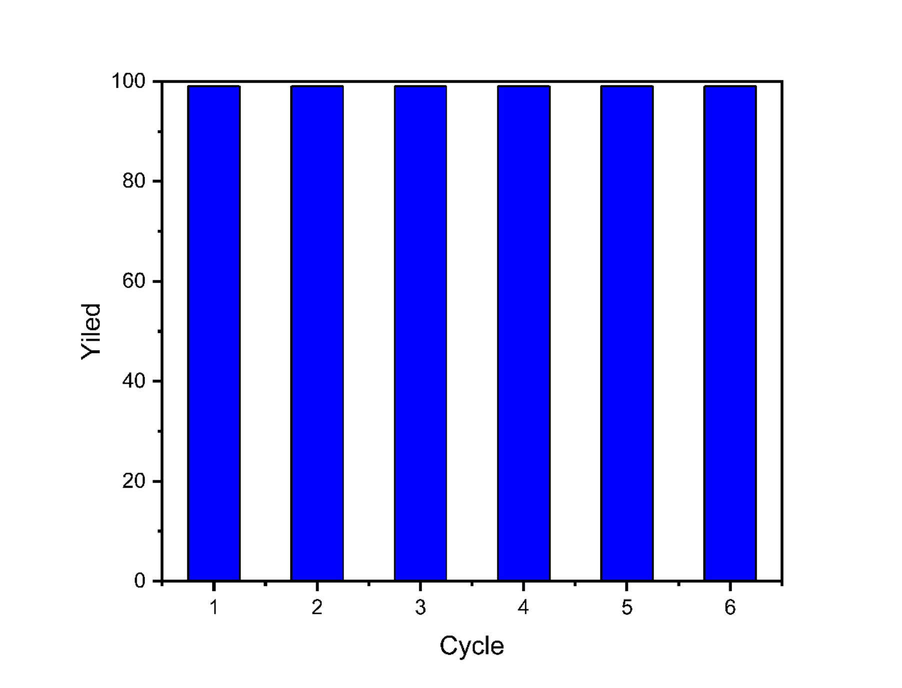

Supplementary Figure 42. Recyclability of the $Cu_3NC^{(NHC)}$ catalyzed A^3 coupling reaction in term of yield (12 h).

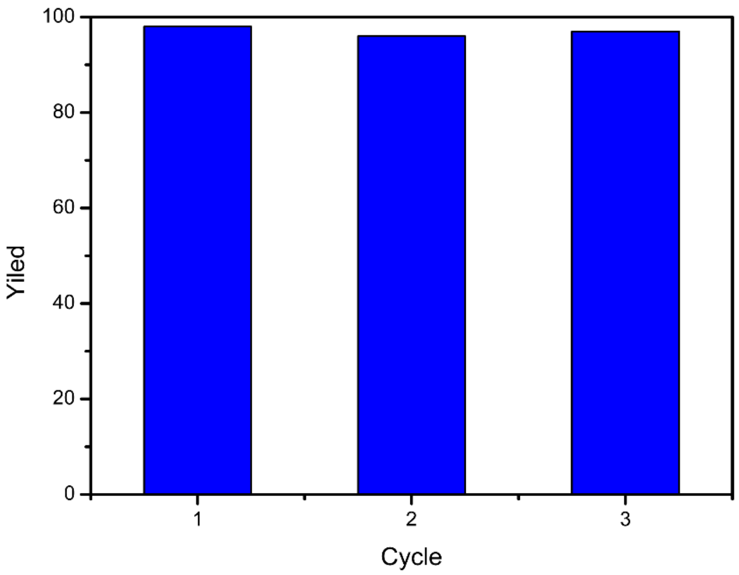

Supplementary Figure 43. Recyclability of the $Cu_3NC^{(NHC)}$ catalyzed Redox-A^3 coupling reaction in term of yield (40 h).

Tables of Crystal Data and Structure Refinements

Supplementary Table 3. Crystal data and structure refinements for $Cu_3NC^{(NHC)}$.

	$Cu_3NC^{(NHC)}$
**Empirical formula**	$C_{91}H_{87}Cu_3F_{18}N_{17}P_3$
**Formula weight**	2044.30
**Temperature/K**	200
**Crystal system**	triclinic
**Space group**	P-1
***a*/Å**	14.84700(10)
***b*/Å**	16.7001(2)
***c*/Å**	19.3691(2)
**α/°**	91.3110(10)
**β/°**	94.2830(10)
**γ/°**	96.5040(10)
**Volume/Å^3**	4755.88(8)
**Z**	2
**$\rho calc$g/cm^3**	1.428
**μ/mm^{-1}**	2.027
**F(000)**	2092.0
**Crystal size/mm^3**	0.12 × 0.11 × 0.1
**Radiation**	CuKα (λ = 1.54184 Å)
**2θrange for data collection/°**	5.328 to 148.822
**Index ranges**	-18 ≤ h ≤ 18, -17 ≤ k ≤ 20, -24 ≤ l ≤ 22
**Reflections collected**	58399
**Independent reflections**	18898 [Rint = 0.0365, Rsigma = 0.0364]
**Data/restraints/parameters**	18898/228/1250
**Goodness-of-fit on F^2**	1.068
**Final R indexes [I>=2σ (I)]**	R_1 = 0.0583, wR_2 = 0.1697
**CCDC number**	2268919

Supplementary Table 4. Crystal data and structure refinements for $Cu_3NC^{(Pz)}$.

	$Cu_3NC^{(Pz)}$
**Empirical formula**	$C_{47}H_{37}C_{l4}Cu_3N_6$
**Formula weight**	1018.24
**Temperature/K**	200.00(10)
**Crystal system**	monoclinic
**Space group**	$P2_1/n$
***a*/Å**	13.5166(2)
***b*/Å**	14.4830(2)
***c*/Å**	22.9824(4)
**α/°**	90
**β/°**	105.043(2)
**γ/°**	90
**Volume/$Å^3$**	4344.88(12)
**Z**	4
***ρcalc*g/cm^3**	1.557
**μ/mm^{-1}**	4.295
**F(000)**	2064.0
**Crystal size/mm^3**	0.1 × 0.08 × 0.06
**Radiation**	CuKα (λ = 1.54184 Å)
**2θrange for data collection/°**	6.908 to 148.792
**Index ranges**	$-13 \leq h \leq 16$, $-17 \leq k \leq 9$, $-28 \leq l \leq 28$
**Reflections collected**	23250
**Independent reflections**	8587 [Rint = 0.0420, Rsigma = 0.0489]
**Data/restraints/parameters**	8587/24/541
**Goodness-of-fit on F^2**	1.029
**Final R indexes [I>=2σ (I)]**	R_1 = 0.0684, wR_2 = 0.1974
**CCDC number**	2268920

Compound Characterization of A^3 Coupling Reaction

N, N-dibenzyl-1,3-diphenylprop-2-yn-1-amine (4a)

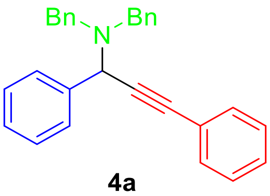

4a

Prepared according to general procedure (B), **4a** was purified by flash chromatography (PE-EA, v/v 10/1) as colorless oil with overall isolated yield: 96% (74.3 mg). **^{1}H NMR** (600 MHz, $CDCl_3$) δ 7.73 (d, J = 7.6 Hz, 2H), 7.63 (dd, J = 7.3, 2.0 Hz, 2H), 7.44 (d, J = 7.4 Hz, 4H), 7.36 (ddd, J = 22.3, 13.0, 6.4 Hz, 9H), 7.28 – 7.21 (m, 3H), 4.93 (s, 1H), 3.79 (d, J = 13.5 Hz, 2H), 3.54 (d, J = 13.5 Hz, 2H). **^{13}C NMR** (151 MHz, $CDCl_3$) δ 139.5, 139.1, 131.9, 128.9, 128.4, 128.3, 128.1, 127.5, 127.0, 123.2, 88.6, 84.6, 56.0, 54.6.

N, N-dibenzyl-1-(2-methoxyphenyl)-3-phenylprop-2-yn-1-amine (4b)

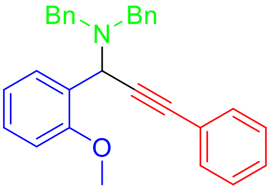

4b

Prepared according to general procedure (B), **4b** was purified by flash chromatography (PE-EA, v/v 10/1) as pale-yellow oil with overall isolated yield: 98% (81.7 mg). **^{1}H NMR** (600 MHz, $CDCl_3$) δ 7.67 (d, *J* = 8.4 Hz, 1H), 7.50 (d, *J* = 6.3 Hz, 2H), 7.28 (dd, *J* = 16.9, 7.4 Hz, 7H), 7.20 (t, *J* = 7.5 Hz, 4H), 7.18 – 7.11 (m, 3H), 6.84 (t, *J* = 7.4 Hz, 1H), 6.77 (d, *J* = 8.2 Hz, 1H), 5.19 (s, 1H), 3.76 (d, *J* = 13.6 Hz, 2H), 3.64 (s, 3H), 3.49 (d, *J* = 13.6 Hz, 2H). **^{13}C NMR** (151 MHz, $CDCl_3$) δ 157.6, 139.9, 131.9, 130.6, 129.1, 129.0, 128.3, 128.0, 127.8, 127.0, 126.7, 123.6, 119.7, 110.8, 87.5, 85.9, 55.0, 54.9, 51.4.

N, N-dibenzyl-1-(3-methoxyphenyl)-3-phenylprop-2-yn-1-amine (4c)

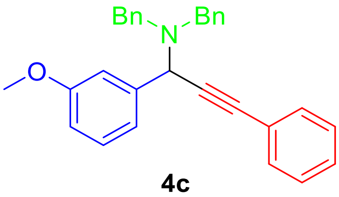

4c

Prepared according to general procedure (B), **4c** was purified by flash chromatography (PE-EA, v/v 10/1) as white solid with overall isolated yield: 95% (79.3 mg). **^{1}H NMR** (600 MHz, $CDCl_3$) δ 7.58 – 7.52 (m, 2H), 7.36 (d, *J* = 7.4 Hz, 4H), 7.33 – 7.29 (m, 3H), 7.28 – 7.22 (m, 6H), 7.21 – 7.14 (m, 3H), 6.73 (d, *J* = 8.0 Hz, 1H), 4.84 (s, 1H), 3.80 – 3.69 (m, 5H), 3.47 (d, *J* = 13.5 Hz, 2H). **^{13}C NMR** (151 MHz, $CDCl_3$) δ 159.5, 140.9, 139.5, 132.0, 129.1, 128.9, 128.4, 128.3, 128.2, 127.0, 123.3, 120.7, 114.2, 112.6, 88.6, 84.7, 56.1, 55.2, 54.7.

N, N-dibenzyl-1-(4-methoxyphenyl)-3-phenylprop-2-yn-1-amine (4d)

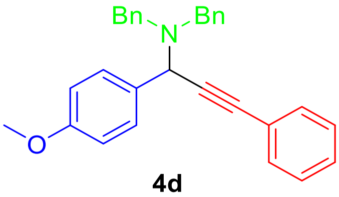

4d

Prepared according to general procedure (B), **4d** was purified by flash chromatography (PE-EA, v/v 10/1) as colorless oil with overall isolated yield: 96% (80.1 mg). **^{1}H NMR** (600 MHz, $CDCl_3$) δ 7.74 – 7.67 (m, 4H), 7.51 (d, *J* = 7.4 Hz, 4H), 7.45 (d, *J* = 6.9 Hz, 3H), 7.39 (t, *J* = 7.5 Hz, 4H), 7.30 (t, *J* = 7.3 Hz, 2H), 6.96 (d, *J* = 8.7 Hz, 2H), 4.96 (s, 1H), 3.87 (d, *J* = 15.1 Hz, 5H), 3.60 (d, *J* = 13.5 Hz, 2H). **^{13}C NMR** (151 MHz, $CDCl_3$) δ 158.9, 139.6, 131.9, 131.2, 129.3, 128.8, 128.4, 128.2, 128.2, 126.9, 123.2, 113.4, 88.4, 85.0, 55.4, 55.2, 54.4.

N, N-dibenzyl-1-(2-fluorophenyl)-3-phenylprop-2-yn-1-amine (4e)

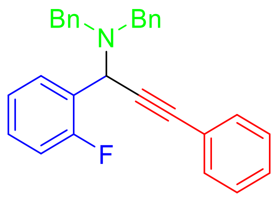

4e

Prepared according to general procedure (B), **4e** was purified by flash chromatography (PE-EA, v/v 10/1) as colorless oil with overall isolated yield: 93% (75.4 mg). **^{1}H NMR** (600 MHz, $CDCl_3$) δ 7.69 (t, *J* = 6.8 Hz, 1H), 7.56 – 7.49 (m, 2H), 7.35 – 7.27 (m, 7H), 7.22 (dd, *J* = 10.5, 4.5 Hz, 4H), 7.17 – 7.12 (m, 3H), 7.01 (t, *J* = 7.5 Hz, 1H), 6.95 (t, *J* = 9.3 Hz, 1H), 5.14 (s, 1H), 3.74 (d, *J* = 13.5 Hz, 2H), 3.48 (d, *J* = 13.5 Hz, 2H). **^{13}C NMR** (151 MHz, $CDCl_3$) δ 161.1 (d, *J* = 251.2 Hz), 139.2, 131.9, 131.0 (d, *J* = 3.4 Hz), 129.6 (d, *J* = 8.2 Hz), 129.0, 128.4, 128.3, 128.1, 127.0, 126.1 (d, *J* = 12.8 Hz), 123.4 (d, *J* = 3.6 Hz), 123.1, 115.6 (d, *J* = 21.4 Hz), 88.4, 84.0, 54.9, 51.5. **^{19}F NMR** (565 MHz, $CDCl_3$) δ -115.03.

N, N-dibenzyl-1-(3-fluorophenyl)-3-phenylprop-2-yn-1-amine (4f)

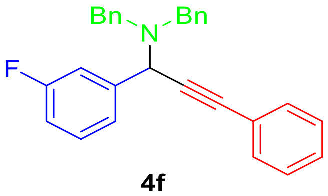

4f

Prepared according to general procedure (B), **4f** was purified by flash chromatography (PE-EA, v/v 10/1) as white solid with overall isolated yield: 95% (77.0 mg). **^{1}H NMR** (600 MHz, $CDCl_3$) δ 7.65 – 7.60 (m, 2H), 7.52 (d, *J* = 7.7 Hz, 1H), 7.47 – 7.37 (m, 8H), 7.34 – 7.28 (m, 5H), 7.23 (dd, *J* = 14.2, 6.9 Hz, 2H), 6.95 (t, *J* = 8.2 Hz, 1H), 4.90 (s, 1H), 3.78 (d, *J* = 13.4 Hz, 2H), 3.53 (d, *J* = 13.5 Hz, 2H). **^{13}C NMR** (151 MHz, $CDCl_3$) δ 162.8 (d, *J* = 245.2 Hz), 142.1 (d, *J* = 7.0 Hz), 139.2, 132.0, 129.5 (d, *J* = 8.1 Hz), 128.9, 128.4, 128.4, 128.4, 127.1, 123.9 (d, *J* = 2.6 Hz), 123.0, 115.2 (d, *J* = 22.5 Hz), 114.4 (d, *J* = 21.3 Hz), 88.9, 84.0, 55.8, 54.7. **^{19}F NMR** (565 MHz, $CDCl_3$) δ -113.43.

N, N-dibenzyl-1-(4-fluorophenyl)-3-phenylprop-2-yn-1-amine (4g)

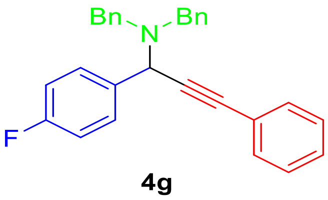

4g

Prepared according to general procedure (B), **4g** was purified by flash chromatography (PE-EA, v/v 10/1) as colorless oil with overall isolated yield: 94% (76.1 mg). **^{1}H NMR** (600 MHz, $CDCl_3$) δ 7.73 (dd, *J* = 8.3, 5.6 Hz, 2H), 7.67 (dd, *J* = 7.4, 1.7 Hz, 2H), 7.48 – 7.42 (m, 7H), 7.37 (t, *J* = 7.6 Hz, 4H), 7.29 (t, *J* = 7.3 Hz, 2H), 7.08 (t, *J* = 8.6 Hz, 2H), 4.94 (s, 1H), 3.83 (d, *J* = 13.5 Hz, 2H), 3.58 (d, *J* = 13.5 Hz, 2H). **^{13}C NMR** (151

MHz, $CDCl_3$) δ 162.2 (d, *J* = 245.7 Hz), 139.4, 135.0 (d, *J* = 3.0 Hz), 132.0, 129.9 (d, *J* = 8.1 Hz), 128.9, 128.4, 128.4, 128.3, 127.1, 123.1, 114.9 (d, *J* = 21.4 Hz), 88.9, 84.4, 55.5, 54.6. ^{19}**F NMR** (565 MHz, $CDCl_3$) δ -115.27.

N, N-dibenzyl-1-(2-bromophenyl)-3-phenylprop-2-yn-1-amine (4h)

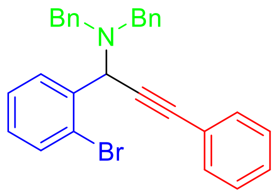

4h

Prepared according to general procedure (B), **4h** was purified by flash chromatography (PE-EA, v/v 5/1) as yellow oil with overall isolated yield: 91% (84.6 mg). ^{1}**H NMR** (600 MHz, $CDCl_3$) δ 7.86 (d, *J* = 7.7 Hz, 1H), 7.64 – 7.57 (m, 2H), 7.52 (d, *J* = 7.8 Hz, 1H), 7.41 – 7.34 (m, 7H), 7.26 (t, *J* = 7.5 Hz, 4H), 7.24 – 7.18 (m, 3H), 7.08 (dd, *J* = 10.9, 4.3 Hz, 1H), 5.18 (s, 1H), 3.81 (d, *J* = 13.2 Hz, 2H), 3.55 (d, *J* = 13.3 Hz, 2H). ^{13}**C NMR** (151 MHz, $CDCl_3$) δ 138.8, 137.8, 133.5, 131.9, 131.8, 129.6, 129.2, 128.4, 128.3, 127.9, 127.0, 126.6, 124.9, 123.1, 89.2, 84.8, 57.1, 55.1.

N, N-dibenzyl-1-(3-bromophenyl)-3-phenylprop-2-yn-1-amine (4i)

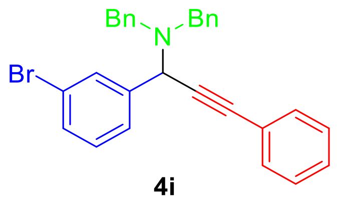

4i

Prepared according to general procedure (B), **4i** was purified by flash chromatography (PE-EA, v/v 10/1) as white sollid with overall isolated yield: 97% (90.2 mg). ^{1}**H NMR** (600 MHz, $CDCl_3$) δ 7.87 (s, 1H), 7.68 – 7.59 (m, 3H), 7.39 (dd, *J* = 17.4, 6.8 Hz, 8H), 7.32 (t, *J* = 7.3 Hz, 4H), 7.26 – 7.18 (m, 3H), 4.89 (s, 1H), 3.77 (d, *J* = 13.4 Hz, 2H), 3.52 (d, *J* = 13.4 Hz, 2H). ^{13}**C NMR** (151 MHz, $CDCl_3$) δ 141.7, 139.2, 132.0, 131.3, 130.6, 129.7, 128.9, 128.4, 128.4, 127.2, 127.0, 123.0, 122.3, 89.2, 83.8, 55.7, 54.7.

N, N-dibenzyl-1-(4-bromophenyl)-3-phenylprop-2-yn-1-amine (4j)

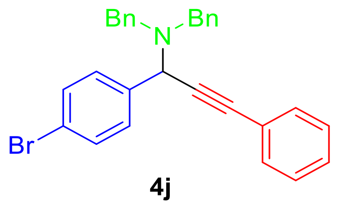

4j

Prepared according to general procedure (A), **4j** was purified by flash chromatography (PE-EA, v/v 5/1) as colorless oil with overall isolated yield: 93% (86.5 mg). ^{1}**H NMR** (600 MHz, $CDCl_3$) δ 7.66 (dt, *J* = 14.1, 5.7 Hz, 4H), 7.54 – 7.50 (m, 2H), 7.45 (dd, *J* = 13.4, 7.0 Hz, 7H), 7.38 (t, *J* = 7.3 Hz, 4H), 7.30 (t, *J* = 6.2 Hz, 2H), 4.92 (s, 1H), 3.83 (d, *J* = 13.4 Hz, 2H), 3.58 (d, *J* = 13.5 Hz, 2H). ^{13}**C NMR** (151 MHz, $CDCl_3$) δ 139.2,

138.4, 132.0, 131.2, 130.0, 128.9, 128.4, 128.4, 128.3, 127.1, 123.0, 121.4, 89.0, 84.0, 55.6, 54.7.

4-(1-(dibenzylamino)-3-phenylprop-2-yn-1-yl) benzonitrile (4k)

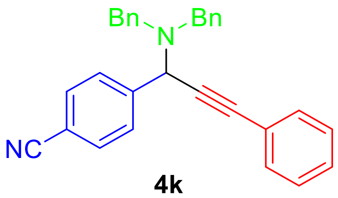

4k

Prepared according to general procedure (B), **4k** was purified by flash chromatography (PE-EA, v/v 10/1) as pale-yellow solid with overall isolated yield: 98% (80.8 mg). **^{1}H NMR** (600 MHz, $CDCl_3$) δ 7.89 (d, *J* = 8.0 Hz, 2H), 7.70 (dd, *J* = 9.1, 4.7 Hz, 4H), 7.46 (d, *J* = 7.2 Hz, 7H), 7.39 (t, *J* = 7.5 Hz, 4H), 7.31 (t, *J* = 7.3 Hz, 2H), 4.99 (s, 1H), 3.81 (d, *J* = 13.5 Hz, 2H), 3.61 (d, *J* = 13.5 Hz, 2H). **^{13}C NMR** (151 MHz, $CDCl_3$) δ 144.9, 138.8, 131.9, 131.9, 128.9, 128.8, 128.6, 128.4, 128.4, 127.3, 122.6, 118.8, 111.3, 89.6, 83.0, 56.0, 54.8.

N, N-dibenzyl-1-(3,5-dimethylphenyl)-3-phenylprop-2-yn-1-amine (4l)

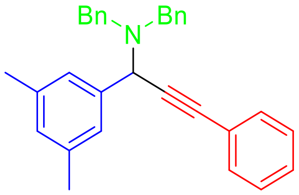

4l

Prepared according to general procedure (B), **4l** was purified by flash chromatography (PE-EA, v/v 10/1) as colorless oil with overall isolated yield: 94% (78.0 mg).**^{1}H NMR** (600 MHz, $CDCl_3$) δ 7.61 (s, 2H), 7.46 – 7.28 (m, 13H), 7.21 (s, 2H), 6.88 (s, 1H), 4.87 (s, 1H), 3.79 (d, *J* = 12.7 Hz, 2H), 3.53 (d, *J* = 12.6 Hz, 2H), 2.32 (s, 6H).**^{13}C NMR** (151 MHz, $CDCl_3$) δ 139.7, 139.0, 137.5, 132.0, 129.1, 128.9, 128.4, 128.3, 128.1, 126.9, 126.1, 123.5, 88.4, 85.2, 56.0, 54.7, 21.4.

N, N-dibenzyl-3-phenylprop-2-yn-1-amine (4m)

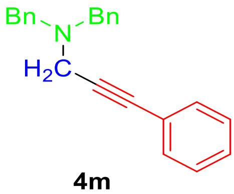

4m

Prepared according to general procedure (B), **4m** was purified by flash chromatography (PE-EA, v/v 10/1) as colorless oil with overall isolated yield: 94% (58.5 mg). **^{1}H NMR** (600 MHz, $CDCl_3$) δ 7.51 (dt, *J* = 4.5, 2.5 Hz, 2H), 7.44 (d, *J* = 7.3 Hz, 4H), 7.37 – 7.30 (m, 7H), 7.26 (t, *J* = 7.3 Hz, 2H), 3.77 (s, 4H), 3.48 (s, 2H). **^{13}C NMR** (151 MHz, $CDCl_3$) δ 138.9, 131.8, 129.1, 128.3, 128.0, 127.1, 123.4, 85.9, 84.4, 57.7, 42.0.

N, N-dibenzyl-1-cyclohexyl-3-phenylprop-2-yn-1-amine (4n)

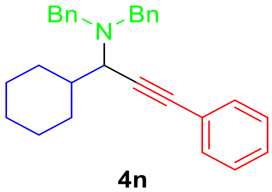

4n

Prepared according to general procedure (B), **4n** was purified by flash chromatography (PE-EA, v/v 10/1) as colorless oil with overall isolated yield: 92% (72.3 mg). **^{1}H NMR** (600 MHz, $CDCl_3$) δ 7.45 – 7.42 (m, 2H), 7.35 (d, *J* = 7.5 Hz, 4H), 7.28 – 7.21 (m, 7H), 7.15 (t, *J* = 7.3 Hz, 2H), 3.81 (d, *J* = 13.7 Hz, 2H), 3.39 (d, *J* = 1.2 Hz, 2H), 3.17 (d, *J* = 10.4 Hz, 1H), 2.25 (d, *J* = 13.1 Hz, 1H), 1.99 (d, *J* = 12.9 Hz, 1H), 1.62 (d, *J* = 11.8 Hz, 3H), 1.54 (d, *J* = 11.7 Hz, 1H), 1.08 (ddd, *J* = 35.5, 27.3, 13.1 Hz, 3H), 0.81 (q, *J* = 12.2 Hz, 1H), 0.70 (q, *J* = 12.1 Hz, 1H). **^{13}C NMR** (151 MHz, $CDCl_3$) δ 139.8, 131.8, 128.9, 128.3, 128.2, 127.8, 126.8, 123.7, 87.2, 86.3, 58.4, 55.1, 39.9, 31.4, 30.4, 26.6, 26.2, 26.0.

N, N-dibenzyl-4-methyl-1-phenylpent-1-yn-3-amine (4o)

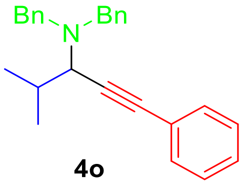

4o

Prepared according to general procedure (B), **4o** was purified by flash chromatography (PE-EA, v/v 10/1) as colorless oil with overall isolated yield: 93% (65.7 mg). **^{1}H NMR** (600 MHz, $CDCl_3$) δ 7.43 (d, *J* = 6.6 Hz, 2H), 7.35 (d, *J* = 7.5 Hz, 4H), 7.28 – 7.20 (m, 7H), 7.14 (dd, *J* = 13.2, 5.9 Hz, 2H), 3.80 (d, *J* = 13.7 Hz, 2H), 3.39 (d, *J* = 13.8 Hz, 2H), 3.04 (d, *J* = 10.4 Hz, 1H), 1.92 (qd, *J* = 13.0, 6.5 Hz, 1H), 0.96 (t, *J* = 6.5 Hz, 6H). **^{13}C NMR** (151 MHz, $CDCl_3$) δ 139. 8, 131.8, 128.9, 128.3, 128.2, 127.8, 126.8, 123.7, 87.4, 86.0, 59.7, 55.1, 30.9, 21.0, 20.0.

N, N-dibenzyl-3-phenyl-1-(pyridin-4-yl) prop-2-yn-1-amine (4p)

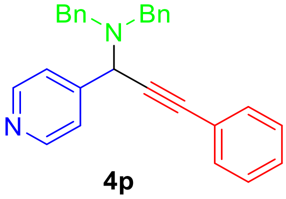

4p

Prepared according to general procedure (B), **4p** was purified by flash chromatography (PE-EA, v/v 5/1) as pale-yellow solid with overall isolated yield: 96% (74.5 mg). **^{1}H NMR** (600 MHz, $CDCl_3$) δ 8.60 (d, *J* = 6.0 Hz, 2H), 7.68 – 7.61 (m, 4H), 7.41 (dd, *J* = 8.3, 4.9 Hz, 7H), 7.33 (dt, *J* = 7.9, 4.9 Hz, 4H), 7.25 (dd, *J* = 9.5, 5.1 Hz, 2H), 4.89 (s, 1H), 3.76 (d, *J* = 13.5 Hz, 2H), 3.55 (d, *J* = 13.5 Hz, 2H). **^{13}C NMR** (151 MHz, $CDCl_3$) δ 149.8, 148.6, 138.9, 132.0, 128.9, 128.5, 128.4, 128.1, 127.3, 126.9, 123.2, 89.4, 82.9, 55.6, 54.9.

N, N-dibenzyl-3-phenyl-1-(pyridin-3-yl) prop-2-yn-1-amine (4q)

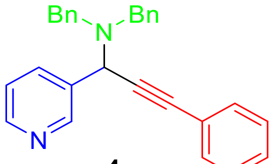

4q

Prepared according to general procedure (B), **4q** was purified by flash chromatography (PE-EA, v/v 5/1) as white solid with overall isolated yield: 95% (73.7 mg). **^{1}H NMR** (600 MHz, $CDCl_3$) δ 8.96 (s, 1H), 8.51 (d, *J* = 4.5 Hz, 1H), 7.98 (d, *J* = 7.9 Hz, 1H), 7.65 – 7.59 (m, 2H), 7.44 – 7.36 (m, 7H), 7.32 (t, *J* = 7.6 Hz, 4H), 7.28 – 7.21 (m, 3H), 4.95 (s, 1H), 3.78 (d, *J* = 13.5 Hz, 2H), 3.55 (d, *J* = 13.5 Hz, 2H). **^{13}C NMR** (151 MHz, $CDCl_3$) δ 150.1, 148.8, 139.0, 135.8, 134.8, 132.0, 128.9, 128.5, 128.4, 128.4, 127.2, 122.9, 122.8, 89.5, 83.1, 54.7, 54.1.

N, N-dibenzyl-3-phenyl-1-(thiophen-2-yl) prop-2-yn-1-amine (4r)

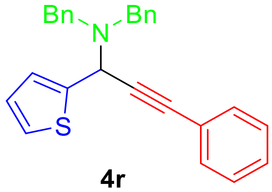

4r

Prepared according to general procedure (B), **4r** was purified by flash chromatography (PE-EA, v/v 10/1) as pale-yellow oil with overall isolated yield: 90% (70.7 mg). **^{1}H NMR** (600 MHz, $CDCl_3$) δ 7.62 – 7.58 (m, 2H), 7.50 (d, *J* = 7.6 Hz, 4H), 7.38 (dd, *J* = 4.9, 1.4 Hz, 2H), 7.33 (t, *J* = 7.5 Hz, 4H), 7.30 – 7.28 (m, 1H), 7.27 – 7.21 (m, 4H), 6.96 – 6.92 (m, 1H), 5.05 (s, 1H), 3.91 (d, *J* = 13.7 Hz, 2H), 3.55 (d, *J* = 13.7 Hz, 2H). **^{13}C NMR** (151 MHz, $CDCl_3$) δ 144.7, 139.3, 132.0, 128.7, 128.4, 128.3, 127.1, 126.2, 126.0, 125.5, 123.0, 87.2, 84.3, 54.6, 52.7.

N, N-dibenzyl-3-phenyl-1-(thiophen-3-yl) prop-2-yn-1-amine (4s)

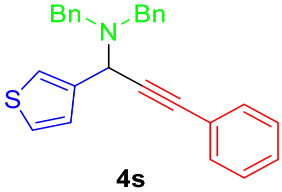

4s

Prepared according to general procedure (B), **4s** was purified by flash chromatography (PE-EA, v/v 5/1) as colorless oil with overall isolated yield: 77% (60.5 mg). **^{1}H NMR** (600 MHz, $CDCl_3$) δ 7.64 (d, *J* = 1.4 Hz, 2H), 7.56 – 7.53 (m, 1H), 7.47 (s, 4H), 7.42 (s, 3H), 7.39 – 7.31 (m, 6H), 7.30 – 7.25 (m, 2H), 4.94 (s, 1H), 3.87 (d, *J* = 13.0 Hz, 2H), 3.58 (d, *J* = 13.1 Hz, 2H). **^{13}C NMR** (151 MHz, $CDCl_3$) δ 141.1, 139.5, 132.0, 128.8, 128.4, 128.3, 128.3, 127.5, 127.0, 125.7, 123.2, 87.0, 85.3, 54.7, 52.6.

N, N-dibenzyl-1-(5-bromothiophen-2-yl)-3-phenylprop-2-yn-1-amine (4t)

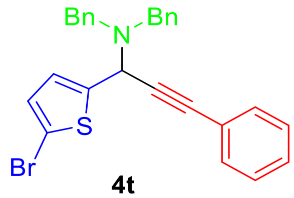

4t

Prepared according to general procedure (B), **4t** was purified by flash chromatography (PE-EA, v/v 10/1) as colorless oil with overall isolated yield: 88% (82.9 mg). ^{1}H NMR (400 MHz, $CDCl_3$) δ 7.63 (dd, *J* = 6.5, 3.0 Hz, 2H), 7.53 (d, *J* = 7.4 Hz, 4H), 7.46 – 7.36 (m, 7H), 7.29 (t, *J* = 7.3 Hz, 2H), 7.09 (d, *J* = 2.5 Hz, 1H), 6.93 (d, *J* = 3.7 Hz, 1H), 5.00 (s, 1H), 3.96 (d, *J* = 13.7 Hz, 2H), 3.57 (d, *J* = 13.7 Hz, 2H). **^{13}C NMR** (101 MHz, $CDCl_3$) δ 146.2, 138.9, 132.1, 132.0, 129.1, 128.7, 128.6, 128.4, 127.2, 126.1, 122.6, 112.2, 87.7, 83.2, 54.6, 52.9.

N, N-dibenzyl-1-(furan-2-yl)-3-phenylprop-2-yn-1-amine (4u)

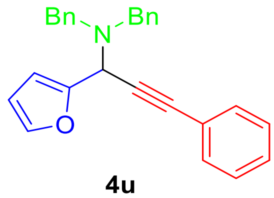

4u

Prepared according to general procedure (B), **4u** was purified by flash chromatography (PE-EA, v/v 10/1) as colorless oil with overall isolated yield: 65% (49.0 mg). **^{1}H NMR** (600 MHz, $CDCl_3$) δ 7.58 – 7.53 (m, 2H), 7.48 – 7.41 (m, 5H), 7.38 – 7.34 (m, 3H), 7.31 (t, *J* = 7.5 Hz, 4H), 7.23 (dd, *J* = 8.8, 5.8 Hz, 2H), 6.51 (d, *J* = 2.4 Hz, 1H), 6.32 (dd, *J* = 2.9, 1.8 Hz, 1H), 4.96 (s, 1H), 3.82 (d, *J* = 13.8 Hz, 2H), 3.63 (d, *J* = 13.8 Hz, 2H). **^{13}C NMR** (151 MHz, $CDCl_3$) δ 152.3, 142.6, 139.4, 132.0, 128.8, 128.3, 128.3, 127.0, 123.0, 110.0, 109.1, 86.2, 83.7, 54.7, 51.1.

N, N-dibenzyl-3-phenyl-1-(pyren-4-yl) prop-2-yn-1-amine (4v)

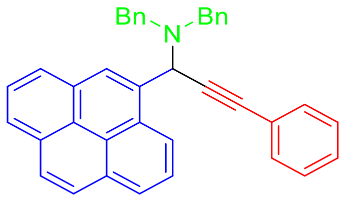

4v

Prepared according to general procedure (B), **4v** was purified by flash chromatography (PE-EA, v/v 5/1) as white solid with overall isolated yield: 95% (97.1 mg). **^{1}H NMR** (600 MHz, $CDCl_3$) δ 8.73 (d, *J* = 6.2 Hz, 1H), 8.22 (dd, *J* = 24.3, 5.6 Hz, 3H), 8.11 – 7.99 (m, 5H), 7.87 (s, 2H), 7.60 – 7.31 (m, 13H), 5.96 (s, 1H), 3.98 (d, *J* = 11.7 Hz, 2H), 3.79 (d, *J* = 11.5 Hz, 2H). **^{13}C NMR** (151 MHz, $CDCl_3$) δ 139.1, 132.0, 131.6, 131.2, 130.7, 129.7, 129.2, 128.5, 128.3, 128.2, 127.6, 127.4, 127.3, 127.2, 126.6, 125.8, 125.1, 125.0, 124.6, 124.4, 123.9, 123.4, 89.6, 85.3, 55.0, 54.7.

N, N-dibenzyl-3-phenyl-1-(4-(pyridin-4-yl) phenyl) prop-2-yn-1-amine (4w)

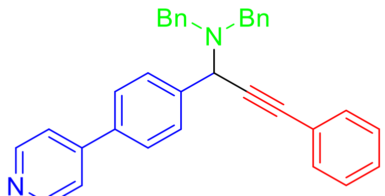

4w

Prepared according to general procedure (B), **4w** was purified by flash chromatography (PE-EA, v/v 10/1) as white solid with overall isolated yield: 74% (68.7 mg). **^{1}H NMR** (600 MHz, $CDCl_3$) δ 8.68 (d, *J* = 4.8 Hz, 2H), 7.88 (d, *J* = 8.1 Hz, 2H), 7.71 – 7.65 (m, 4H), 7.52 (d, *J* = 5.7 Hz, 2H), 7.49 (d, *J* = 7.6 Hz, 4H), 7.44 (t, *J* = 6.7 Hz, 3H), 7.37 (t, *J* = 7.4 Hz, 4H), 7.30 – 7.27 (m, 2H), 5.02 (s, 1H), 3.86 (d, *J* = 13.5 Hz, 2H), 3.61 (d, *J* = 13.5 Hz, 2H). **^{13}C NMR** (151 MHz, $CDCl_3$) δ 150.2, 148.0, 140.5, 139.3, 137.3, 132.0, 129.0, 128.9, 128.4, 128.4, 128.3, 127.1, 126.7, 123.1, 121.5, 89.0, 84.2, 55.9, 54.7.

1-(anthracen-9-yl)-N, N-dibenzyl-3-phenylprop-2-yn-1-amine (4x)

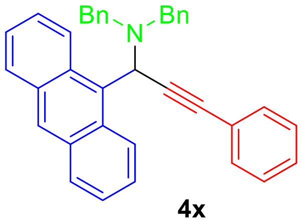

4x

Prepared according to general procedure (B), **4x** was purified by flash chromatography (PE-EA, v/v 5/1) as yellow solid with overall isolated yield: 52% (50.7 mg). **^{1}H NMR** (600 MHz, $CDCl_3$) δ 8.79 (s, 1H), 8.37 (s, 1H), 7.94 (d, *J* = 8.2 Hz, 2H), 7.43 (ddd, *J* = 17.6, 15.7, 6.9 Hz, 6H), 7.25 (tt, *J* = 21.4, 7.4 Hz, 14H), 6.15 (s, 1H), 3.97 (d, *J* = 13.5 Hz, 2H), 3.85 (d, *J* = 13.5 Hz, 2H). **^{13}C NMR** (151 MHz, $CDCl_3$) δ 138.2, 131.6, 130.9, 129.7, 129.6, 129.0, 128.8, 128.3, 128.2, 128.1, 127.0, 125.6, 125.4, 124.7, 123.4, 89.1, 88.6, 54.0, 52.2.

1,1'-([1,1'-biphenyl]-4,4'-diyl) bis (N, N-dibenzyl-3-phenylprop-2-yn-1-amine) (4y)

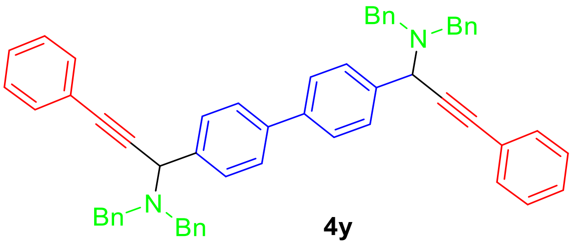

4y

Prepared according to general procedure (C), **4y** was purified by flash chromatography (PE-EA, v/v 10/1) as white solid with overall isolated yield: 86% (66.4 mg). **^{1}H NMR** (600 MHz, $CDCl_3$) δ 7.78 (d, *J* = 7.7 Hz, 4H), 7.64 (d, *J* = 6.5 Hz, 4H), 7.58 (d, *J* = 7.2 Hz, 4H), 7.45 (d, *J* = 7.1 Hz, 8H), 7.39 (d, *J* = 7.1 Hz, 6H), 7.32 (t, *J* = 7.2 Hz, 8H), 7.22 (dd, *J* = 12.5, 5.1 Hz, 4H), 4.97 (s, 2H), 3.83 (d, *J* = 13.5 Hz, 4H), 3.56 (d, *J* = 13.5

Hz, 4H). **^{13}C NMR** (151 MHz, $CDCl_3$) δ 140.1, 139.5, 138.3, 132.0, 128.9, 128.7, 128.4, 128.3, 128.3, 127.0, 126.8, 123.3, 88.7, 84.7, 55.9, 54.7.

N, N-dibenzyl-1-phenyl-3-(m-tolyl) prop-2-yn-1-amine (4z)

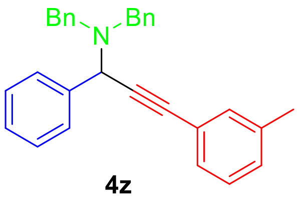

4z

Prepared according to general procedure (B), **4z** was purified by flash chromatography (PE-EA, v/v 10/1) as colorless oil with overall isolated yield: 98% (78.6 mg). **^{1}H NMR** (600 MHz, $CDCl_3$) δ 7.64 (d, *J* = 7.7 Hz, 2H), 7.37 – 7.32 (m, 6H), 7.26 (t, *J* = 7.6 Hz, 2H), 7.22 (t, *J* = 7.5 Hz, 4H), 7.19 – 7.11 (m, 4H), 7.08 (d, *J* = 7.3 Hz, 1H), 4.84 (s, 1H), 3.70 (d, *J* = 13.5 Hz, 2H), 3.46 (d, *J* = 13.5 Hz, 2H), 2.30 (s, 3H). **^{13}C NMR** (151 MHz, $CDCl_3$) δ 139.6, 139.3, 138.1, 132.5, 129.1, 129.1, 128.9, 128.3, 128.3, 128.1, 127.4, 127.0, 123.1, 88.8, 84.3, 56.1, 54.6, 21.3.

N, N-dibenzyl-1-phenyl-3-(p-tolyl) prop-2-yn-1-amine (4aa)

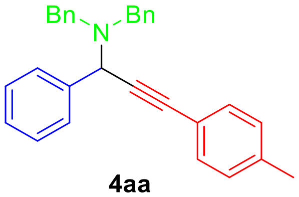

4aa

Prepared according to general procedure (B), **4aa** was purified by flash chromatography (PE-EA, v/v 10/1) as pale-yellow oil with overall isolated yield: 98% (78.5 mg). **^{1}H NMR** (600 MHz, $CDCl_3$) δ 7.65 (s, 2H), 7.46 – 7.41 (m, 2H), 7.35 (s, 4H), 7.29 – 7.20 (m, 6H), 7.13 (ddd, *J* = 21.0, 8.4, 5.1 Hz, 5H), 4.85 (s, 1H), 3.71 (d, *J* = 13.5 Hz, 2H), 3.46 (d, *J* = 13.5 Hz, 2H), 2.30 (s, 3H). **^{13}C NMR** (151 MHz, $CDCl_3$) δ 139.6, 139.3, 138.3, 131.8, 129.1, 128.9, 128.3, 128.3, 128.1, 127.4, 127.0, 120.2, 88.7, 83.9, 56.1, 54.6, 21.5.

N, N-dibenzyl-3-(2-methoxyphenyl)-1-phenylprop-2-yn-1-amine (4ab)

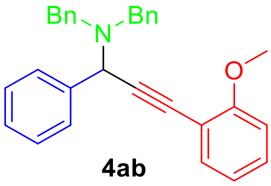

4ab

Prepared according to general procedure (B), **4ab** was purified by flash chromatography (PE-EA, v/v 10/1) as white solid with overall isolated yield: 94% (78.4 mg). **^{1}H NMR** (600 MHz, $CDCl_3$) δ 7.70 (d, *J* = 7.7 Hz, 2H), 7.50 (dd, *J* = 7.4, 1.2 Hz, 1H), 7.37 (d, *J* = 7.5 Hz, 4H), 7.30 – 7.21 (m, 7H), 7.16 (dt, *J* = 14.4, 7.3 Hz, 3H), 6.93 – 6.85 (m, 2H), 4.88 (s, 1H), 3.89 (s, 3H), 3.71 (d, *J* = 13.5 Hz, 2H), 3.52 (d, *J* = 13.5

Hz, 2H). **^{13}C NMR** (151 MHz, $CDCl_3$) δ 160.5, 139.8, 139.4, 133.5, 129.5, 129.0, 128.4, 128.2, 128.0, 127.3, 126.9, 120.4, 112.7, 110.8, 89.0, 84.8, 56.4, 55.8, 54.6.

N, N-dibenzyl-3-(4-methoxyphenyl)-1-phenylprop-2-yn-1-amine (4ac)

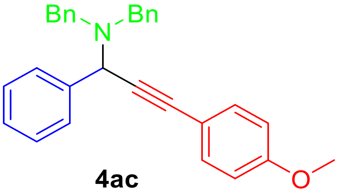

4ac

Prepared according to general procedure (B), **4ac** was purified by flash chromatography (PE-EA, v/v 10/1) as white solid with overall isolated yield: 99% (82.6 mg). **^{1}H NMR** (600 MHz, $CDCl_3$) δ 7.72 (d, *J* = 7.7 Hz, 2H), 7.56 (d, *J* = 8.7 Hz, 2H), 7.43 (d, *J* = 7.5 Hz, 4H), 7.33 (dt, *J* = 21.1, 7.6 Hz, 6H), 7.23 (dt, *J* = 14.9, 7.3 Hz, 3H), 6.92 (d, *J* = 8.7 Hz, 2H), 4.91 (s, 1H), 3.84 (s, 3H), 3.78 (d, *J* = 13.6 Hz, 2H), 3.53 (d, *J* = 13.5 Hz, 2H). **^{13}C NMR** (151 MHz, $CDCl_3$) δ 159.6, 139.6, 139.4, 133.3, 128.9, 128.3, 128.3, 128.1, 127.4, 127.0, 115.4, 114.0, 88.5, 83.1, 56.1, 55.4, 54.6.

N, N-dibenzyl-3-(2-fluorophenyl)-1-phenylprop-2-yn-1-amine (4ad)

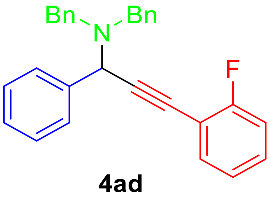

4ad

Prepared according to general procedure (B), **4ad** was purified by flash chromatography (PE-EA, v/v 10/1) as colorless oil with overall isolated yield: 95% (77.0 mg). **^{1}H NMR** (600 MHz, $CDCl_3$) δ 7.77 (d, *J* = 7.6 Hz, 2H), 7.63 (td, *J* = 7.6, 1.8 Hz, 1H), 7.47 (d, *J* = 7.5 Hz, 4H), 7.41 – 7.33 (m, 7H), 7.28 (dt, *J* = 20.5, 7.3 Hz, 3H), 7.19 (ddd, *J* = 8.4, 3.7, 2.8 Hz, 2H), 5.00 (s, 1H), 3.84 (d, *J* = 13.5 Hz, 2H), 3.59 (d, *J* = 13.5 Hz, 2H). **^{13}C NMR** (151 MHz, $CDCl_3$) δ 163.1 (d, *J* = 251.2 Hz), 139.5, 138.9, 133.7 (d, *J* = 0.9 Hz), 129.9 (d, *J* = 7.9 Hz), 128.9, 128.3, 128.3, 128.1, 127.5, 127.0, 124.0 (d, *J* = 3.7 Hz), 115.6 (d, *J* = 20.9 Hz), 111.9 (d, *J* = 15.9 Hz), 90.3 (d, *J* = 3.3 Hz), 81.9, 56.3, 54.6. **^{19}F NMR** (565 MHz, $CDCl_3$) δ -109.42.

N, N-dibenzyl-3-(2-bromophenyl)-1-phenylprop-2-yn-1-amine (4ae)

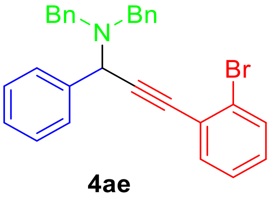

4ae

Prepared according to general procedure (B), **4ae** was purified by flash chromatography (PE-EA, v/v 10/1) as yellow oil with overall isolated yield: 96% (89.2 mg). **^{1}H NMR** (600 MHz, $CDCl_3$) δ 7.76 (d, *J* = 7.7 Hz, 2H), 7.65 (t, *J* = 7.4 Hz, 2H), 7.44 (d, *J* = 7.5 Hz, 4H), 7.35 (t, *J* = 7.6 Hz, 2H), 7.31 (t, *J* = 7.5 Hz, 5H), 7.28 – 7.18 (m, 4H), 4.98 (s,

1H), 3.81 (d, *J* = 13.5 Hz, 2H), 3.64 (d, *J* = 13.5 Hz, 2H). ^{13}**C NMR** (151 MHz, $CDCl_3$) δ 139.5, 138.9, 133.8, 132.5, 129.4, 128.9, 128.4, 128.3, 128.1, 127.5, 127.1, 127.0, 125.6, 125.5, 90.0, 87.0, 56.2, 54.6.

N, N-dibenzyl-3-(3-bromophenyl)-1-phenylprop-2-yn-1-amine (4af)

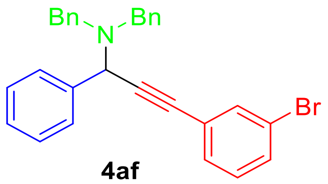

4af

Prepared according to general procedure (B), **4af** was purified by flash chromatography (PE-EA, v/v 10/1) as colorless oil with overall isolated yield: 95% (88.3 mg). ^{1}**H NMR** (600 MHz, $CDCl_3$) δ 7.81 (s, 1H), 7.75 (d, *J* = 6.3 Hz, 2H), 7.57 (dd, *J* = 23.4, 7.7 Hz, 2H), 7.48 (d, *J* = 6.3 Hz, 4H), 7.39 (dt, *J* = 23.1, 6.5 Hz, 6H), 7.34 – 7.27 (m, 4H), 4.99 (s, 1H), 3.85 (d, *J* = 13.4 Hz, 2H), 3.56 (d, *J* = 13.5 Hz, 2H). ^{13}**C NMR** (151 MHz, $CDCl_3$) δ 139.4, 138.8, 134.7, 131.4, 130.5, 129.8, 128.9, 128.3, 128.2, 128.2, 127.6, 127.1, 125.2, 122.2, 87.2, 86.4, 56.1, 54.7.

N, N-dibenzyl-3-(4-bromophenyl)-1-phenylprop-2-yn-1-amine (4ag)

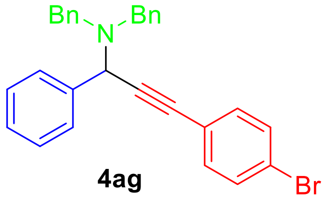

4ag

Prepared according to general procedure (B), **4ag** was purified by flash chromatography (PE-EA, v/v 10/1) as yellow solid with overall isolated yield: 98% (91.1 mg). ^{1}**H NMR** (600 MHz, $CDCl_3$) δ 7.75 (d, *J* = 7.9 Hz, 2H), 7.57 (d, *J* = 8.5 Hz, 2H), 7.52 (d, *J* = 8.5 Hz, 2H), 7.48 (d, *J* = 7.5 Hz, 4H), 7.41 (t, *J* = 7.7 Hz, 2H), 7.37 (t, *J* = 7.6 Hz, 4H), 7.30 (dt, *J* = 18.9, 7.3 Hz, 3H), 4.98 (s, 1H), 3.85 (d, *J* = 13.5 Hz, 2H), 3.57 (d, *J* = 13.5 Hz, 2H). ^{13}**C NMR** (151 MHz, $CDCl_3$) δ 139.4, 138.9, 133.4, 131.6, 128.9, 128.3, 128.2, 128.1, 127.6, 127.1, 122.4, 122.2, 87.6, 86.1, 56.1, 54.7.

4-(3-(dibenzylamino)-3-phenylprop-1-yn-1-yl) benzonitrile (4ah)

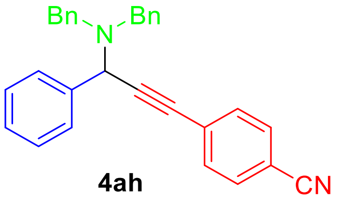

4ah

Prepared according to general procedure (A), **4ah** was purified by flash chromatography (PE-EA, v/v 10/1) as pale-yellow oil with overall isolated yield: 91% (75.0 mg). ^{1}**H NMR** (600 MHz, $CDCl_3$) δ 7.71 – 7.66 (m, 6H), 7.41 (d, *J* = 7.5 Hz, 4H), 7.37 (t, *J* = 7.6 Hz, 2H), 7.32 (t, *J* = 7.6 Hz, 4H), 7.28 (t, *J* = 7.3 Hz, 1H), 7.26 – 7.23 (m, 2H), 4.97 (s, 1H), 3.81 (d, *J* = 13.5 Hz, 2H), 3.50 (d, *J* = 13.5 Hz, 2H). ^{13}**C NMR**

(151 MHz, $CDCl_3$) δ 139.2, 138.4, 132.5, 132.1, 128.9, 128.4, 128.3, 128.2, 128.1, 127.8, 127.2, 111.7, 89.9, 87.2, 56.2, 54.7.

N, N-dibenzyl-1-phenyl-3-(4-(trifluoromethyl) phenyl) prop-2-yn-1-amine (4ai)

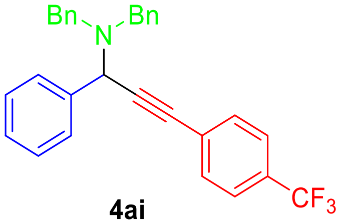

4ai

Prepared according to general procedure (B), **4ai** was purified by flash chromatography (PE-EA, v/v 10/1) as white solid with overall isolated yield: 90% (81.9 mg). **^{1}H NMR** (600 MHz, $CDCl_3$) δ 7.75 (s, 4H), 7.69 (d, *J* = 7.9 Hz, 2H), 7.48 (d, *J* = 7.0 Hz, 4H), 7.41 (t, *J* = 7.5 Hz, 2H), 7.37 (t, *J* = 7.4 Hz, 4H), 7.30 (dt, *J* = 21.5, 7.2 Hz, 3H), 5.02 (s, 1H), 3.87 (d, *J* = 13.4 Hz, 2H), 3.58 (d, *J* = 13.5 Hz, 2H). **^{13}C NMR** (151 MHz, $CDCl_3$) δ 139.3, 138.7, 132.2, 130.1 (d, *J* = 32.6 Hz), 128.9, 128.3, 128.2, 127.7, 127.1, 127.0, 125.3 (q, *J* = 3.7 Hz), 124.9, 123.1, 87.7, 87.4, 56.1, 54.7. **^{19}F NMR** (565 MHz, $CDCl_3$) δ -62.68.

4-(3-(dibenzylamino)-3-phenylprop-1-yn-1-yl)-N, N-dimethylaniline (4aj)

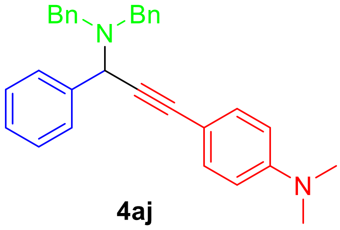

4aj

Prepared according to general procedure (B), **4aj** was purified by flash chromatography (PE-EA, v/v 10/1) as colorless oil with overall isolated yield: 91% (78.3 mg). **^{1}H NMR** (600 MHz, $CDCl_3$) δ 7.74 (d, *J* = 8.0 Hz, 2H), 7.50 (d, *J* = 8.8 Hz, 2H), 7.43 (d, *J* = 7.5 Hz, 4H), 7.34 (t, *J* = 7.7 Hz, 2H), 7.31 (t, *J* = 7.6 Hz, 4H), 7.25 – 7.20 (m, 3H), 6.71 (d, *J* = 8.8 Hz, 2H), 4.91 (s, 1H), 3.77 (d, *J* = 13.5 Hz, 2H), 3.55 (d, *J* = 13.5 Hz, 2H), 3.01 (s, 6H). **^{13}C NMR** (151 MHz, $CDCl_3$) δ 150.2, 139.8, 139.8, 133.0, 128.9, 128.4, 128.2, 128.0, 127.3, 126.9, 112.0, 110.3, 89.4, 82.0, 56.2, 54.6, 40.3.

N, N-dibenzyl-3-(4-(tert-butyl) phenyl)-1-phenylprop-2-yn-1-amine (4ak)

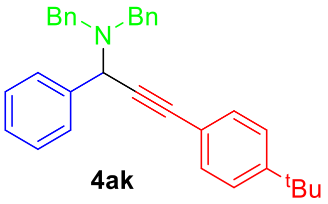

4ak

Prepared according to general procedure (B), **4ak** was purified by flash chromatography (PE-EA, v/v 10/1) as colorless oil with overall isolated yield: 99% (87.7 mg). **^{1}H NMR** (600 MHz, $CDCl_3$) δ 7.64 (d, *J* = 7.7 Hz, 2H), 7.48 (d, *J* = 8.2 Hz, 2H), 7.34 (t, *J* = 7.1 Hz, 6H), 7.24 (dt, *J* = 20.3, 7.6 Hz, 6H), 7.18 – 7.11 (m, 3H), 4.84 (s, 1H), 3.70 (d, *J* = 13.7 Hz, 2H), 3.45 (d, *J* = 13.5 Hz, 2H), 1.27 (s, 9H). **^{13}C NMR** (151 MHz, $CDCl_3$) δ 151.5, 139.6, 139.3, 131.7, 128.9, 128.3, 128.3, 128.1, 127.4, 127.0, 125.4, 120.3, 88.7, 84.0, 56.1, 54.6, 34.8, 31.2.

N, N-dibenzyl-3-cyclohexyl-1-phenylprop-2-yn-1-amine (4al)

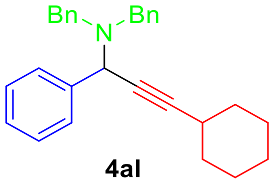

4al

Prepared according to general procedure (B), **4al** was purified by flash chromatography (PE-EA, v/v 10/1) as colorless oil with overall isolated yield: 73% (57.4 mg). **^{1}H NMR** (600 MHz, $CDCl_3$) δ 7.58 (d, *J* = 0.8 Hz, 2H), 7.31 (s, 4H), 7.22 (d, *J* = 7.3 Hz, 6H), 7.13 (d, *J* = 3.5 Hz, 3H), 4.61 (s, 1H), 3.61 (d, *J* = 13.4 Hz, 2H), 3.35 (d, *J* = 13.4 Hz, 2H), 2.57 (s, 1H), 1.86 (s, 2H), 1.76 (s, 2H), 1.52 (d, *J* = 44.3 Hz, 3H), 1.35 (s, 3H). **^{13}C NMR** (151 MHz, $CDCl_3$) δ 139.9, 139.8, 128.9, 128.3, 128.2, 127.9, 127.2, 126.9, 93.2, 74.5, 55.6, 54.5, 33.3, 29.2, 26.0, 24.8.

N, N-dibenzyl-4,4-dimethyl-1-phenylpent-2-yn-1-amine (4am)

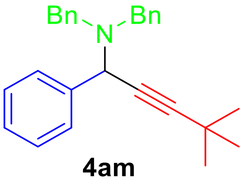

4am

Prepared according to general procedure (B), **4am** was purified by flash chromatography (PE-EA, v/v 10/1) as pale-yellow oil with overall isolated yield: 77% (56.5 mg). **^{1}H NMR** (600 MHz, $CDCl_3$) δ 7.56 (d, *J* = 5.9 Hz, 2H), 7.31 (d, *J* = 6.3 Hz, 4H), 7.25 – 7.17 (m, 6H), 7.15 – 7.07 (m, 3H), 4.58 (s, 1H), 3.60 (d, *J* = 13.3 Hz, 2H), 3.31 (d, *J* = 13.3 Hz, 2H), 1.32 (s, 9H). **^{13}C NMR** (151 MHz, $CDCl_3$) δ 139.9, 139.8, 128.9, 128.3, 128.2, 127.9, 127.2, 126.9, 97.8, 72.9, 55.4, 54.5, 31.6, 27.8.

Ethyl 4-(dibenzylamino)-4-phenylbut-2-ynoate (4an)

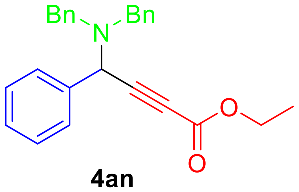

4an

Prepared according to general procedure (B), **4an** was purified by flash chromatography (PE-EA, v/v 10/1) as pale-yellow oil with overall isolated yield: 97% (74.3 mg). **^{1}H NMR** (600 MHz, $CDCl_3$) δ 7.61 (d, *J* = 7.9 Hz, 2H), 7.39 (d, *J* = 7.5 Hz, 4H), 7.35 (t, *J* = 7.6 Hz, 2H), 7.31 (t, *J* = 7.6 Hz, 4H), 7.27 (t, *J* = 7.3 Hz, 1H), 7.25 – 7.21 (m, 2H), 4.85 (s, 1H), 4.34 (q, *J* = 7.1 Hz, 2H), 3.78 (d, *J* = 13.5 Hz, 2H), 3.47 (d, *J* = 13.5 Hz, 2H), 1.40 (t, *J* = 7.2 Hz, 3H). **^{13}C NMR** (151 MHz, $CDCl_3$) δ 153.6, 138.8, 137.1, 128.9, 128.4, 128.3, 128.1, 127.9, 127.2, 83.4, 80.5, 62.2, 55.6, 54.5, 14.1.

N, N-dibenzyl-1-phenylhept-2-yn-1-amine (4ao)

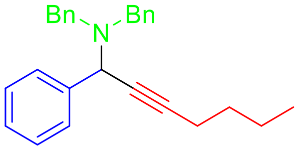

4ao

Prepared according to general procedure (B), **4ao** was purified by flash chromatography (PE-EA, v/v 10/1) as colorless oil with overall isolated yield: 85% (62.4 mg). **^{1}H NMR** (600 MHz, $CDCl_3$) δ 7.58 (d, *J* = 7.6 Hz, 2H), 7.32 (d, *J* = 7.4 Hz, 4H), 7.22 (dt, *J* = 14.9, 7.6 Hz, 6H), 7.16 – 7.11 (m, 3H), 4.60 (s, 1H), 3.61 (d, *J* = 13.5 Hz, 2H), 3.35 (d, *J* = 13.5 Hz, 2H), 2.35 (t, *J* = 7.0 Hz, 2H), 1.65 – 1.55 (m, 2H), 1.55 – 1.44 (m, 2H), 0.93 (t, *J* = 7.3 Hz, 3H). **^{13}C NMR** (151 MHz, $CDCl_3$) δ 139. 9, 139.8, 128.9, 128.3, 128.2, 127.9, 127.2, 126.9, 88.8, 74.7, 55.6, 54.5, 31.4, 22.1, 18.6, 13.7.

N, N-dibenzyl-1-phenyl-3-(thiophen-2-yl) prop-2-yn-1-amine (4ap)

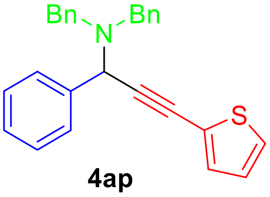

4ap

Prepared according to general procedure (B), **4ap** was purified by flash chromatography (PE-EA, v/v 10/1) as pale-yellow oil with overall isolated yield: 98% (77.0 mg). **^{1}H NMR** (600 MHz, $CDCl_3$) δ 7.77 (d, *J* = 7.3 Hz, 2H), 7.50 (d, *J* = 7.3 Hz, 4H), 7.41 (dt, *J* = 22.6, 7.6 Hz, 7H), 7.32 (dt, *J* = 14.5, 6.1 Hz, 4H), 7.12 – 7.08 (m, 1H), 5.03 (s, 1H), 3.87 (d, *J* = 13.5 Hz, 2H), 3.59 (d, *J* = 13.5 Hz, 2H). **^{13}C NMR** (151 MHz, $CDCl_3$) δ 139.4, 138.9, 132.0, 128.9, 128.3, 128.2, 128.1, 127.5, 127.0, 127.0, 126.8, 123.2, 89.0, 81.6, 56.4, 54.6.

N, N-dibenzyl-1-phenyl-3-(thiophen-3-yl) prop-2-yn-1-amine (4aq)

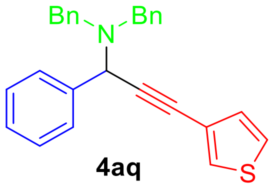

4aq

Prepared according to general procedure (B), **4aq** was purified by flash chromatography (PE-EA, v/v 10/1) as pale-yellow oil with overall isolated yield: 97% (76.2 mg). **^{1}H NMR** (600 MHz, $CDCl_3$) δ 7.63 (d, J = 7.8 Hz, 2H), 7.49 (d, J = 2.7 Hz, 1H), 7.34 (d, J = 7.5 Hz, 4H), 7.24 (ddd, J = 15.1, 14.4, 7.6 Hz, 7H), 7.16 (ddd, J = 17.9, 7.6, 1.9 Hz, 4H), 4.83 (s, 1H), 3.70 (d, J = 13.4 Hz, 2H), 3.44 (d, J = 13.5 Hz, 2H). **^{13}C NMR** (151 MHz, $CDCl_3$) δ 139.5, 139.1, 130.3, 128.9, 128.6, 128.3, 128.1, 127.5, 127.0, 125.3, 122.2, 84.3, 83.5, 56.1, 54.6.

N, N-dibenzyl-1-phenyl-3-(pyrimidin-2-yl) prop-2-yn-1-amine (4ar)

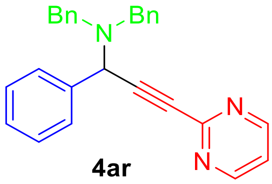

4ar

Prepared according to general procedure (B), **4ar** was purified by flash chromatography (PE-EA, v/v 5/1) as pale-yellow oil with overall isolated yield: 90% (70.0 mg). **^{1}H NMR** (600 MHz, $CDCl_3$) δ 8.82 (d, J = 4.9 Hz, 2H), 7.77 (d, J = 7.8 Hz, 2H), 7.47 (d, J = 7.5 Hz, 4H), 7.38 (t, J = 7.6 Hz, 2H), 7.34 (t, J = 7.5 Hz, 4H), 7.32 – 7.24 (m, 4H), 5.04 (s, 1H), 3.88 (d, J = 13.5 Hz, 2H), 3.66 (d, J = 13.6 Hz, 2H). **^{13}C NMR** (151 MHz, $CDCl_3$) δ 157.3, 153.1, 139.2, 138.1, 128.9, 128.3, 128.2, 128.2, 127.7, 127.1, 119.9, 87.7, 84.5, 56.0, 54.6.

N, N-dibenzyl-1-phenyl-3-(pyridin-4-yl) prop-2-yn-1-amine (4as)

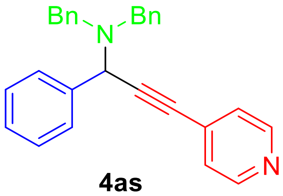

4as

Prepared according to general procedure (B), **4as** was purified by flash chromatography (PE-EA, v/v 5/1) as white solid with overall isolated yield: 92% (71.4 mg). **^{1}H NMR** (600 MHz, $CDCl_3$) δ 8.69 (d, J = 6.0 Hz, 2H), 7.73 (d, J = 7.9 Hz, 2H), 7.51 (dd, J = 4.5, 1.5 Hz, 2H), 7.47 (d, J = 7.5 Hz, 4H), 7.41 (t, J = 7.7 Hz, 2H), 7.38 – 7.36 (m, 4H), 7.32 (t, J = 7.4 Hz, 1H), 7.28 (t, J = 7.4 Hz, 2H), 5.02 (s, 1H), 3.86 (d, J = 17.4 Hz, 2H), 3.56 (d, J = 13.5 Hz, 2H). **^{13}C NMR** (151 MHz, $CDCl_3$) δ 149.8, 140.3, 139.1, 138.3, 131.3, 128.8, 128.3, 128.2, 128.1, 127.7, 127.1, 126.9, 126.0, 90.2, 86.2, 56.1, 54.6.

N, N-dibenzyl-1-phenyl-3-(pyridin-3-yl)prop-2-yn-1-amine (4at)

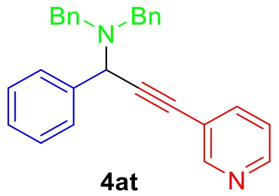

4at

Prepared according to general procedure (B), **4at** was purified by flash chromatography (PE-EA, v/v 5/1) as colorless oil with overall isolated yield: 95% (73.7 mg). **^{1}H NMR** (600 MHz, $CDCl_3$) δ 8.91 (s, 1H), 8.64 (d, *J* = 3.7 Hz, 1H), 7.93 (d, *J* = 7.8 Hz, 1H), 7.75 (d, *J* = 7.8 Hz, 2H), 7.48 (d, *J* = 7.5 Hz, 4H), 7.41 (t, *J* = 7.7 Hz, 2H), 7.39 – 7.35 (m, 5H), 7.33 – 7.26 (m, 3H), 5.02 (s, 1H), 3.87 (d, *J* = 13.4 Hz, 2H), 3.58 (d, *J* = 13.5 Hz, 2H). **^{13}C NMR** (151 MHz, $CDCl_3$) δ 152.6, 148.6, 139.2, 138.8, 138.6, 128.8, 128.3, 128.2, 128.2, 127.6, 127.1, 123.0, 120.3, 88. 5, 85.3, 56.1, 54.6.

3-([1,1'-biphenyl]-4-yl)-N, N-dibenzyl-1-phenylprop-2-yn-1-amine (4au)

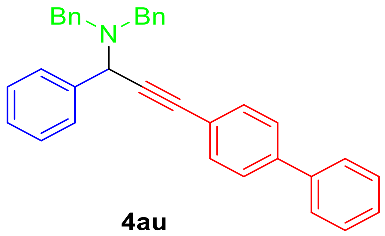

4au

Prepared according to general procedure (B), **4au** was purified by flash chromatography (PE-EA, v/v 10/1) as pale-yellow solid with overall isolated yield: 93% (86.1 mg). **^{1}H NMR** (600 MHz, $CDCl_3$) δ 7.80 (d, *J* = 7.9 Hz, 2H), 7.75 (d, *J* = 8.3 Hz, 2H), 7.68 (d, *J* = 8.3 Hz, 4H), 7.51 (dd, *J* = 15.0, 7.3 Hz, 6H), 7.45 – 7.40 (m, 3H), 7.38 (t, *J* = 7.6 Hz, 4H), 7.30 (dt, *J* = 19.1, 7.3 Hz, 3H), 5.02 (s, 1H), 3.87 (d, *J* = 13.5 Hz, 2H), 3.62 (d, *J* = 13.5 Hz, 2H). **^{13}C NMR** (151 MHz, $CDCl_3$) δ 141.1, 140.4, 139.5, 139.2, 132.4, 128.9, 128.9, 128.3, 128.1, 127.7, 127.5, 127.1, 127.1, 127.0, 122.2, 88.5, 85.5, 56.2, 54.7.

N, N-dibenzyl-3-(3,4-difluorophenyl)-1-phenylprop-2-yn-1-amine (4av)

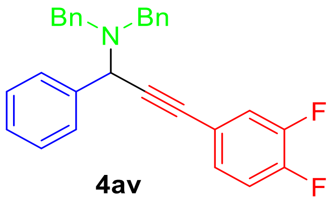

4av

Prepared according to general procedure (B), **4av** was purified by flash chromatography (PE-EA, v/v 10/1) as white solid with overall isolated yield: 91% (77.0 mg). **^{1}H NMR** (600 MHz, $CDCl_3$) δ 7.60 (d, *J* = 7.5 Hz, 2H), 7.33 (d, *J* = 7.5 Hz, 5H), 7.29 – 7.21 (m, 7H), 7.16 (dt, *J* = 21.2, 7.9 Hz, 3H), 7.08 (dt, *J* = 10.1, 8.3 Hz, 1H), 4.84 (s, 1H), 3.71 (d, *J* = 13.5 Hz, 2H), 3.41 (d, *J* = 13.5 Hz, 2H). **^{13}C NMR** (151 MHz, $CDCl_3$) δ 151.1 (dd, *J* = 80.8, 12.8 Hz), 149.5 (dd, *J* = 78.6, 12.8 Hz), 139.3, 138.8, 128.9, 128.5 (dd, *J* = 6.3, 3.5 Hz), 128.3, 128.2, 127.6, 127.1, 120.9 (d, *J* = 18.2 Hz), 120.0 (dd, *J* = 7.6,

4.1 Hz), 117.5 (d, J = 17.8 Hz), 86.6, 85.6, 56.0, 54.7. ^{19}**F NMR** (565 MHz, $CDCl_3$) δ -135.97 (d, J = 21.3 Hz), -137.06 (d, J = 21.4 Hz).

N, N-dibenzyl-3-(naphthalen-2-yl)-1-phenylprop-2-yn-1-amine (4aw)

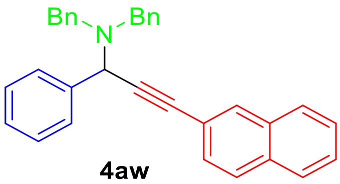

4aw

Prepared according to general procedure (B), **4aw** was purified by flash chromatography (PE-EA, v/v 10/1) as yellow oil with overall isolated yield: 99% (86.5 mg). ^{1}**H NMR** (600 MHz, $CDCl_3$) δ 8.21 (d, J = 4.0 Hz, 1H), 7.92 (dd, J = 7.9, 3.4 Hz, 3H), 7.85 (t, J = 6.1 Hz, 2H), 7.74 (dd, J = 8.2, 4.1 Hz, 1H), 7.60 – 7.51 (m, 6H), 7.42 (ddd, J = 28.0, 11.8, 6.8 Hz, 6H), 7.37 – 7.28 (m, 3H), 5.08 (s, 1H), 3.92 (d, J = 13.4 Hz, 2H), 3.69 (d, J = 13.5 Hz, 2H). ^{13}**C NMR** (151 MHz, $CDCl_3$) δ 139.5, 139.2, 133.1, 132.8, 131.6, 128.9, 128.8, 128.3, 128.1, 128.0, 127.8, 127.7, 127.5, 127.0, 126.6, 120.5, 89.0, 85.1, 56.2, 54.7.

N, N-dibenzyl-3-(naphthalen-1-yl)-1-phenylprop-2-yn-1-amine (4ax)

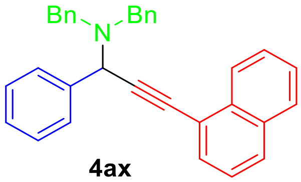

4ax

Prepared according to general procedure (B), **4ax** was purified by flash chromatography (PE-EA, v/v 10/1) as pale-yellow oil with overall isolated yield: 98% (85.6 mg). ^{1}**H NMR** (600 MHz, $CDCl_3$) δ 8.70 – 8.60 (m, 1H), 8.01 – 7.89 (m, 5H), 7.75 – 7.70 (m, 1H), 7.68 – 7.62 (m, 1H), 7.59 (dd, J = 11.1, 4.3 Hz, 5H), 7.51 – 7.31 (m, 9H), 5.23 (s, 1H), 4.00 (d, J = 13.4 Hz, 2H), 3.77 (d, J = 13.4 Hz, 2H). ^{13}**C NMR** (151 MHz, $CDCl_3$) δ 139.5, 139.2, 133.5, 133.3, 130.9, 128.9, 128.7, 128.4, 128.4, 128.3, 128.2, 127.5, 127.1, 126.9, 126.4, 126.3, 125.3, 121.0, 89.8, 86.7, 56.5, 54.8.

N, N-dibenzyl-3-(phenanthren-9-yl)-1-phenylprop-2-yn-1-amine (4ay)

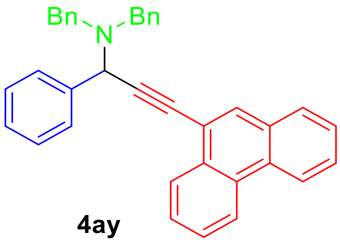

4ay

Prepared according to general procedure (B), **4ay** was purified by flash chromatography (PE-EA, v/v 10/1) as yellow solid with overall isolated yield: 95% (92.5 mg). ^{1}**H NMR** (600 MHz, $CDCl_3$) δ 8.78 (d, J = 7.8 Hz, 1H), 8.73 (t, J = 8.7 Hz, 2H), 8.24 (s, 1H), 7.97 (d, J = 7.7 Hz, 1H), 7.91 (d, J = 6.7 Hz, 2H), 7.80 (s, 2H), 7.73 (t, J = 7.5 Hz, 1H),

7.68 (t, J = 7.3 Hz, 1H), 7.55 (d, J = 6.8 Hz, 4H), 7.46 (t, J = 6.8 Hz, 2H), 7.41 (t, J = 6.8 Hz, 4H), 7.34 (dd, J = 27.6, 6.9 Hz, 3H), 5.22 (s, 1H), 3.99 (d, J = 13.4 Hz, 2H), 3.77 (d, J = 13.4 Hz, 2H). **^{13}C NMR** (151 MHz, $CDCl_3$) δ 139.5, 139.2, 132.3, 131.4, 131.3, 130.3, 130.2, 128.9, 128.5, 128.4, 128.3, 128.2, 127.6, 127.5, 127.1, 127.1, 127.0, 122.9, 122.7, 119.7, 89.5, 86.8, 56.5, 54.9.

3,3'-(1,4-phenylene)bis(N,N-dibenzyl-1-phenylprop-2-yn-1-amine) (4az)

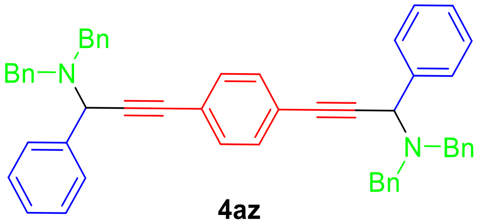

4az

Prepared according to general procedure (C), **4az** was purified by flash chromatography (PE-EA, v/v 10/1) as white solid with overall isolated yield: 82% (57.1 mg). **^{1}H NMR** (600 MHz, $CDCl_3$) δ 7.65 (d, J = 7.6 Hz, 4H), 7.55 (s, 4H), 7.36 (d, J = 7.4 Hz, 8H), 7.29 (t, J = 7.5 Hz, 4H), 7.24 (t, J = 7.4 Hz, 8H), 7.17 (dt, J = 21.5, 7.2 Hz, 6H), 4.89 (s, 2H), 3.74 (d, J = 13.5 Hz, 4H), 3.48 (d, J = 13.5 Hz, 4H). **^{13}C NMR** (151 MHz, $CDCl_3$) δ 139.5, 139.0, 131.9, 128.9, 128.3, 128.3, 128.2, 127. 6, 127.1, 123.1, 88.3, 86.8, 56.2, 54.7.

1-(1,3-diphenylprop-2-yn-1-yl) azocane (4ba)

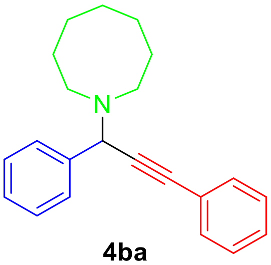

4ba

Prepared according to general procedure (B), **4ba** was purified by flash chromatography (PE-EA, v/v 10/1) as colorless oil with overall isolated yield: 90% (54.5 mg). **^{1}H NMR** (600 MHz, $CDCl_3$) δ 7.70 (d, J = 7.6 Hz, 2H), 7.52 (dd, J = 7.7, 1.8 Hz, 2H), 7.38 – 7.30 (m, 5H), 7.27 (t, J = 7.3 Hz, 1H), 4.91 (s, 1H), 2.74 – 2.63 (m, 4H), 1.68 (dd, J = 10.7, 5.2 Hz, 2H), 1.64 – 1.52 (m, 6H), 1.48 – 1.39 (m, 2H). **^{13}C NMR** (151 MHz, $CDCl_3$) δ 139.9, 131.8, 128.6, 128.2, 127.9, 127.9, 127.2, 123.5, 87.2, 86.8, 62.6, 51.29, 28.0, 27.8, 25.9.

4-(1,3-diphenylprop-2-yn-1-yl) thiomorpholine (4bb)

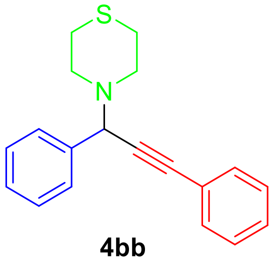

4bb

Prepared according to general procedure (B), **4bb** was purified by flash chromatography (PE-EA, v/v 10/1) as white solid with overall isolated yield: 95% (55.7 mg). **^{1}H NMR** (600 MHz, $CDCl_3$) δ 7.65 (d, *J* = 7.6 Hz, 2H), 7.54 (dd, *J* = 6.5, 3.0 Hz, 2H), 7.40 – 7.29 (m, 6H), 4.83 (s, 1H), 2.90 (t, *J* = 4.9 Hz, 4H), 2.78 – 2.65 (m, 4H). **^{13}C NMR** (151 MHz, $CDCl_3$) δ 138.0, 131.8, 128.4, 128.3, 128.2, 128.2, 127.7, 123.0, 88.5, 84.9, 62.8, 51.8, 28.3.

1-(1,3-diphenylprop-2-yn-1-yl) pyrrolidine (4bc)

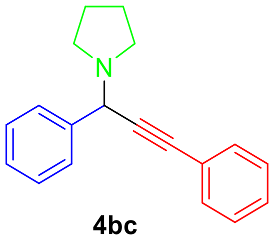

4bc

Prepared according to general procedure (B), **4bc** was purified by flash chromatography (PE-EA, v/v 10/1) as colorless oil with overall isolated yield: 83% (43.3 mg). **^{1}H NMR** (600 MHz, $CDCl_3$) δ 7.61 (d, *J* = 7.5 Hz, 2H), 7.49 (dd, *J* = 6.5, 3.0 Hz, 2H), 7.36 (t, *J* = 7.6 Hz, 2H), 7.34 – 7.27 (m, 4H), 4.89 (s, 1H), 2.70 (t, *J* = 6.5 Hz, 4H), 1.84 – 1.77 (m, 4H). **^{13}C NMR** (151 MHz, $CDCl_3$) δ 139.6, 131.8, 128.3, 128.3, 128.3, 128.1, 127.6, 123.3, 86.9, 86.8, 59.1, 50.3, 23.5.

4-(1,3-diphenylprop-2-yn-1-yl) morpholine (4bd)

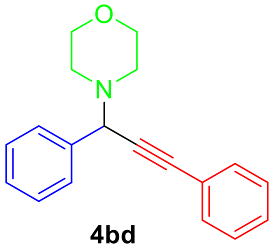

4bd

Prepared according to general procedure (B), **4bd** was purified by flash chromatography (PE-EA, v/v 10/1) as colorless oil with overall isolated yield: 72% (39.9 mg). **^{1}H NMR** (600 MHz, $CDCl_3$) δ 7.64 (d, *J* = 7.5 Hz, 2H), 7.52 (dt, *J* = 4.9, 3.0 Hz, 2H), 7.38 (t, *J* = 7.5 Hz, 2H), 7.35 – 7.29 (m, 4H), 4.80 (s, 1H), 3.79 – 3.69 (m, 4H), 3.71 – 3.60 (m, 4H). **^{13}C NMR** (151 MHz, $CDCl_3$) δ 137.8, 131.8, 128.6, 128.3, 128.2, 128.2, 127.7, 123.0, 88.5, 85.1, 67.2, 62.1, 49.9.

N-benzyl-N-(1,3-diphenylprop-2-yn-1-yl) aniline (4be)

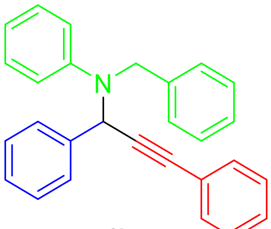

4be

Prepared according to general procedure (B), **4be** was purified by flash chromatography (PE-EA, v/v 10/1) as colorless oil with overall isolated yield: 64% (47.7 mg). **^{1}H NMR** (600 MHz, $CDCl_3$) δ 7.62 (d, *J* = 7.7 Hz, 2H), 7.33 (t, *J* = 8.0 Hz, 6H), 7.28 – 7.21 (m, 6H), 7.17 (dd, *J* = 15.9, 7.5 Hz, 3H), 6.95 (d, *J* = 8.2 Hz, 2H), 6.79 (t, *J* = 7.3 Hz, 1H), 6.05 (s, 1H), 4.60 (d, *J* = 16.6 Hz, 1H), 4.47 (d, *J* = 16.6 Hz, 1H). **^{13}C NMR** (151 MHz, $CDCl_3$) δ 148.5, 139.3, 138.4, 131.7, 128.9, 128.4, 128.2, 128.2, 128.2, 127.7, 127.7, 127.2, 126.5, 122.8, 119.1, 116.5, 87.0, 86.5, 57.0, 52.7.

N-(1,3-diphenylprop-2-yn-1-yl)-N-methylaniline (4bf)

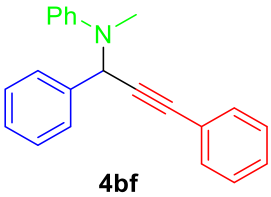

4bf

Prepared according to general procedure (B), **4bf** was purified by flash chromatography (PE-EA, v/v 10/1) as pale-yellow oil with overall isolated yield: 70% (41.6 mg). **^{1}H NMR** (600 MHz, $CDCl_3$) δ 7.63 (d, *J* = 7.7 Hz, 2H), 7.48 – 7.45 (m, 2H), 7.38 (t, *J* = 7.6 Hz, 2H), 7.34 – 7.27 (m,, 6H), 7.03 (d, *J* = 8.3 Hz, 2H), 6.85 (t, *J* = 7.3 Hz, 1H), 6.00 (s, 1H), 2.78 (s, 3H). **^{13}C NMR** (151 MHz, $CDCl_3$) δ 150.3, 138.6, 131.8, 129.2, 128.4, 128.3, 127.7, 127.6, 123.0, 118.8, 115.3, 87.0, 85.8, 57.1, 33.8.

N-(1,3-diphenylprop-2-yn-1-yl)-N,3-dimethylaniline (4bg)

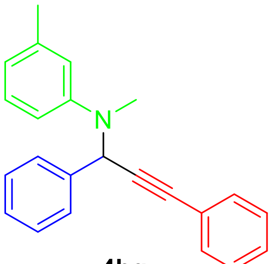

4bg

Prepared according to general procedure (B), **4bg** was purified by flash chromatography (PE-EA, v/v 10/1) as pale-yellow oil with overall isolated yield: 68% (42.3 mg). **^{1}H NMR** (600 MHz, $CDCl_3$) δ 7.66 (d, *J* = 7.5 Hz, 2H), 7.53 – 7.47 (m, 2H), 7.41 (t, *J* = 7.6 Hz, 2H), 7.37 – 7.31 (m, 4H), 7.22 (t, *J* = 11.9 Hz, 1H), 6.88 (d, *J* = 6.4 Hz, 2H), 6.71 (d, *J* = 7.5 Hz, 1H), 6.03 (s, 1H), 2.79 (s, 3H), 2.38 (s, 3H). **^{13}C NMR**

(151 MHz, $CDCl_3$) δ 150.4, 138.9, 138.7, 131.8, 129.0, 128.4, 128.3, 128.2, 127.7, 127.6, 123.0, 119.7, 116.1, 112.4, 87.0, 85.9, 57.0, 33.7, 21.9.

N-(1,3-diphenylprop-2-yn-1-yl)-N,2-dimethylaniline (4bh)

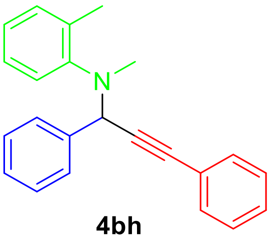

4bh

Prepared according to general procedure (B), **4bh** was purified by flash chromatography (PE-EA, v/v 10/1) as pale-yellow oil with overall isolated yield: 62% (38.5 mg). **^{1}H NMR** (600 MHz, $CDCl_3$) δ 7.76 (d, J = 7.7 Hz, 2H), 7.50 (dd, J = 6.6, 3.0 Hz, 2H), 7.45 – 7.37 (m, 3H), 7.34 – 7.30 (m, 4H), 7.21 (dd, J = 15.3, 7.5 Hz, 2H), 7.05 (t, J = 7.3 Hz, 1H), 5.32 (s, 1H), 2.62 (s, 3H), 2.47 (s, 3H). **^{13}C NMR** (151 MHz, $CDCl_3$) δ 151.0, 138.6, 133.3, 131.8, 131.0, 128.3, 128.2, 128.2, 128.1, 127.6, 126.4, 123.9, 123.2, 122.4, 88.0, 86.3, 60.0, 35.5, 18.5.

N-(1,3-diphenylprop-2-yn-1-yl)-2-fluoro-N-methylaniline (4bi)

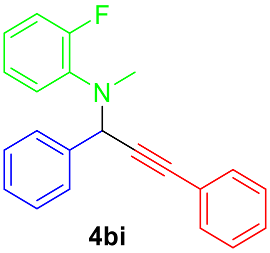

4bi

Prepared according to general procedure (B), **4bi** was purified by flash chromatography (PE-EA, v/v 10/1) as pale-yellow oil with overall isolated yield: 60% (37.8 mg). **^{1}H NMR** (600 MHz, $CDCl_3$) δ 7.77 (d, J = 7.7 Hz, 2H), 7.49 (dd, J = 6.5, 3.1 Hz, 2H), 7.41 (t, J = 7.6 Hz, 2H), 7.37 – 7.31 (m, 4H), 7.20 (t, J = 8.2 Hz, 1H), 7.11 (t, J = 9.5 Hz, 2H), 7.03 – 6.97 (m, 1H), 5.75 (s, 1H), 2.67 (s, 3H). **^{13}C NMR** (151 MHz, $CDCl_3$) δ 156.1 (d, J = 245.2 Hz), 139.7 (d, J = 8.5 Hz), 138.1, 131.8, 128.3, 128.3, 128.1, 127.8, 124.3 (d, J = 3.6 Hz), 123.0, 122.8 (d, J = 7.9 Hz), 121.4 (d, J = 2.6 Hz), 116.1 (d, J = 20.9 Hz), 88.2, 85.2, 59.3 (d, J = 5.7 Hz), 33.8. **^{19}F NMR** (565 MHz, $CDCl_3$) δ -122.02.

N-(1,3-diphenylprop-2-yn-1-yl)-4-fluoro-N-methylaniline (4bj)

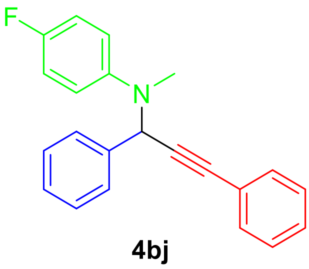

4bj

Prepared according to general procedure (B), **4bj** was purified by flash chromatography (PE-EA, v/v 10/1) as yellow oil with overall isolated yield: 72% (45.3 mg). **^{1}H NMR** (600 MHz, $CDCl_3$) δ 7.65 (d, *J* = 7.4 Hz, 2H), 7.50 – 7.46 (m, 2H), 7.41 (t, *J* = 7.7 Hz, 2H), 7.37 – 7.31 (m, 4H), 7.01 (d, *J* = 6.2 Hz, 4H), 5.84 (s, 1H), 2.75 (s, 3H). **^{13}C NMR** (151 MHz, $CDCl_3$) δ 157.0 (d, *J* = 238.2 Hz), 147.0, 138.3, 131.8, 128.4, 128.3 (d, *J* = 6.0 Hz), 127.8, 127.7, 122.9, 117.9 (d, *J* = 7.6 Hz), 115.5 (d, *J* = 22.1 Hz), 87.5, 85.4, 58.7, 34.6. **^{19}F NMR** (565 MHz, $CDCl_3$) δ -125.29.

N-(1,3-diphenylprop-2-yn-1-yl)-N-ethylaniline (4bk)

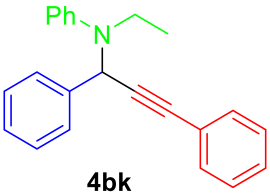

4bk

Prepared according to general procedure (B), **4bk** was purified by flash chromatography (PE-EA, v/v 10/1) as pale-yellow oil with overall isolated yield: 61% (37.9 mg). **^{1}H NMR** (600 MHz, $CDCl_3$) δ 7.64 (d, *J* = 7.8 Hz, 2H), 7.47 (dd, *J* = 6.5, 3.0 Hz, 2H), 7.37 (t, *J* = 7.6 Hz, 2H), 7.33 – 7.26 (m, 6H), 6.99 (d, *J* = 8.2 Hz, 2H), 6.82 (t, *J* = 7.2 Hz, 1H), 5.90 (s, 1H), 3.43 – 3.32 (m, 2H), 1.13 (t, *J* = 7.0 Hz, 3H). **^{13}C NMR** (151 MHz, $CDCl_3$) δ 148.3, 139.0, 131.7, 129.1, 128.4, 128.3, 128.2, 127.7, 127.6, 123.1, 118.4, 115.9, 87.0, 86.5, 56.6, 42.3, 29.7.

1-(1,3-diphenylprop-2-yn-1-yl) piperidine (4bl)

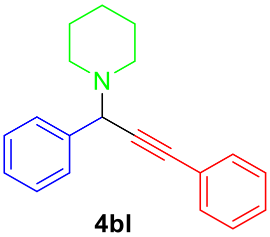

4bl

Prepared according to general procedure (B), **4bl** was purified by flash chromatography (PE-EA, v/v 10/1) as colorless oil with overall isolated yield: 93% (51.1 mg). **^{1}H NMR** (600 MHz, $CDCl_3$) δ 7.66 (d, *J* = 7.3 Hz, 2H), 7.54 (dt, *J* = 4.8, 3.0 Hz, 2H), 7.38 (t, *J* = 7.6 Hz, 2H), 7.36 – 7.33 (m, 3H), 7.31 (t, *J* = 7.3 Hz, 1H), 4.82 (s, 1H), 2.65 – 2.52 (m, 4H), 1.70 – 1.55 (m, 4H), 1.52 – 1.43 (m, 2H). **^{13}C NMR** (151 MHz, $CDCl_3$) δ 138.7, 131.8, 128.5, 128.2, 128.0, 128.0, 127.4, 123.4, 87.8, 86.1, 62.4, 50.7, 26.2, 24.5.

1-(1,3-diphenylprop-2-yn-1-yl)-4-ethylpiperazine (4bm)

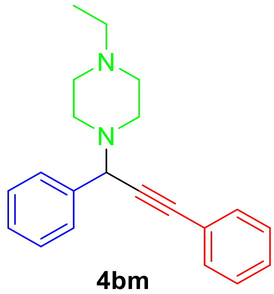

4bm

Prepared according to general procedure (B), **4bm** was purified by flash chromatography (DCM-EtOH, v/v 20/1) as yellow oil with overall isolated yield: 91% (55.3 mg). ^{1}**H NMR** (600 MHz, $CDCl_3$) δ 7.63 (d, *J* = 7.5 Hz, 2H), 7.52 – 7.48 (m, 2H), 7.36 (t, *J* = 7.6 Hz, 2H), 7.33 – 7.27 (m, 4H), 4.82 (s, 1H), 2.70 (s, 4H), 2.62 – 2.24 (m, 6H), 1.09 (t, *J* = 7.2 Hz, 3H). ^{13}**C NMR** (151 MHz, $CDCl_3$) δ 138.3, 131.8, 128.5, 128.2, 128.1, 128.1, 127.6, 123.1, 88.2, 85.5, 61.6, 53.0, 52.3, 11.9.

1-(1,3-diphenylprop-2-yn-1-yl)-4-phenylpiperazine (4bn)

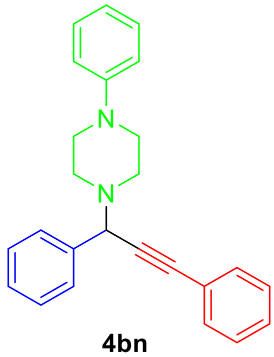

4bn

Prepared according to general procedure (B), **4bn** was purified by flash chromatography (DCM-EtOH, v/v 20/1) as colorless oil with overall isolated yield: 90% (63.4 mg). ^{1}**H NMR** (600 MHz, $CDCl_3$) δ 7.67 (d, *J* = 7.4 Hz, 2H), 7.51 (dd, *J* = 3.1, 1.6 Hz, 2H), 7.38 (t, *J* = 7.4 Hz, 2H), 7.34 – 7.29 (m, 4H), 7.27 – 7.22 (m, 2H), 6.92 (d, *J* = 7.9 Hz, 2H), 6.84 (t, *J* = 7.3 Hz, 1H), 4.89 (s, 1H), 3.28 – 3.16 (m, 4H), 2.85 – 2.75 (m, 4H). ^{13}**C NMR** (151 MHz, $CDCl_3$) δ 151.4, 138.1, 131.8, 129.1, 128.5, 128.3, 128.2, 128.2, 127.7, 123.0, 119.6, 116.1, 88.5, 85.2, 61.7, 49.5, 49.3.

1-(1,3-diphenylprop-2-yn-1-yl)-4-methylpiperazine (4bo)

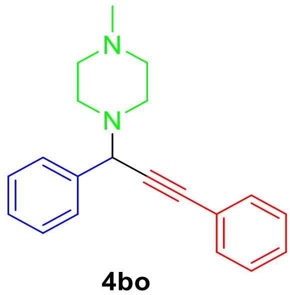

4bo

Prepared according to general procedure (B), **4bo** was purified by flash chromatography (DCM-EtOH, v/v 20/1) as yellow oil with overall isolated yield: 85% (49.3 mg). **^{1}H NMR** (600 MHz, $CDCl_3$) δ 7.64 (d, *J* = 7.5 Hz, 2H), 7.51 (dt, *J* = 4.5, 3.2 Hz, 2H), 7.36 (t, *J* = 7.5 Hz, 2H), 7.32 – 7.28 (m, 4H), 4.83 (s, 1H), 2.69 (s, 4H), 2.50 (s, 4H), 2.30 (s, 3H). **^{13}C NMR** (151 MHz, $CDCl_3$) δ 138.3, 131.8, 128.5, 128.2, 128.1, 128.1, 127.6, 123.1, 88.3, 85.3, 61.6, 55.3, 46.0.

_N_-benzyl-_N_-methyl-1,3-diphenylprop-2-yn-1-amine (4bp)

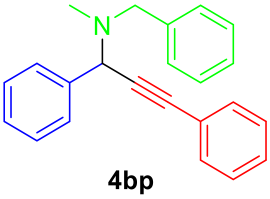

4bp

Prepared according to general procedure (B), **4bp** was purified by flash chromatography (PE-EA, v/v 10/1) as pale-yellow oil with overall isolated yield: 95% (59.1 mg). **^{1}H NMR** (600 MHz, $CDCl_3$) δ 7.69 (d, *J* = 7.7 Hz, 2H), 7.58 (dd, *J* = 7.5, 1.9 Hz, 2H), 7.42 (d, *J* = 7.4 Hz, 2H), 7.35 (ddd, *J* = 20.0, 14.3, 7.6 Hz, 7H), 7.27 (dt, *J* = 18.2, 7.3 Hz, 2H), 4.93 (s, 1H), 3.74 (d, *J* = 13.1 Hz, 1H), 3.65 (d, *J* = 13.1 Hz, 1H), 2.25 (s, 3H). **^{13}C NMR** (151 MHz, $CDCl_3$) δ 139.3, 139.0, 131.9, 129.0, 128.3, 128.3, 128.1, 128.1, 127.5, 127.0, 123.3, 88.6, 84.7, 59.6, 58.9, 38.0.

2-(1,3-diphenylprop-2-yn-1-yl)-1,2,3,4-tetrahydroisoquinoline (4bq)

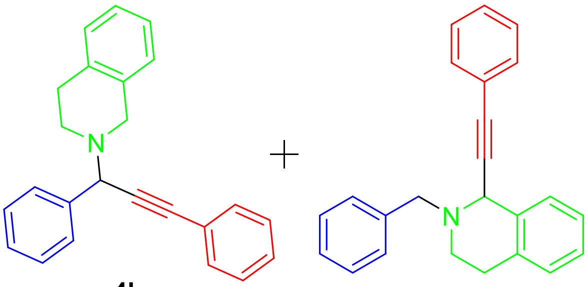

4bq **5a**

Prepared according to general procedure (B), the crude mixture was purified by flash chromatography (PE-EA, v/v 10/1) as yellow oil with overall isolated yield: 94% (60.7 mg, Inseparable mixture of **4bq** and **5a** products, isolated ratio =5:3, original ratio =1:1 Figure S176). **^{1}H NMR** (600 MHz, $CDCl_3$) δ 7.75 (d, *J* = 7.5 Hz, 2H), 7.53 (dd, *J* = 3.9, 1.7 Hz, 2H), 7.51 – 7.45 (m, 2H), 7.40 (t, *J* = 7.1 Hz, 2H), 7.38 – 7.27 (m, 8H), 7.19 (t, *J* = 7.0 Hz, 1H), 7.16 – 7.10 (m, 4H), 7.03 (d, *J* = 7.6 Hz, 1H), 5.09 (s, 1H), 4.82 (s, 0.6H), 3.96 (q, *J* = 13.1 Hz, 1.2H), 3.91 – 3.84 (m, 2H), 3.08 (ddd, *J* = 22.9, 14.2, 5.3 Hz, 1.2H), 3.00 – 2.87 (m, 4H), 2.84 (dd, *J* = 21.5, 10.1 Hz, 1.2H). **^{13}C NMR** (151 MHz, $CDCl_3$) δ 138.4, 138.3, 135.5, 135.3, 134.4, 134.1, 131.8, 131.8, 129.2, 129.0, 128.7, 128.5, 128.3, 128.2, 128.2, 128.0, 127.8, 127.7, 127.1, 126.9, 126.7, 126.0, 125.8, 125.5, 123.3, 123.1, 88.5, 87.6, 86.8, 85.1, 61.6, 59.6, 54.4, 52.2, 47.3, 45.8, 29.7, 29.1.

N, N-dibenzyl-1-(9-ethyl-9H-carbazol-3-yl)-3-phenylprop-2-yn-1-amine (4br)

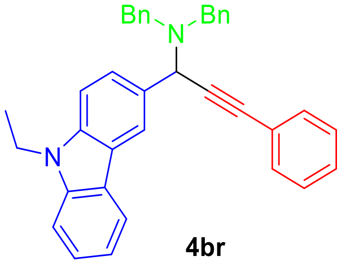

4br

Prepared according to general procedure (A), **4br** was purified by flash chromatography (PE-EA, v/v 10/1) as colorless oil with overall isolated yield: 90% (90.7 mg). **^{1}H NMR** (600 MHz, $CDCl_3$) δ 8.44 (s, 1H), 8.16 – 8.11 (m, 1H), 7.83 – 7.77 (m, 1H), 7.72 – 7.67 (m, 2H), 7.49 – 7.37 (m, 10H), 7.32 (dd, *J* = 9.3, 5.3 Hz, 4H), 7.22 (d, *J* = 4.2 Hz, 3H), 5.15 (s, 1H), 4.34 (q, *J* = 7.0 Hz, 2H), 3.88 (d, *J* = 13.5 Hz, 2H), 3.61 (d, *J* = 13.5 Hz, 2H), 1.41 (t, *J* = 7.1 Hz, 3H). **^{13}C NMR** (151 MHz, $CDCl_3$) δ 140.2, 139.8, 139.5, 132.0, 129.6, 129.0, 128.4, 128.2, 128.2, 126.9, 126.2, 125.5, 123.6, 122.9, 122.5, 120.4, 120.3, 118.7, 108.5, 108.1, 88.5, 85.7, 56.3, 54.6, 37.6, 13.8.

(1*R*)-2-(1,3-diphenylprop-2-yn-1-yl)-1-phenyl-1,2,3,4-tetrahydroisoquinoline (4bs)

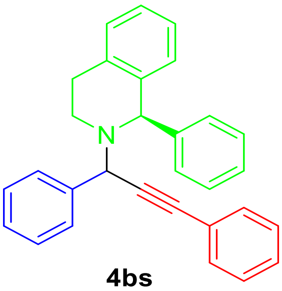

4bs

Prepared according to general procedure (B), **4bs** was purified by flash chromatography (PE-EA, v/v 10/1) as colorless oil with overall isolated yield: 90% (71.8 mg), dr 15.8:1. **^{1}H NMR** (600 MHz, $CDCl_3$) δ 7.68 (d, *J* = 7.2 Hz, 2H), 7.63 – 7.56 (m, 4H), 7.44 – 7.29 (m, 9H), 7.13 (q, *J* = 7.3 Hz, 2H), 7.07 – 7.02 (m,, 1H), 6.77 (d, *J* = 7.7 Hz, 1H), 5.16 (s, 1H), 4.94 (s, 1H), 3.20 – 3.09 (m, 1H), 2.91 (d, *J* = 11.5 Hz, 2H), 2.75 (d, *J* = 15.7 Hz, 1H).**^{13}C NMR** (151 MHz, $CDCl_3$) δ 144.1, 139.4, 139.2, 134.9, 131.9, 129.8, 128.9, 128.6, 128.3, 128.3, 128.2, 128.0, 127.6, 127.3, 125.7, 125.6, 123.2, 88.6, 84.6, 67.5, 56.8, 43.3, 30.0.

Compound Characterization of Redox-A^3 Coupling Reaction

2-benzyl-1-(phenylethynyl)-1,2,3,4-tetrahydroisoquinoline (5a)

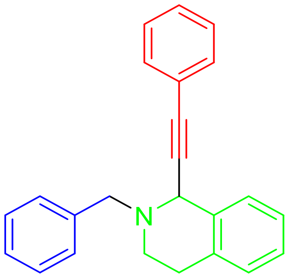

5a

Prepared according to general procedure (E), **5a** was purified by flash chromatography (PE-EA, v/v 10/1) as pale-yellow oil with overall isolated yield: 98% (63.3 mg), regioselectivity for 0:1 (4/5). **^{1}H NMR** (600 MHz, $CDCl_3$) δ 7.51 – 7.46 (m, 4H), 7.36 (t, *J* = 7.4 Hz, 2H), 7.33 – 7.27 (m, 5H), 7.21 – 7.13 (m, 3H), 4.82 (s, 1H), 3.96 (q, *J* = 13.1 Hz, 2H), 3.15 – 3.01 (m, 2H), 2.87 – 2.80 (m, 2H). **^{13}C NMR** (151 MHz, $CDCl_3$) δ 138.4, 135.5, 134.1, 131.8, 129.2, 129.0, 128.3, 128.2, 128.0, 127.8, 127.1, 126.9, 125.8, 123.3, 87.6, 86.8, 59.6, 54.4, 45.8, 29.1.

2-(4-methoxybenzyl)-1-(phenylethynyl)-1,2,3,4-tetrahydroisoquinoline (5b)

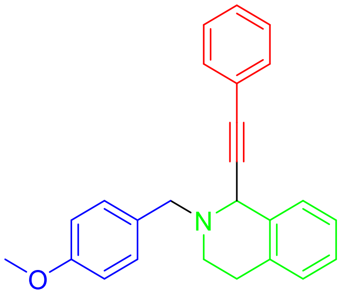

5b

Prepared according to general procedure (E), **5b** was purified by flash chromatography (PE-EA, v/v 10/1) as colorless oil with overall isolated yield: 99% (69.8 mg), regioselectivity for 1:2.6 (4/5). **^{1}H NMR** (600 MHz, $CDCl_3$) δ 7.48 – 7.44 (m, 2H), 7.39 (d, *J* = 8.5 Hz, 2H), 7.32 – 7.29 (m, 3H), 7.27 (d, *J* = 6.9 Hz, 1H), 7.19 – 7.15 (m, 2H), 7.13 (d, *J* = 7.4 Hz, 1H), 6.89 (d, *J* = 8.6 Hz, 2H), 4.78 (s, 1H), 3.88 (q, *J* = 13.0 Hz, 2H), 3.82 (s, 3H), 3.11 – 3.00 (m, 2H), 2.86 – 2.78 (m, 2H). **^{13}C NMR** (151 MHz, $CDCl_3$) δ 158.9, 135.6, 134.1, 131.8, 130.4, 129.0, 128.2, 128.0, 127.8, 126.9, 125.8, 123.3, 113.7, 87.6, 86.8, 58.9, 55.3, 54.2, 45.7, 29.1.

2-(2-methoxybenzyl)-1-(phenylethynyl)-1,2,3,4-tetrahydroisoquinoline (5c)

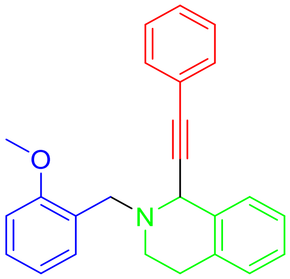

5c

Prepared according to general procedure (E), **5c** was purified by flash chromatography (PE-EA, v/v 10/1) as yellow oil with overall isolated yield: 96% (67.8 mg), regioselectivity for 0:1 (4/5). **^{1}H NMR** (600 MHz, $CDCl_3$) δ 7.57 (dd, *J* = 7.4, 1.3 Hz, 1H), 7.51 – 7.46 (m, 2H), 7.36 – 7.26 (m, 5H), 7.22 – 7.17 (m, 2H), 7.17 – 7.13 (m, 1H), 6.99 (td, *J* = 7.4, 0.9 Hz, 1H), 6.92 (d, *J* = 8.2 Hz, 1H), 4.92 (s, 1H), 4.02 (q, *J* = 14.1 Hz, 2H), 3.85 (s, 3H), 3.21 – 3.13 (m, 1H), 3.09 – 3.01 (m, 1H), 2.91 – 2.81 (m, 2H). **^{13}C NMR** (151 MHz, $CDCl_3$) δ 158.0, 135.8, 134.2, 131.8, 130.1, 128.9, 128.1, 128.0, 127.9, 127.8, 126.8, 125.7, 123.4, 120.4, 110.6, 88.2, 86.5, 55.5, 55.0, 53.0, 45.9, 29.1.

2-(2-fluorobenzyl)-1-(phenylethynyl)-1,2,3,4-tetrahydroisoquinoline (5d)

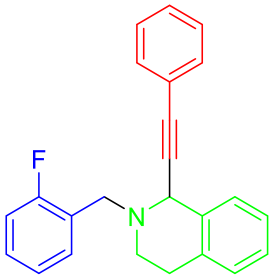

5d

Prepared according to general procedure (E), **5d** was purified by flash chromatography (PE-EA, v/v 10/1) as pale-yellow oil with overall isolated yield: 98% (66.8 mg), regioselectivity for 0:1 (4/5). **^{1}H NMR** (600 MHz, $CDCl_3$) δ 7.56 (t, *J* = 7.0 Hz, 1H), 7.47 – 7.42 (m, 2H), 7.32 – 7.22 (m, 5H), 7.19 – 7.14 (m, 2H), 7.12 (dd, *J* = 10.5, 4.1 Hz, 2H), 7.05 (t, *J* = 9.1 Hz, 1H), 4.84 (s, 1H), 4.00 (q, *J* = 13.7 Hz, 2H), 3.16 – 3.08 (m, 1H), 3.00 (dt, *J* = 7.0, 5.6 Hz, 1H), 2.84 – 2.78 (m, 2H). **^{13}C NMR** (151 MHz, $CDCl_3$) δ 161.6 (d, *J* = 246.8 Hz), 135.4, 134.0, 131.8, 131.3 (d, *J* = 4.4 Hz), 129.0, 128.7 (d, *J* = 8.2 Hz), 128.2, 128.0, 127.8, 126.9, 125.8, 125.3 (d, *J* = 14.1 Hz), 123.9 (d, *J* = 3.5 Hz), 123.2, 115.3 (d, *J* = 22.0 Hz), 87.5, 86.8, 54.7, 52.2 (d, *J* = 2.3 Hz), 45.9, 29.1. **^{19}F NMR** (565 MHz, $CDCl_3$) δ -117.74.

1-(phenylethynyl)-2-(thiophen-2-ylmethyl)-1,2,3,4-tetrahydroisoquinoline (5e)

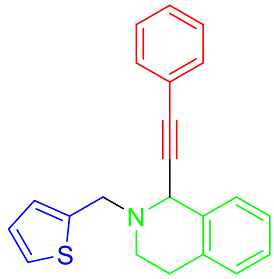

5e

Prepared according to general procedure (E), **5e** was purified by flash chromatography (PE-EA, v/v 10/1) as pale-yellow oil with overall isolated yield: 80% (52.6 mg), regioselectivity for 0:1 (4/5). **^{1}H NMR** (600 MHz, $CDCl_3$) δ 7.46 (dd, *J* = 6.6, 3.0 Hz, 2H), 7.32 – 7.27 (m, 5H), 7.22 – 7.10 (m, 4H), 7.00 (dd, *J* = 4.9, 3.6 Hz, 1H), 4.93 (s, 1H), 4.18 (dd, *J* = 34.6, 13.7 Hz, 2H), 3.11 (s, 2H), 2.99 – 2.80 (m, 2H). **^{13}C NMR** (151 MHz, $CDCl_3$) δ 141.2, 134.9, 133.8, 131.8, 129.0, 128.2, 128.2, 127.8, 127.1, 126.6, 125.9, 125.4, 123.0, 87.2, 86.8, 54.2, 53.9, 45.7, 28.9.

2-((1,9-dihydropyren-4-yl)methyl)-1-(phenylethynyl)-1,2,3,4-tetrahydroiso-quinoline (5f)

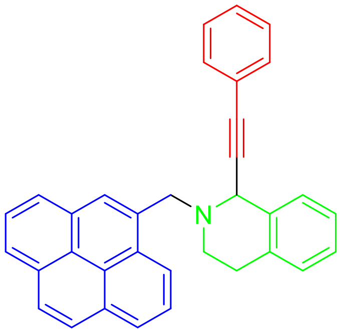

5f

Prepared according to general procedure (E), **5f** was purified by flash chromatography (PE-EA, v/v 5/1) as yellow solid with overall isolated yield: 97% (86.7 mg), regioselectivity for 0:1 (4/5). **^{1}H NMR** (600 MHz, $CDCl_3$) δ 8.70 (d, *J* = 9.2 Hz, 1H), 8.21 – 8.15 (m, 4H), 8.10 – 8.06 (m, 3H), 8.00 (t, *J* = 7.6 Hz, 1H), 7.57 (dd, *J* = 7.5, 2.0 Hz, 2H), 7.39 – 7.34 (m, 3H), 7.26 (dd, *J* = 4.8, 2.8 Hz, 1H), 7.20 – 7.12 (m, 3H), 4.88 (s, 1H), 4.74 (d, *J* = 13.0 Hz, 1H), 4.54 (d, *J* = 13.0 Hz, 1H), 3.23 (td, *J* = 10.9, 4.3 Hz, 1H), 3.08 – 3.00 (m, 1H), 3.00 – 2.95 (m, 1H), 2.81 (dd, *J* = 16.2, 3.5 Hz, 1H). **^{13}C NMR** (151 MHz, $CDCl_3$) δ 135.6, 134.2, 131.9, 131.8, 131.3, 130.9, 130.9, 130.3, 128.9, 128.4, 128.3, 128.1, 127.8, 127.4, 127.2, 127.2, 126.9, 125.8, 125.8, 125.1, 125.0, 124.9, 124.8, 124.4, 124.4, 123.4, 87.9, 87.0, 57.9, 54.7, 45.9, 29.2.

2-(cyclohexylmethyl)-1-(phenylethynyl)-1,2,3,4-tetrahydroisoquinoline (5g)

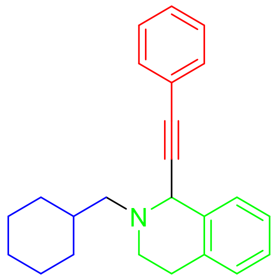

5g

Prepared according to general procedure (E), **5g** was purified by flash chromatography (PE-EA, v/v 10/1) as yellow oil with overall isolated yield: 87% (57.2 mg), regioselectivity for 0:1 (4/5). ^{1}**H NMR** (600 MHz, $CDCl_3$) δ 7.48 – 7.43 (m, 2H), 7.33 – 7.28 (m, 3H), 7.18 – 7.12 (m, 3H), 7.12 – 7.08 (m, 1H), 3.95 (d, *J* = 14.8 Hz, 1H), 3.78 (d, *J* = 14.8 Hz, 1H), 3.41 (d, *J* = 10.1 Hz, 1H), 3.02 (dq, *J* = 10.5, 5.3 Hz, 1H), 2.99 – 2.92 (m, 2H), 2.76 (dt, *J* = 11.3, 5.8 Hz, 1H), 2.23 (d, *J* = 12.8 Hz, 1H), 2.14 (d, *J* = 13.4 Hz, 1H), 1.79 (dddd, *J* = 19.1, 12.7, 8.1, 3.4 Hz, 4H), 1.41 – 1.33 (m, 1H), 1.32 – 1.22 (m, 2H), 1.13 (qd, *J* = 12.6, 3.5 Hz, 1H), 1.06 – 0.97 (m, 1H). ^{13}**C NMR** (151 MHz, $CDCl_3$) δ 135.6, 134.7, 131.7, 128.6, 128.2, 127.7, 126.7, 125.8, 125.4, 123.5, 86.9, 86.6, 63.7, 52.4, 47.2, 39.8, 31.3, 30.4, 29.7, 26.7, 26.2, 26.0.

2-benzyl-1-((4-methoxyphenyl) ethynyl)-1,2,3,4-tetrahydroisoquinoline (5h)

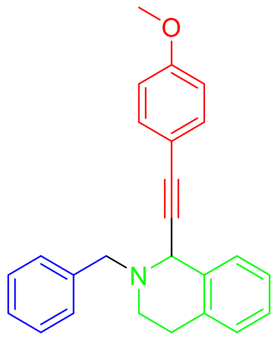

5h

Prepared according to general procedure (E), **5h** was purified by flash chromatography (PE-EA, v/v 10/1) as white solid with overall isolated yield: 99% (69.7 mg), regioselectivity for 1:11.5 (4/5). ^{1}**H NMR** (600 MHz, $CDCl_3$) δ 7.50 (d, *J* = 7.2 Hz, 2H), 7.41 (d, *J* = 8.8 Hz, 2H), 7.36 (t, *J* = 7.5 Hz, 2H), 7.32 – 7.28 (m, 2H), 7.18 (td, *J* = 6.5, 1.6 Hz, 2H), 7.16 – 7.13 (m, 1H), 6.85 (d, *J* = 8.8 Hz, 2H), 4.80 (s, 1H), 3.95 (q, *J* = 13.1 Hz, 2H), 3.82 (s, 3H), 3.14 – 3.01 (m, 2H), 2.86 – 2.80 (m, 2H). ^{13}**C NMR** (151 MHz, $CDCl_3$) δ 159.4, 138.5, 135.7, 134.0, 133.2, 129.2, 129.0, 128.3, 127.8, 127.1, 126.8, 125.8, 115.4, 113.8, 86.6, 86.0, 59.6, 55.3, 54.5, 45.7, 29.1.

2-benzyl-1-(m-tolylethynyl)-1,2,3,4-tetrahydroisoquinoline (5i)

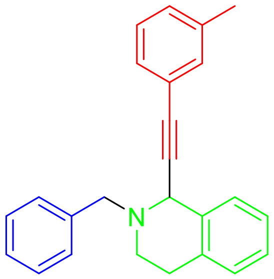

5i

Prepared according to general procedure (E), **5i** was purified by flash chromatography (PE-EA, v/v 10/1) as colorless oil with overall isolated yield: 99% (66.7 mg), regioselectivity for 1:9 (4/5). **^{1}H NMR** (600 MHz, $CDCl_3$) δ 7.50 (d, J = 7.1 Hz, 2H), 7.37 (t, J = 7.4 Hz, 2H), 7.32 – 7.28 (m, 4H), 7.23 – 7.17 (m, 3H), 7.14 (dd, J = 12.0, 7.5 Hz, 2H), 4.82 (s, 1H), 3.96 (q, J = 13.1 Hz, 2H), 3.15 – 3.02 (m, 2H), 2.88 – 2.79 (m, 2H), 2.35 (s, 3H). **^{13}C NMR** (151 MHz, $CDCl_3$) δ 138.4, 137.9, 135.6, 134.1, 132.3, 129.2, 129.0, 128.9, 128.8, 128.3, 128.1, 127.8, 127.1, 126.9, 125.8, 123.1, 87.2, 87.0, 59.6, 54.4, 45.7, 29.1, 21.2.

2-benzyl-1-((3-bromophenyl) ethynyl)-1,2,3,4-tetrahydroisoquinoline (5j)

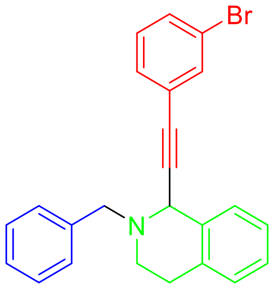

5j

Prepared according to general procedure (E), **5j** was purified by flash chromatography (PE-EA, v/v 10/1) as pale-yellow oil with overall isolated yield: 96% (77.0 mg), regioselectivity for 0:1 (4/5). **^{1}H NMR** (600 MHz, $CDCl_3$) δ 7.58 (s, 1H), 7.45 (d, J = 7.2 Hz, 2H), 7.42 – 7.40 (m, 1H), 7.37 – 7.32 (m, 3H), 7.28 (t, J = 7.3 Hz, 1H), 7.24 – 7.21 (m, 1H), 7.18 – 7.11 (m, 4H), 4.78 (s, 1H), 3.90 (q, J = 13.2 Hz, 2H), 3.08 – 2.98 (m, 2H), 2.86 – 2.76 (m, 2H). **^{13}C NMR** (151 MHz, $CDCl_3$) δ 138.3, 135.2, 134.5, 134.1, 131.2, 130.3, 129.6, 129.2, 129.1, 128.3, 127.7, 127.2, 127.0, 125.9, 125.2, 122.0, 89.1, 85.4, 59.6, 54.3, 45.8, 29.0.

2-benzyl-1-((2-fluorophenyl) ethynyl)-1,2,3,4-tetrahydroisoquinoline (5k)

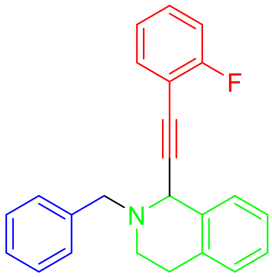

5k

Prepared according to general procedure (E), **5k** was purified by flash chromatography (PE-EA, v/v 10/1) as colorless oil with overall isolated yield: 85% (58.0 mg), regioselectivity for 0:1 (4/5). **^{1}H NMR** (600 MHz, $CDCl_3$) δ 7.51 (d, *J* = 7.2 Hz, 2H), 7.45 – 7.41 (m, 1H), 7.37 (t, *J* = 7.4 Hz, 2H), 7.30 (dd, *J* = 15.0, 7.4 Hz, 3H), 7.21 – 7.14 (m, 3H), 7.10 – 7.06 (m, 2H), 4.84 (s, 1H), 3.97 (s, 2H), 3.15 – 3.04 (m, 2H), 2.90 – 2.79 (m, 2H). **^{13}C NMR** (151 MHz, $CDCl_3$) δ 162.9 (d, *J* = 251.1 Hz), 138.3, 135.2, 134.1, 133.6, 129.6 (d, *J* = 7.8 Hz), 129.3, 129.0, 128.4, 128.3, 127.8, 127.1 (d, *J* = 27.9 Hz), 125.8, 123.8 (d, *J* = 3.7 Hz), 115.4 (d, *J* = 21.0 Hz), 111.8 (d, *J* = 15.7 Hz), 93.0, 80.1, 59.5, 54.4, 45.8, 29.1. **^{19}F NMR** (565 MHz, $CDCl_3$) δ -109.70.

2-benzyl-1-(phenanthren-9-ylethynyl)-1,2,3,4-tetrahydroisoquinoline (5l)

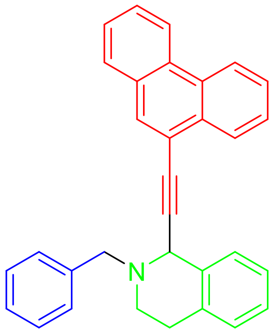

5l

Prepared according to general procedure (E), **5l** was purified by flash chromatography (PE-EA, v/v 10/1) as pale-yellow oil with overall isolated yield: 75% (63.4 mg), regioselectivity for 0:1 (4/5). **^{1}H NMR** (600 MHz, $CDCl_3$) δ 8.67 (dd, *J* = 21.3, 8.1 Hz, 2H), 8.44 (dd, *J* = 7.9, 1.1 Hz, 1H), 8.01 (s, 1H), 7.85 (d, *J* = 7.8 Hz, 1H), 7.69 (ddd, *J* = 19.9, 11.7, 4.4 Hz, 3H), 7.62 – 7.58 (m, 1H), 7.55 (d, *J* = 7.0 Hz, 2H), 7.42 – 7.37 (m, 3H), 7.33 (t, *J* = 7.3 Hz, 1H), 7.25 – 7.19 (m, 3H), 5.02 (s, 1H), 4.09 (s, 2H), 3.25 (td, *J* = 11.5, 4.1 Hz, 1H), 3.12 (ddd, *J* = 16.4, 10.2, 6.2 Hz, 1H), 2.99 – 2.92 (m, 1H), 2.89 (d, *J* = 16.0 Hz, 1H). **^{13}C NMR** (151 MHz, $CDCl_3$) δ 138.4, 135.6, 134.1, 131.9, 131.3, 131.2, 130.2, 130.1, 129.3, 129.1, 128.4, 128.4, 127.9, 127.3, 127.2, 127.0, 127.0, 127.0, 126.9, 125.9, 122.7, 122.6, 119.6, 92.3, 85.2, 59.8, 54.7, 46.0, 29.2.

2-benzyl-1-(thiophen-3-ylethynyl)-1,2,3,4-tetrahydroisoquinoline (5m)

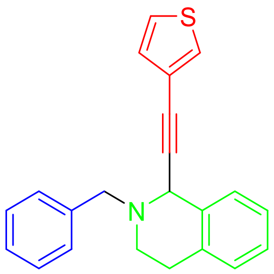

5m

Prepared according to general procedure (E), **5m** was purified by flash chromatography (PE-EA, v/v 10/1) as pale-yellow oil with overall isolated yield: 98% (64.5 mg), regioselectivity for 1:4.9 (4/5). **^{1}H NMR** (600 MHz, $CDCl_3$) δ 7.45 (d, *J* = 7.4 Hz, 2H), 7.43 – 7.39 (m, 1H), 7.33 (t, *J* = 7.5 Hz, 2H), 7.28 (d, *J* = 6.8 Hz, 1H), 7.25 – 7.22 (m, 2H), 7.15 (t, *J* = 7.6 Hz, 2H), 7.13 – 7.09 (m, 2H), 4.77 (s, 1H), 3.91 (q, *J* = 13.1 Hz, 2H), 3.10 – 2.97 (m, 2H), 2.79 (t, *J* = 11.9 Hz, 2H). **^{13}C NMR** (151 MHz, $CDCl_3$) δ 138.4, 135.5, 134.1, 130.1, 129.2, 129.0, 128.4, 128.3, 127.8, 127.1, 126.9, 125.8, 125.1, 122.2, 87.1, 81.8, 59.6, 54.5, 45.7, 29.0.

2-benzyl-1-(hex-1-yn-1-yl)-1,2,3,4-tetrahydroisoquinoline (5n)

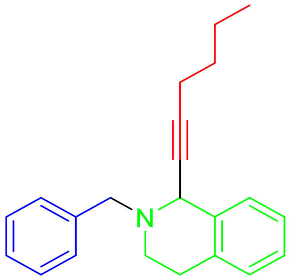

5n

Prepared according to general procedure (E), **5n** was purified by flash chromatography (PE-EA, v/v 10/1) as colorless oil with overall isolated yield: 90% (54.5 mg), regioselectivity for 0:1 (4/5). **^{1}H NMR** (600 MHz, $CDCl_3$) δ 7.45 (d, *J* = 7.3 Hz, 2H), 7.34 (t, *J* = 7.5 Hz, 2H), 7.30 – 7.28 (m, 1H), 7.23 – 7.20 (m, 1H), 7.15 (pd, *J* = 7.2, 3.6 Hz, 2H), 7.12 – 7.09 (m, 1H), 4.56 (s, 1H), 3.90 (d, *J* = 13.1 Hz, 1H), 3.82 (d, *J* = 13.1 Hz, 1H), 3.05 – 2.94 (m, 2H), 2.83 – 2.70 (m, 2H), 2.26 (td, *J* = 7.0, 2.0 Hz, 2H), 1.59 – 1.51 (m, 2H), 1.50 – 1.42 (m, 2H), 0.94 (t, *J* = 7.3 Hz, 3H). **^{13}C NMR** (151 MHz, $CDCl_3$) δ 138.6, 136.4, 133.9, 129.2, 128.9, 128.2, 127.7, 127.0, 126.6, 125.7, 87.1, 77.9, 59.5, 54.1, 45.6, 31.1, 29.1, 22.0, 18.5, 13.6.

^{1}H, ^{13}C and ^{19}F NMR Spectra of New Compounds

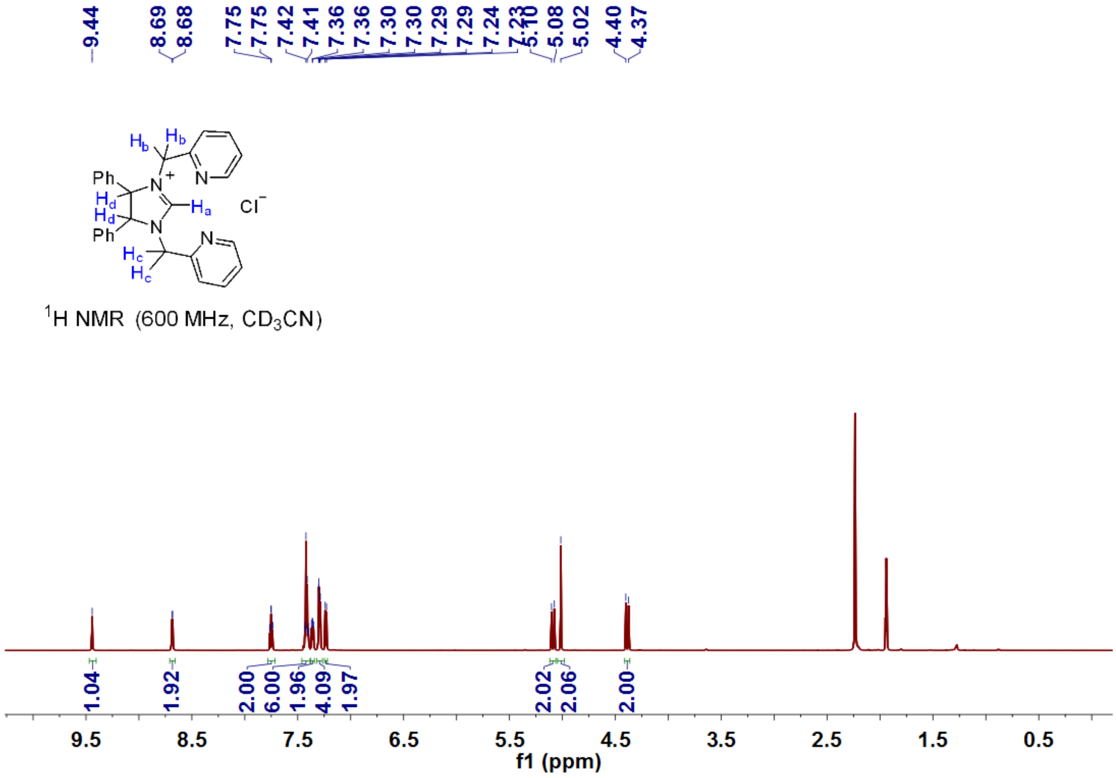

Supplementary Figure 44. ^{1}**H NMR** spectrum of **NHC** ligand.

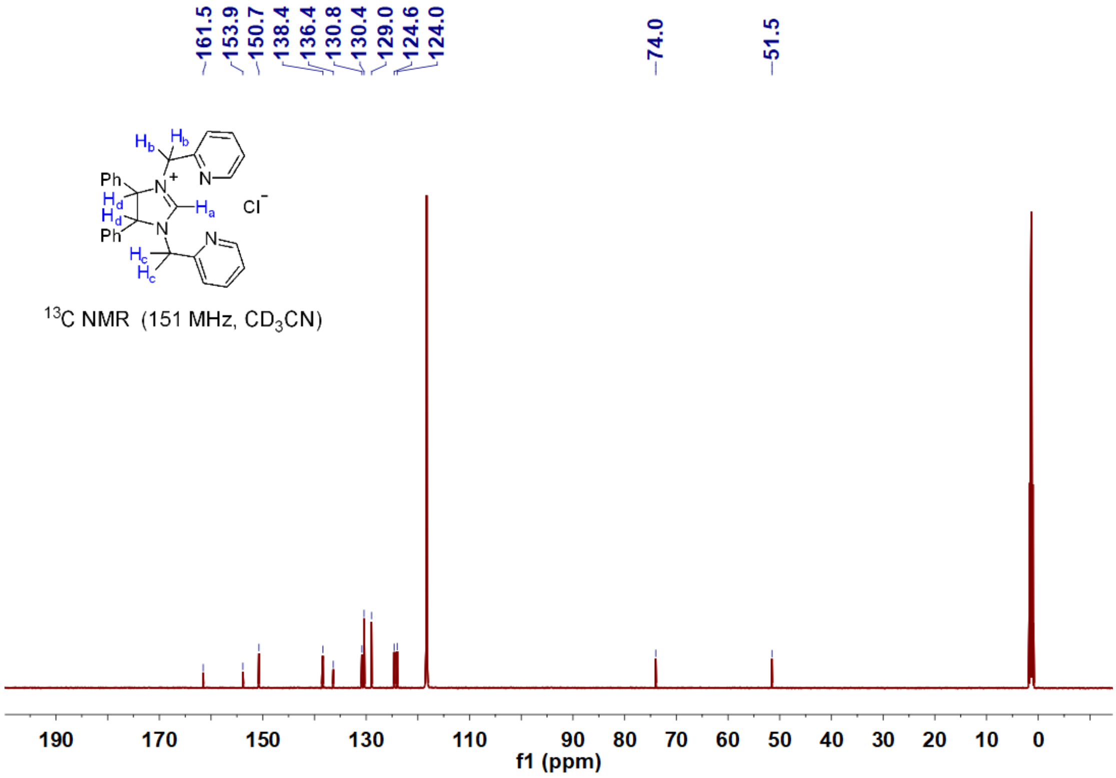

Supplementary Figure 45. ^{13}**C NMR** spectrum of **NHC** ligand.

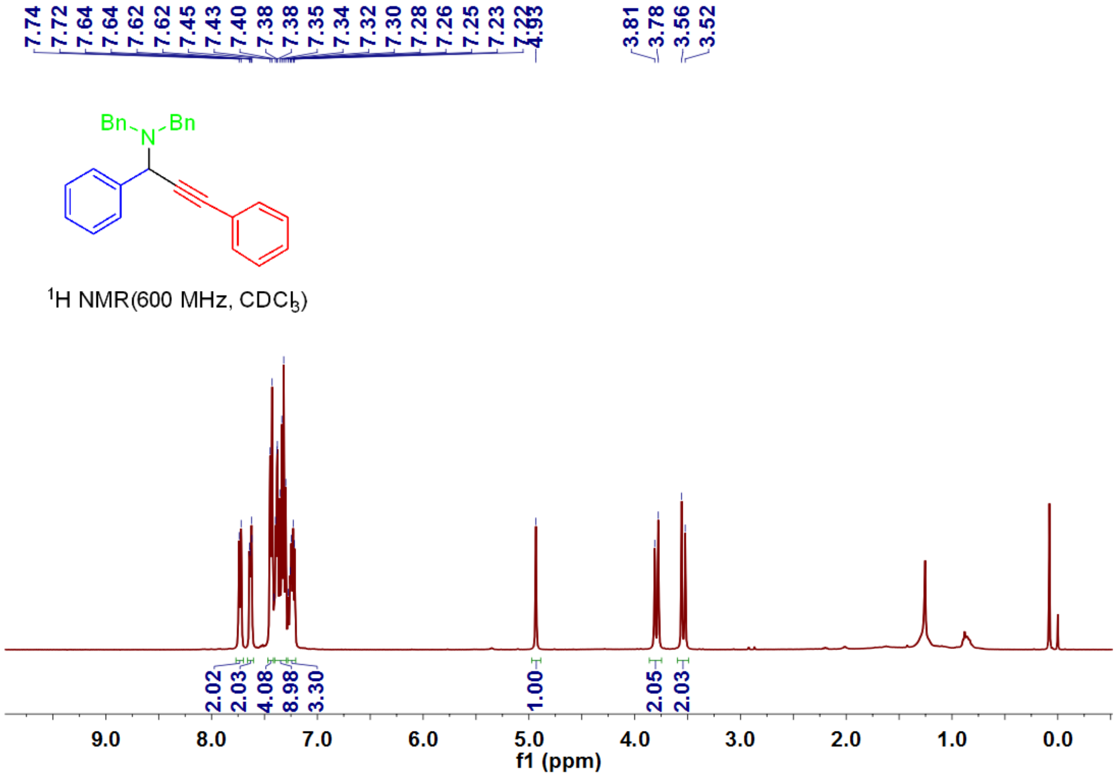

Supplementary Figure 46. ^{1}**H NMR** spectrum of compound **4a**.

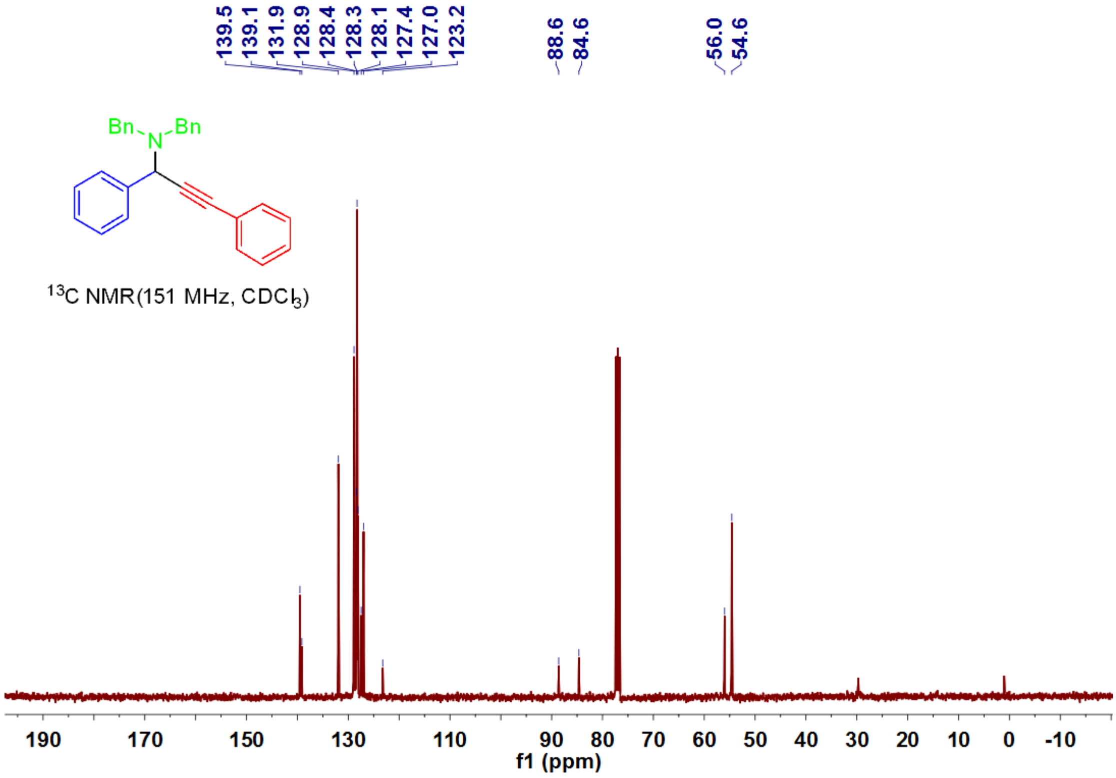

Supplementary Figure 47. ^{13}**C NMR** spectrum of compound **4a**.

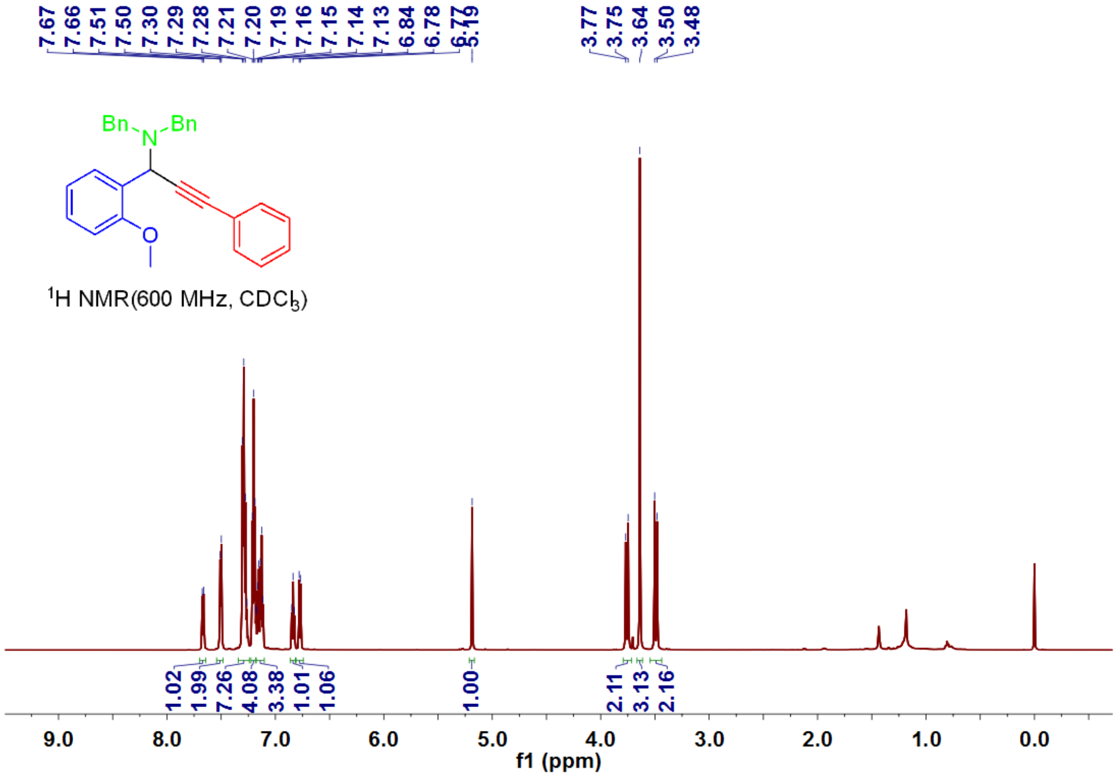

Supplementary Figure 48. ^{1}**H NMR** spectrum of compound **4b**.

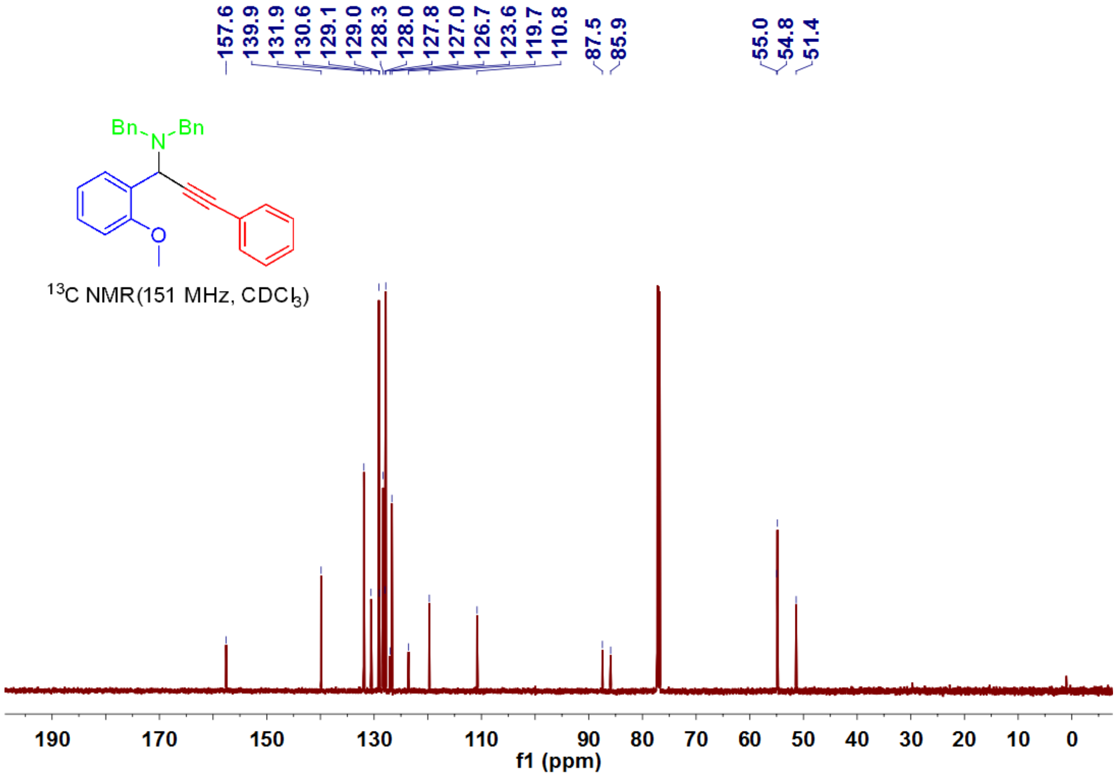

Supplementary Figure 49. ^{13}**C NMR** spectrum of compound **4b**.

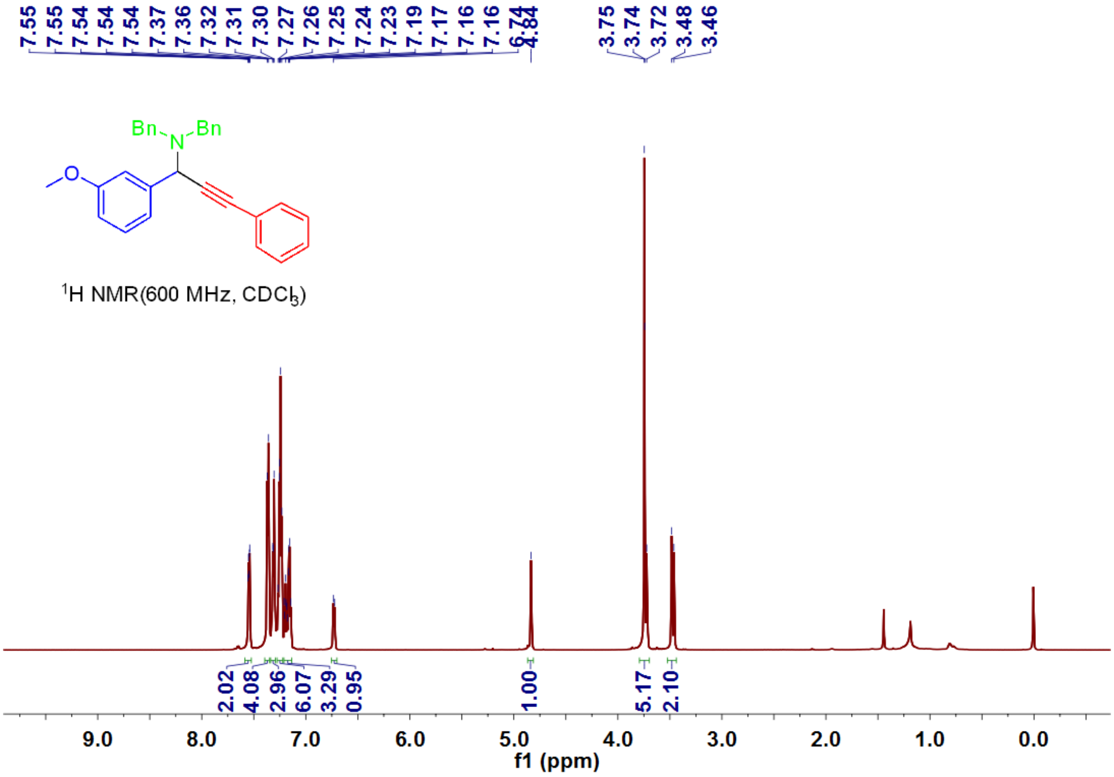

Supplementary Figure 50. ^{1}**H NMR** spectrum of compound **4c**.

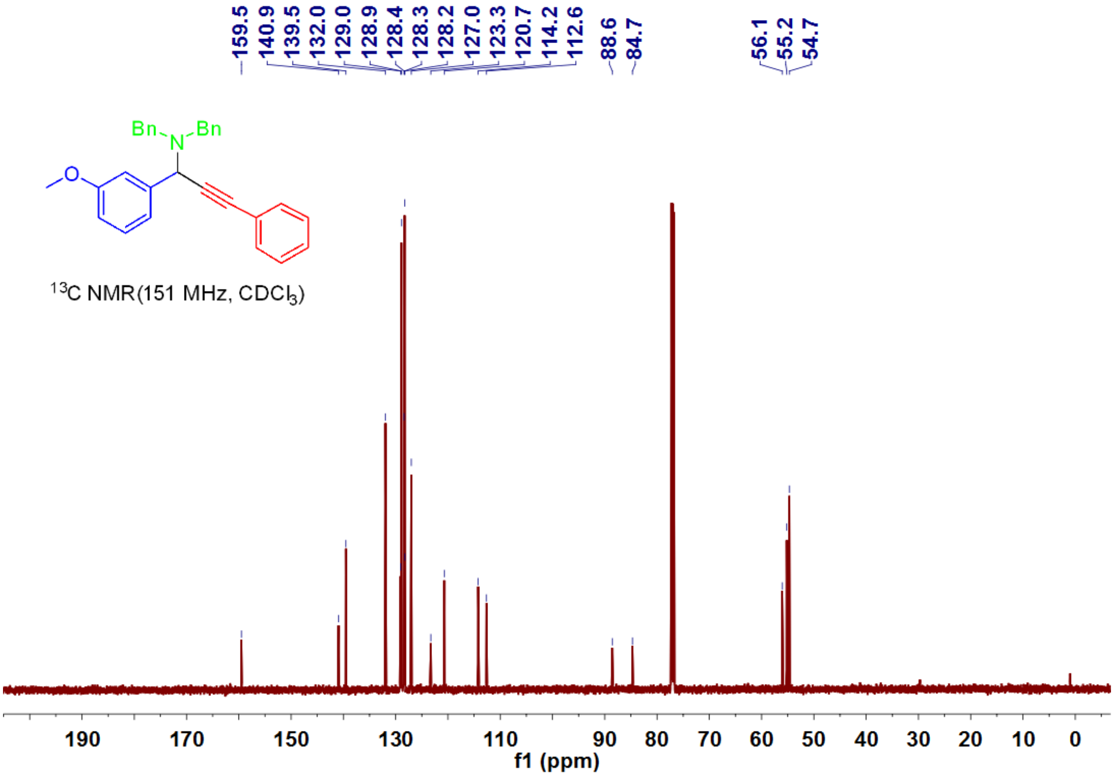

Supplementary Figure 51. ^{13}**C NMR** spectrum of compound **4c**.

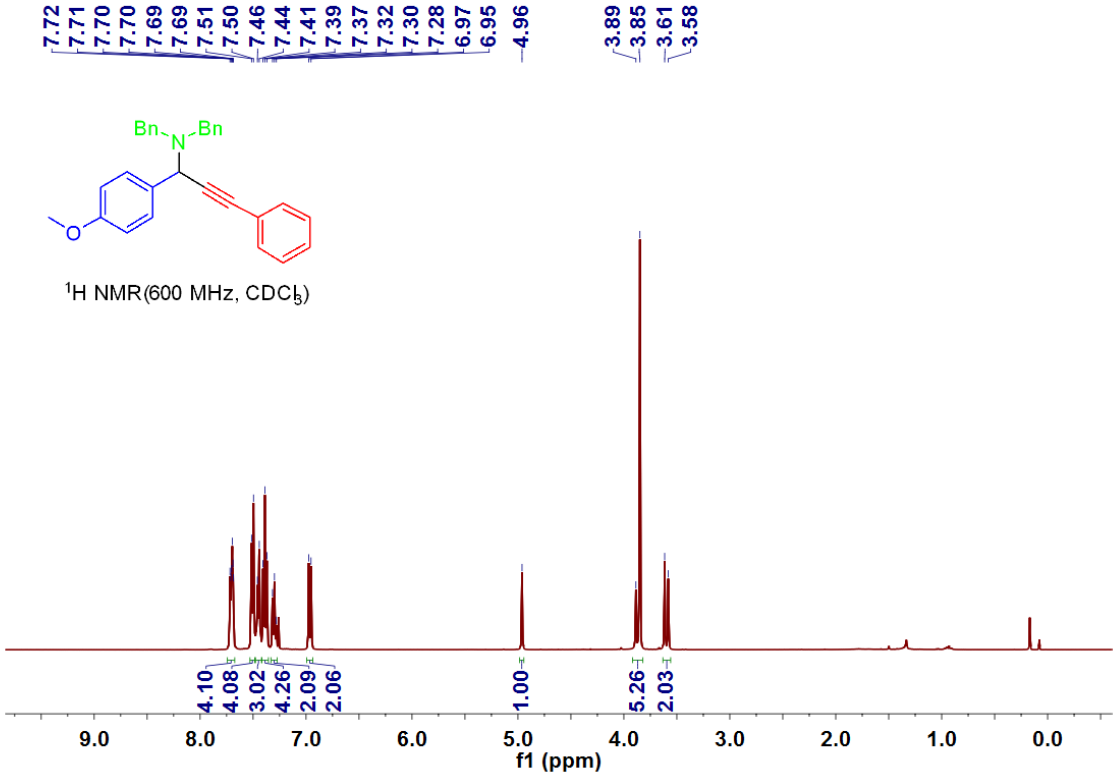

Supplementary Figure 52. ^{1}**HMR** spectrum of compound **4d.**

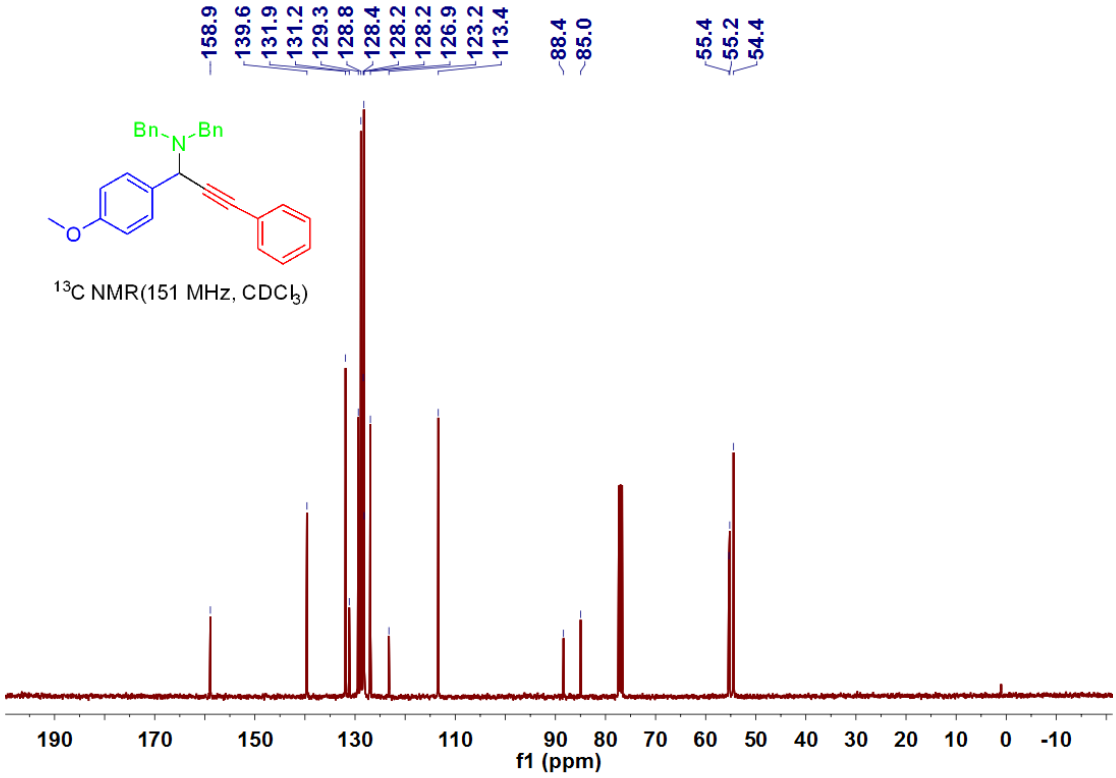

Supplementary Figure 53. ^{13}**C NMR** spectrum of compound **4d**.

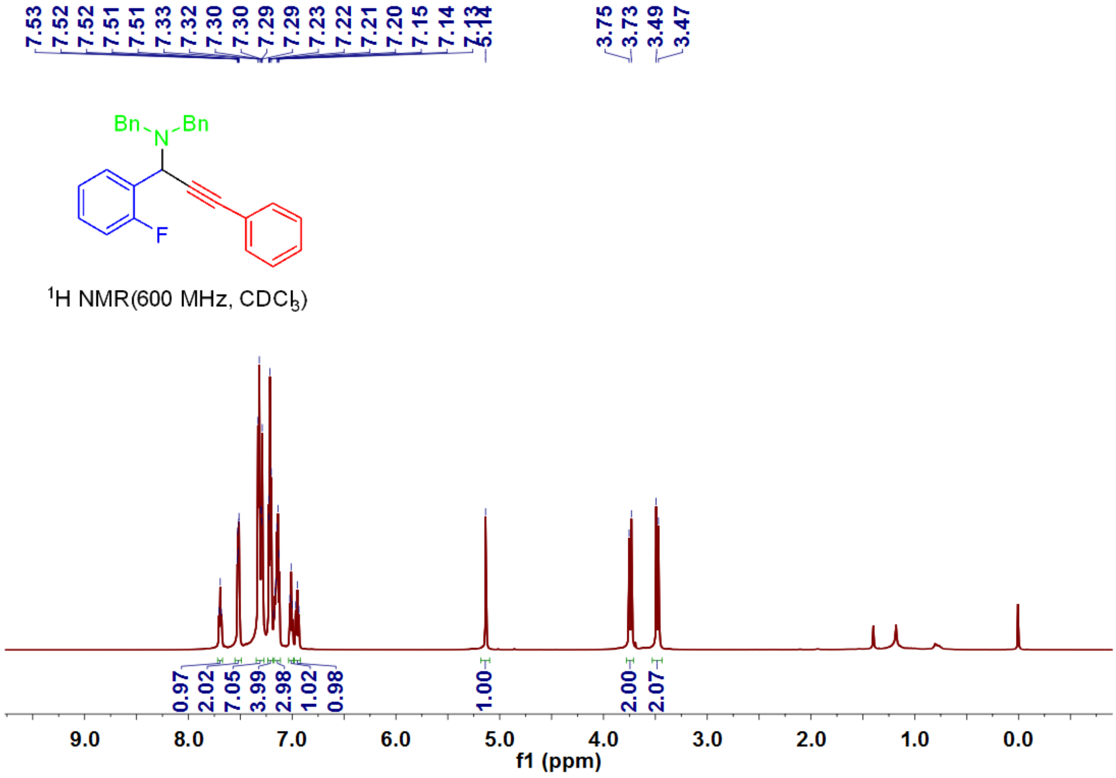

Supplementary Figure 54. **^{1}H NMR** spectrum of compound **4e**.

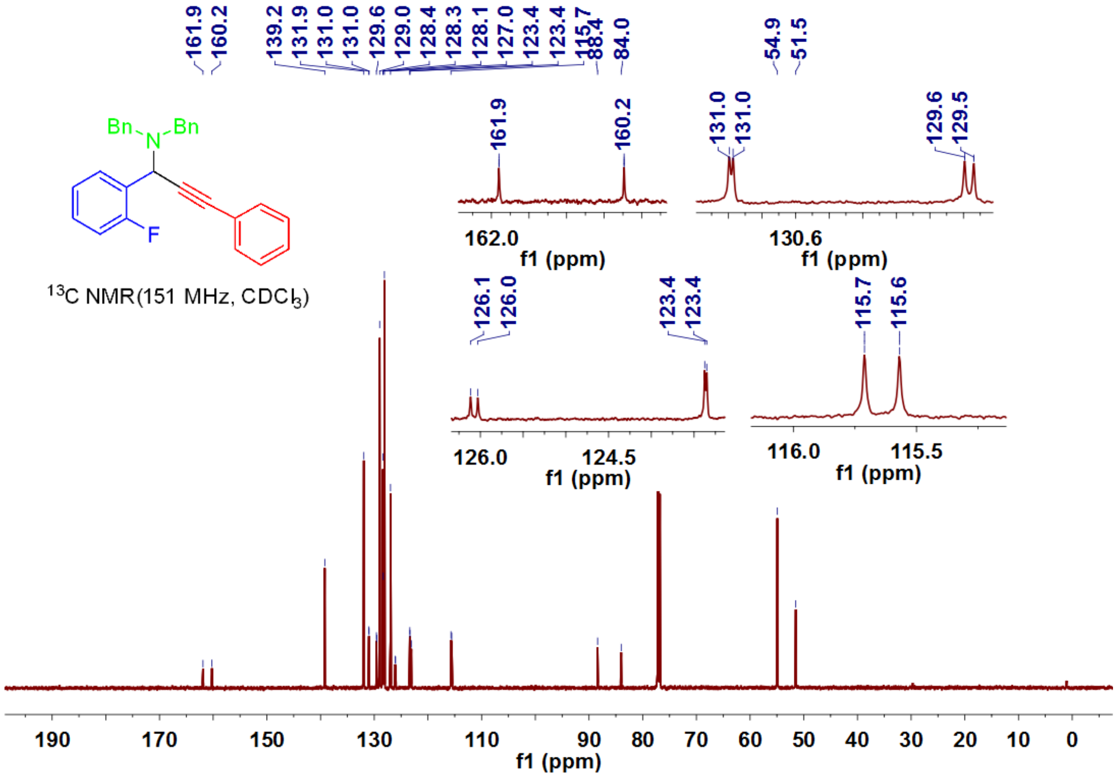

Supplementary Figure 55. **^{13}C NMR** spectrum of compound **4e**.

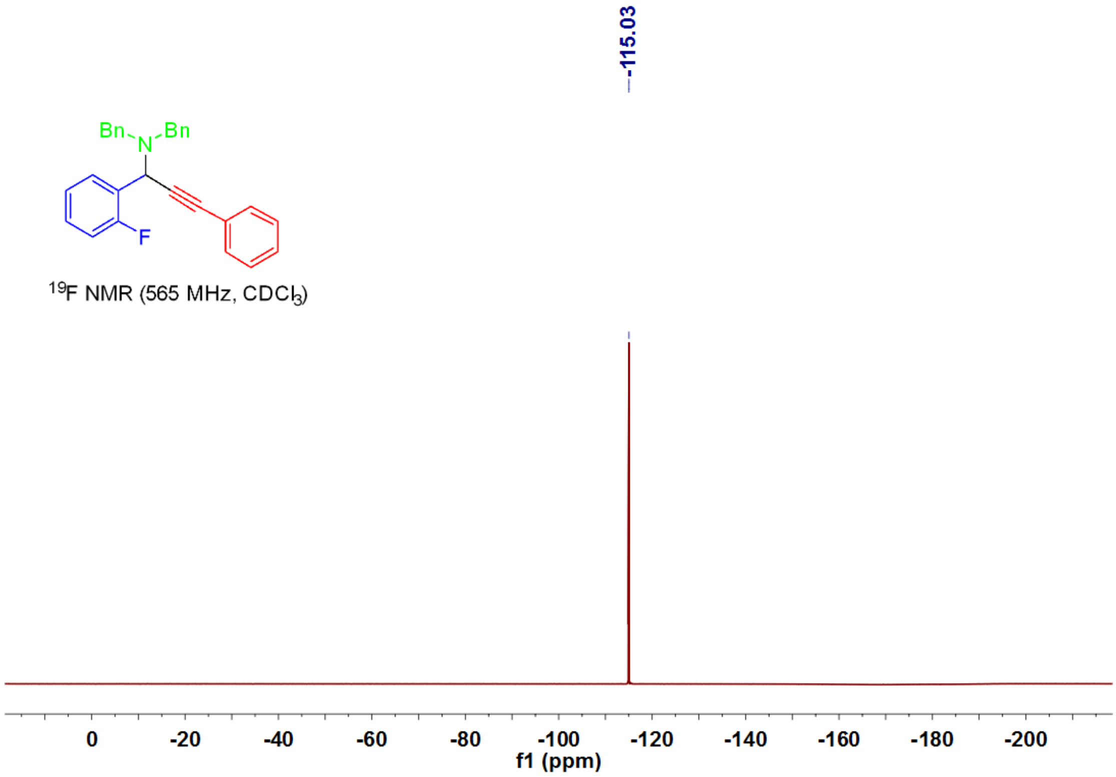

Supplementary Figure 56. ^{19}**F NMR** spectrum of compound **4e**.

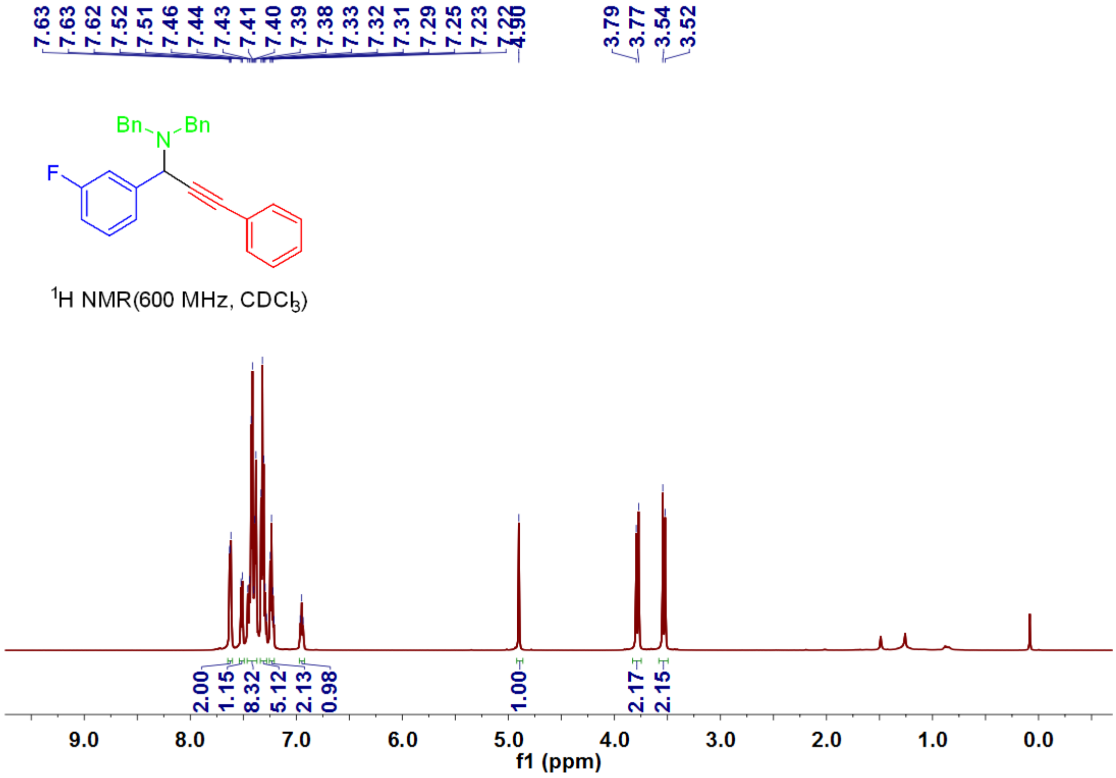

Supplementary Figure 57. ^{1}**H NMR** spectrum of compound **4f**.

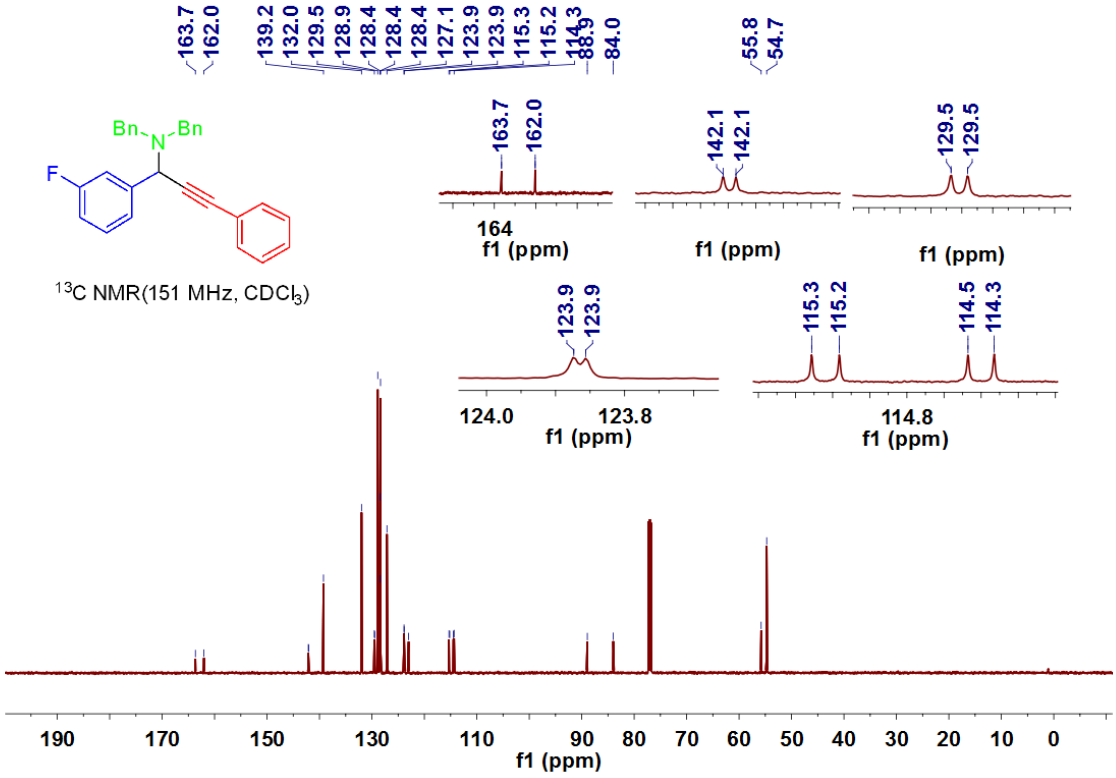

Supplementary Figure 58. ^{13}**C NMR** spectrum of compound **4f**.

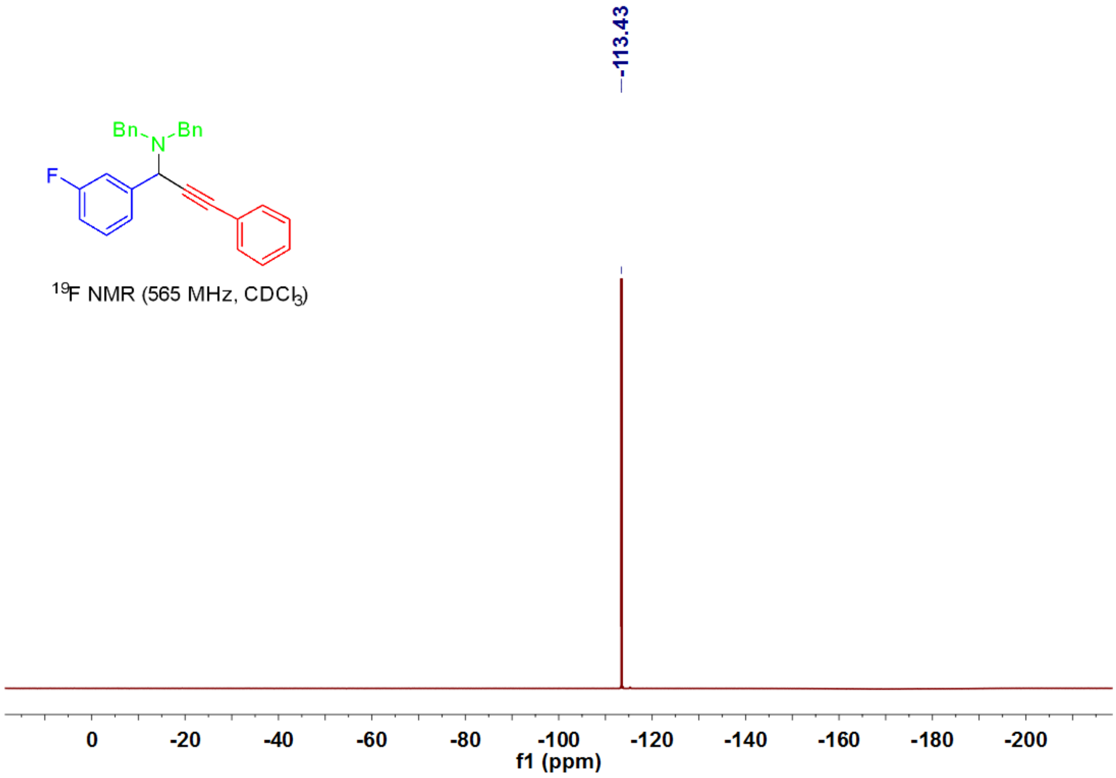

Supplementary Figure 59. ^{19}**F NMR** spectrum of compound **4f**.

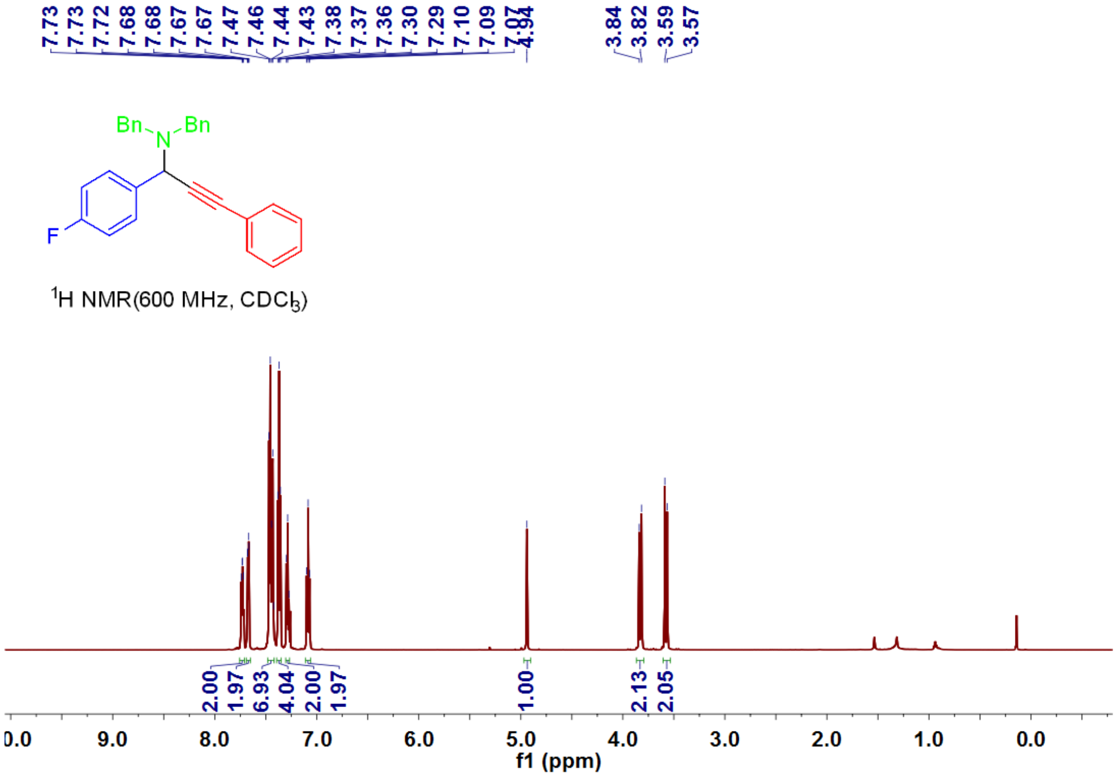

Supplementary Figure 60. **^{1}H NMR** spectrum of compound **4g**.

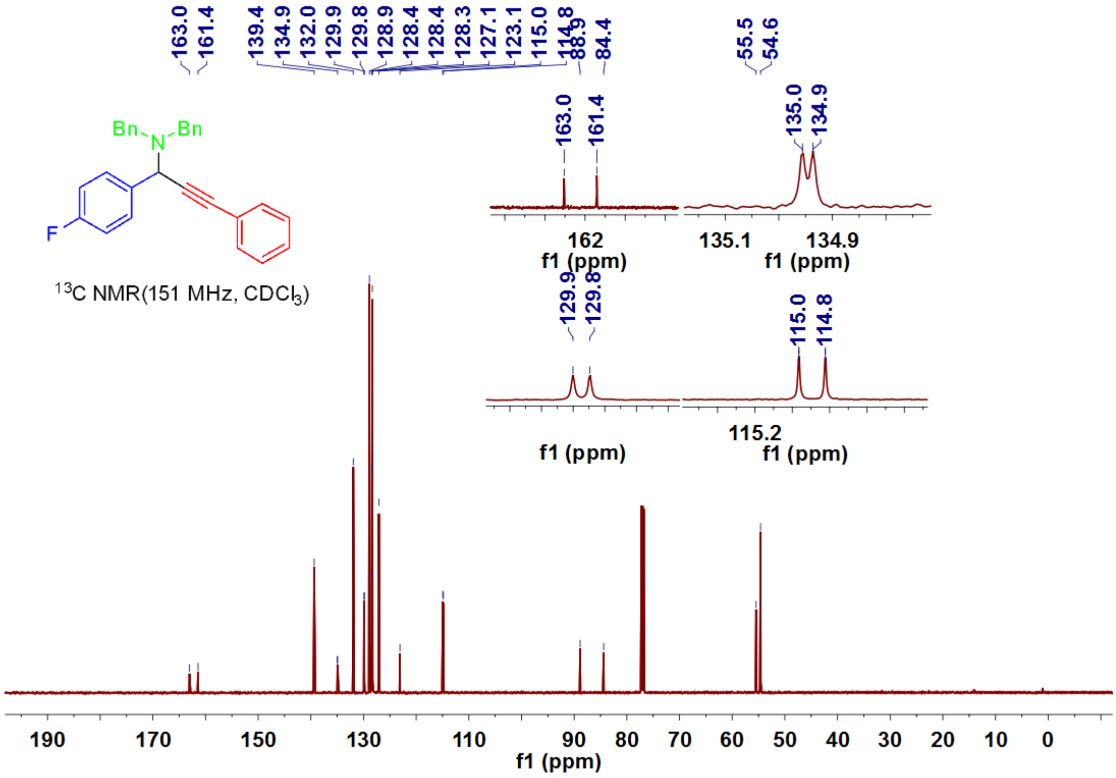

Supplementary Figure 61. **^{13}C NMR** spectrum of compound **4g**.

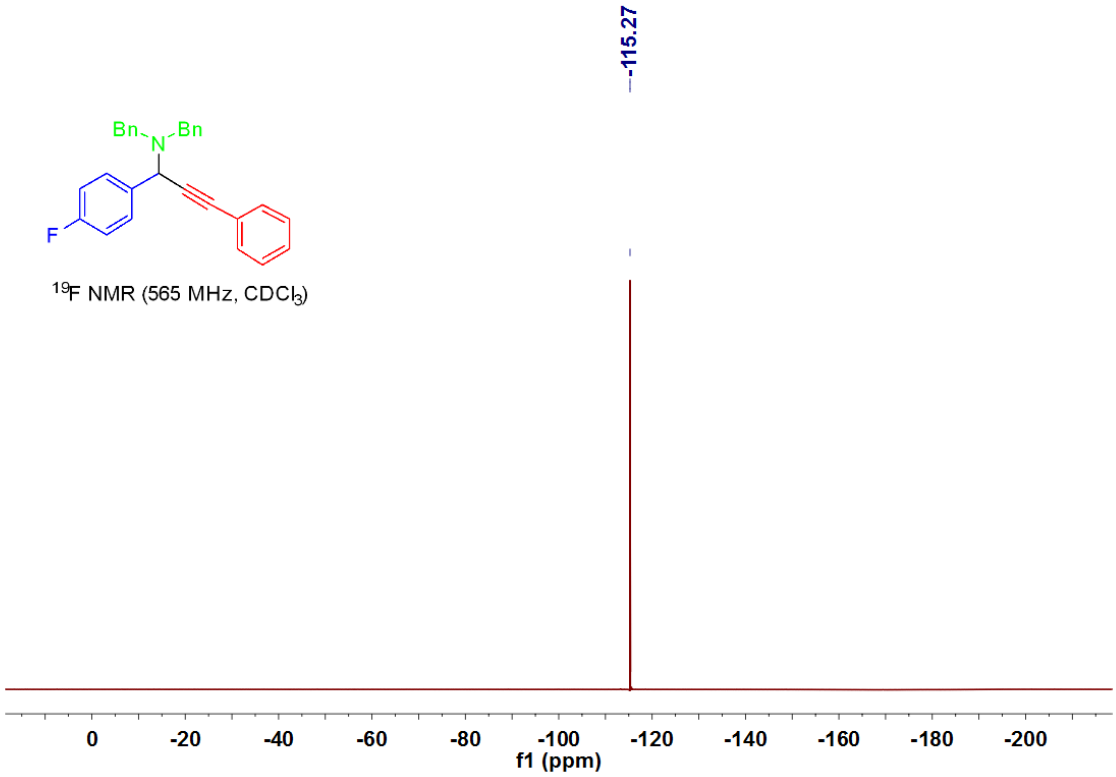

Supplementary Figure 62. **^{19}F NMR** spectrum of compound **4g**.

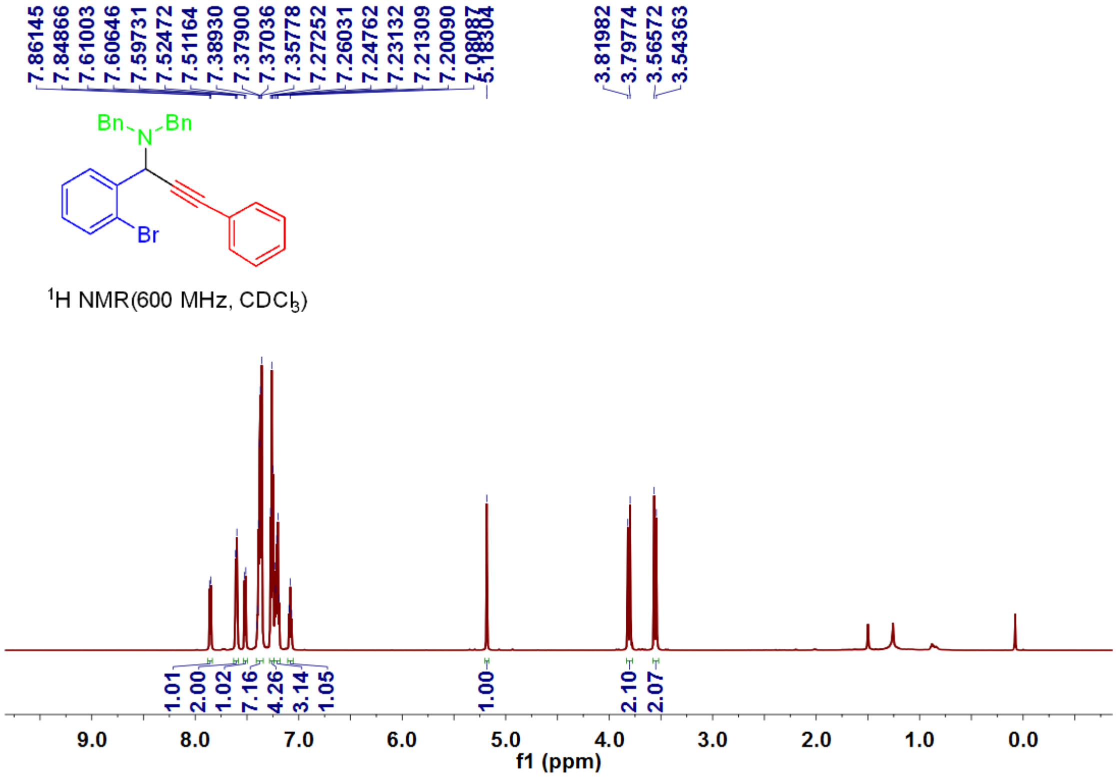

Supplementary Figure 63. **^{1}H NMR** spectrum of compound **4h**.

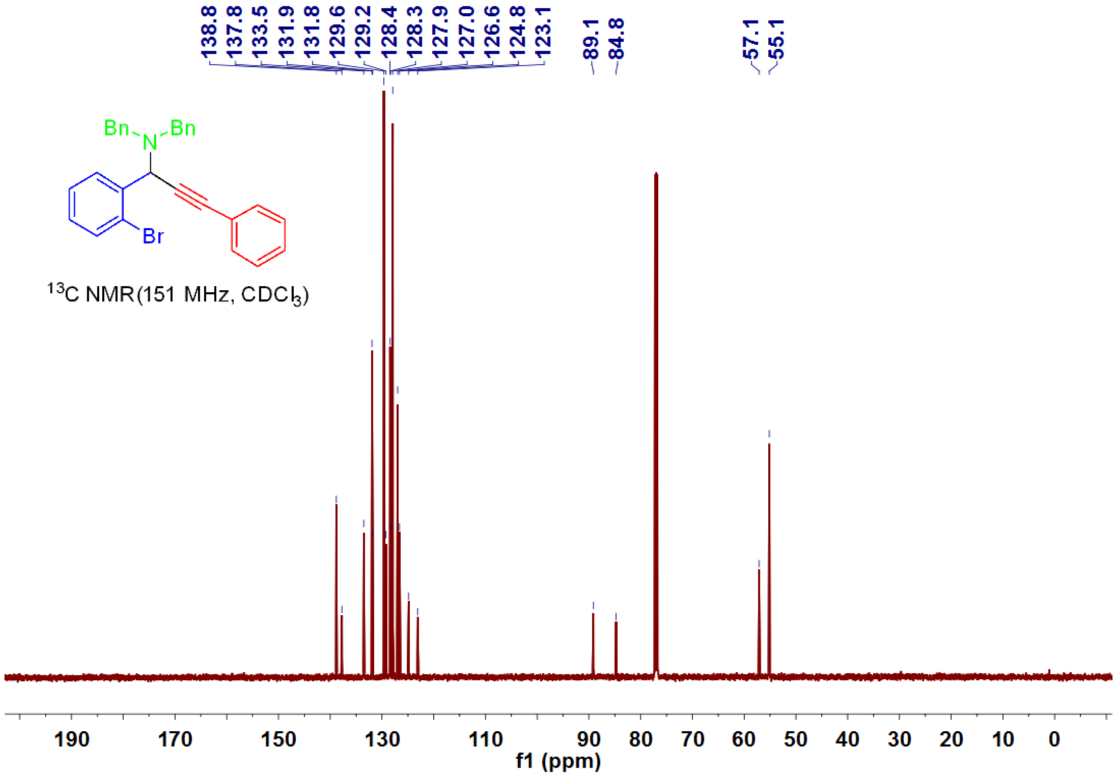

Supplementary Figure 64. ^{13}C NMR spectrum of compound **4h**.

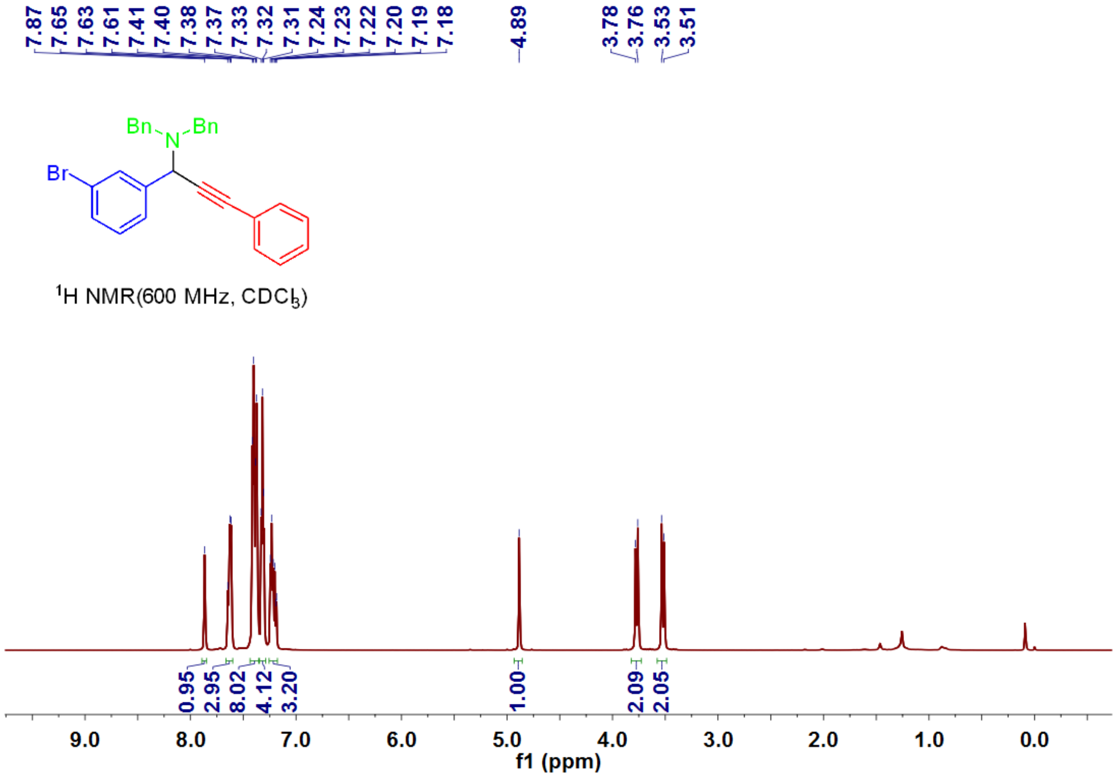

Supplementary Figure 65. ^{1}H NMR spectrum of compound **4i**.

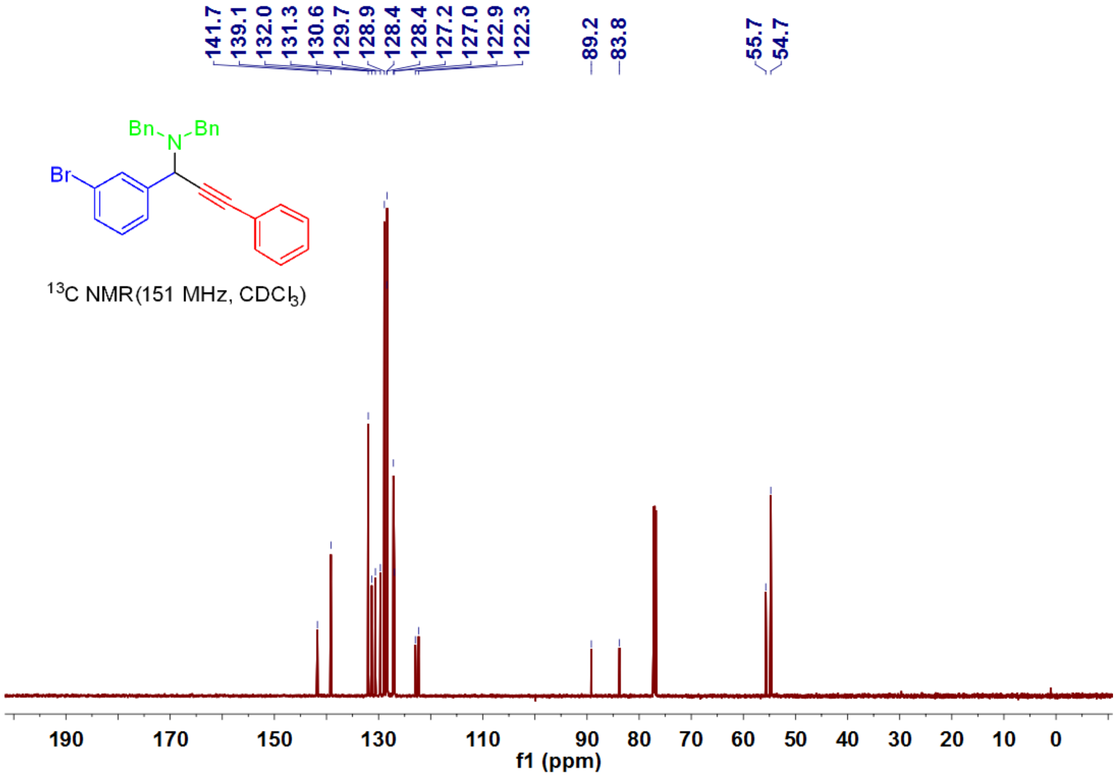

Supplementary Figure 66. ^{13}**C NMR** spectrum of compound **4i**.

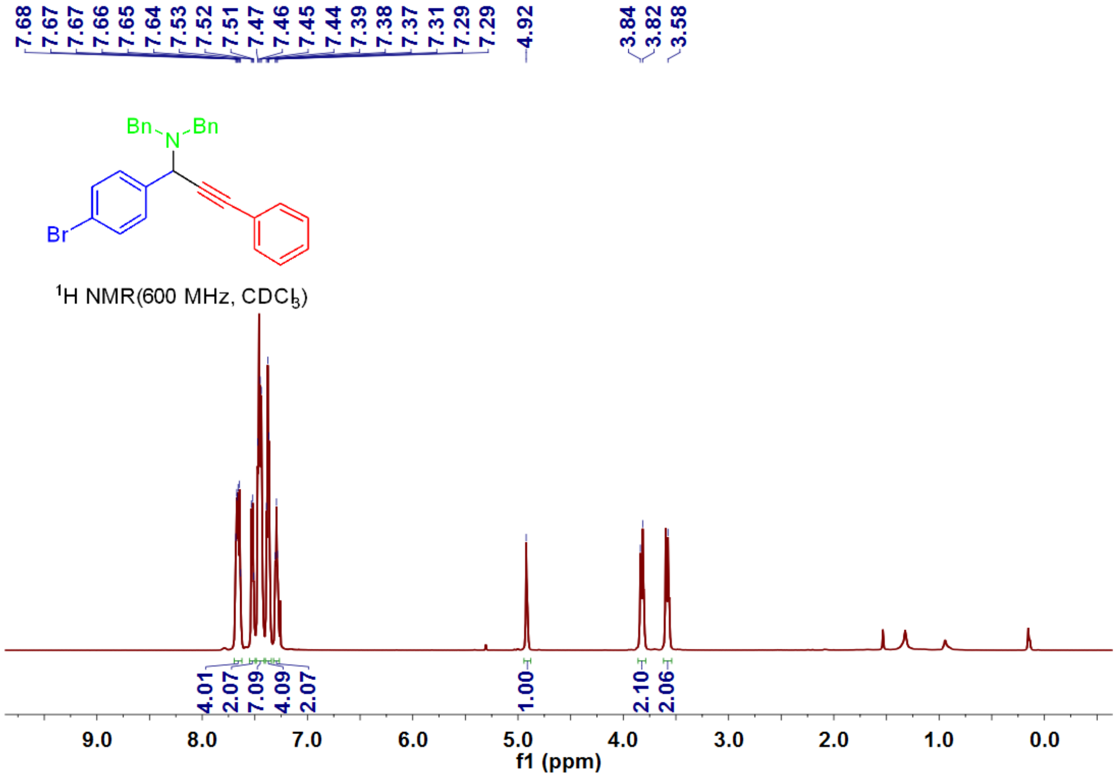

Supplementary Figure 67. ^{1}**H NMR** spectrum of compound **4j**.

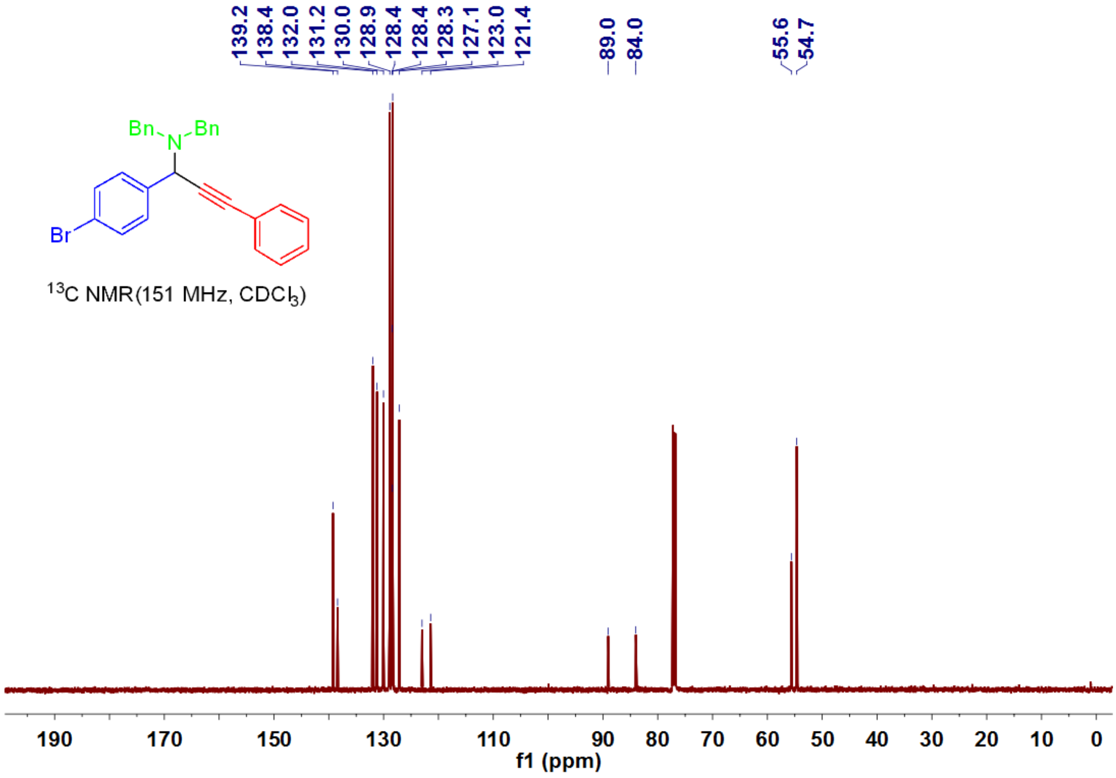

Supplementary Figure 68. ^{13}**C NMR** spectrum of compound **4j**.

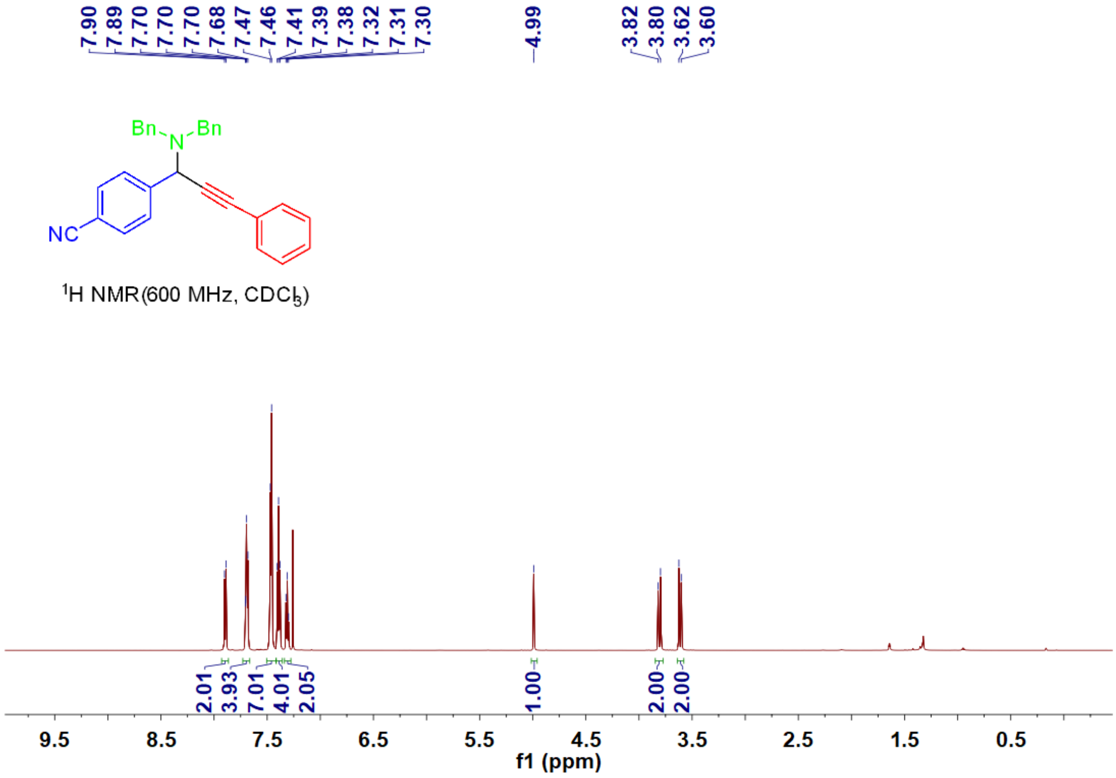

Supplementary Figure 69. ^{1}**H NMR** spectrum of compound **4k**.

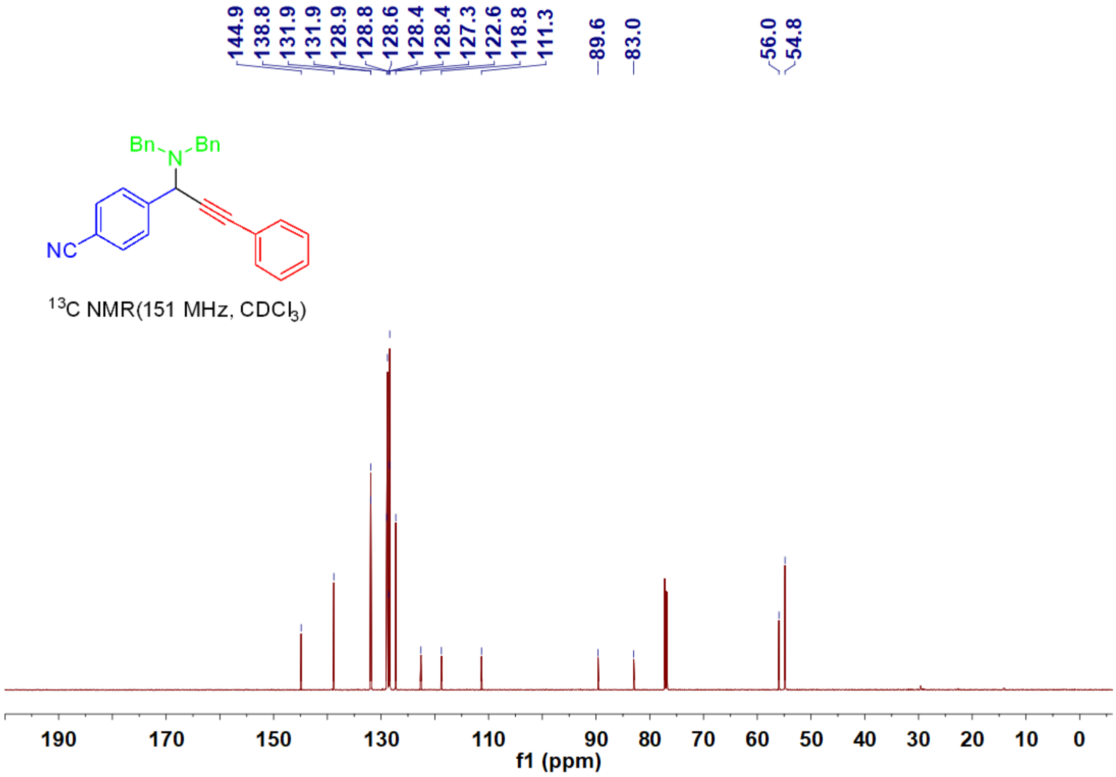

Supplementary Figure 70. ^{13}**C NMR** spectrum of compound **4k**.

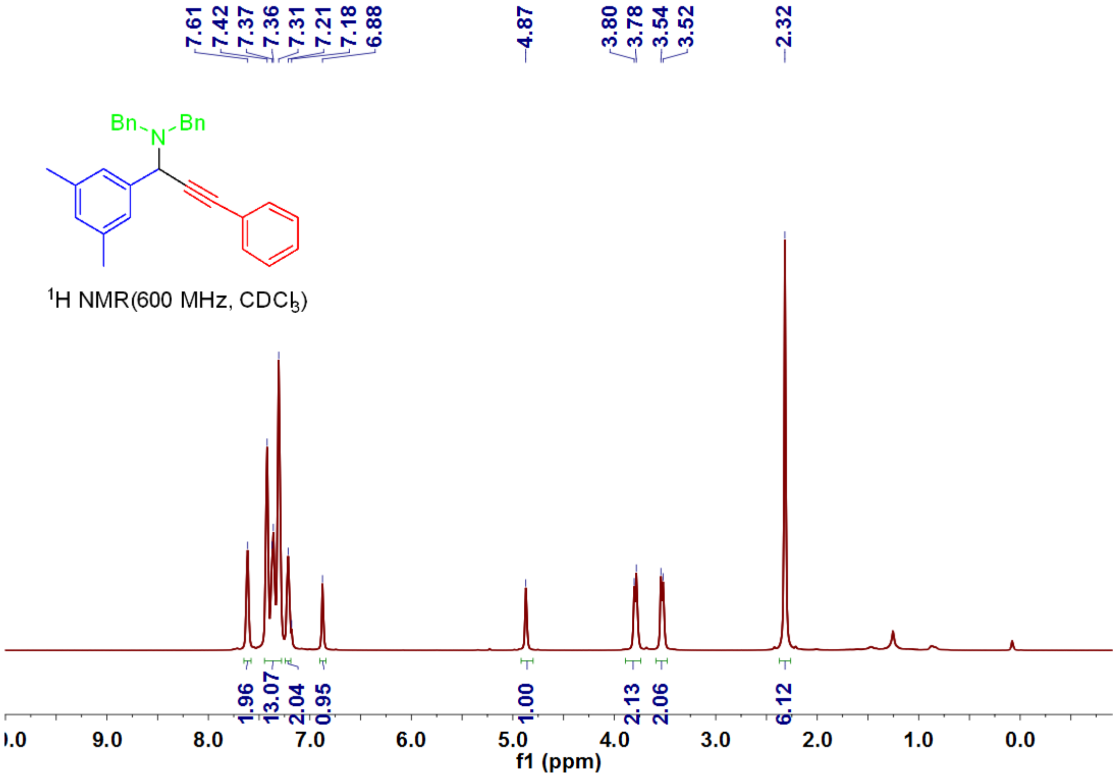

Supplementary Figure 71. ^{1}**H NMR** spectrum of compound **4l**.

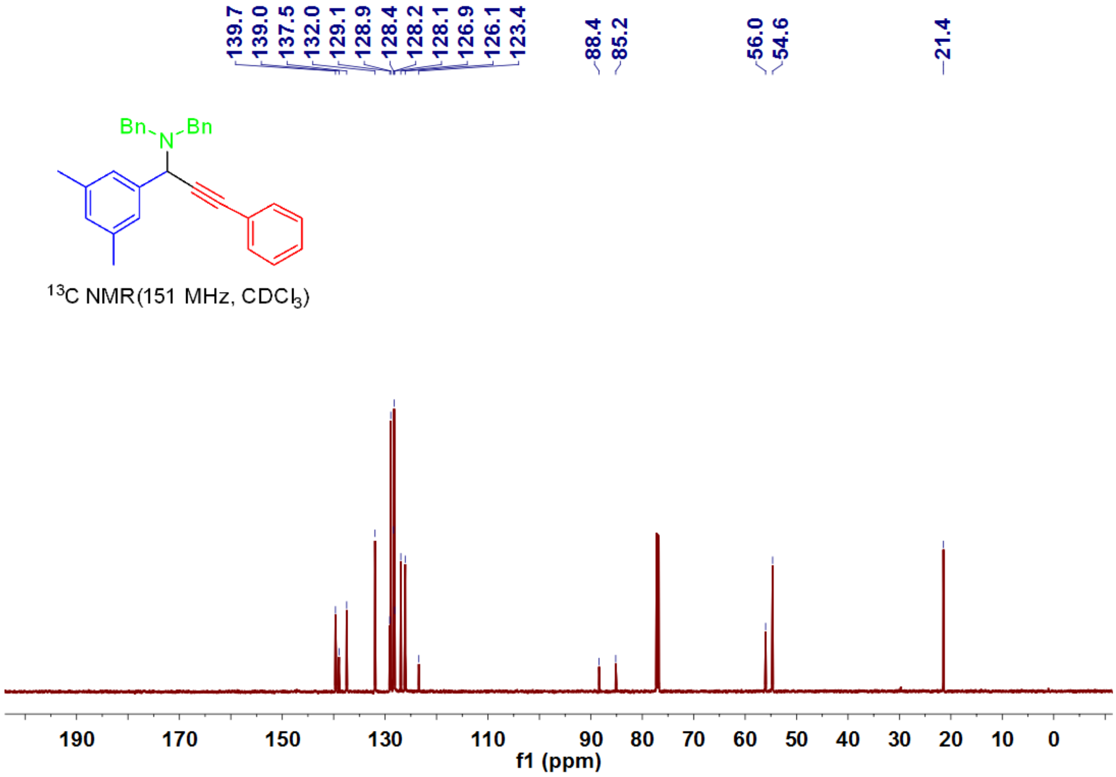

Supplementary Figure 72. ^{13}**C NMR** spectrum of compound **4l**.

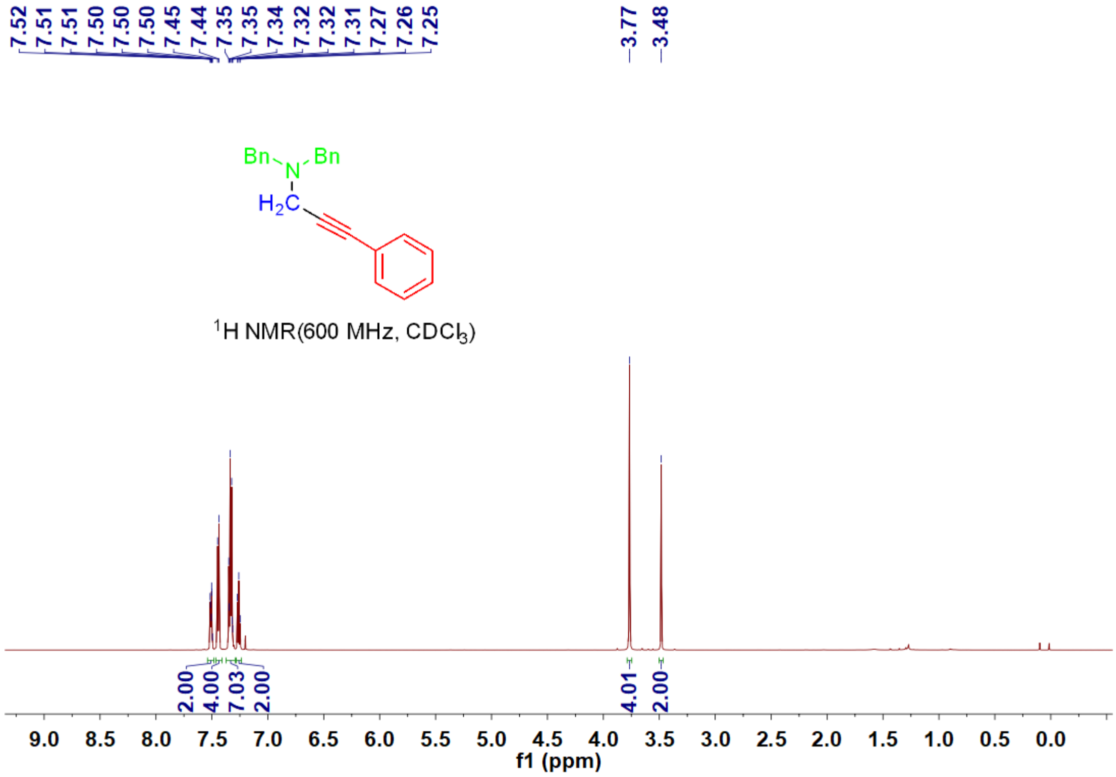

Supplementary Figure 73. ^{1}**H NMR** spectrum of compound **4m**.

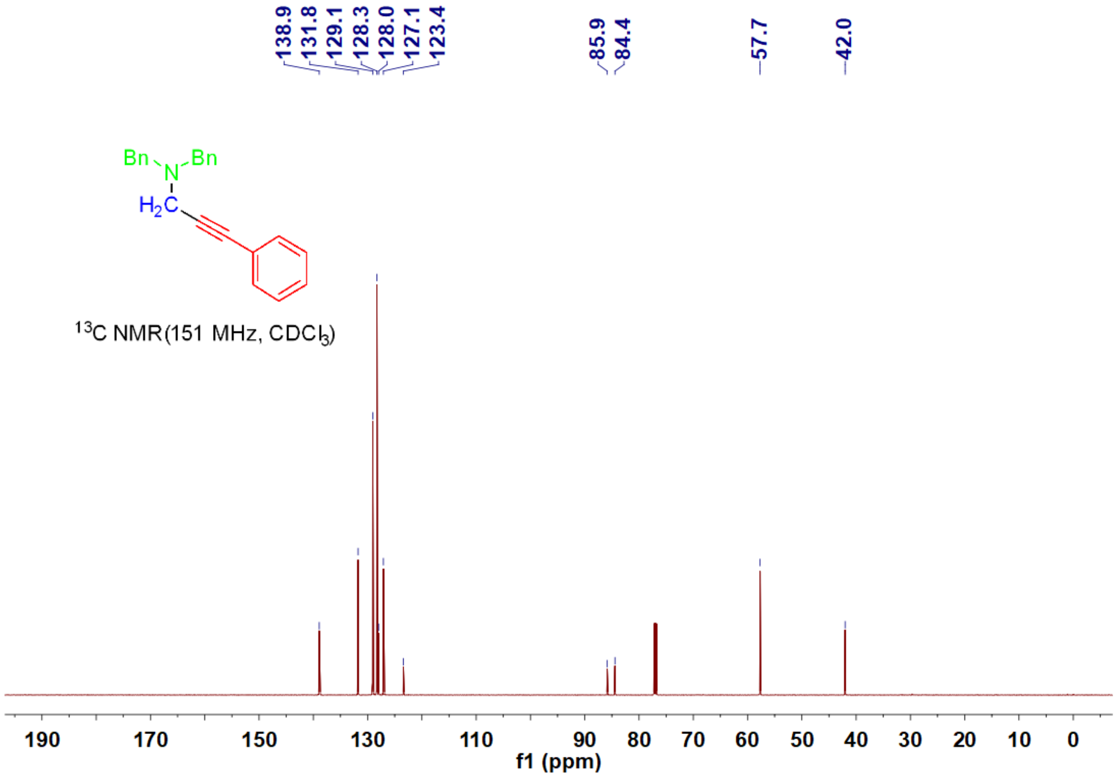

Supplementary Figure 74. ^{13}**C NMR** spectrum of compound **4m**.

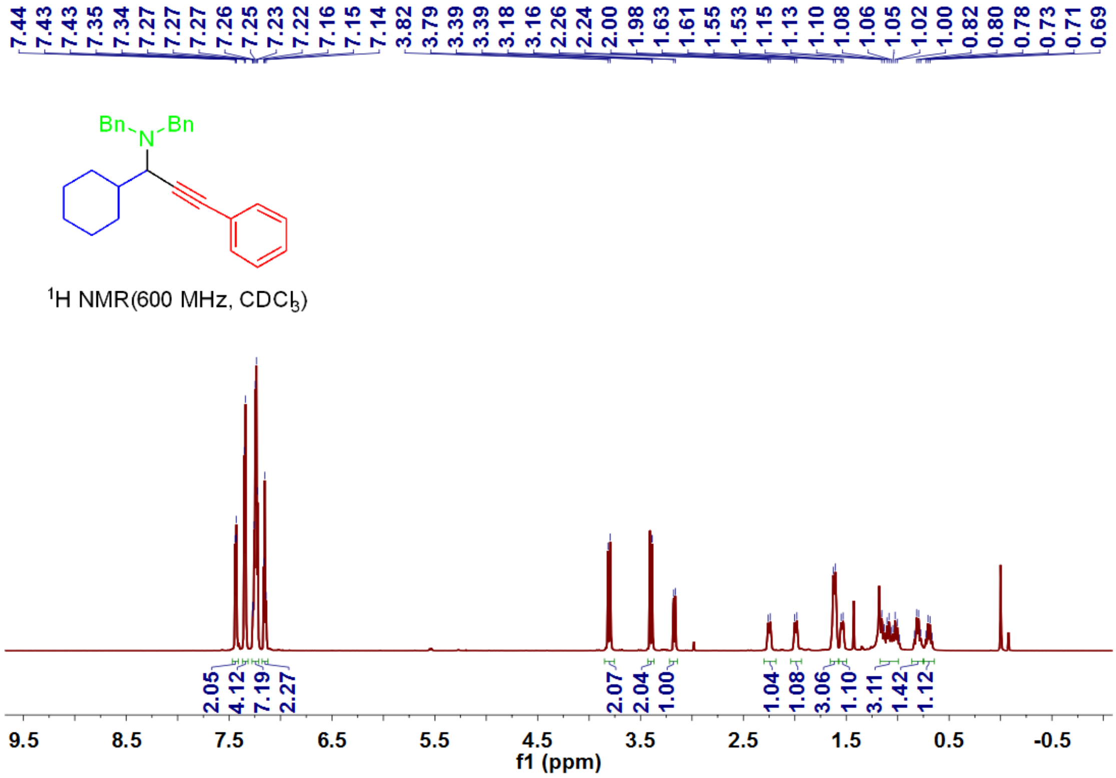

Supplementary Figure 75. ^{1}**H NMR** spectrum of compound **4n**.

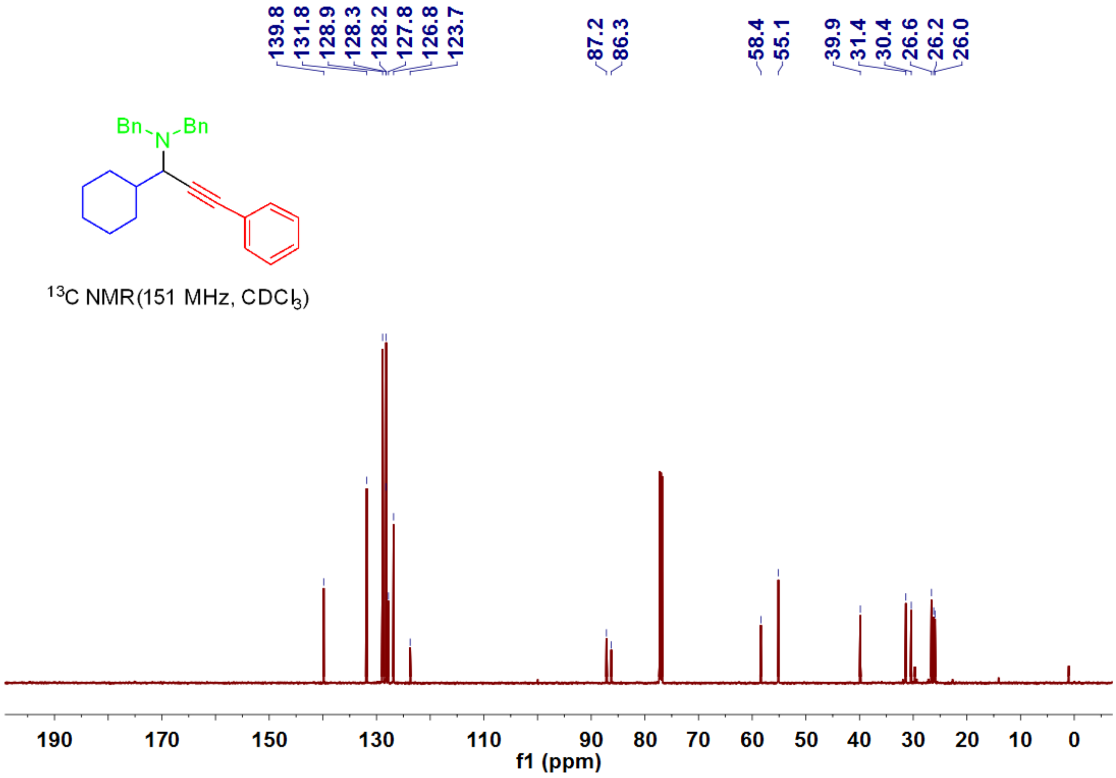

Supplementary Figure 76. ^{13}**C NMR** spectrum of compound **4n**.

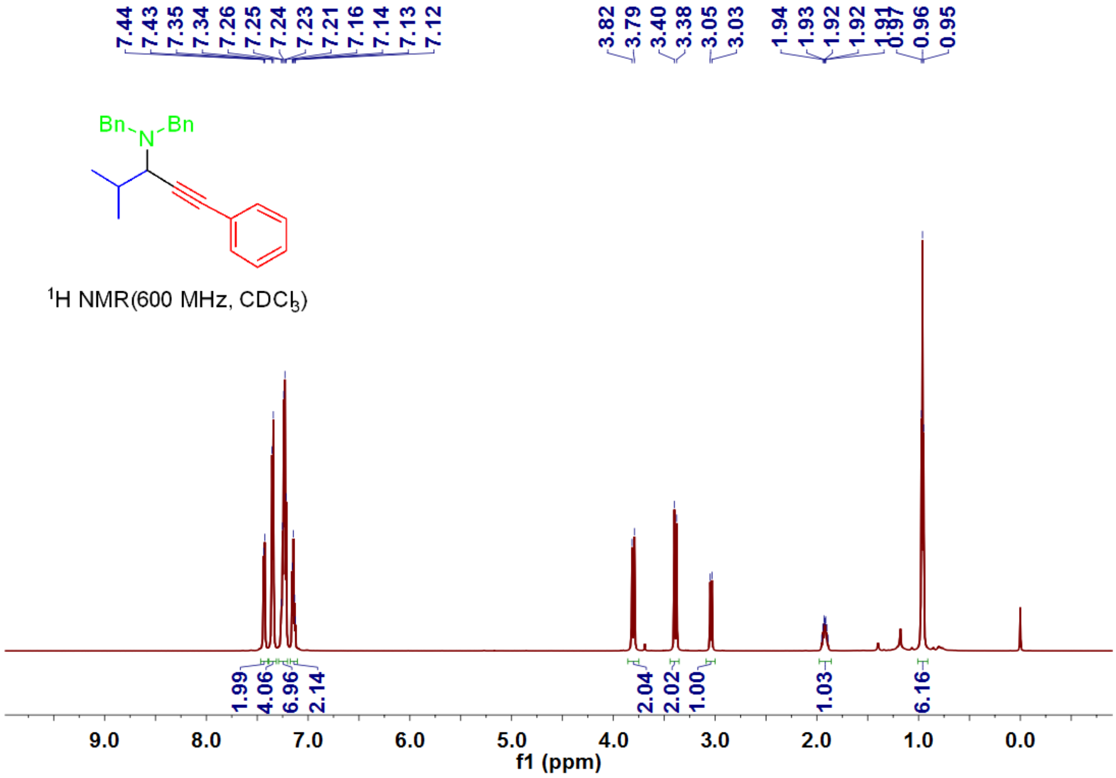

Supplementary Figure 77. ^{1}**H NMR** spectrum of compound **4o**.

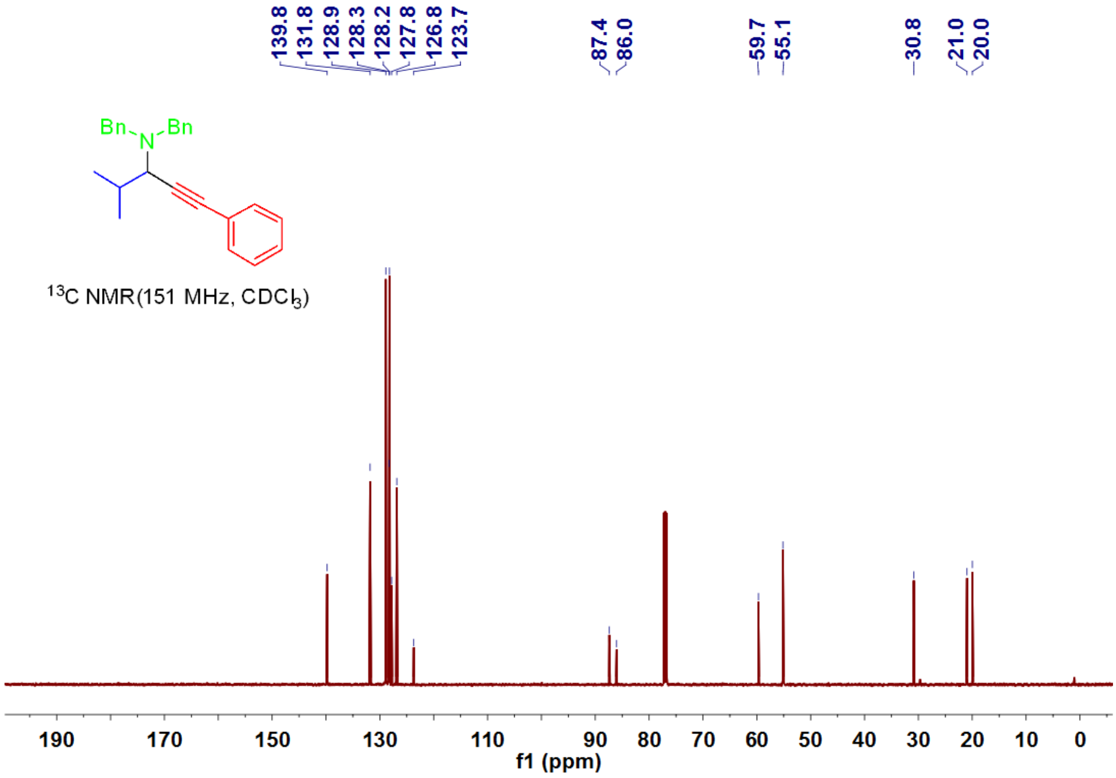

Supplementary Figure 78. ^{13}**C NMR** spectrum of compound **4o**.

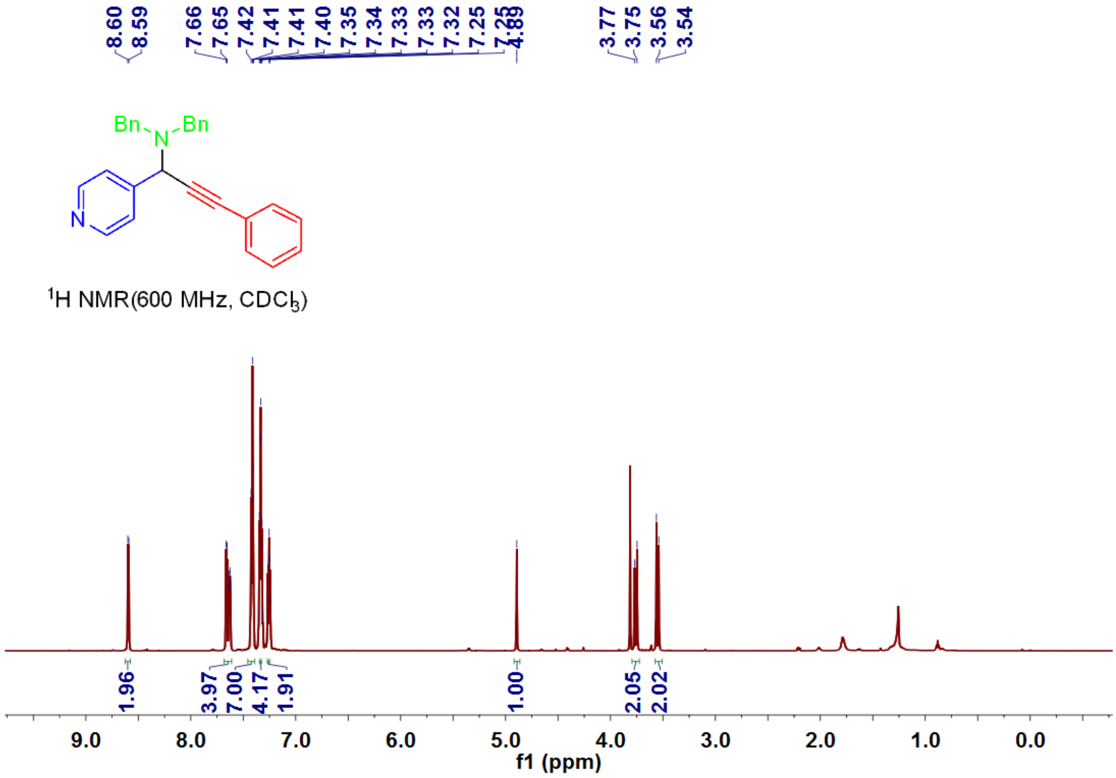

Supplementary Figure 79. ^{1}**H NMR** spectrum of compound **4p**.

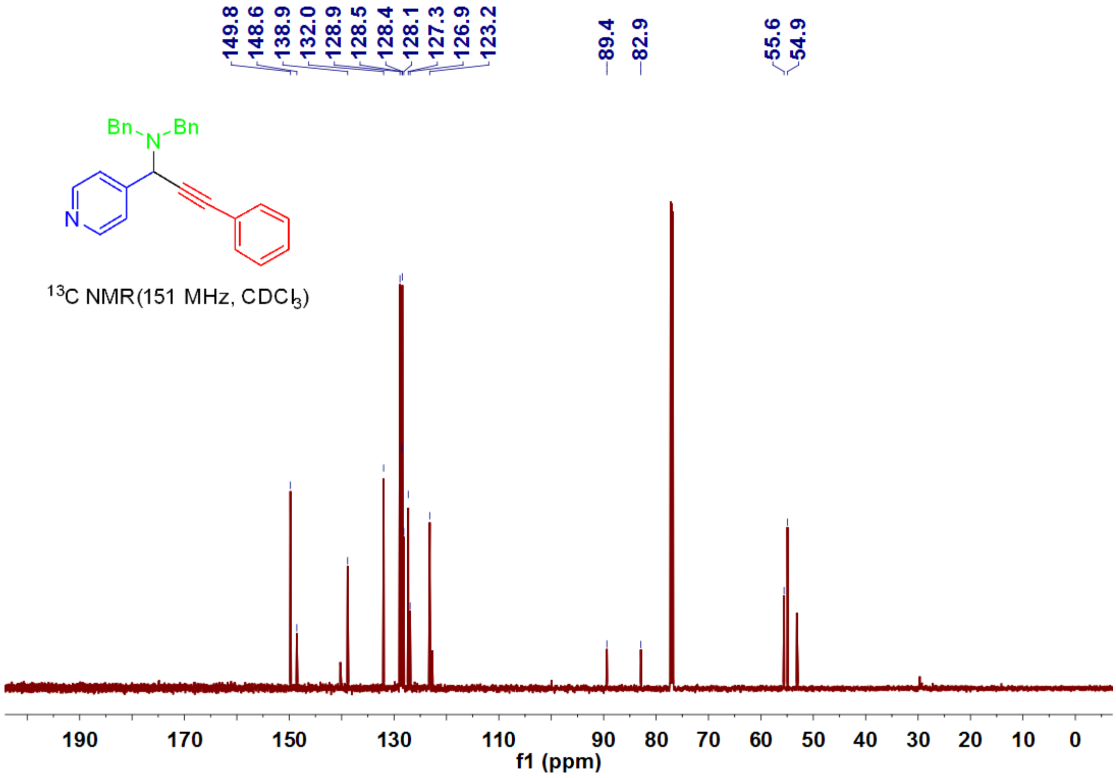

Supplementary Figure 80. ^{13}**C NMR** spectrum of compound **4p**.

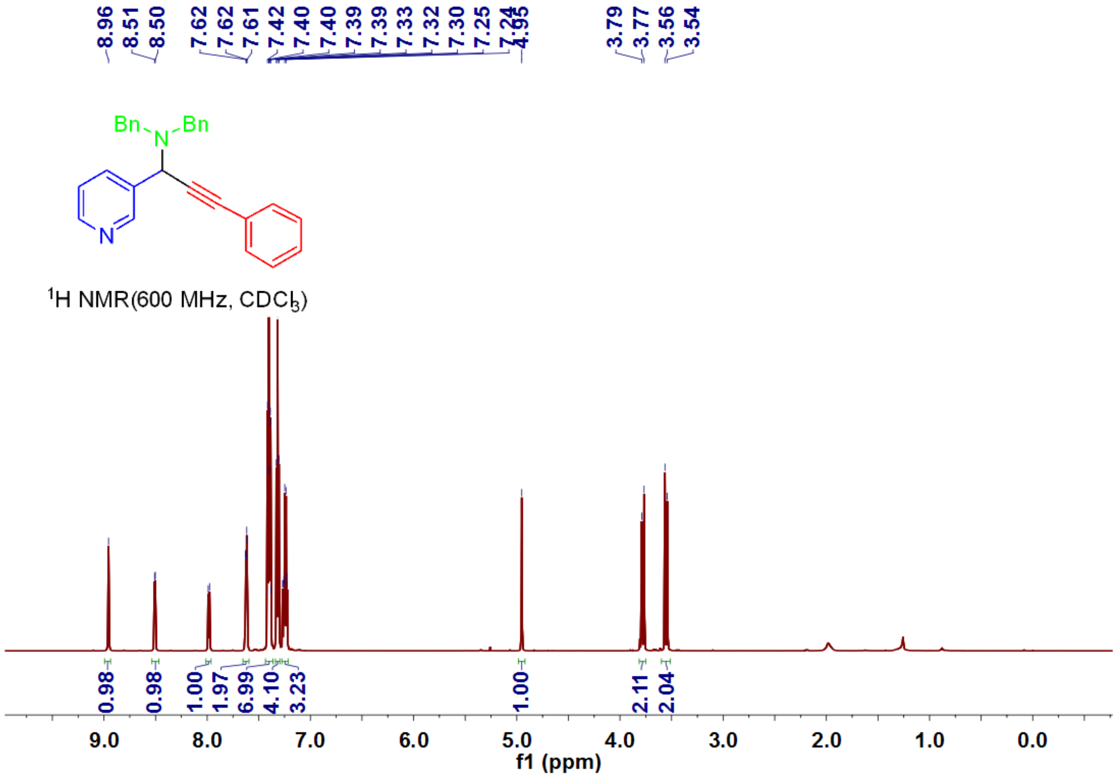

Supplementary Figure 81. ^{1}**H NMR** spectrum of compound **4q**.

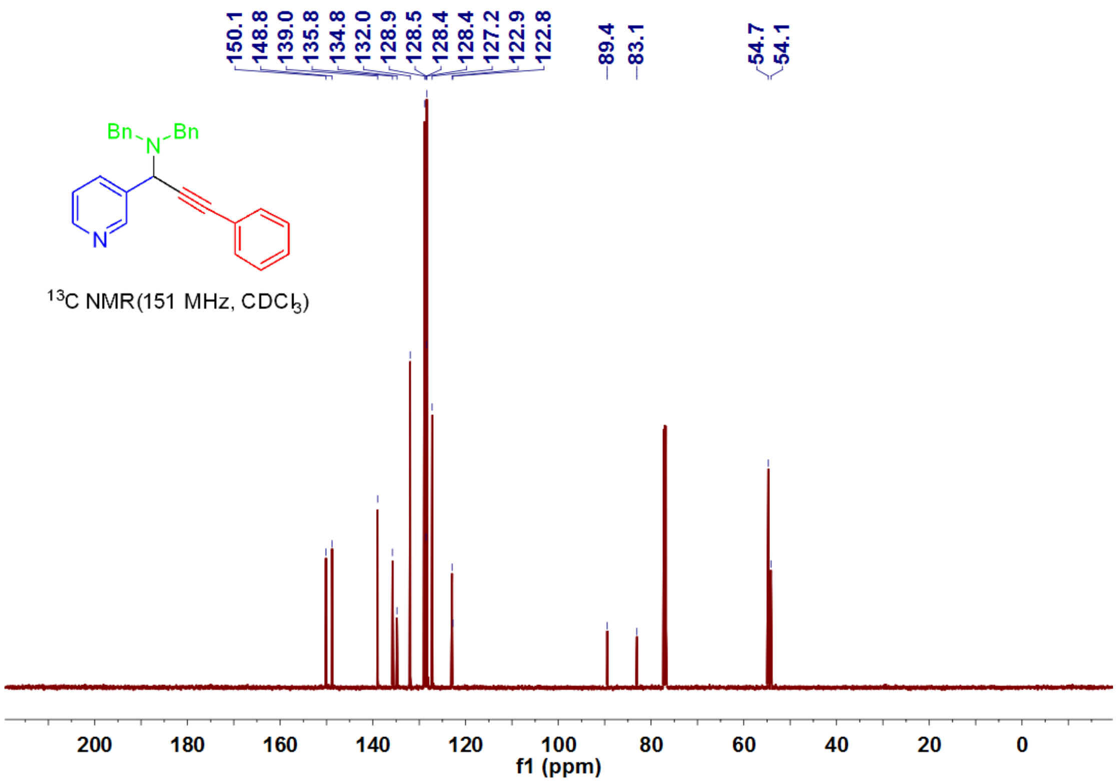

Supplementary Figure 82. **^{13}C NMR** spectrum of compound **4q**.

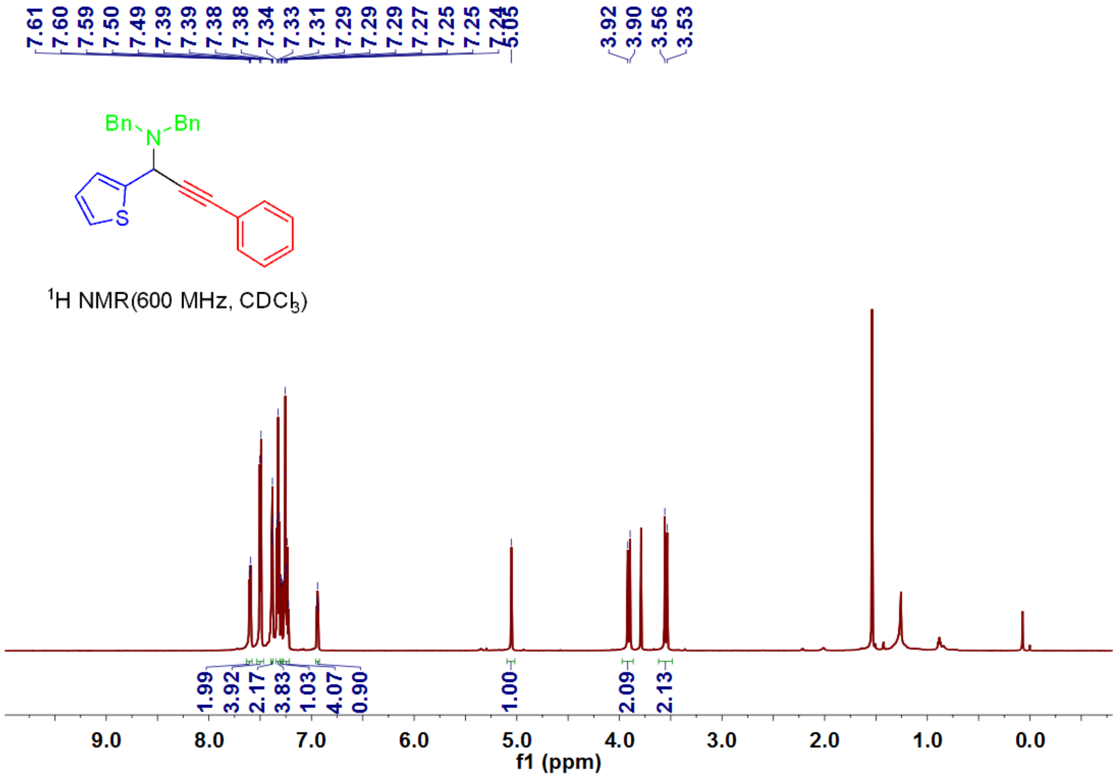

Supplementary Figure 83. **^{1}H NMR** spectrum of compound **4r**.

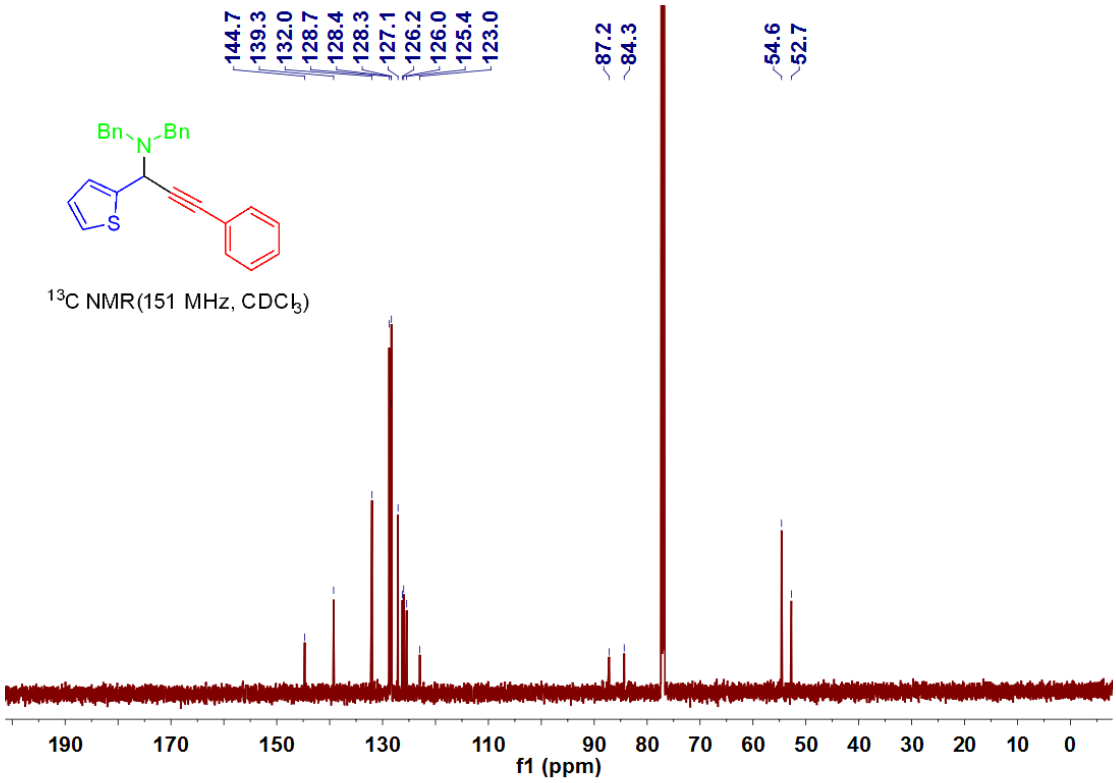

Supplementary Figure 84. **^{13}C NMR** spectrum of compound **4r**.

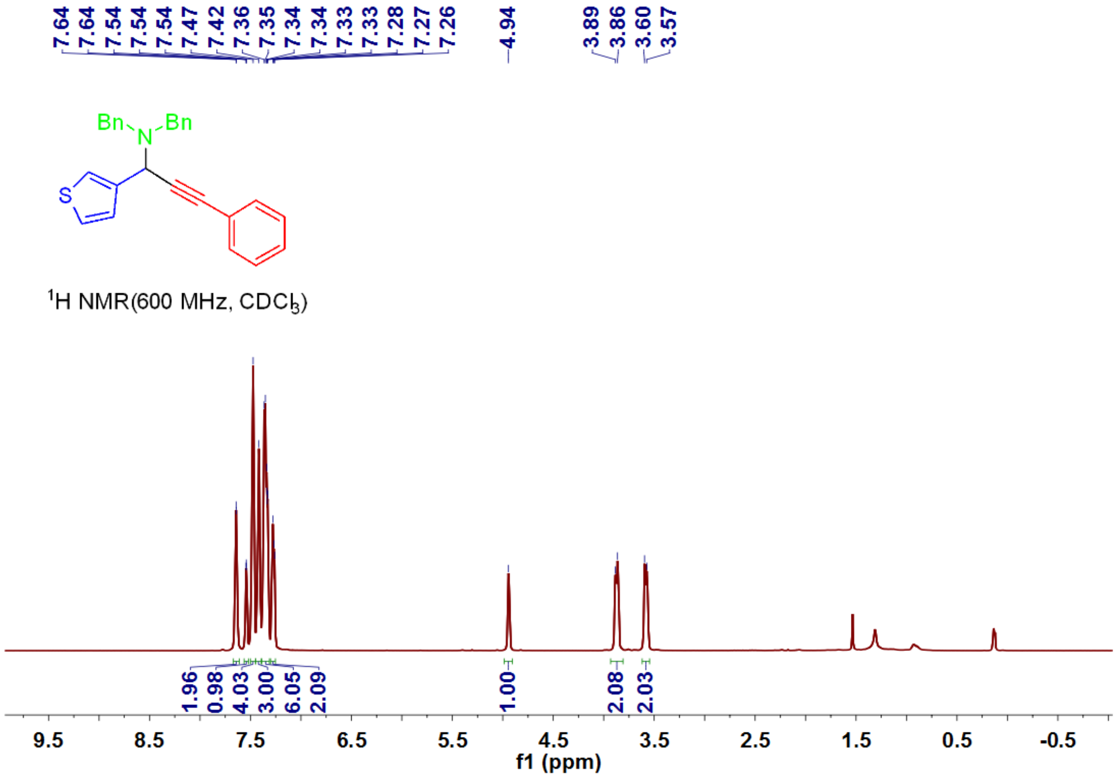

Supplementary Figure 85. **^{1}H NMR** spectrum of compound **4s**.

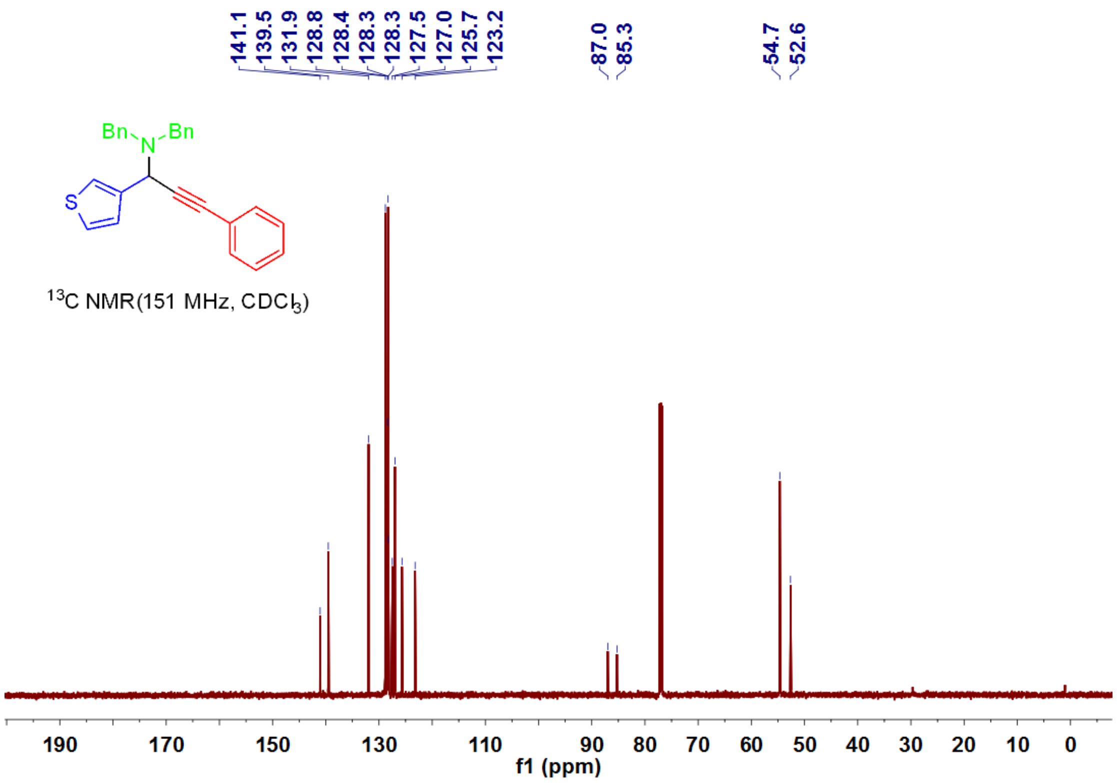

Supplementary Figure 86. ^{13}**C NMR** spectrum of compound **4s**.

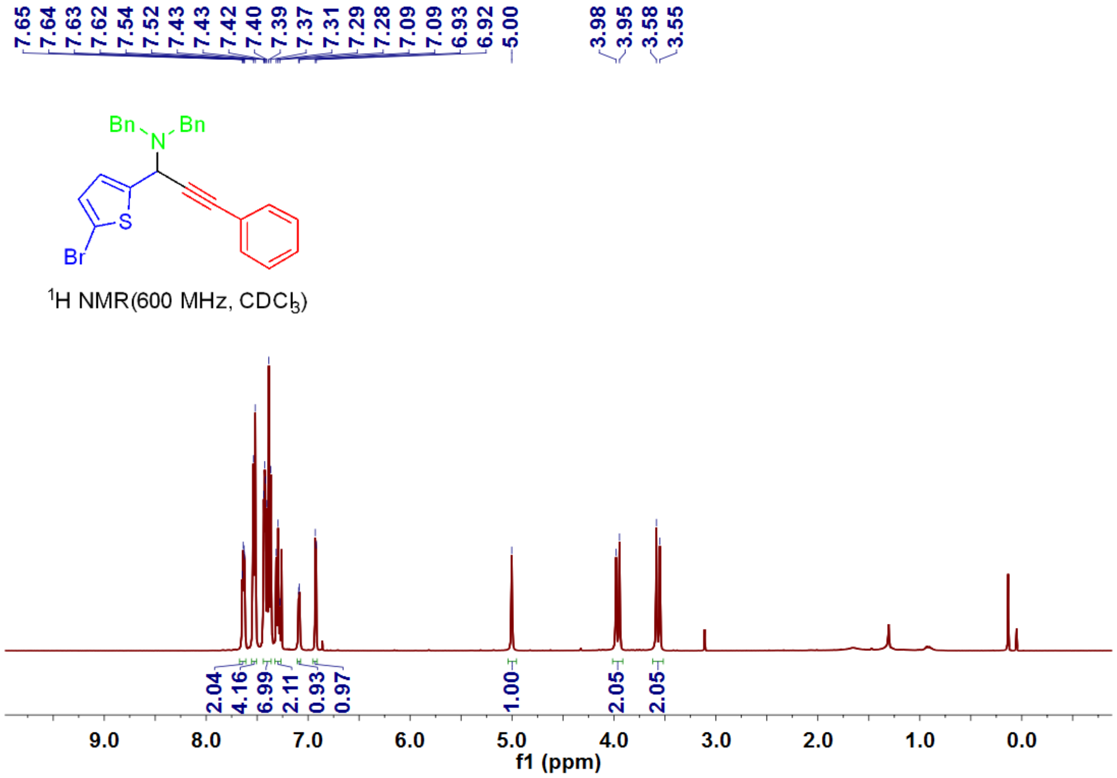

Supplementary Figure 87. ^{1}**H NMR** spectrum of compound **4t**.

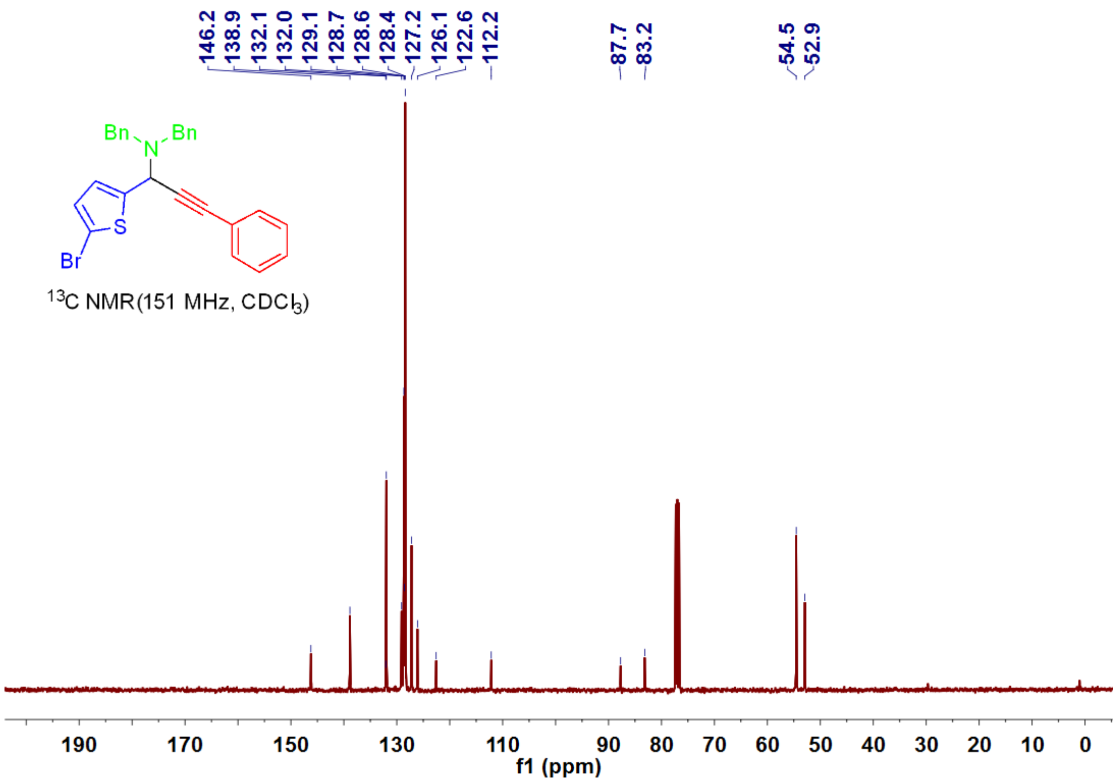

Supplementary Figure 88. ^{13}**C NMR** spectrum of compound **4t**.

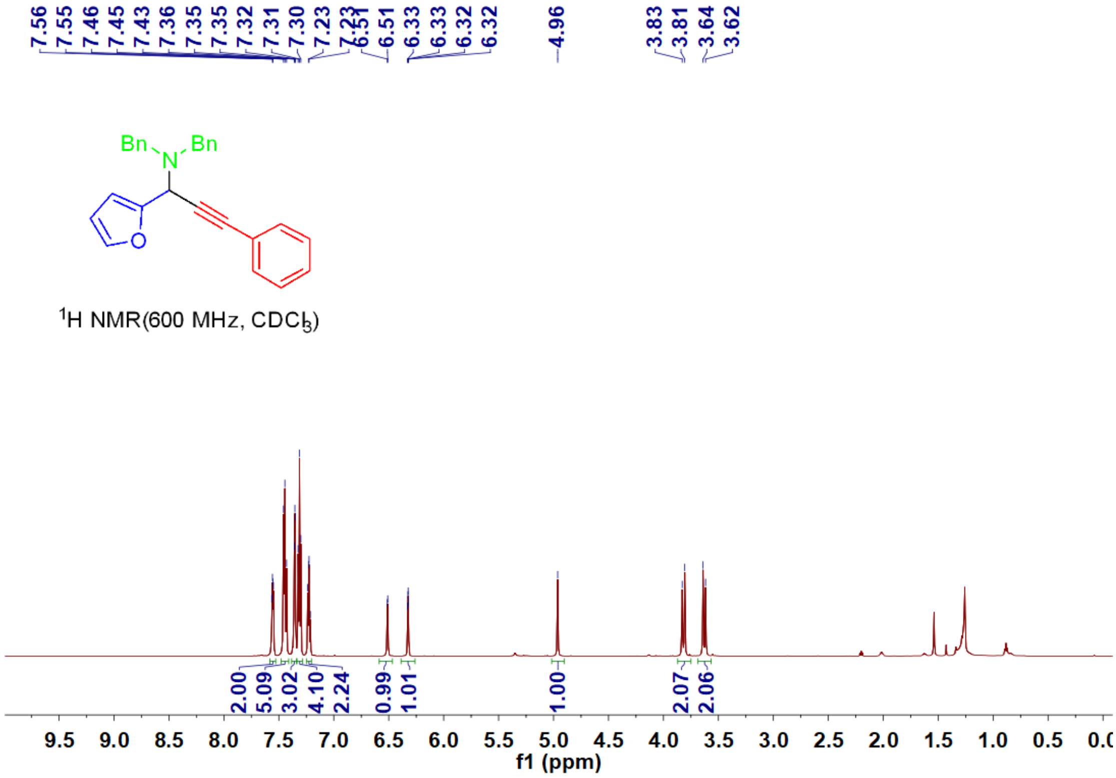

Supplementary Figure 89. ^{1}**H NMR** spectrum of compound **4u**.

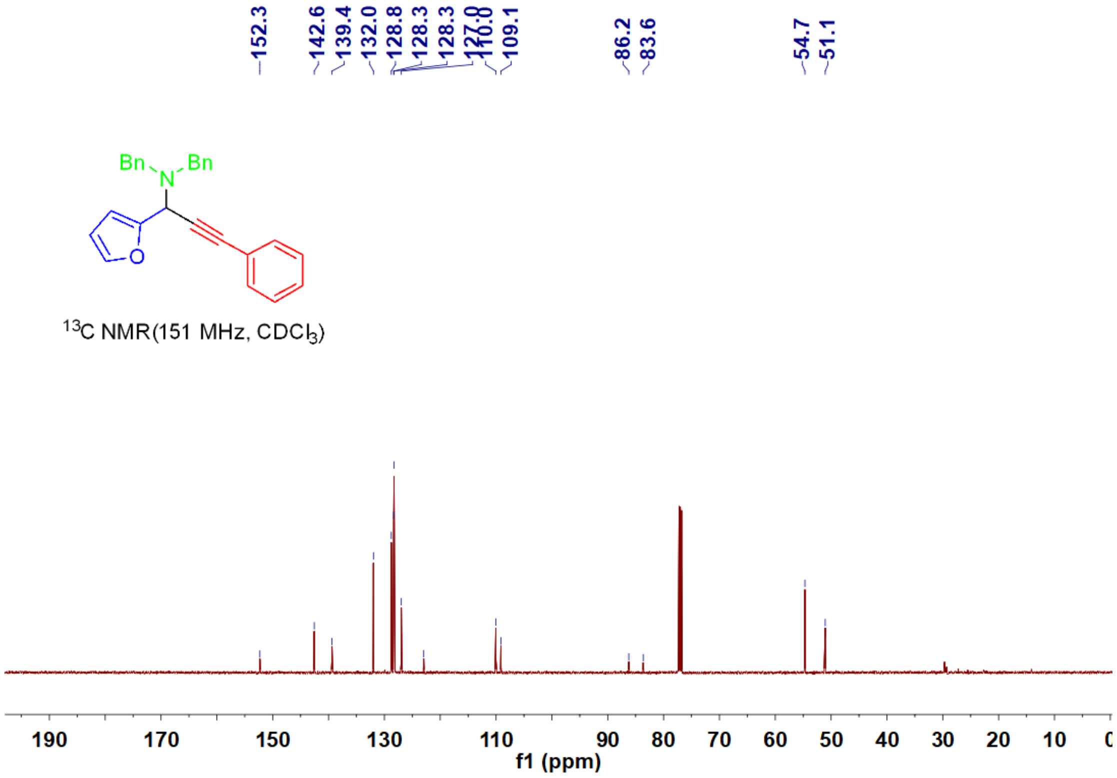

Supplementary Figure 90. ^{13}C NMR spectrum of compound **4u**.

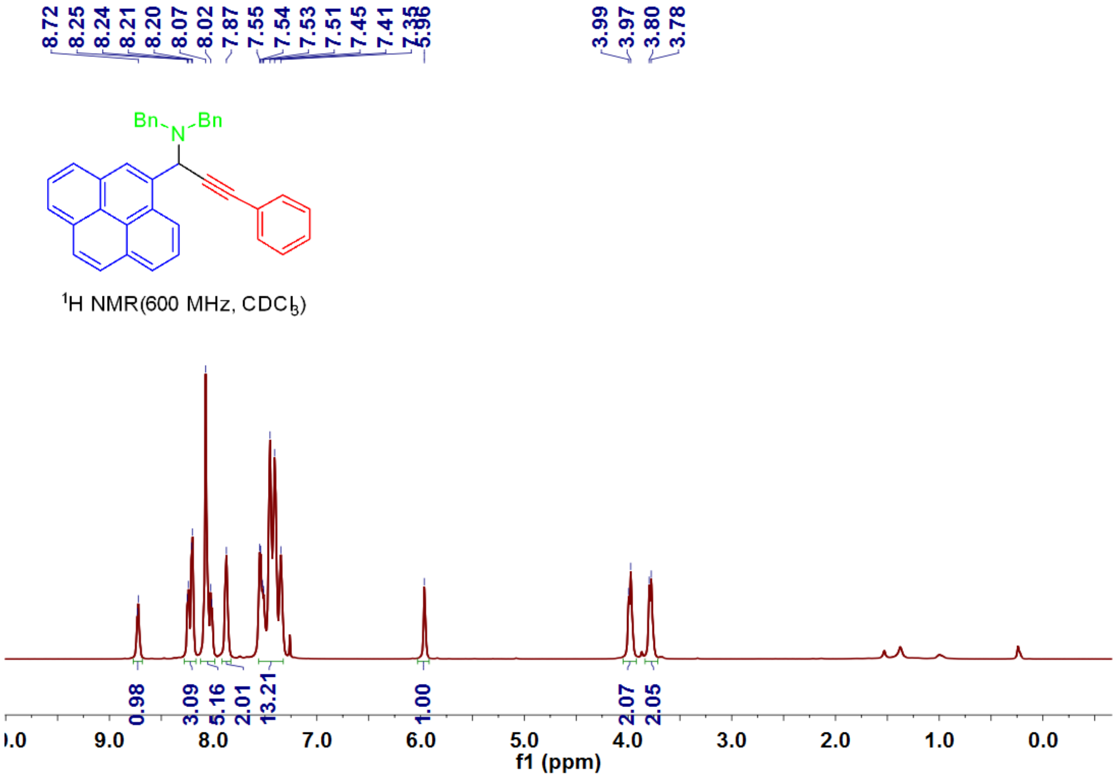

Supplementary Figure 91. ^{1}H NMR spectrum of compound **4v**.

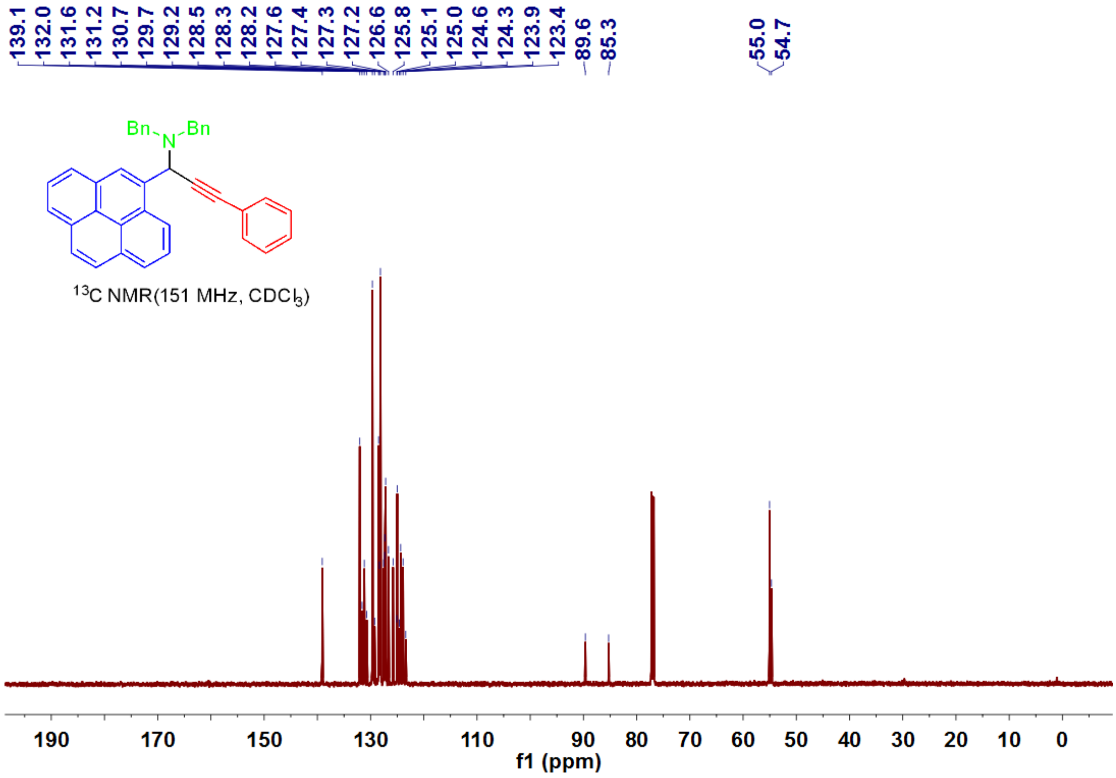

Supplementary Figure 92. ^{13}**C NMR** spectrum of compound **4v**.

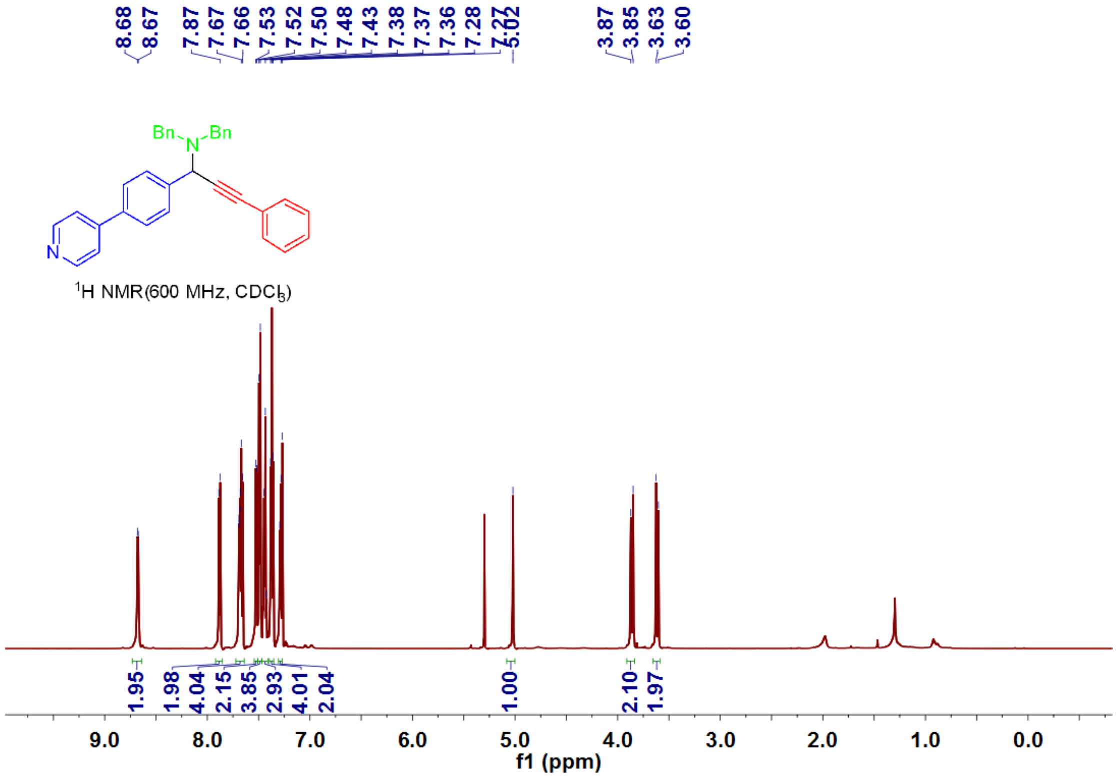

Supplementary Figure 93. ^{1}**H NMR** spectrum of compound **4w**.

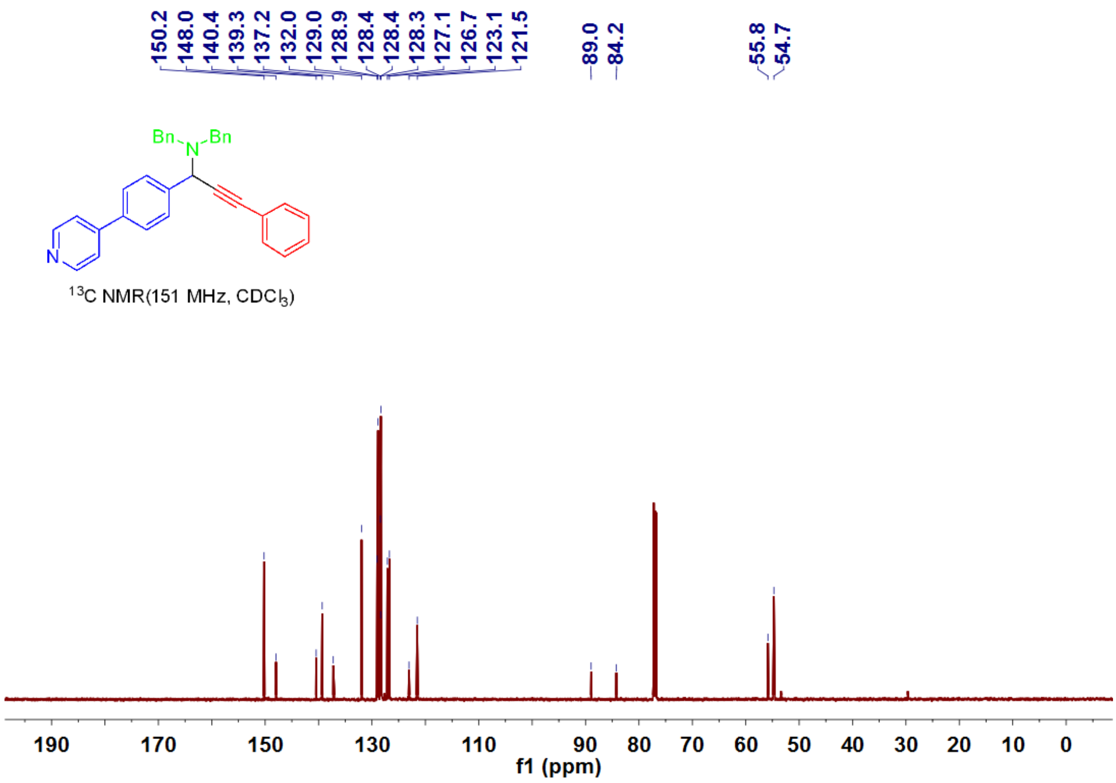

Supplementary Figure 94. ^{13}**C NMR** spectrum of compound **4w**.

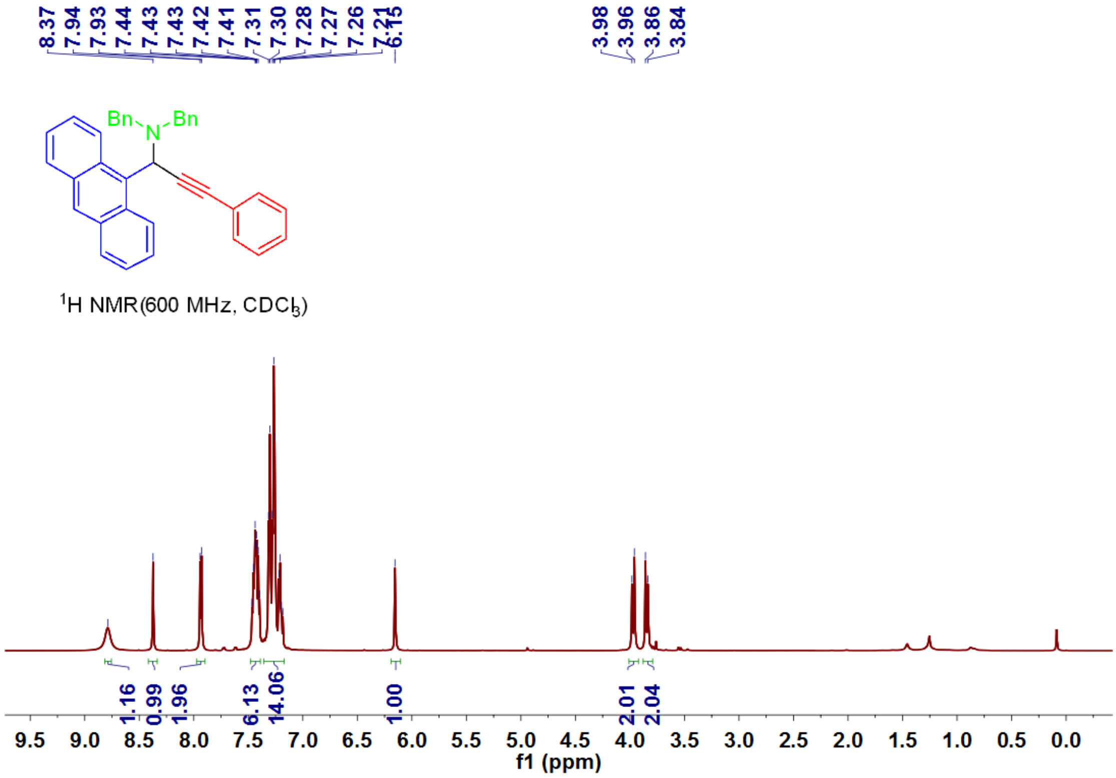

Supplementary Figure 95. ^{1}**H NMR** spectrum of compound **4x**.

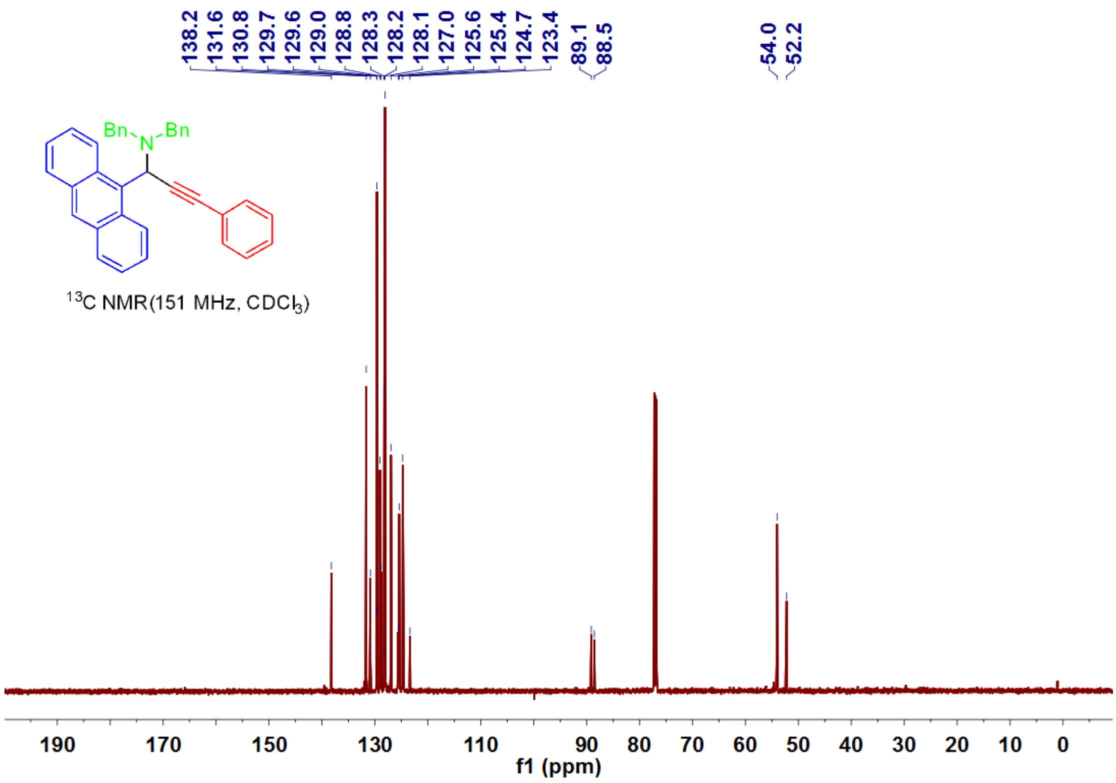

Supplementary Figure 96. ^{13}**C NMR** spectrum of compound **4x**.

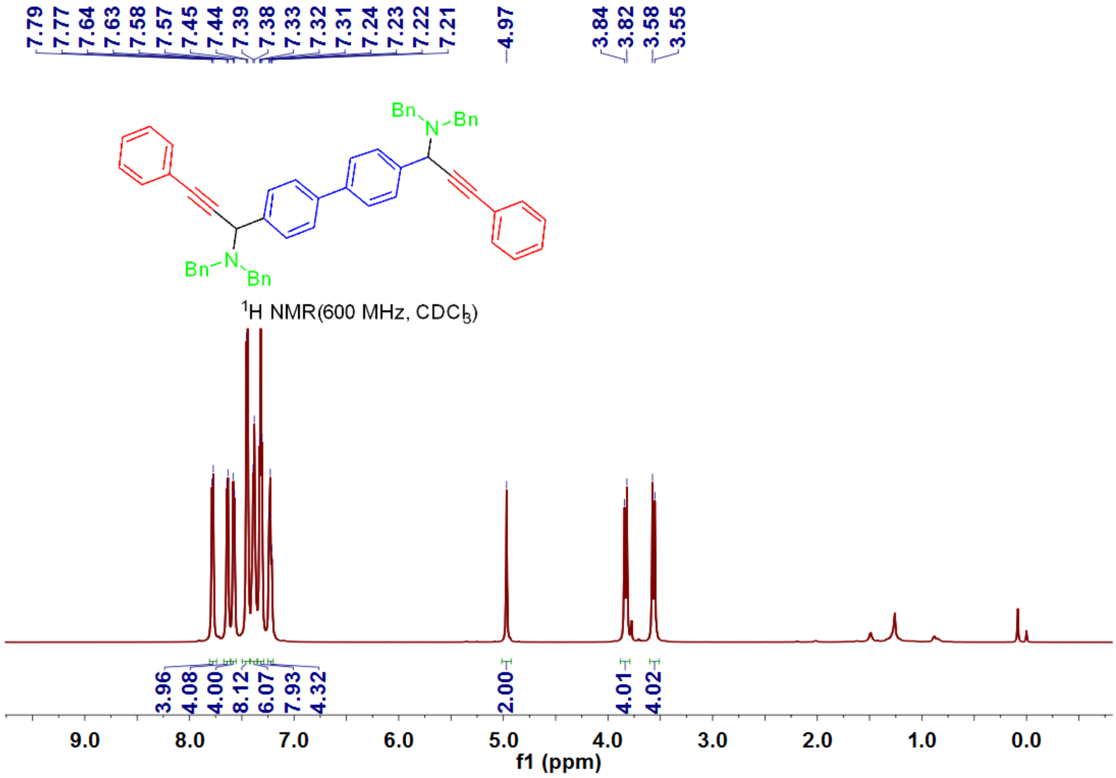

Supplementary Figure 97. ^{1}**H NMR** spectrum of compound **4y**.

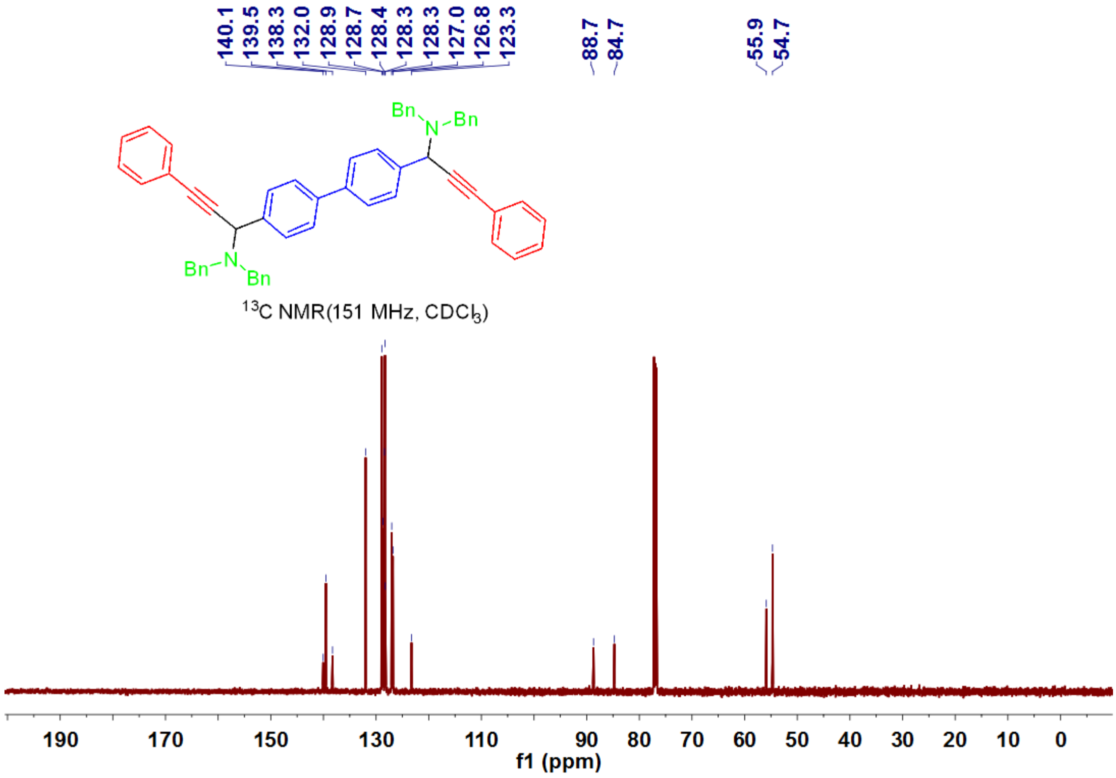

Supplementary Figure 98. ^{13}**C NMR** spectrum of compound **4y**.

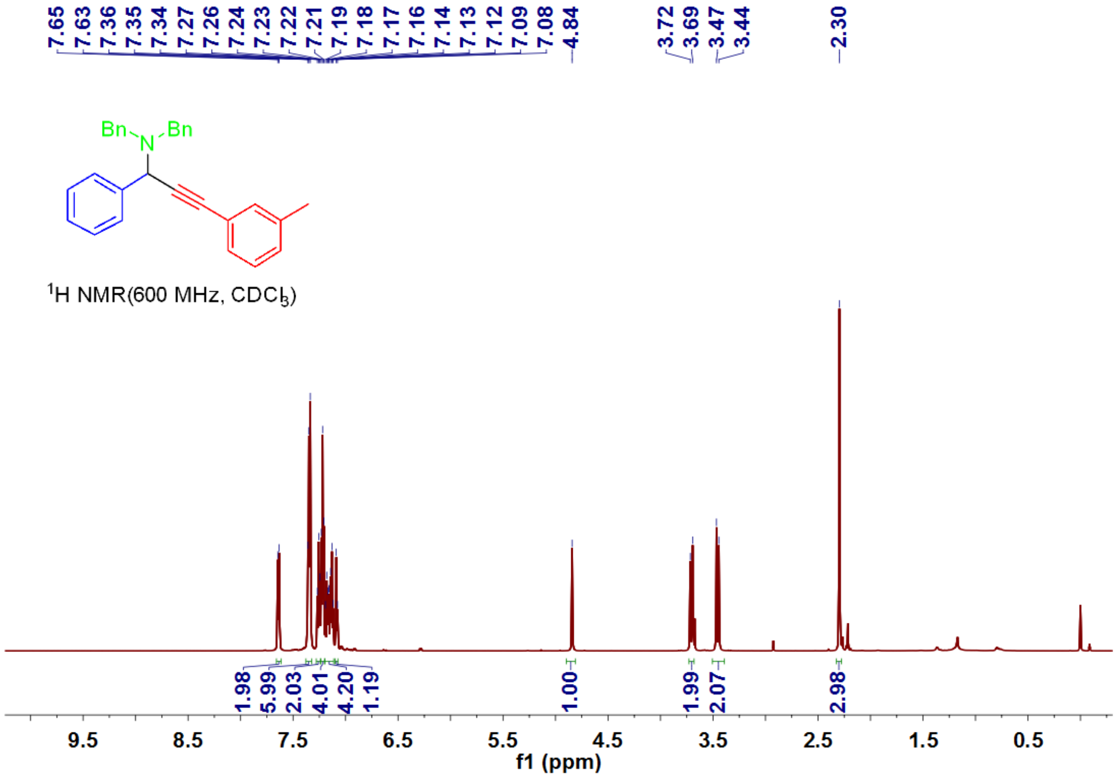

Supplementary Figure 99. ^{1}**H NMR** spectrum of compound **4z**.

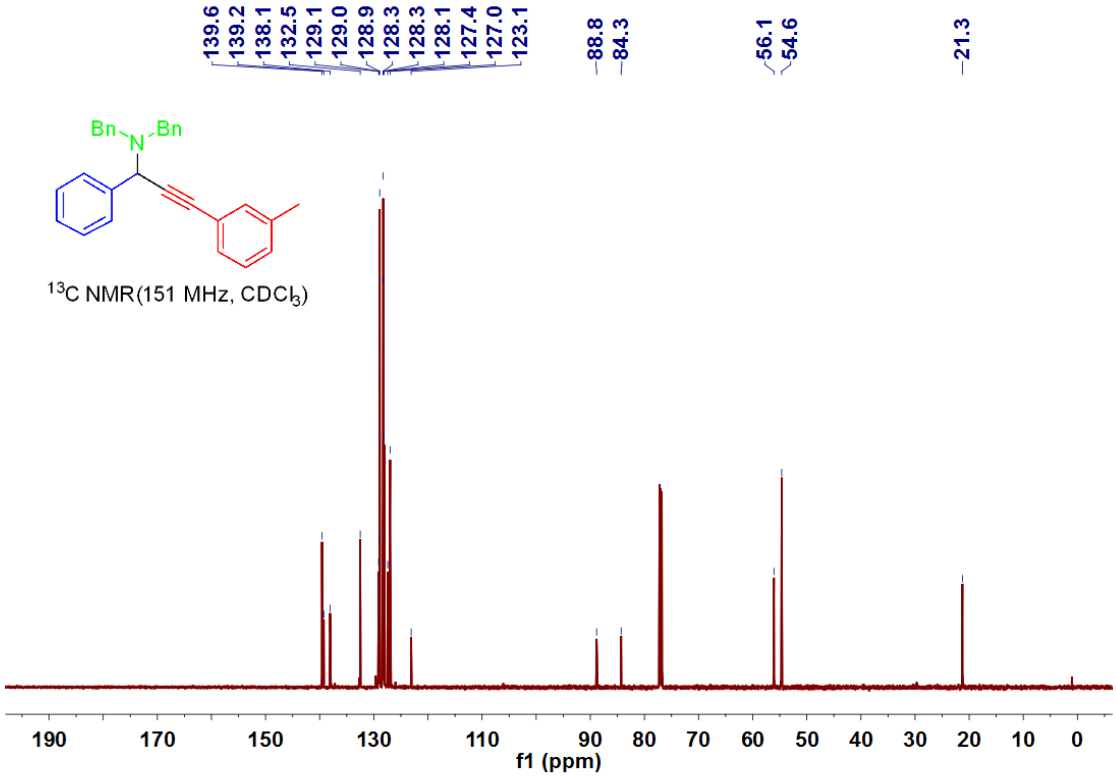

Supplementary Figure 100. ^{13}**C NMR** spectrum of compound **4z**.

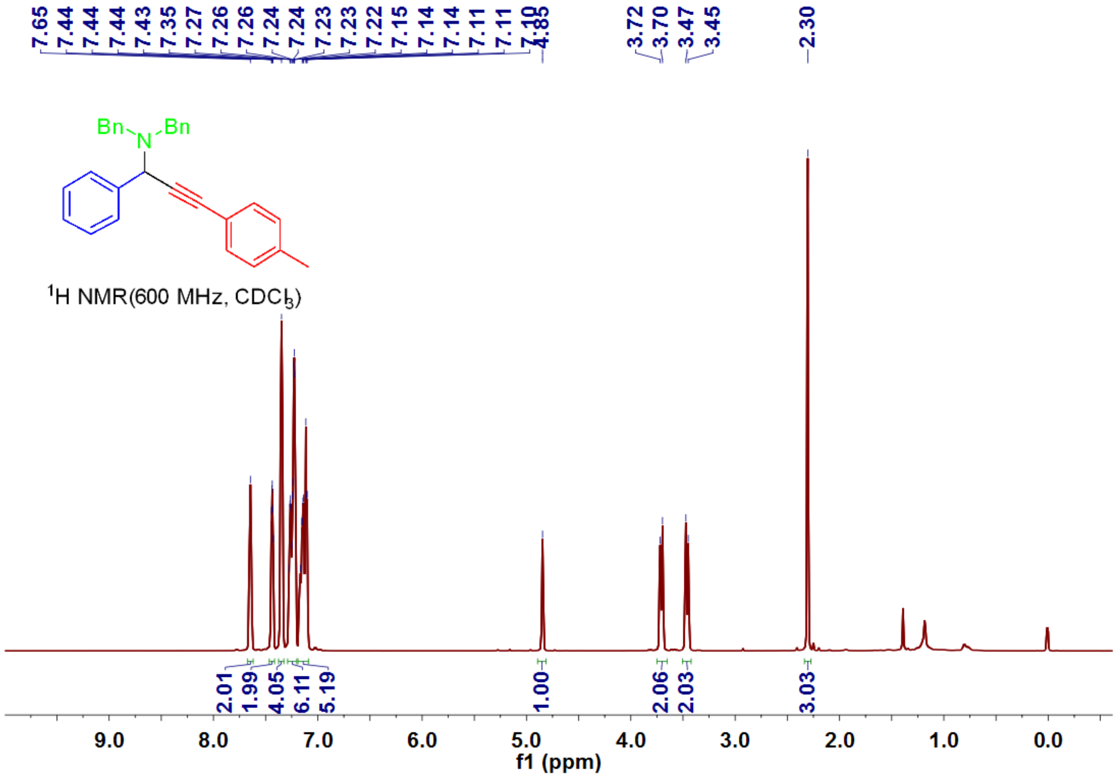

Supplementary Figure 101. ^{1}**H NMR** spectrum of compound **4aa**.

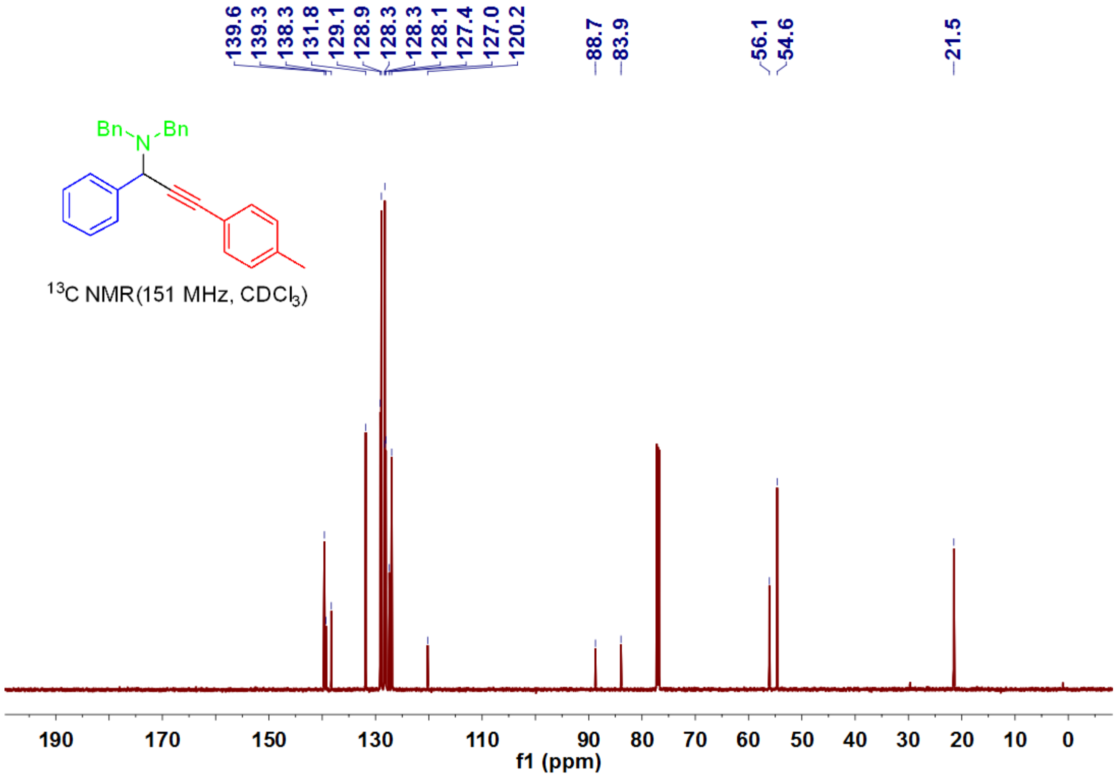

Supplementary Figure 102. ^{13}**C NMR** spectrum of compound **4aa**.

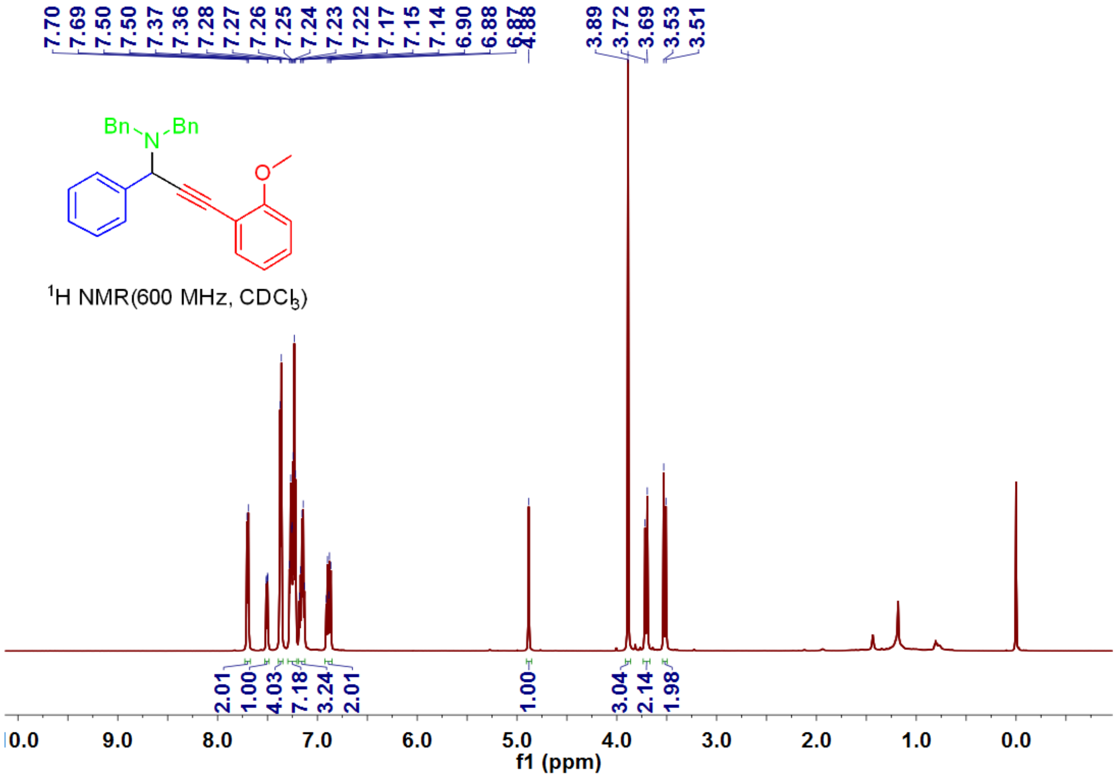

Supplementary Figure 103. ^{1}**H NMR** spectrum of compound **4ab**.

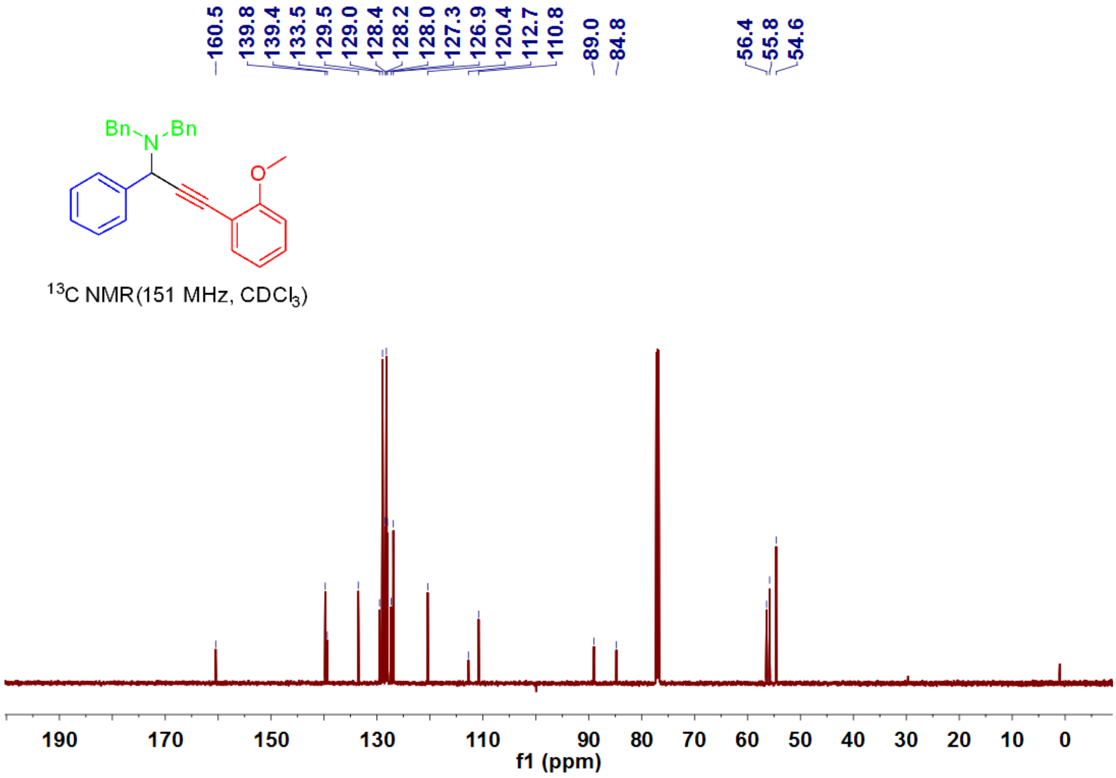

Supplementary Figure 104. ^{13}**C NMR** spectrum of compound **4ab**.

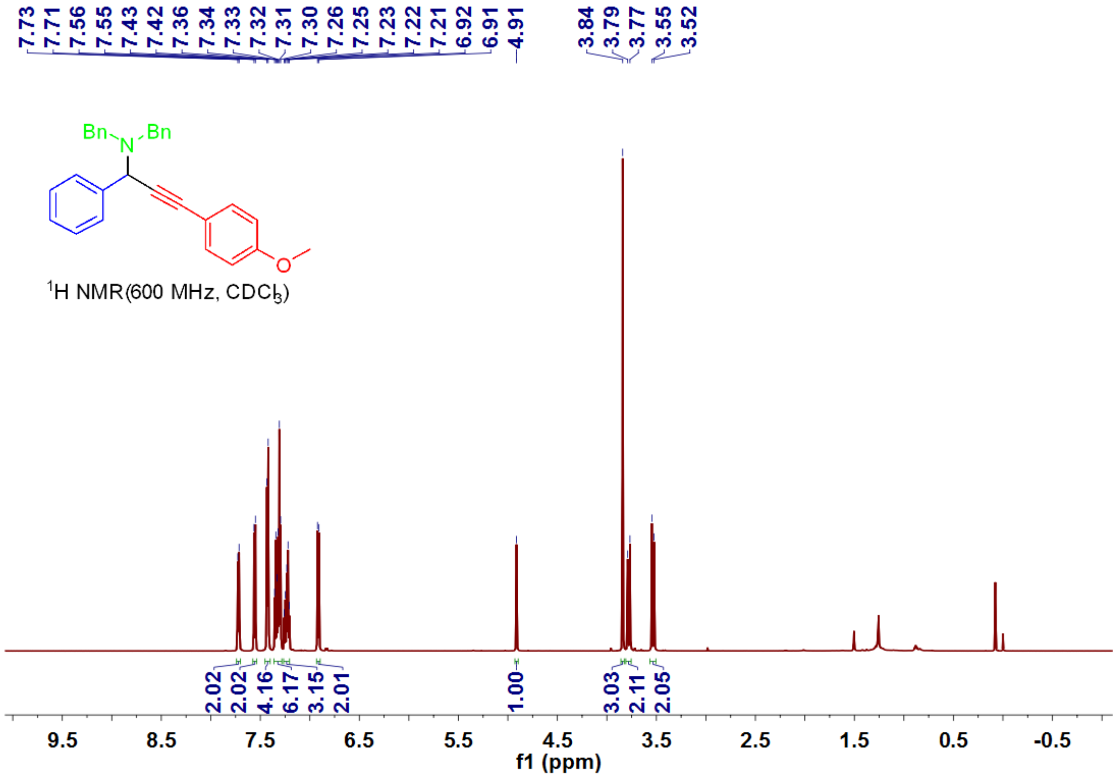

Supplementary Figure 105. ^{1}**H NMR** spectrum of compound **4ac**.

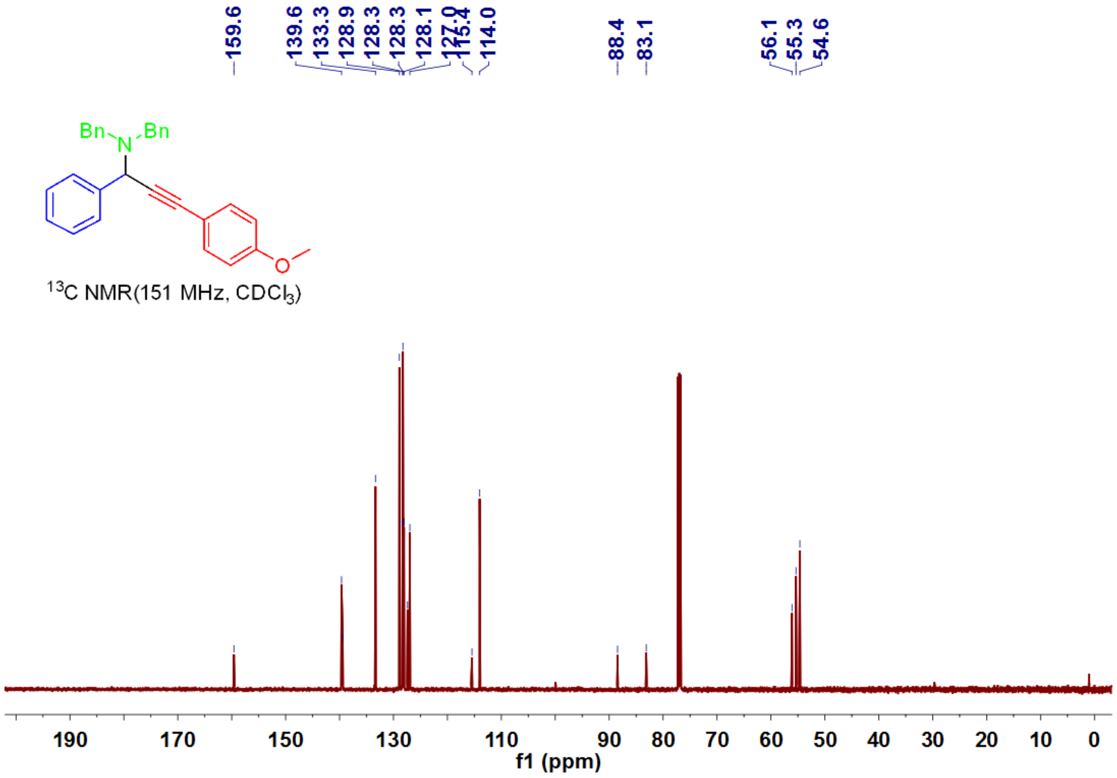

Supplementary Figure 106. ^{13}**C NMR** spectrum of compound **4ac**.

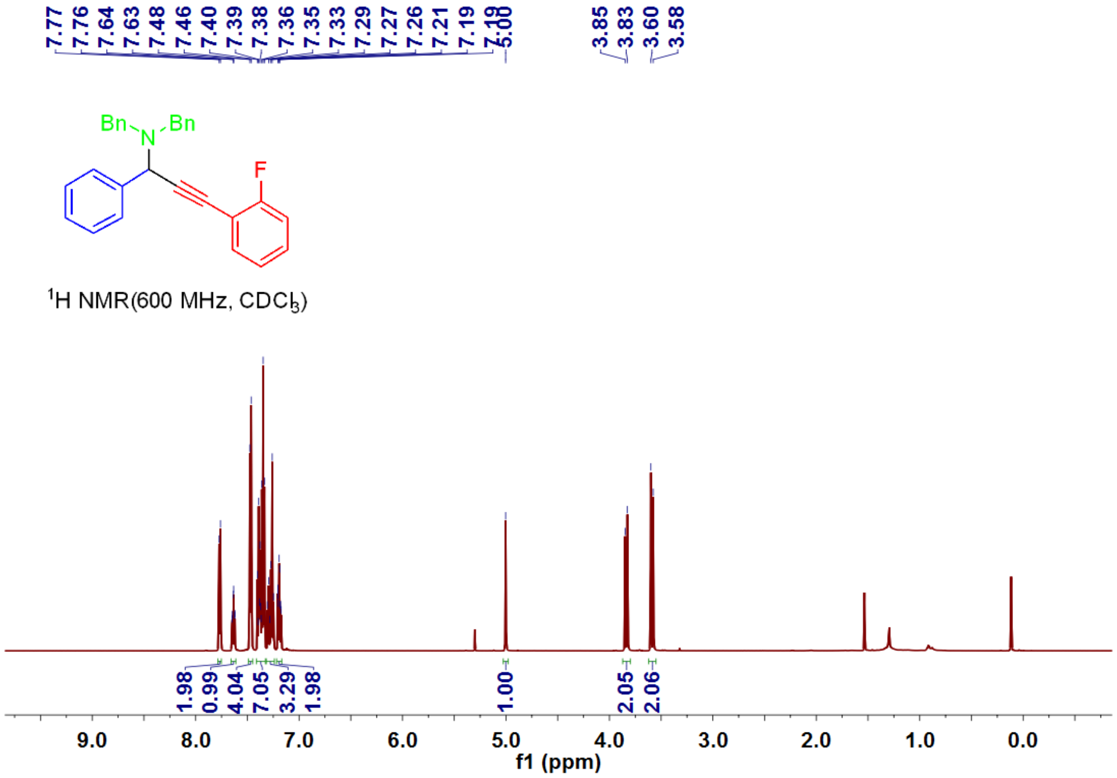

Supplementary Figure 107. ^{1}**H NMR** spectrum of compound **4ad**.

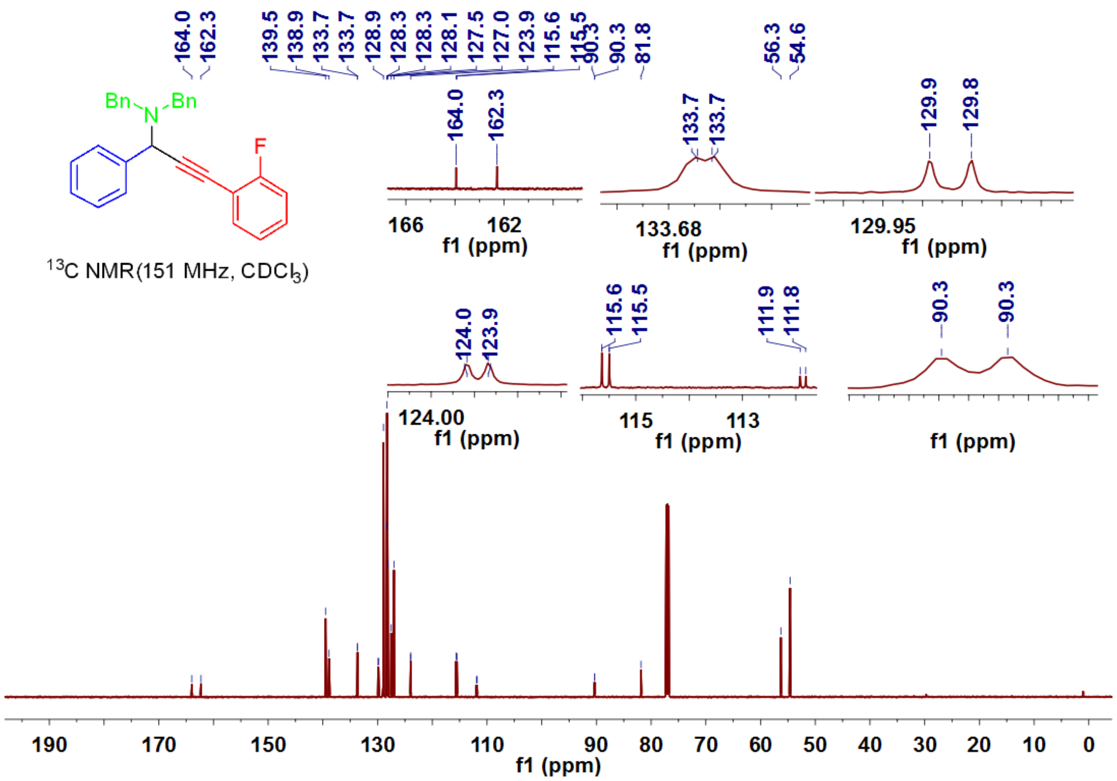

Supplementary Figure 108. **^{13}C NMR** spectrum of compound **4ad**.

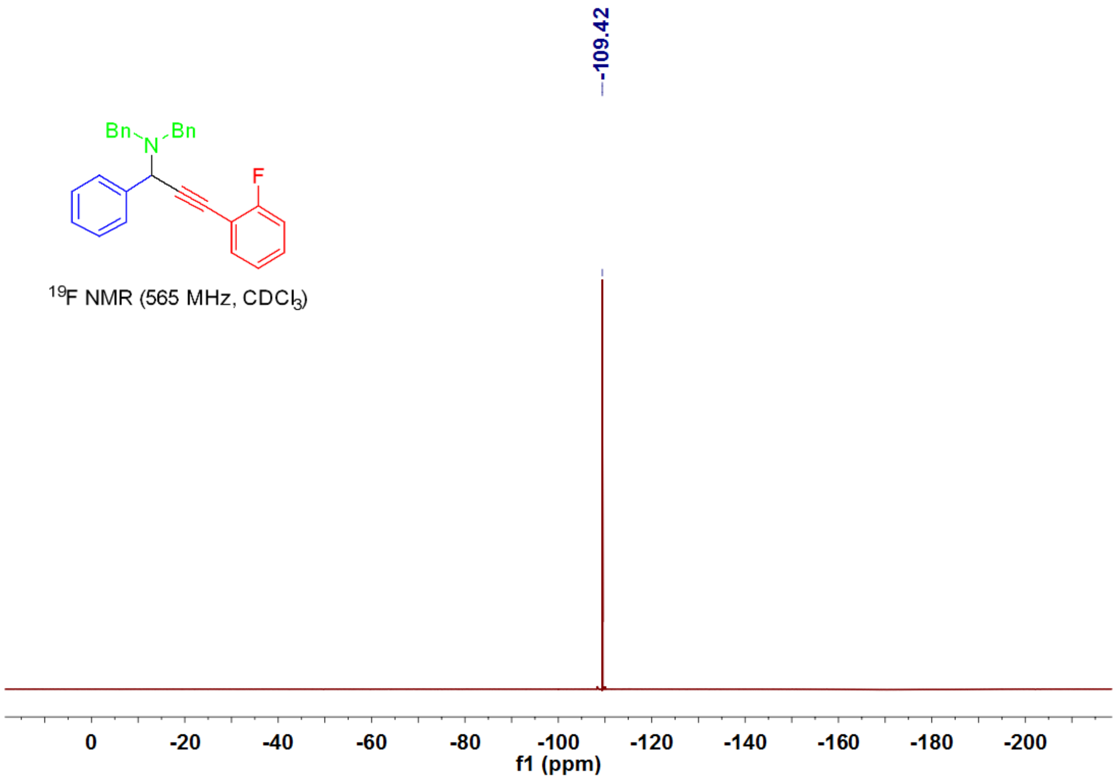

Supplementary Figure 109. **^{19}F NMR** spectrum of compound **4ad**.

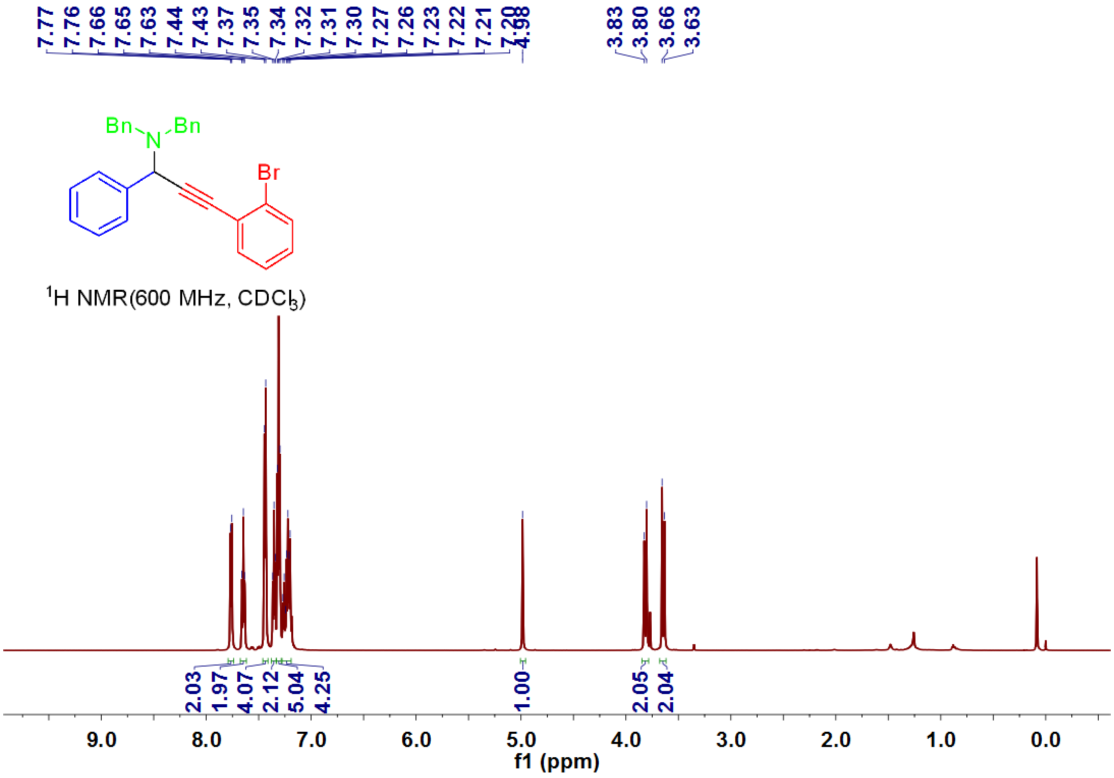

Supplementary Figure 110. ^{1}**H NMR** spectrum of compound **4ae**.

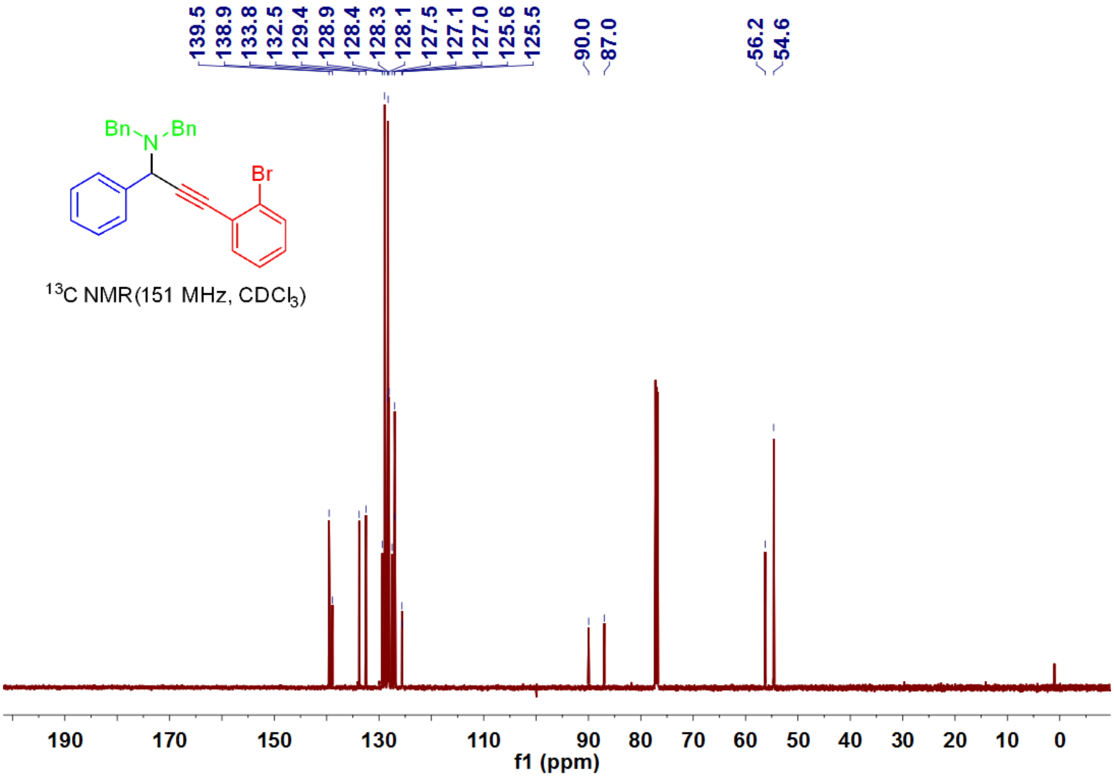

Supplementary Figure 111. ^{13}**C NMR** spectrum of compound **4ae**.

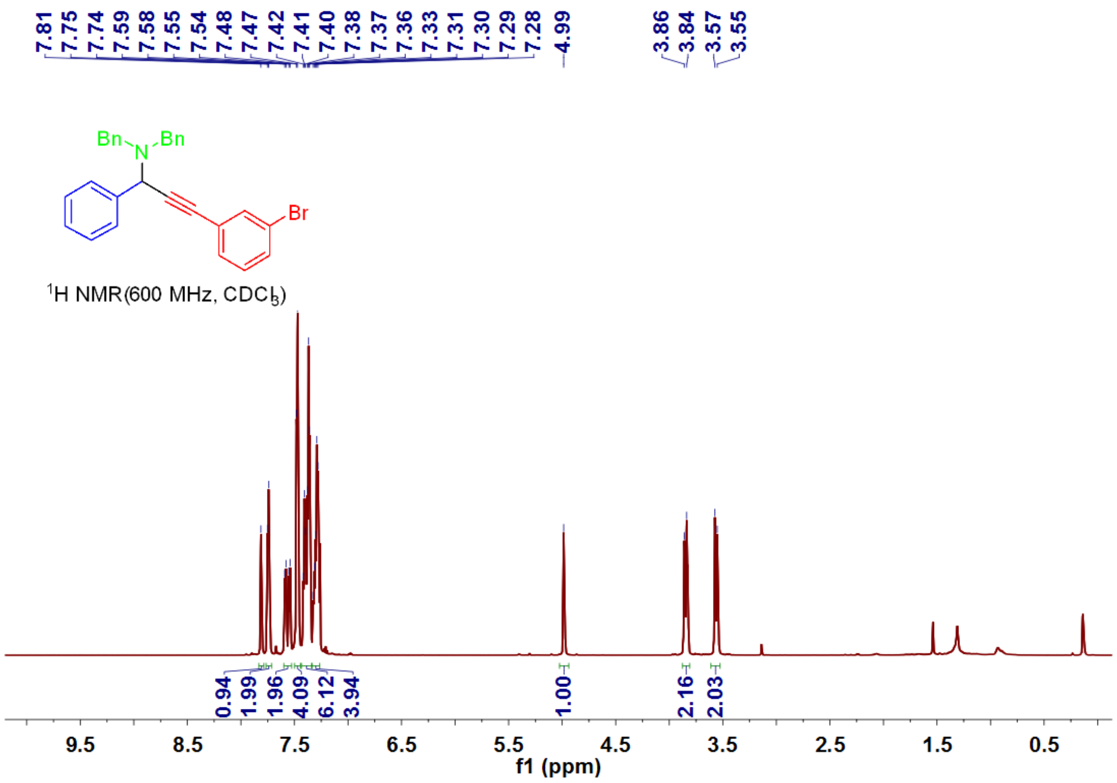

Supplementary Figure 112. ^{1}**H NMR** spectrum of compound **4af**.

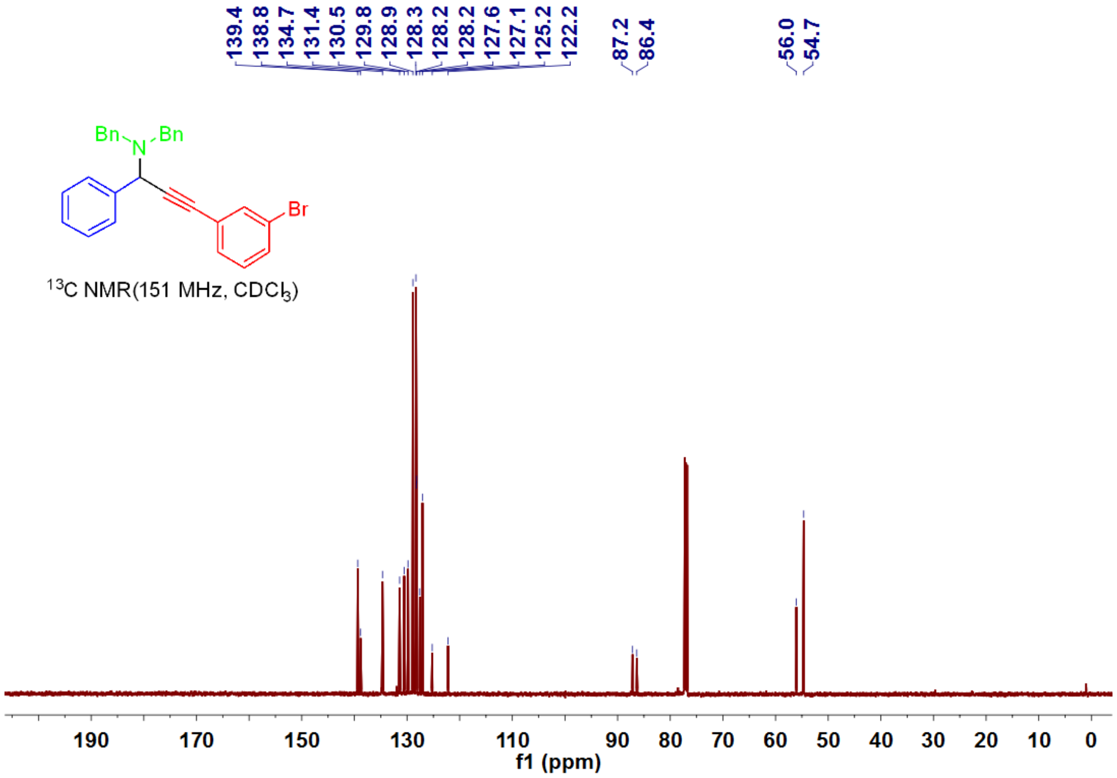

Supplementary Figure 113. ^{13}**C NMR** spectrum of compound **4af**.

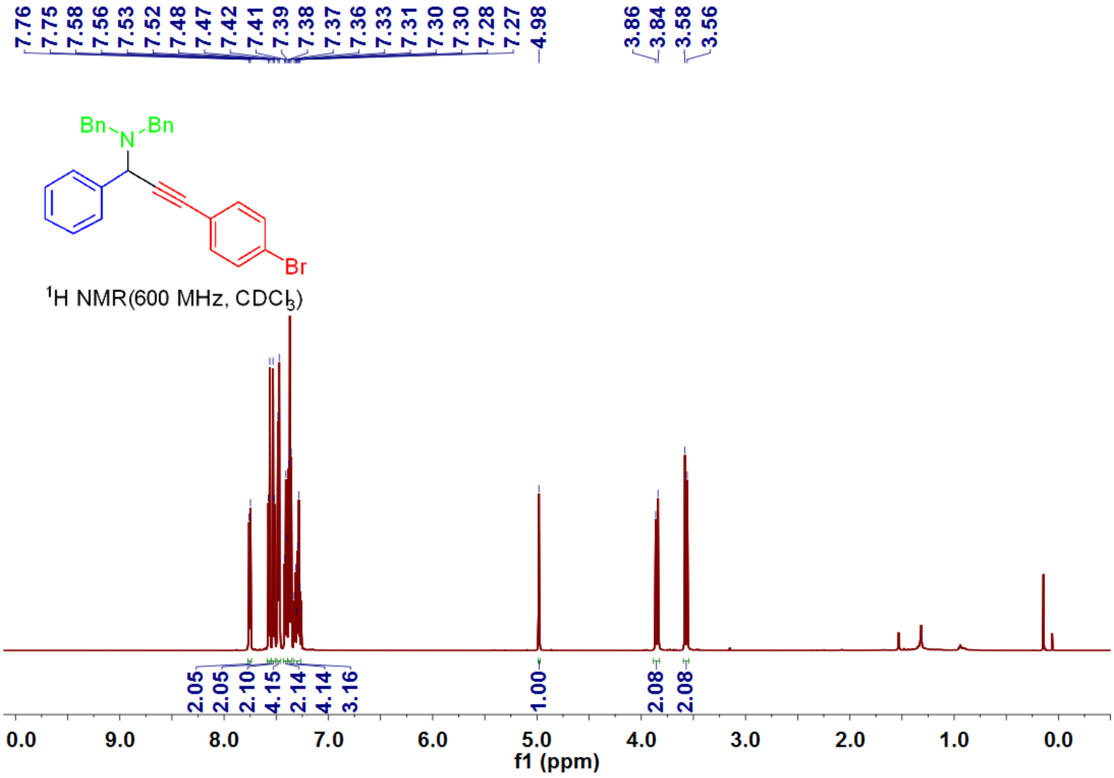

Supplementary Figure 114. **^{1}H NMR** spectrum of compound **4ag**.

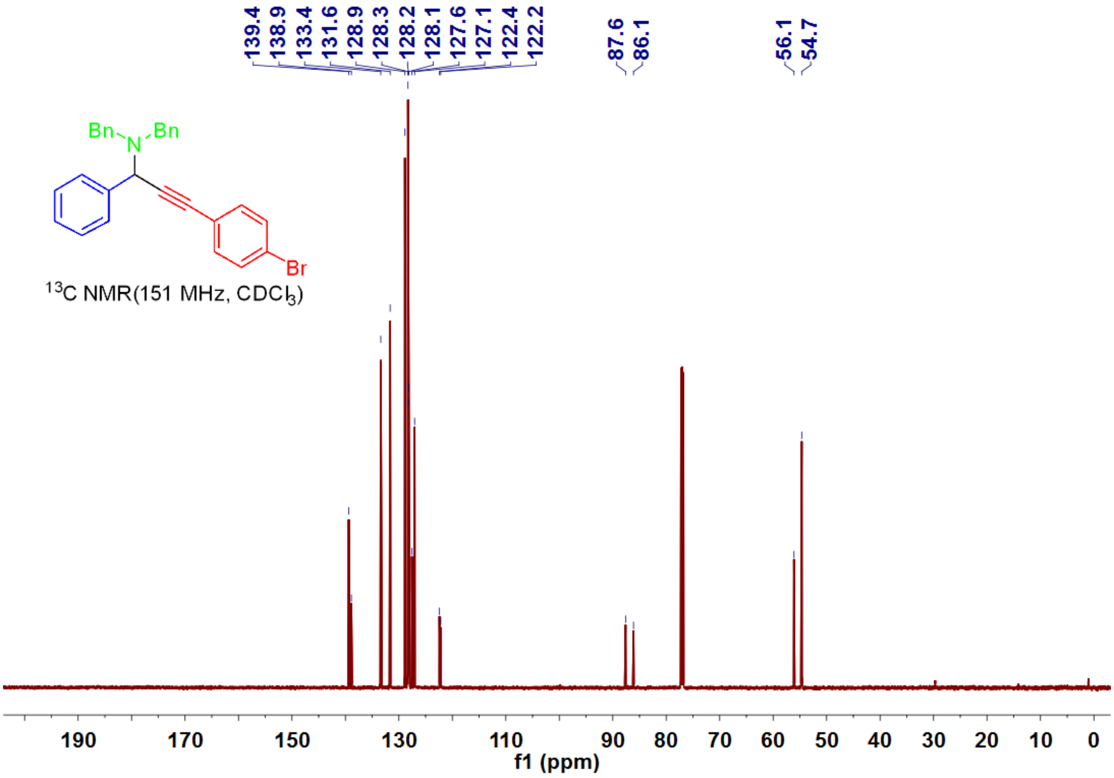

Supplementary Figure 115. **^{13}C NMR** spectrum of compound **4ag**.

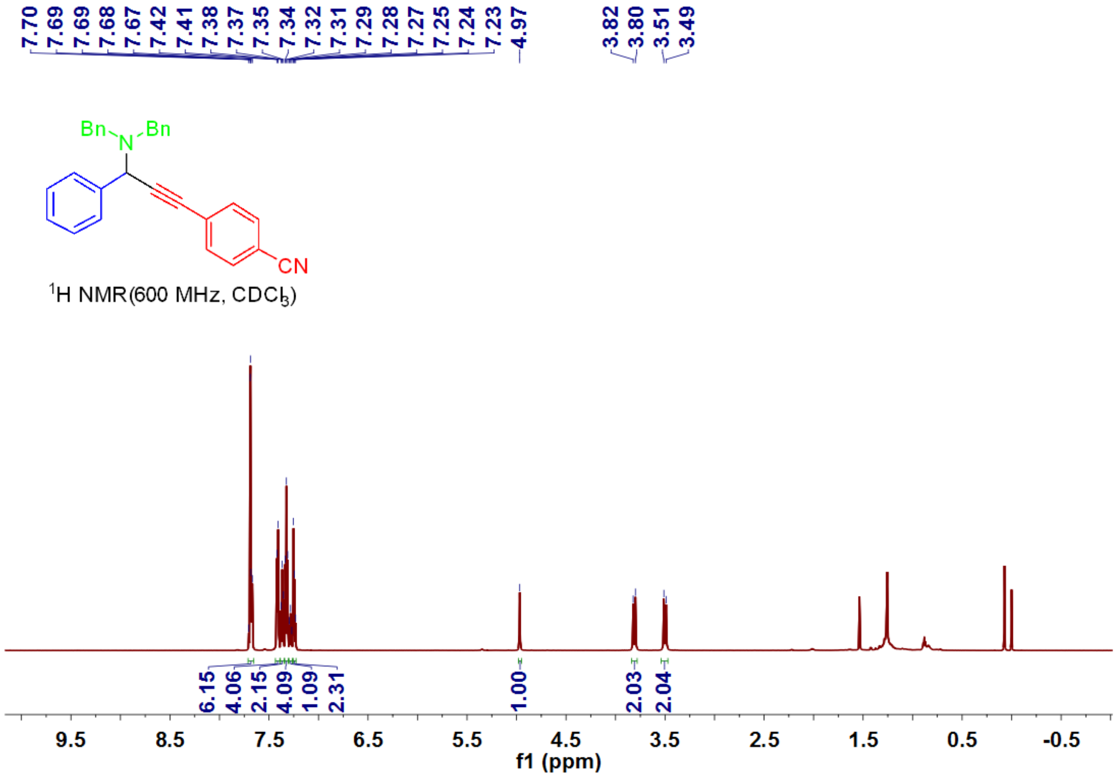

Supplementary Figure 116. **^{1}H NMR** spectrum of compound **4ah**.

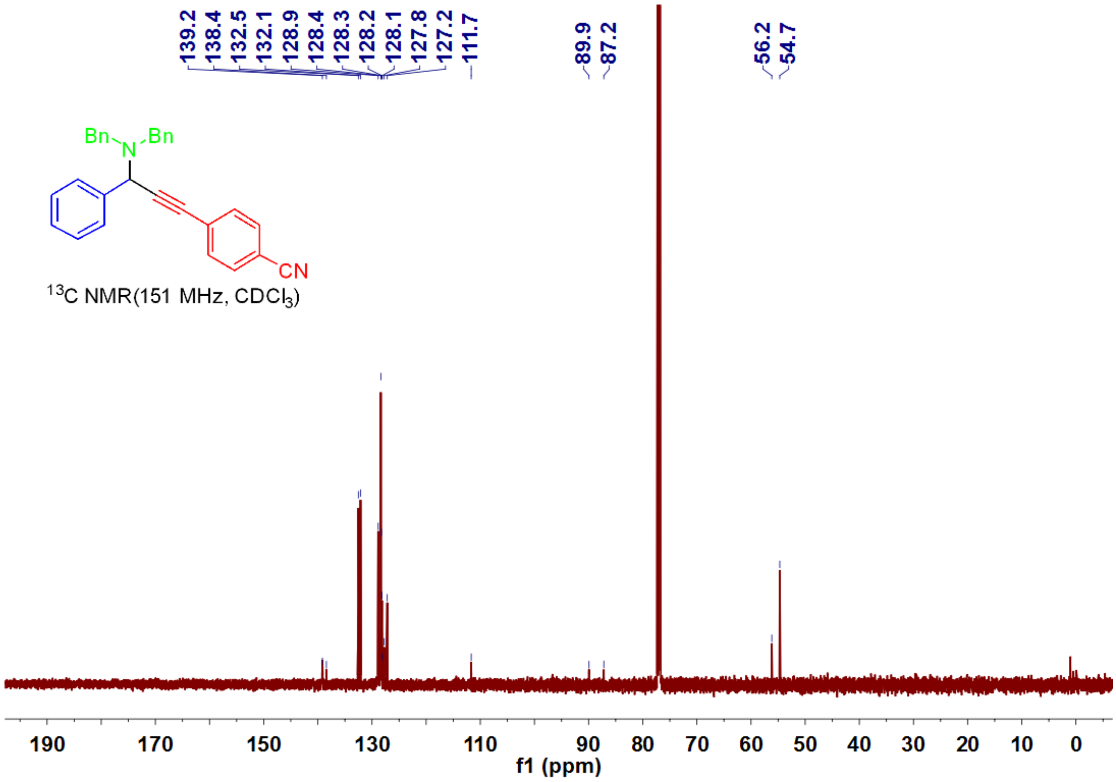

Supplementary Figure 117. **^{13}C NMR** spectrum of compound **4ah**.

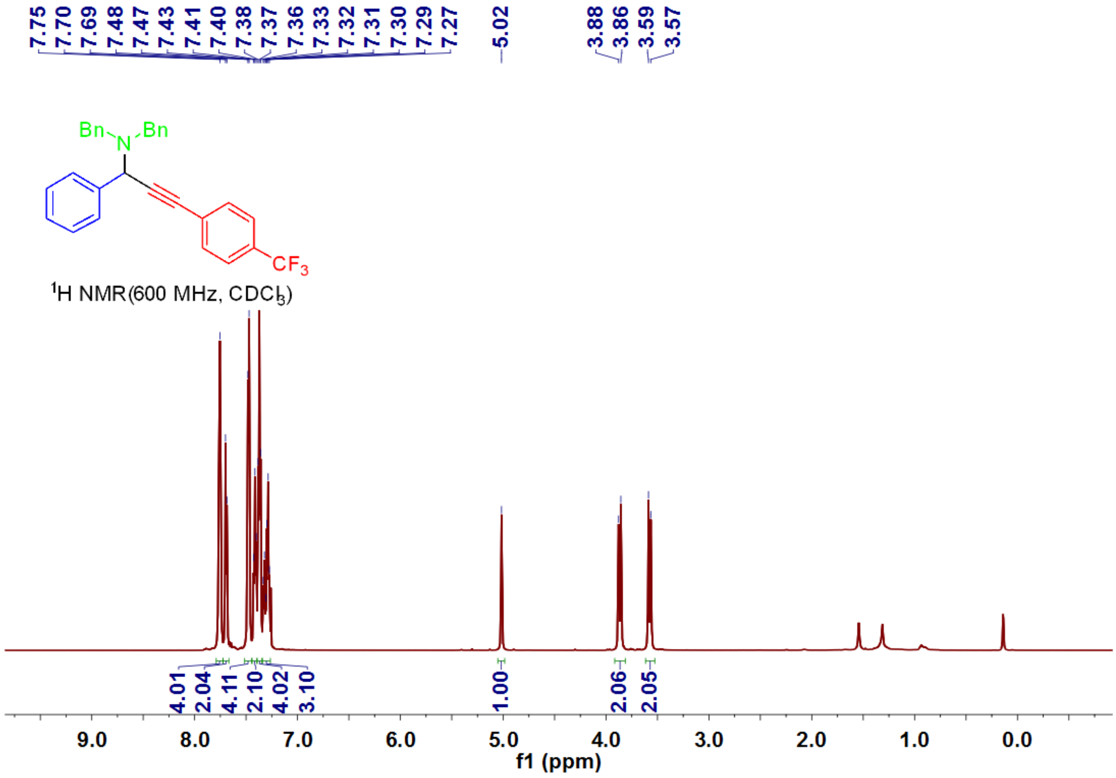

Supplementary Figure 118. ^{1}**H NMR** spectrum of compound **4ai**.

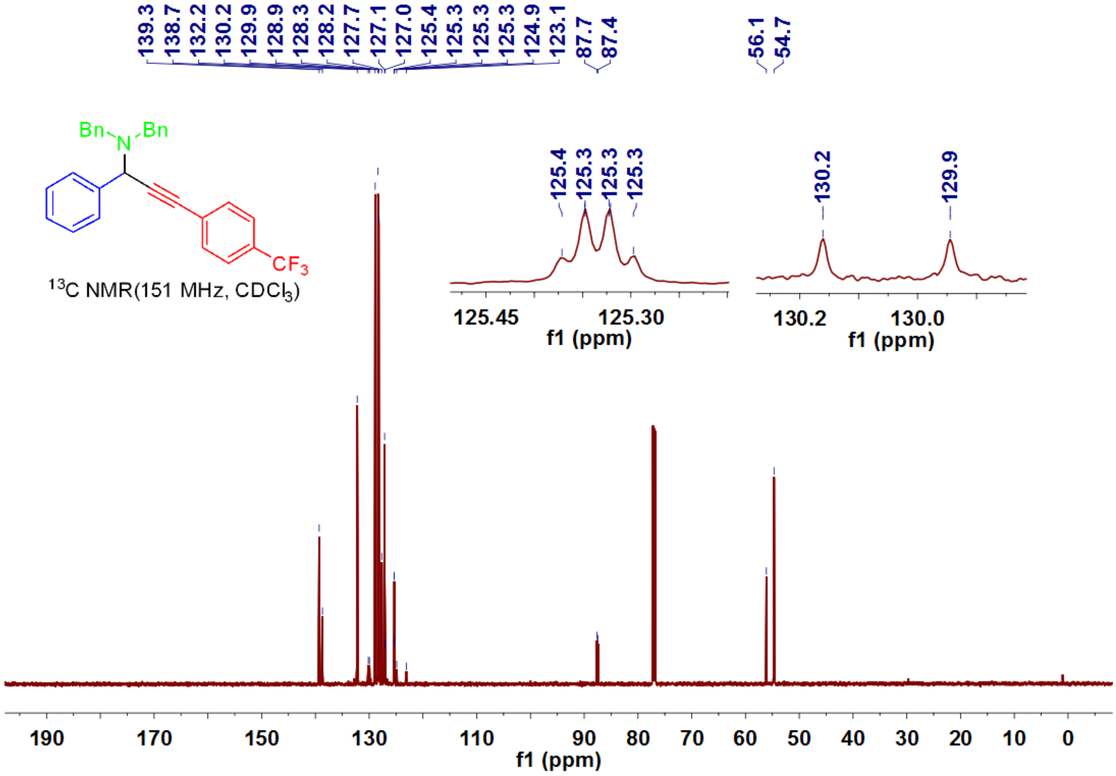

Supplementary Figure 119. ^{13}**C NMR** spectrum of compound **4ai**.

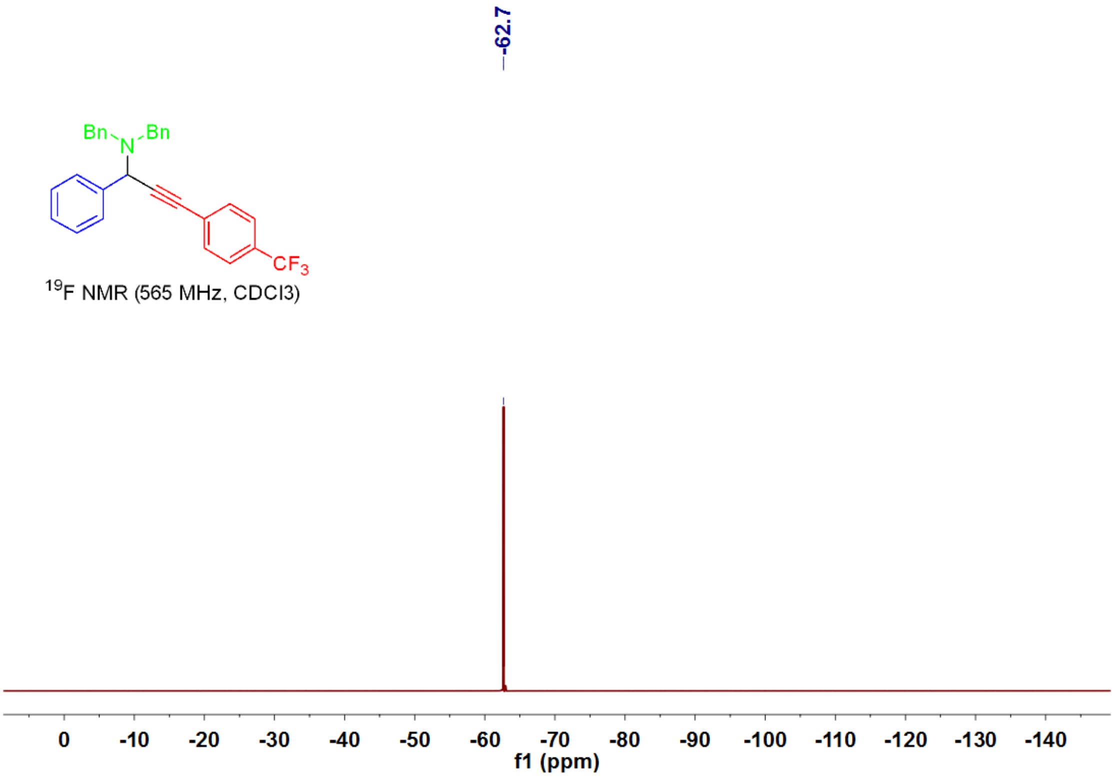

Supplementary Figure 120. ^{19}**F NMR** spectrum of compound **4ai**.

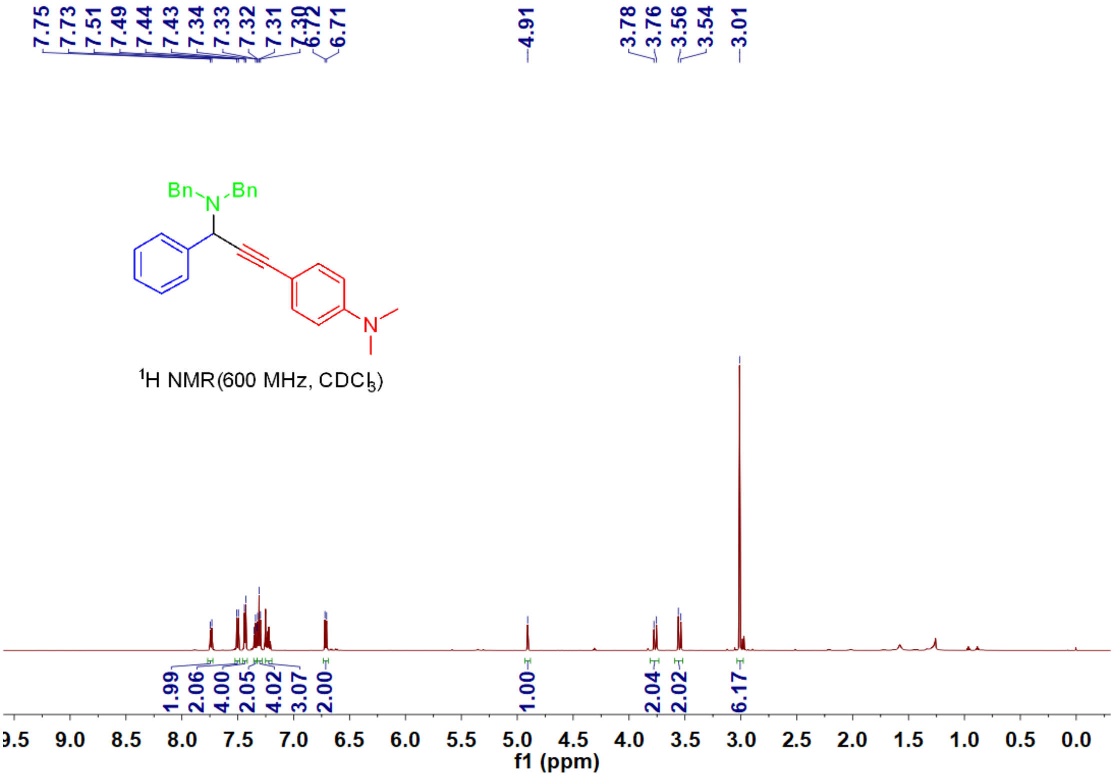

Supplementary Figure 121. ^{1}**H NMR** spectrum of compound **4aj**.

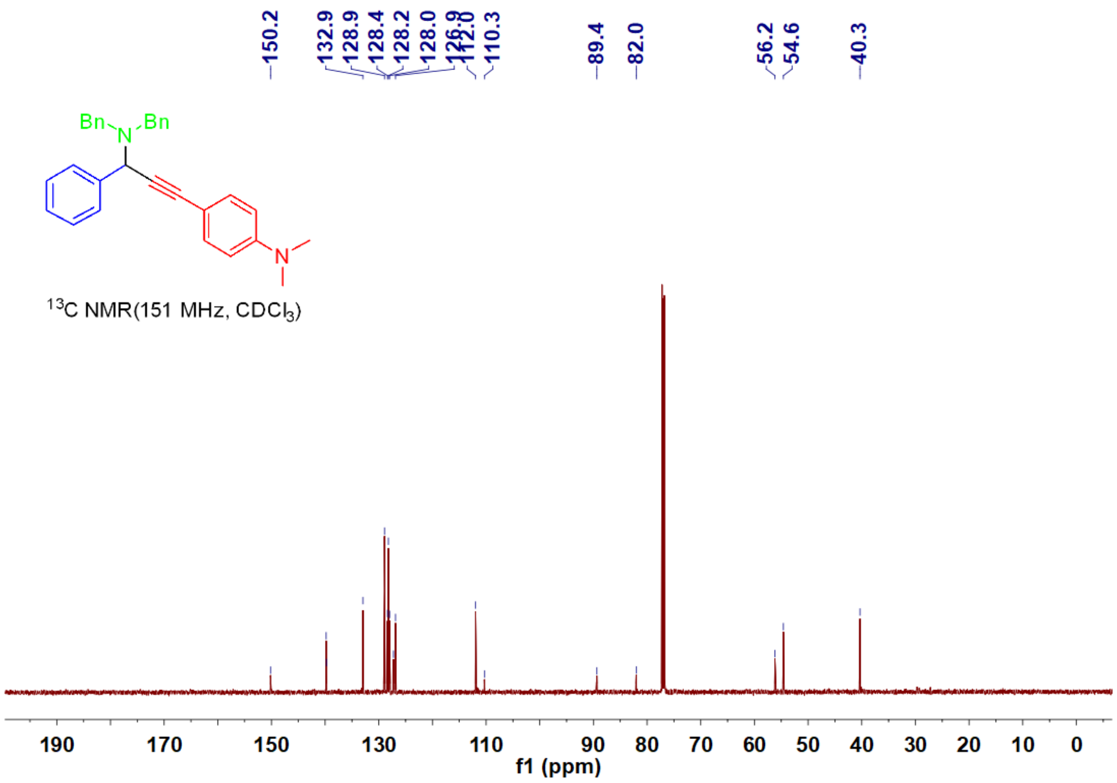

Supplementary Figure 122. ^{13}**C NMR** spectrum of compound **4aj**.

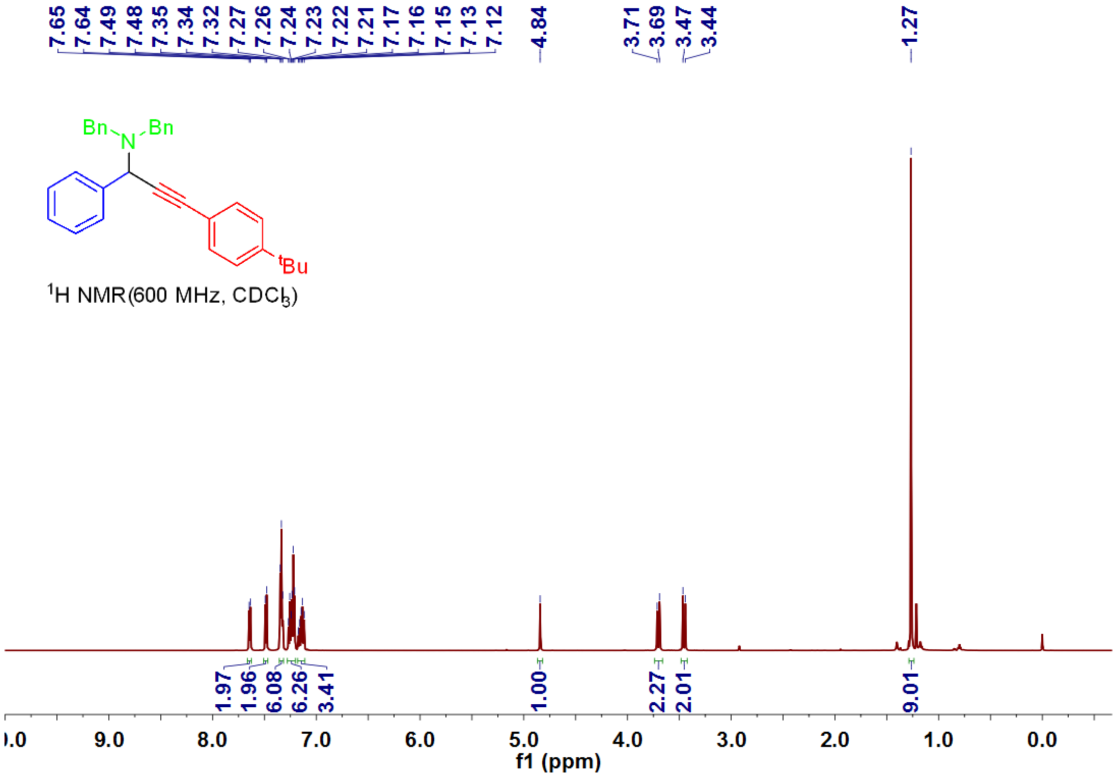

Supplementary Figure 123. ^{1}**H NMR** spectrum of compound **4ak**.

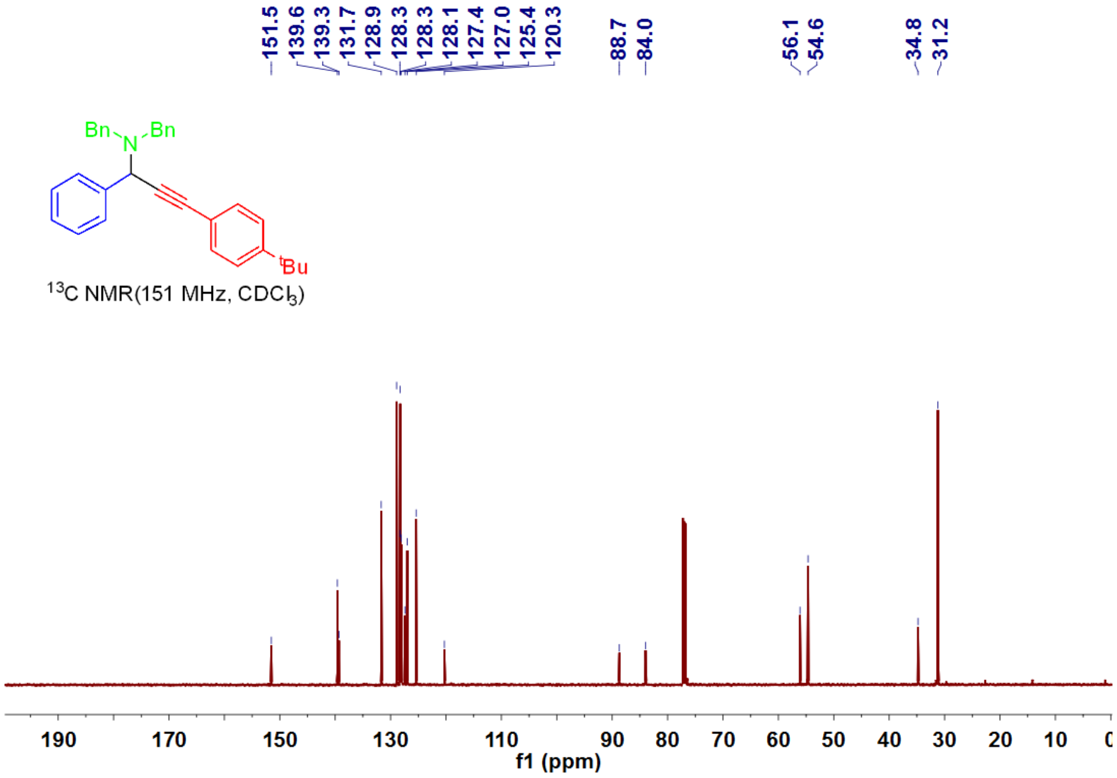

Supplementary Figure 124. ^{13}**C NMR** spectrum of compound **4ak**.

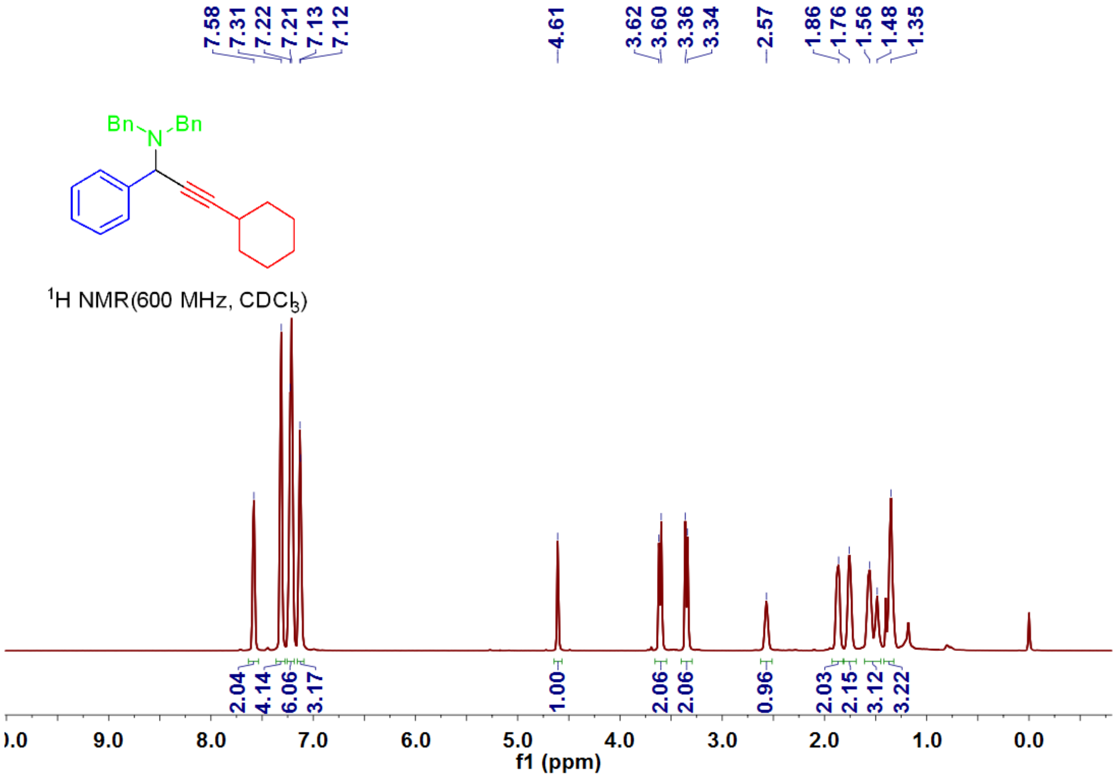

Supplementary Figure 125. ^{1}**H NMR** spectrum of compound **4al**.

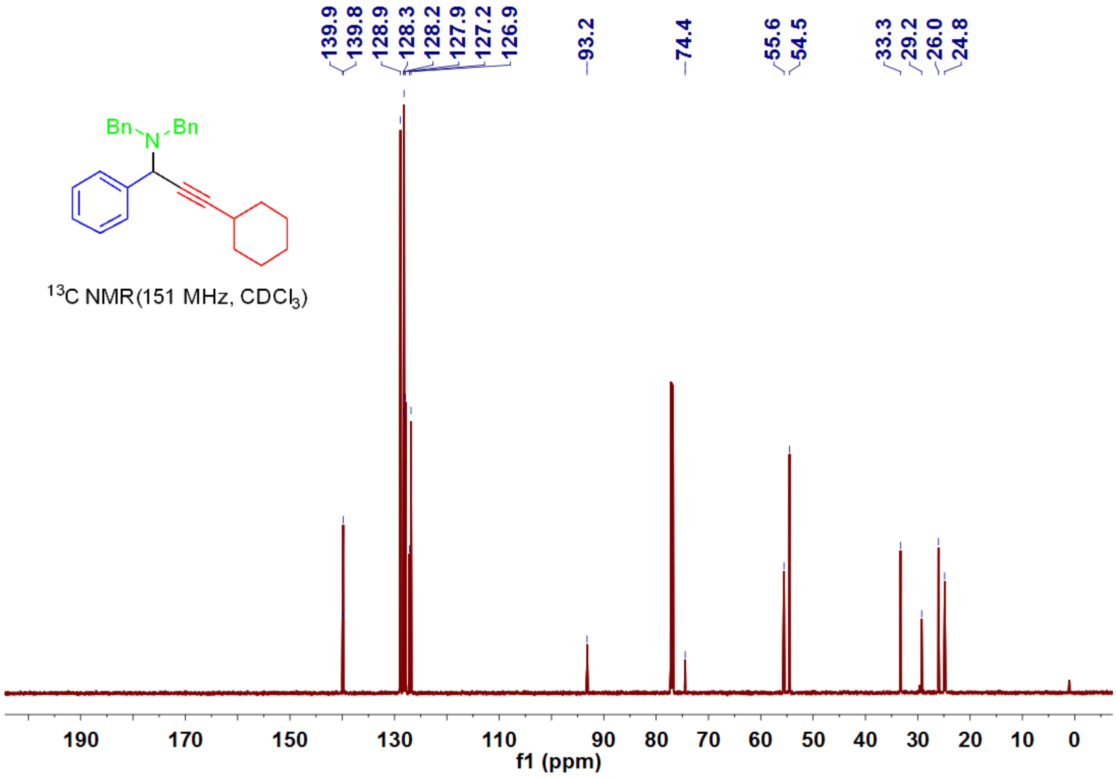

Supplementary Figure 126. **^{13}C NMR** spectrum of compound **4al**.

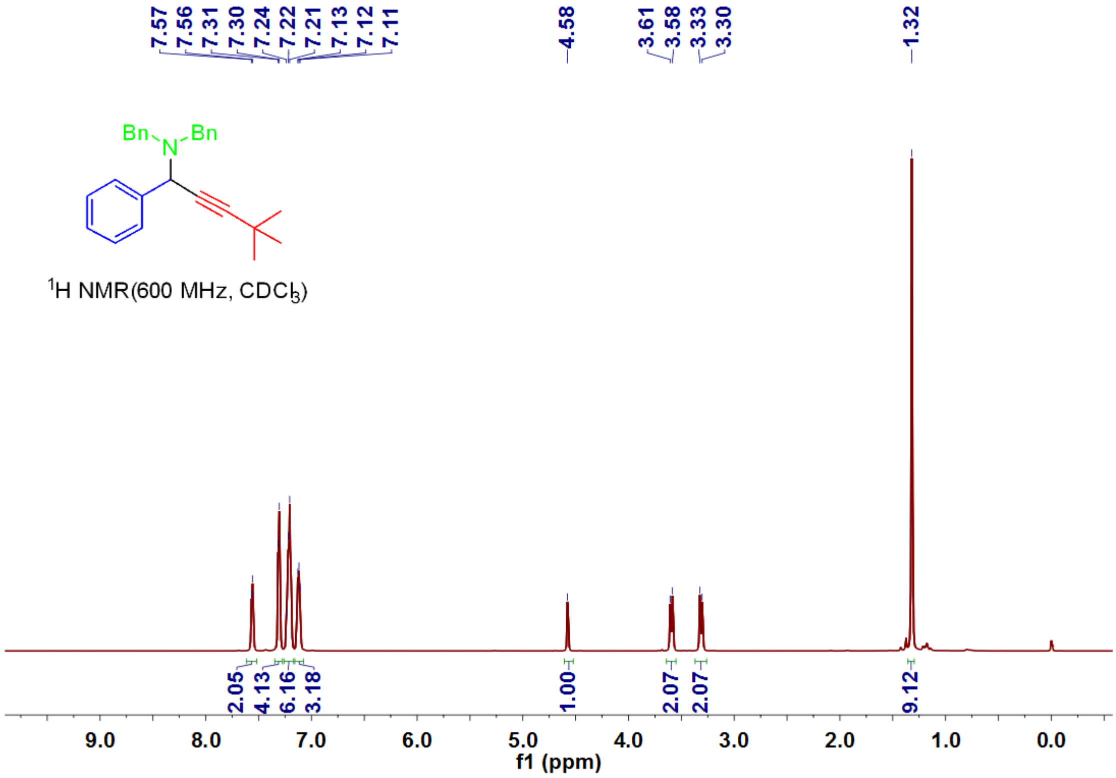

Supplementary Figure 127. **^{1}H NMR** spectrum of compound **4am**.

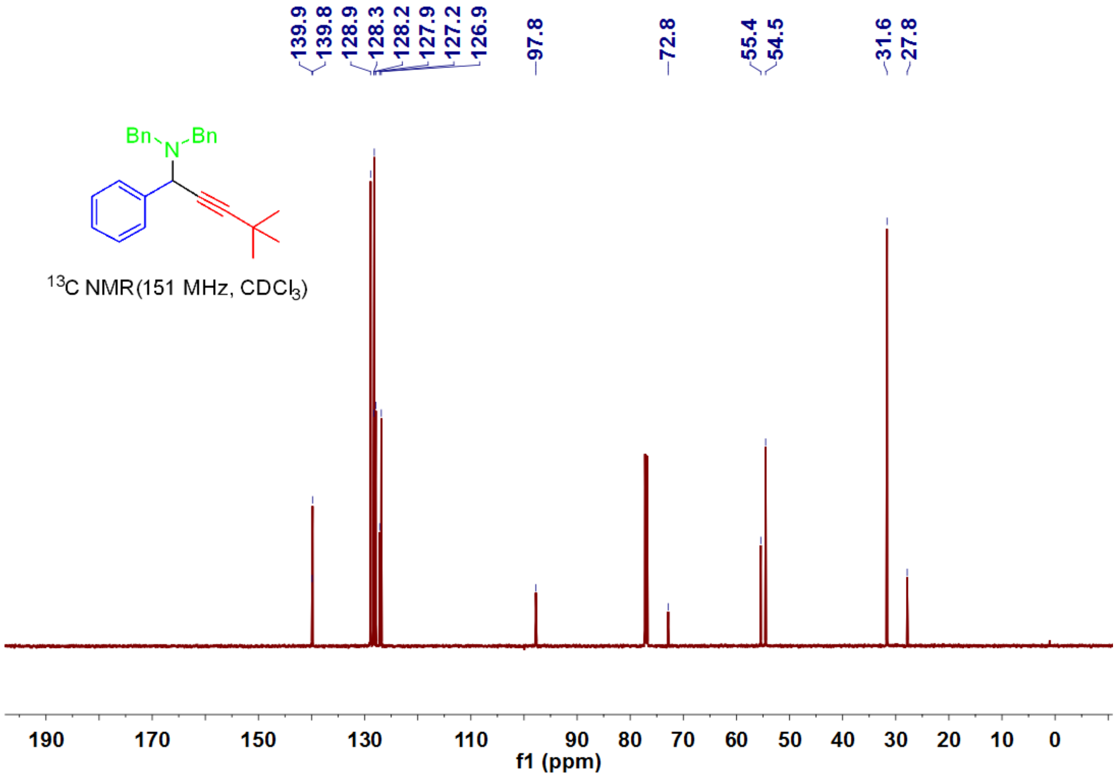

Supplementary Figure 128. ^{13}**C NMR** spectrum of compound **4am**.

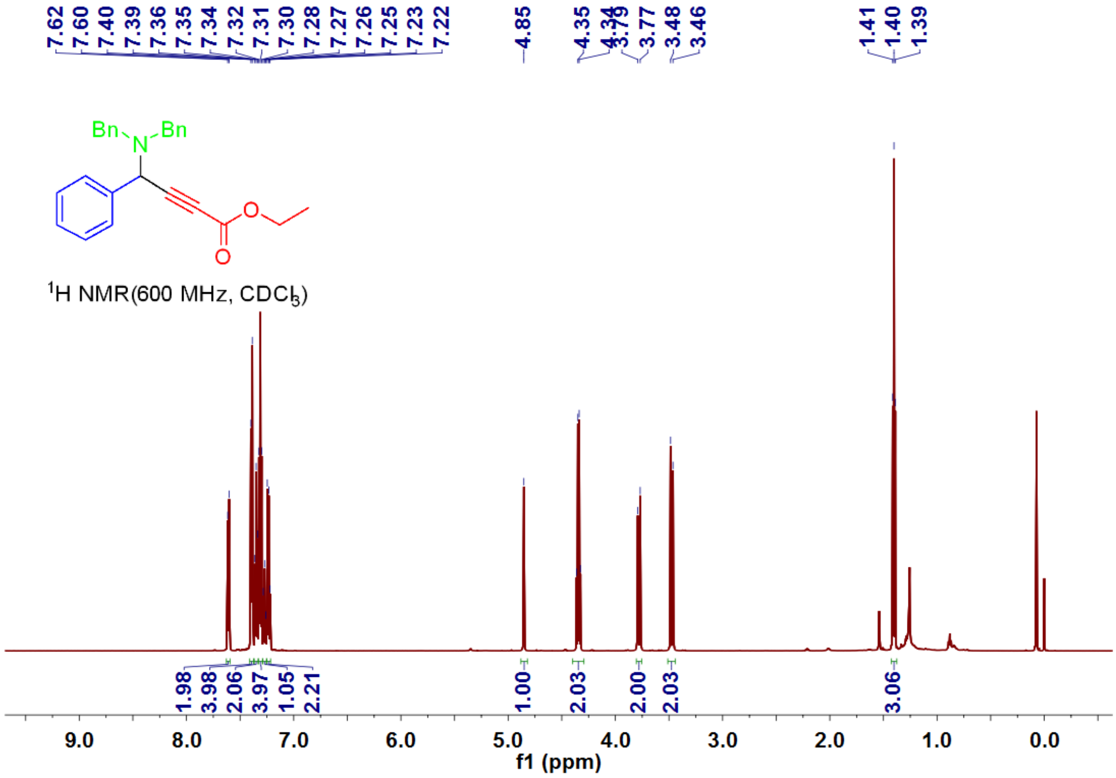

Supplementary Figure 129. ^{1}**H NMR** spectrum of compound **4an**.

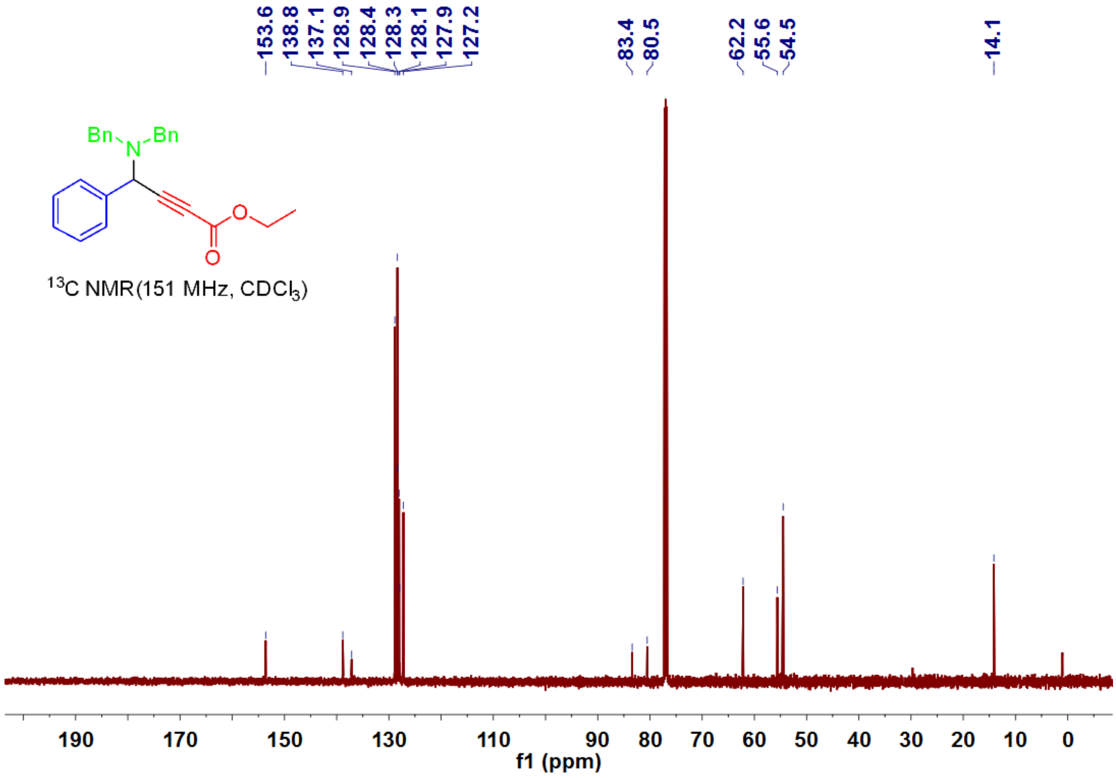

Supplementary Figure 130. ^{13}**C NMR** spectrum of compound **4an**.

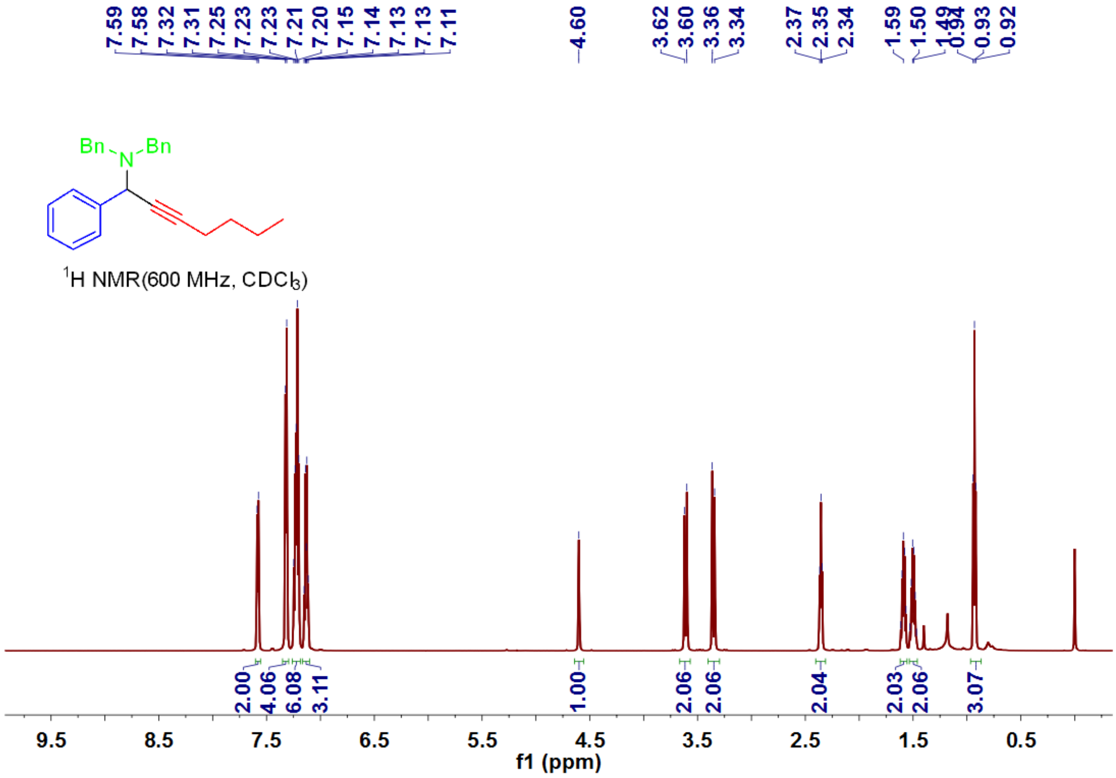

Supplementary Figure 131. ^{1}**H NMR** spectrum of compound **4ao**.

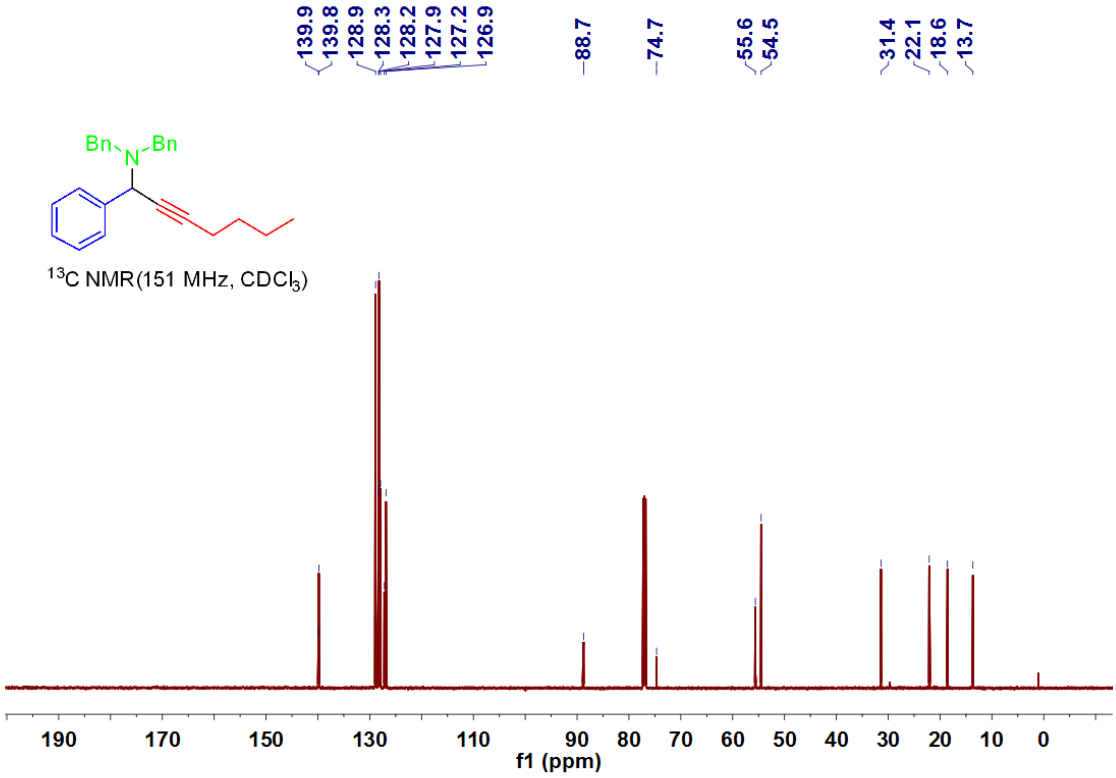

Supplementary Figure 132. ^{13}**C NMR** spectrum of compound **4ao**.

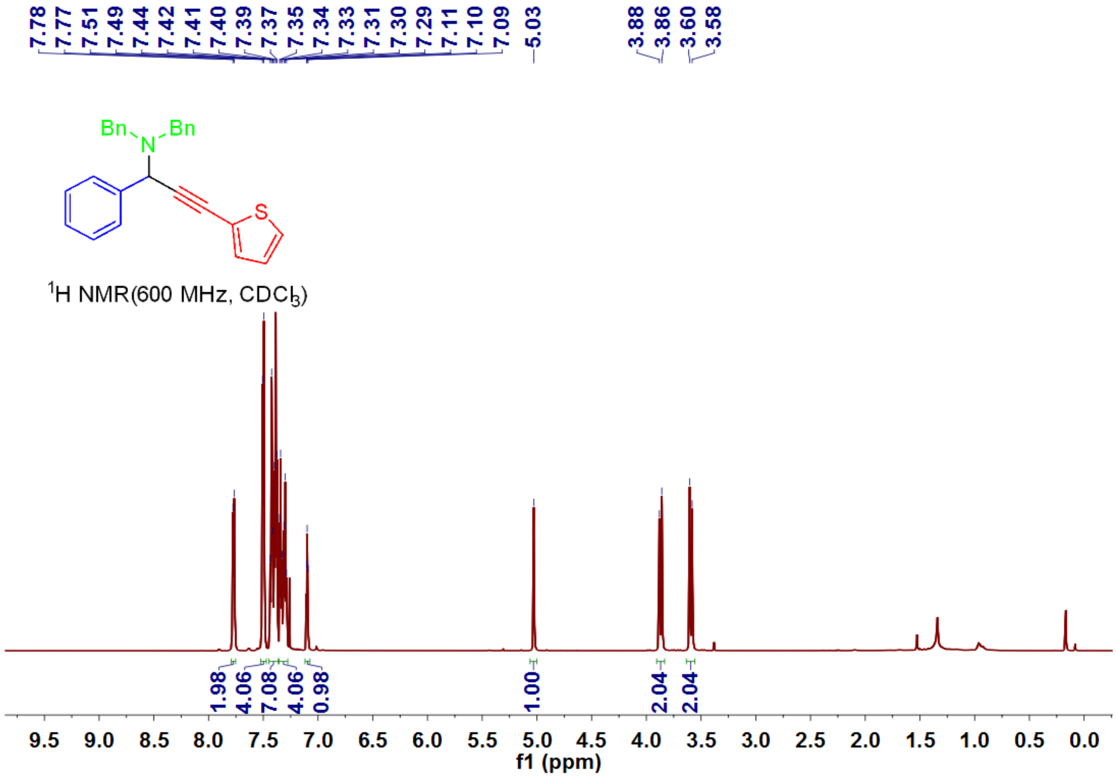

Supplementary Figure 133. ^{1}**H NMR** spectrum of compound **4ap**.

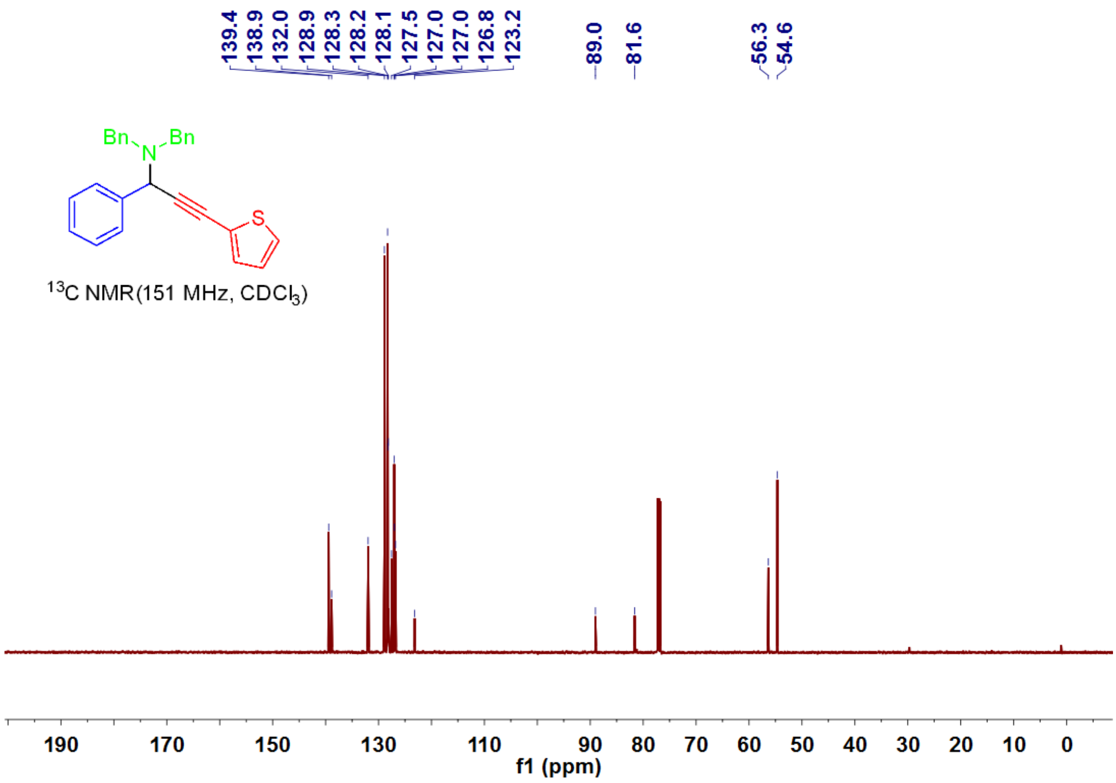

Supplementary Figure 134. ^{13}**C NMR** spectrum of compound **4ap**.

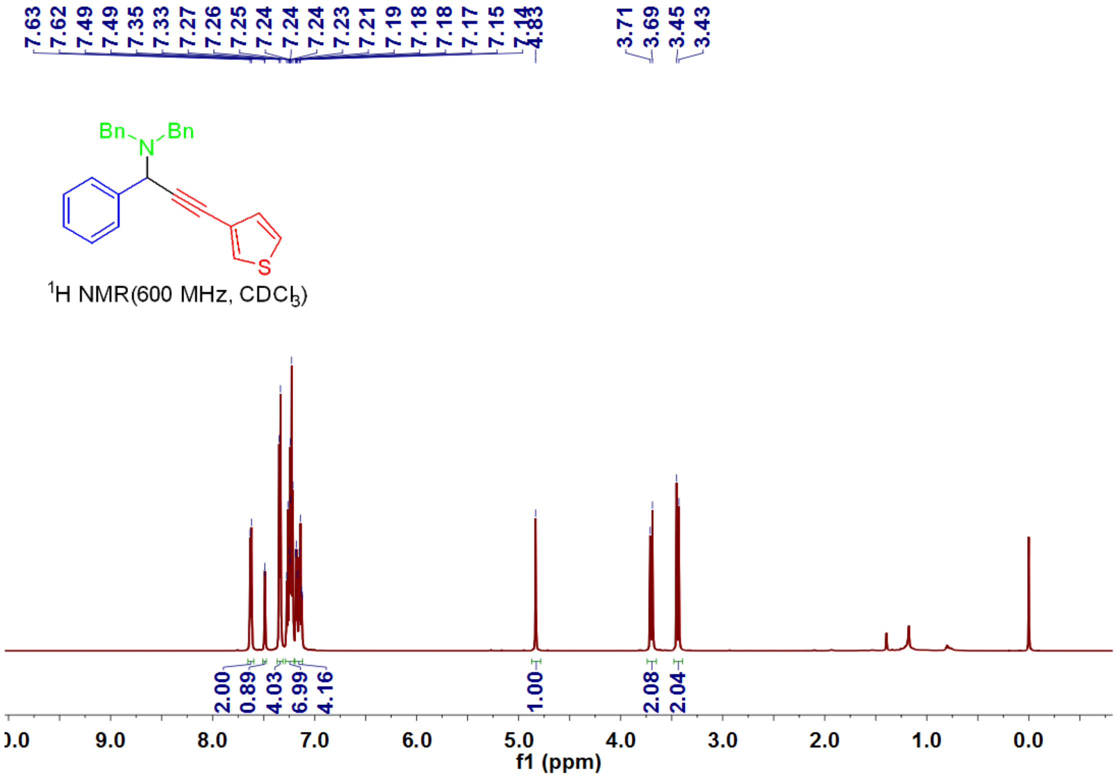

Supplementary Figure 135. ^{1}**H NMR** spectrum of compound **4aq**.

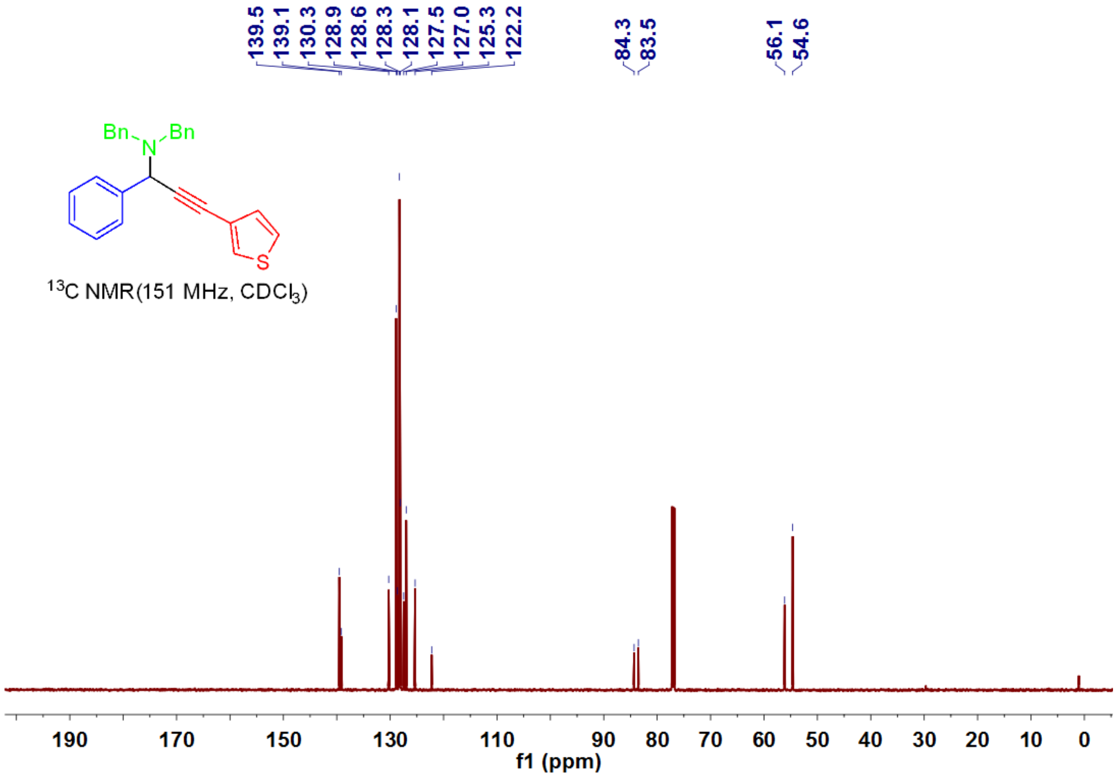

Supplementary Figure 136. ^{13}C NMR spectrum of compound **4aq**.

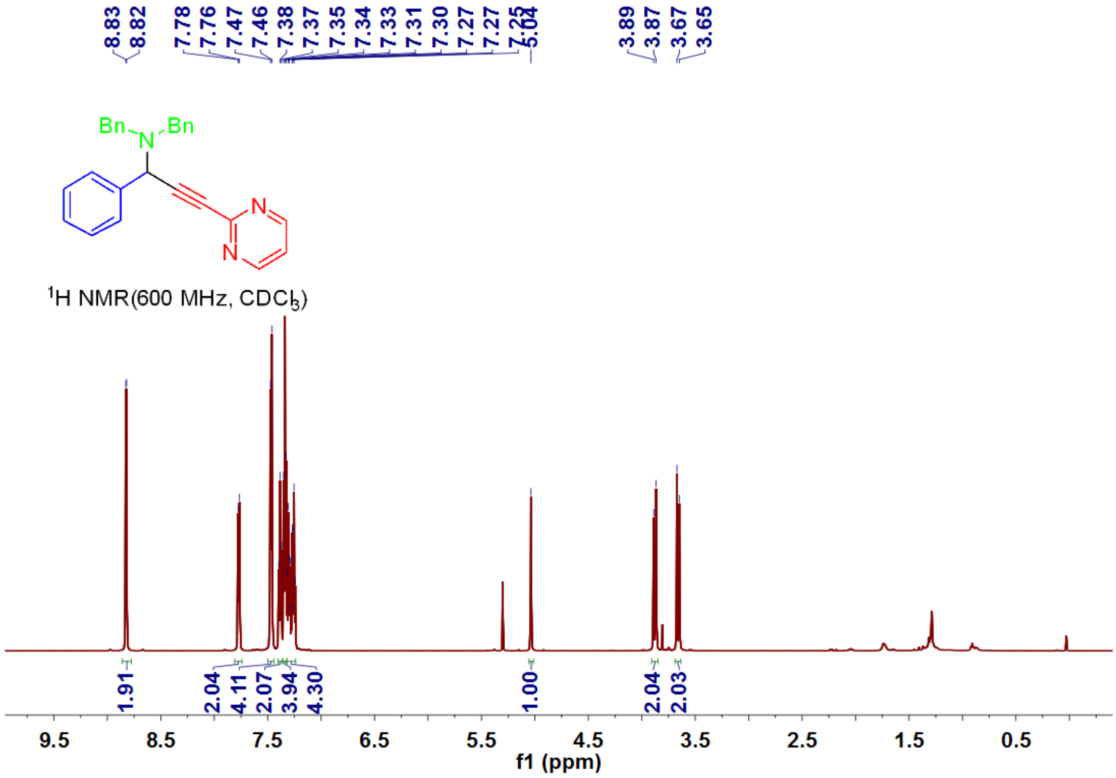

Supplementary Figure 137. ^{1}H NMR spectrum of compound **4ar**.

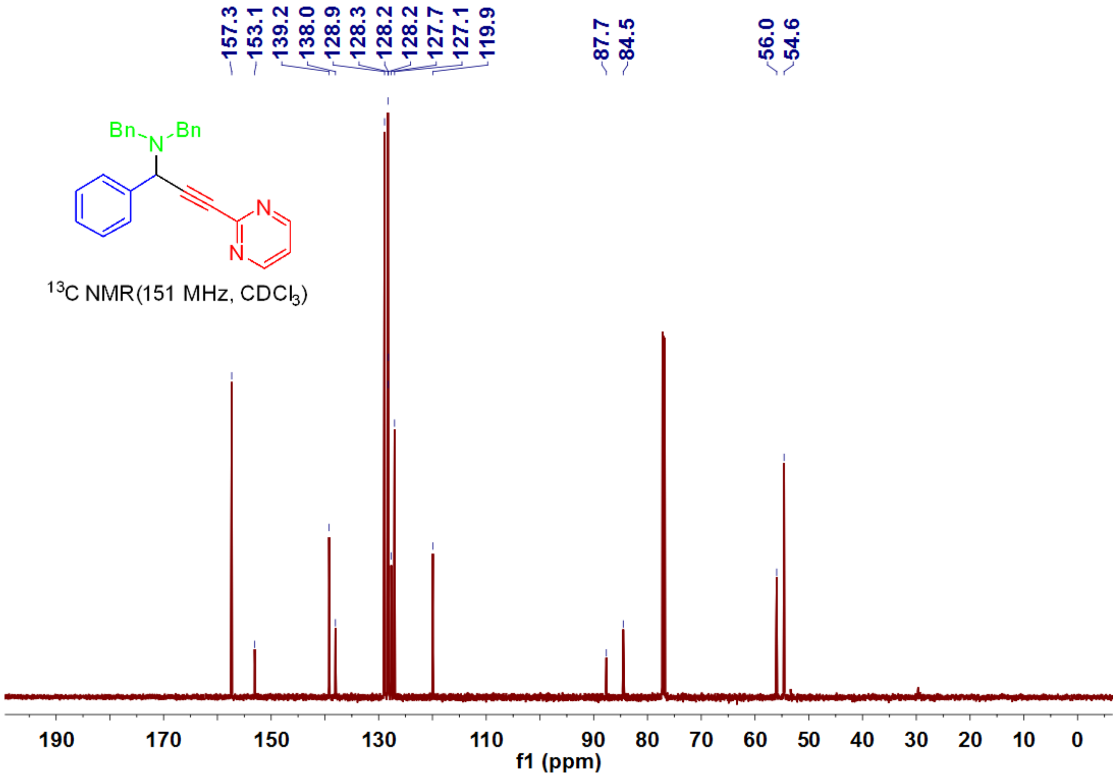

Supplementary Figure 138. ^{13}**C NMR** spectrum of compound **4ar**.

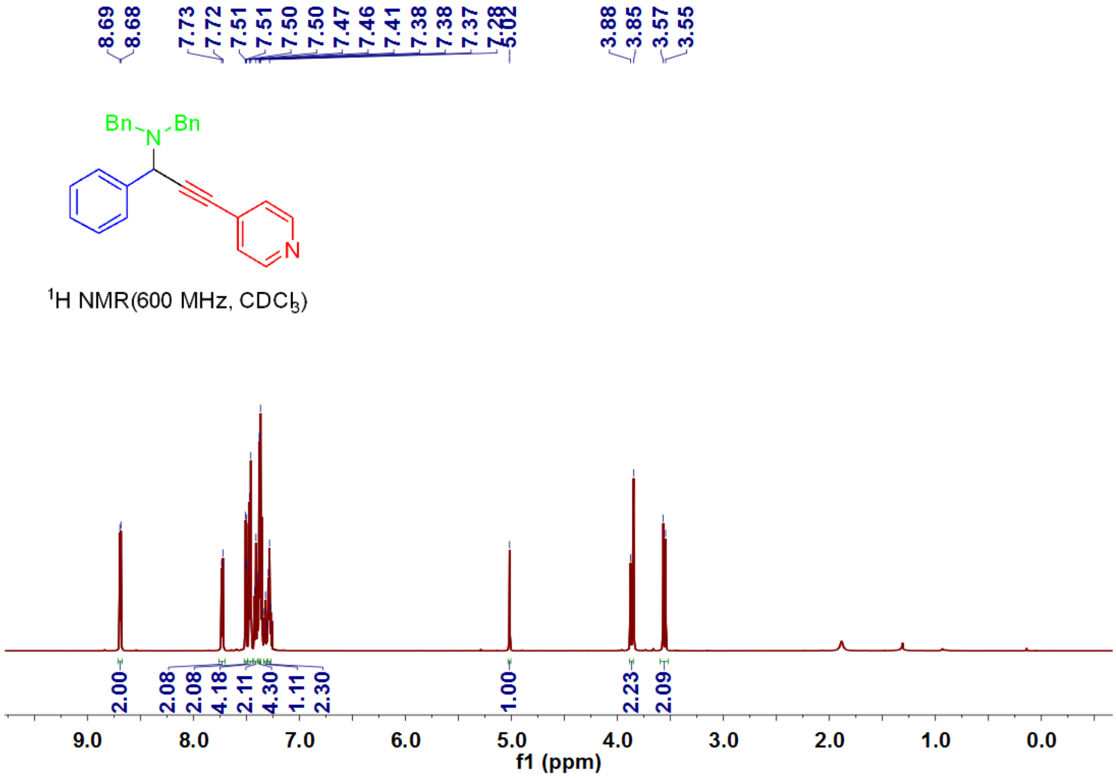

Supplementary Figure 139. ^{1}**H NMR** spectrum of compound **4as**.

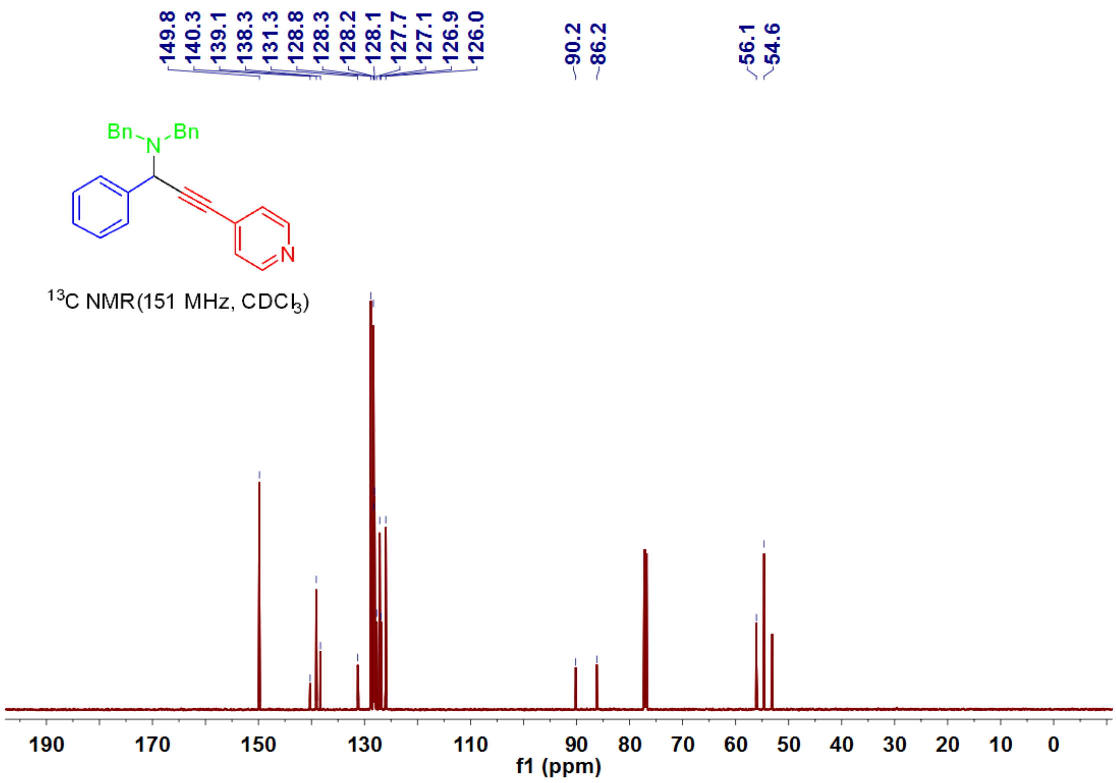

Supplementary Figure 140. **^{13}C NMR** spectrum of compound **4as**.

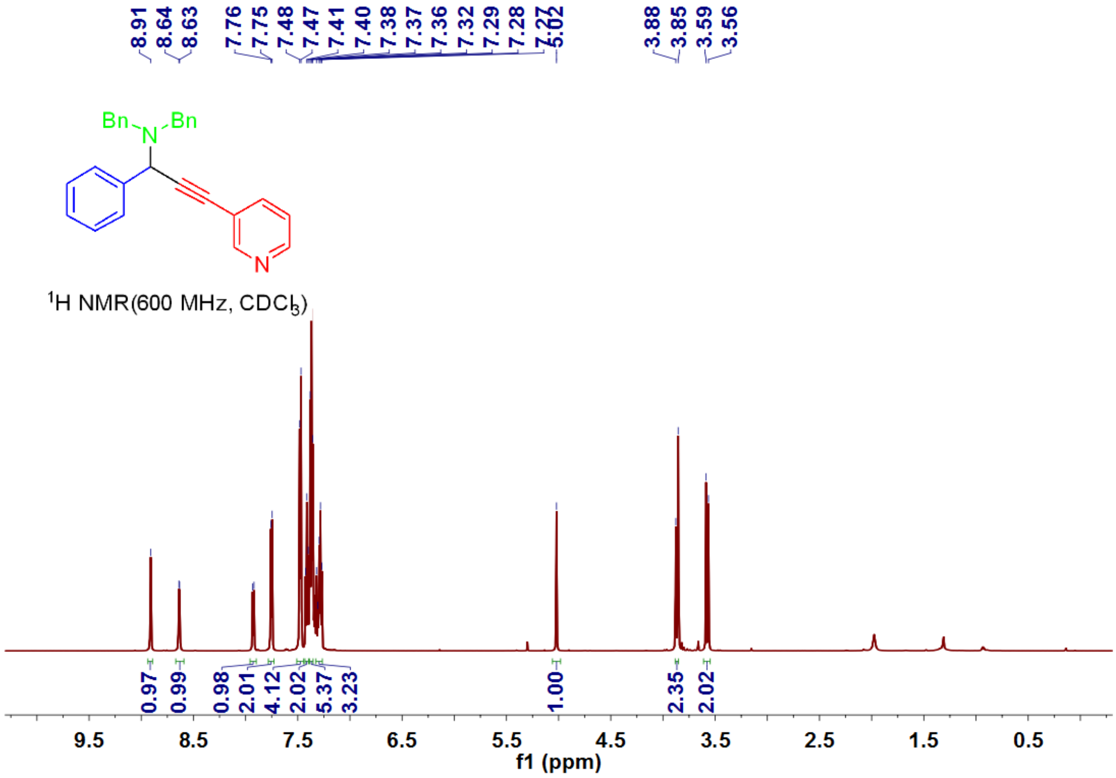

Supplementary Figure 141. **^{1}H NMR** spectrum of compound **4at**.

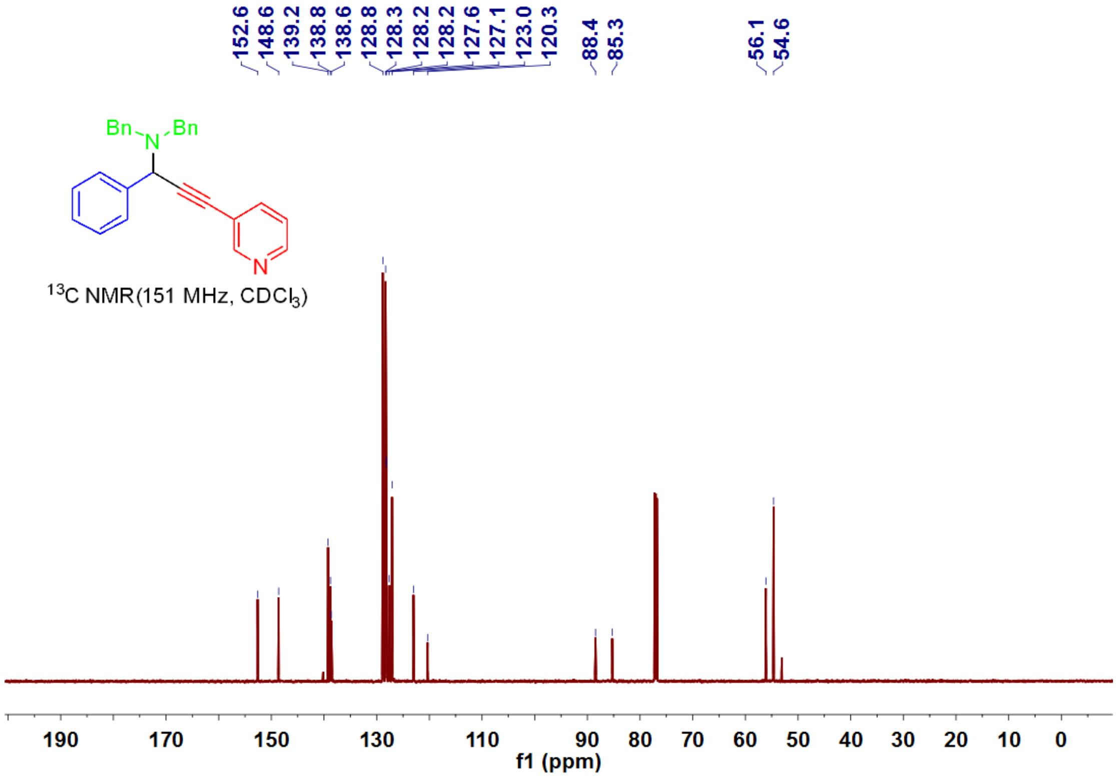

Supplementary Figure 142. ^{13}**C NMR** spectrum of compound **4at**.

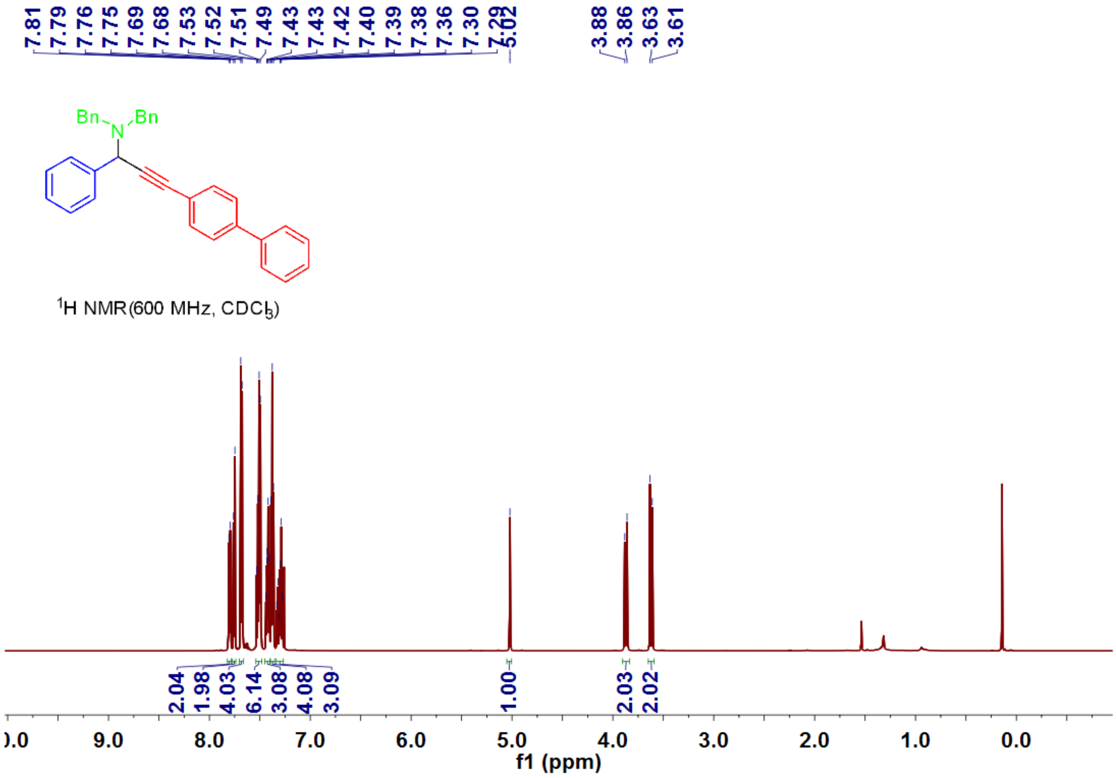

Supplementary Figure 143. ^{1}**H NMR** spectrum of compound **4au**.

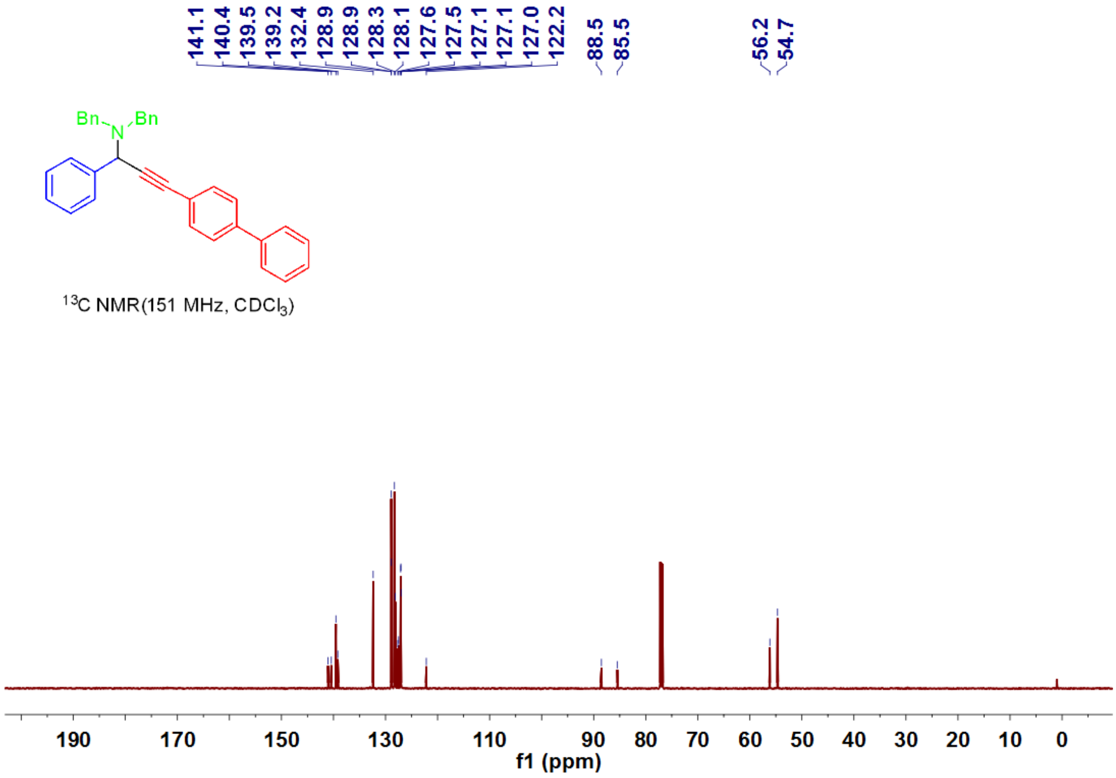

Supplementary Figure 144. ^{13}**C NMR** spectrum of compound **4au**.

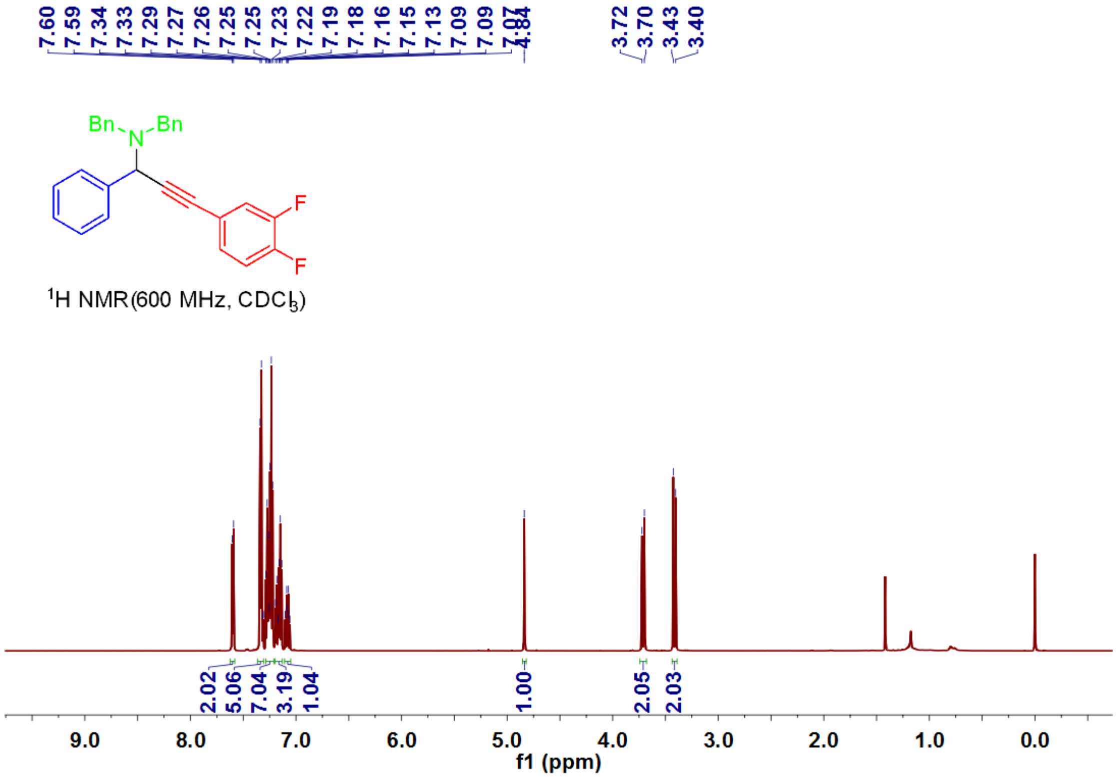

Supplementary Figure 145. ^{1}**H NMR** spectrum of compound **4av**.

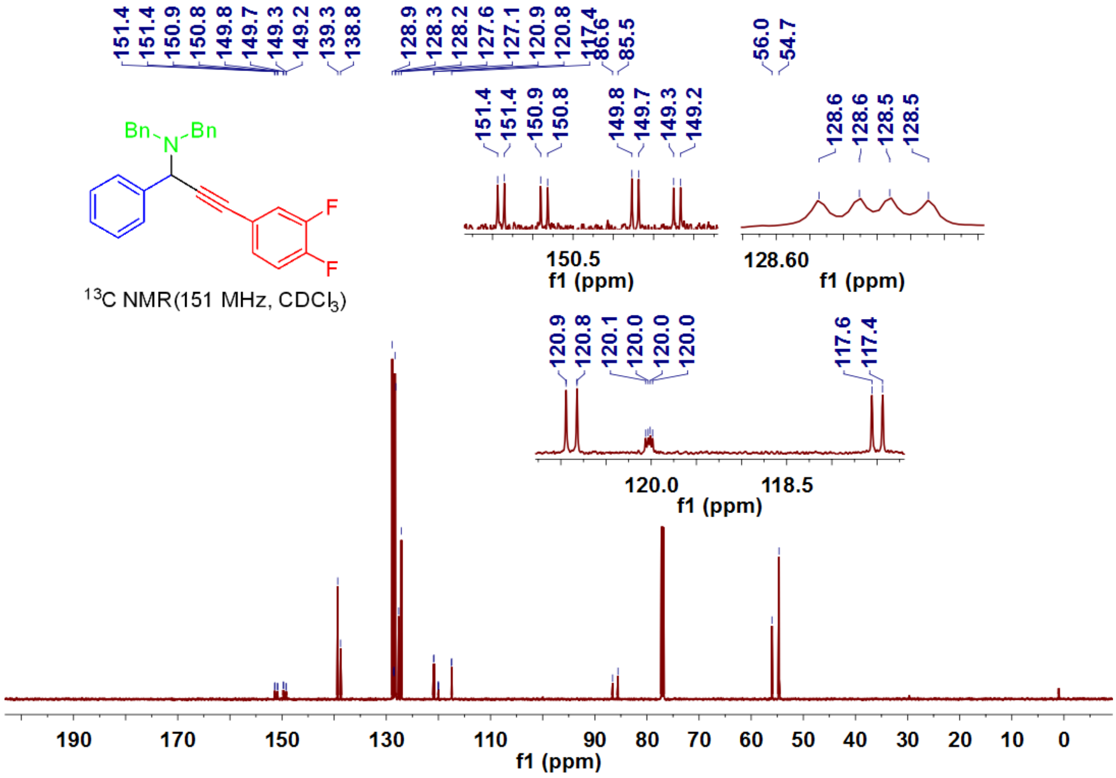

Supplementary Figure 146. ^{13}**C NMR** spectrum of compound **4av**.

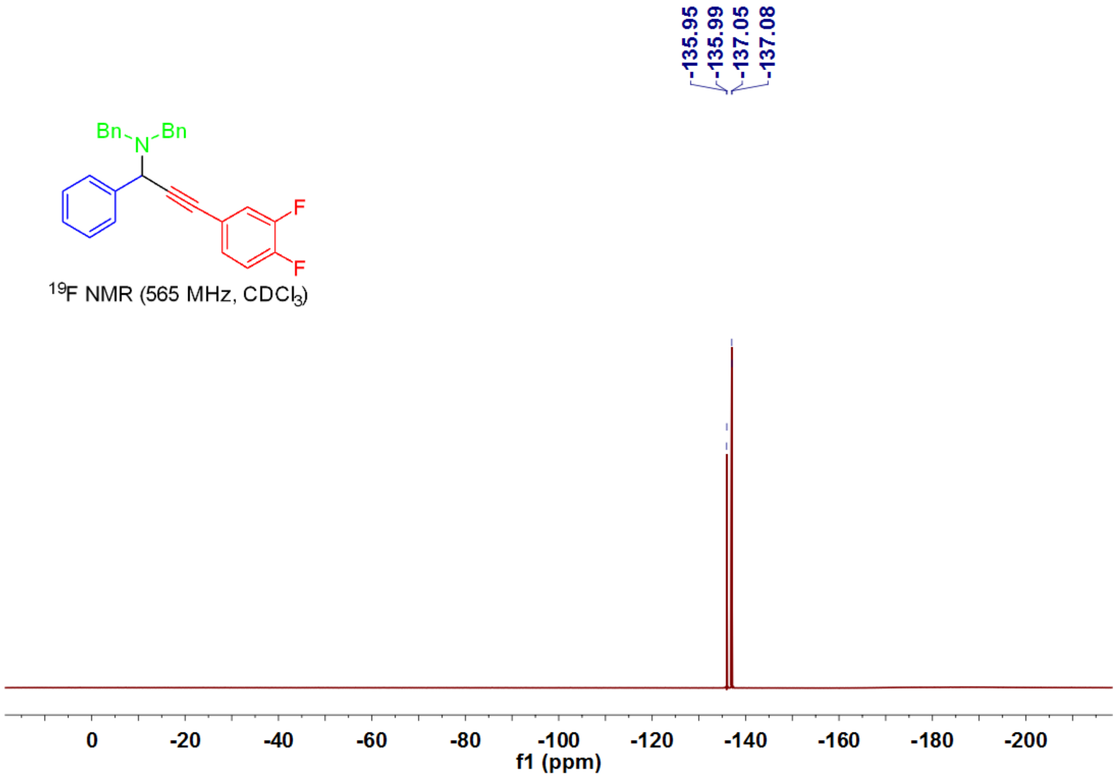

Supplementary Figure 147. ^{19}**F NMR** spectrum of compound **4av**.

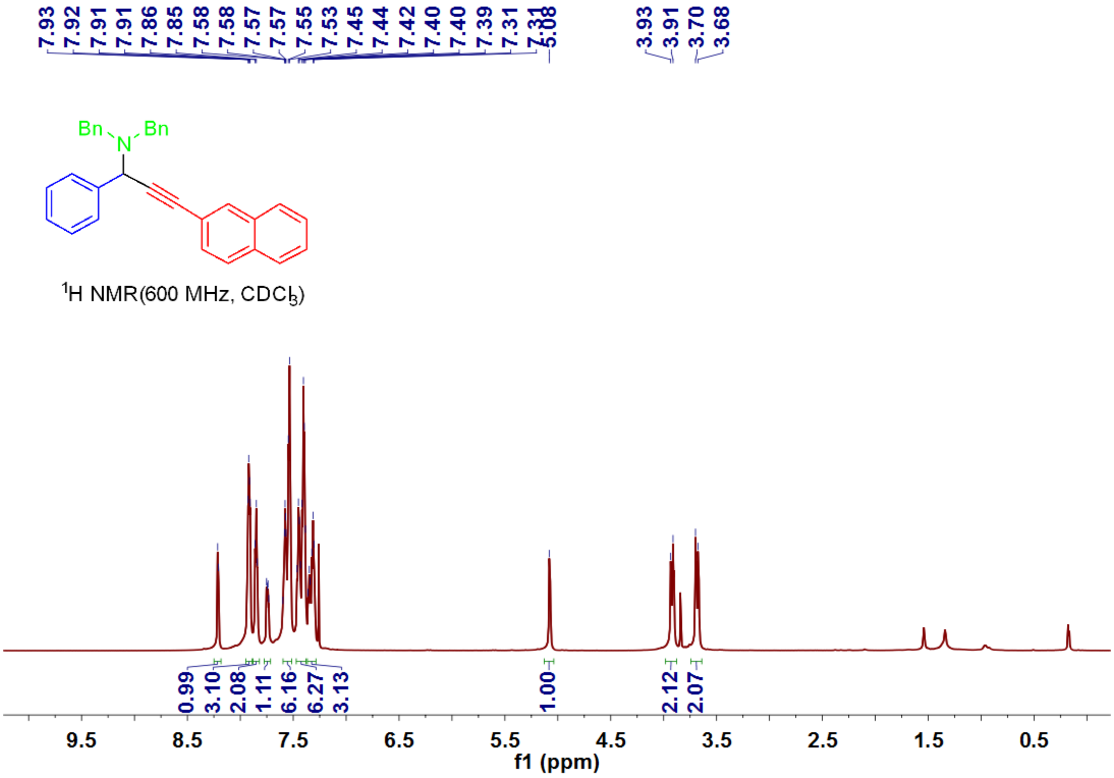

Supplementary Figure 148. ^{1}**H NMR** spectrum of compound **4aw**.

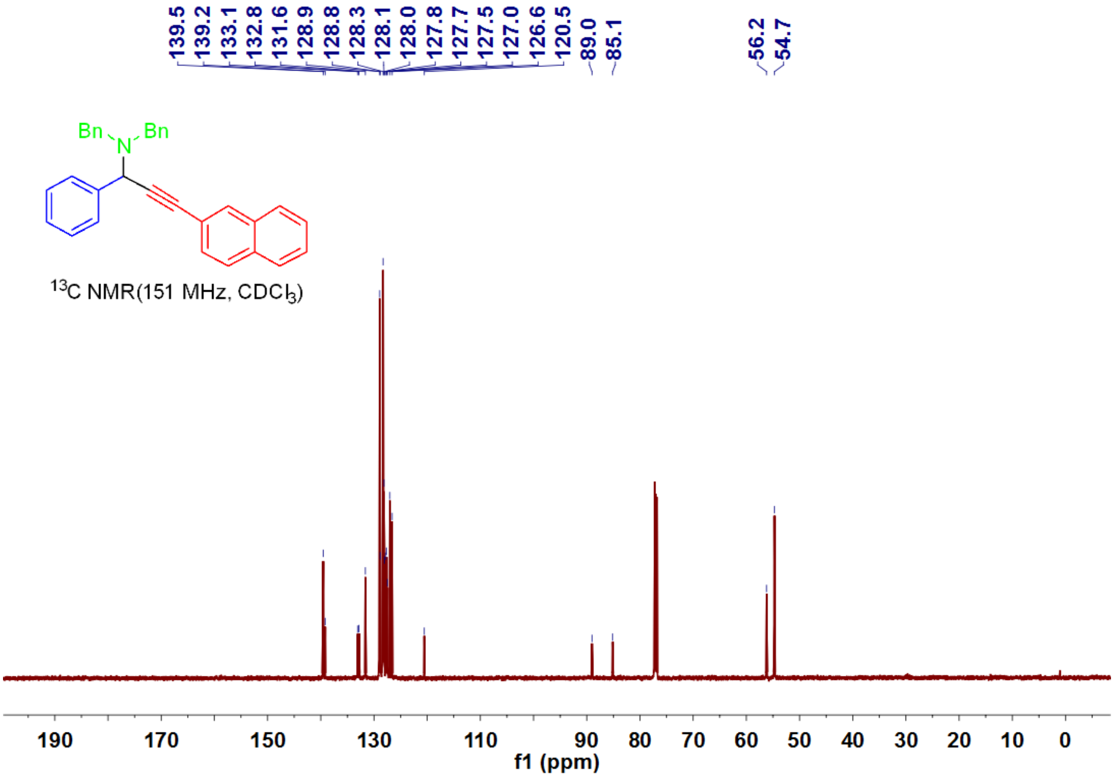

Supplementary Figure 149. ^{13}**C NMR** spectrum of compound **4aw**.

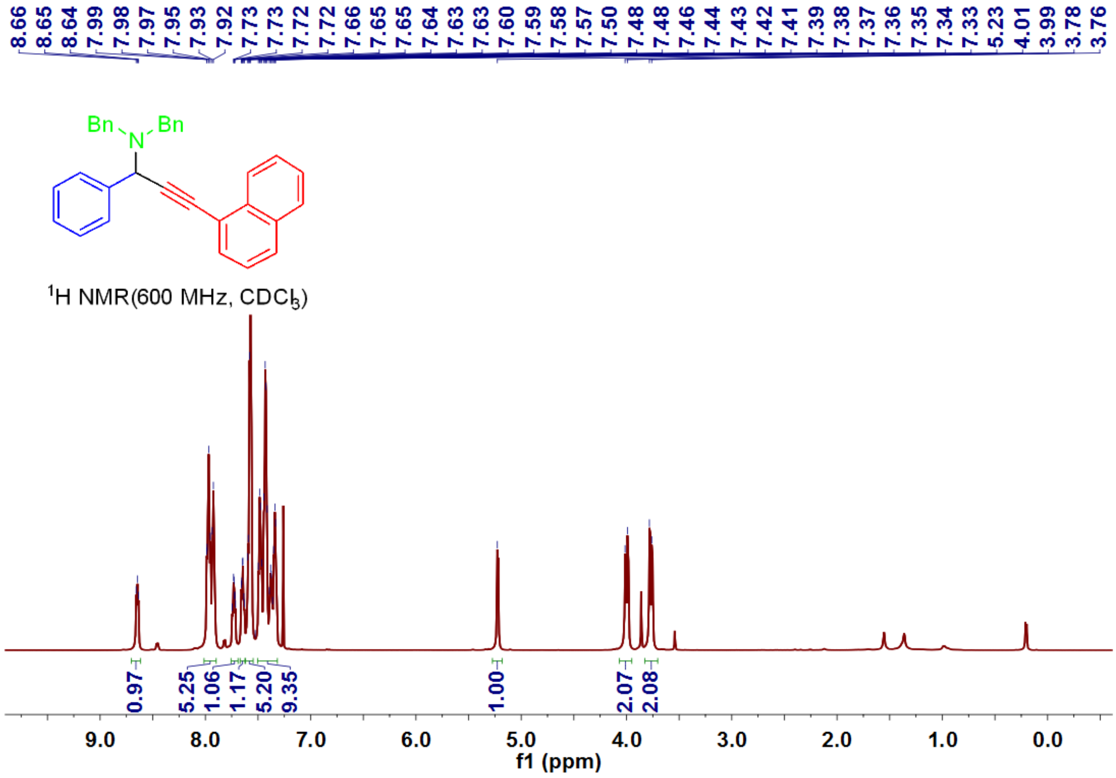

Supplementary Figure 150. ^{1}**H NMR** spectrum of compound **4ax**.

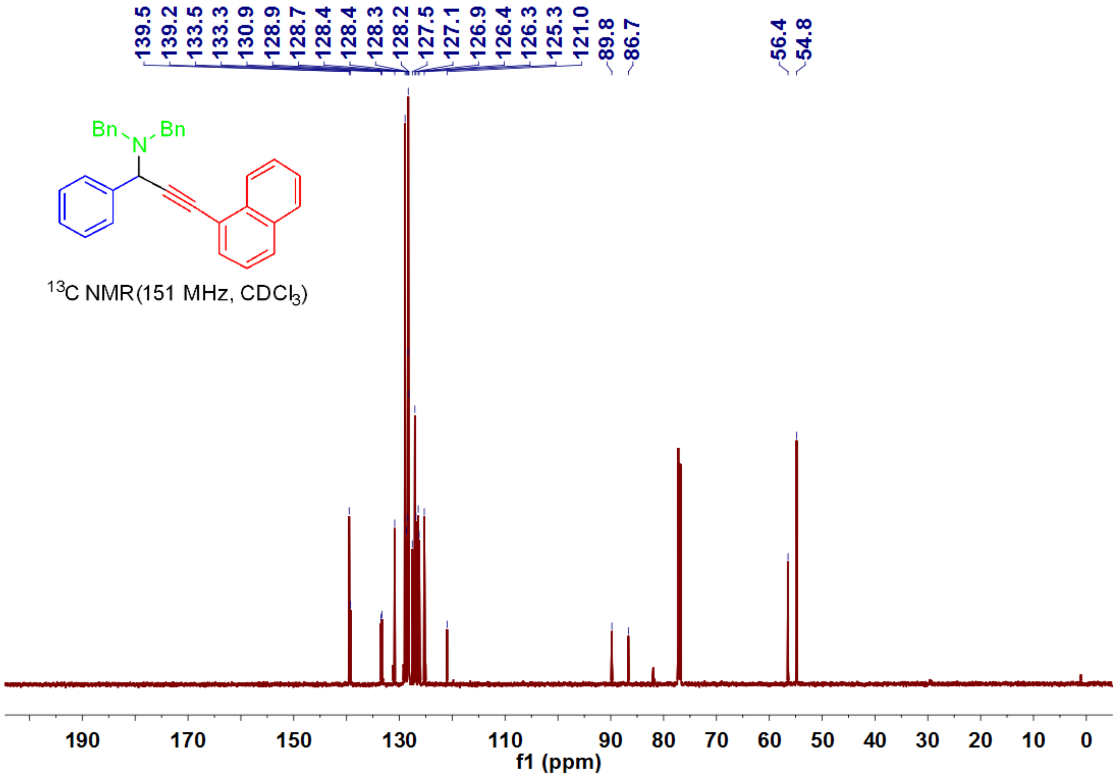

Supplementary Figure 151. ^{13}**C NMR** spectrum of compound **4ax**.

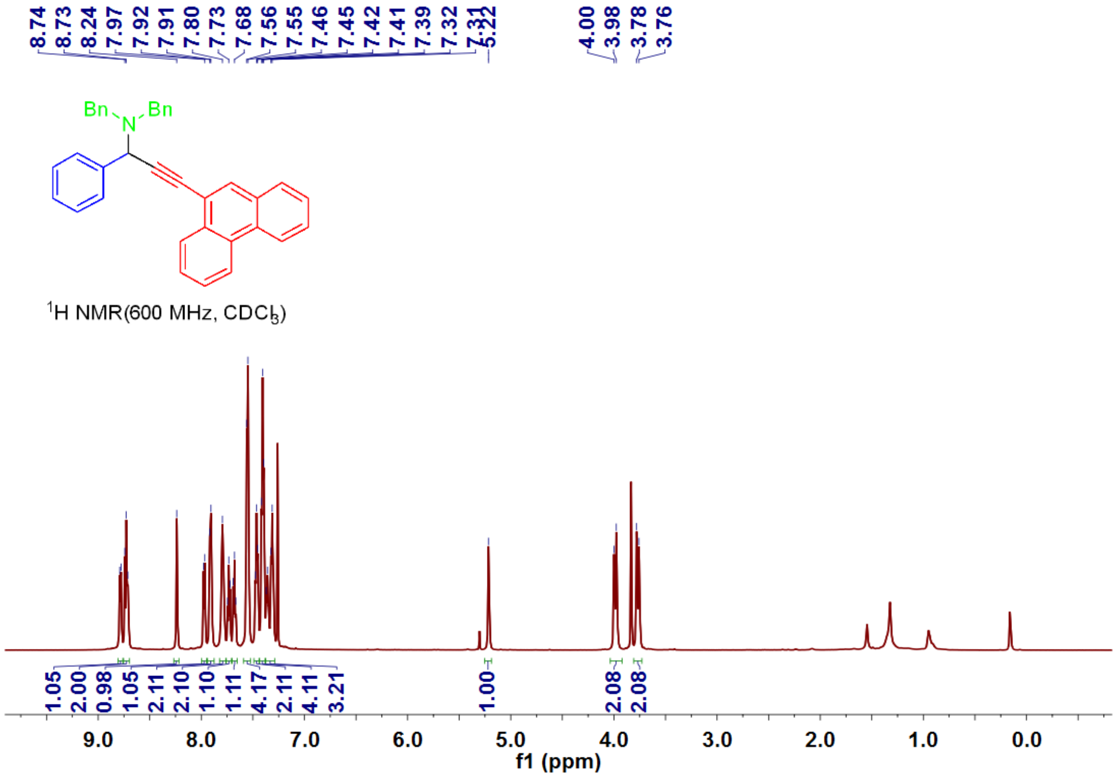

Supplementary Figure 152. ^{1}**H NMR** spectrum of compound **4ay**.

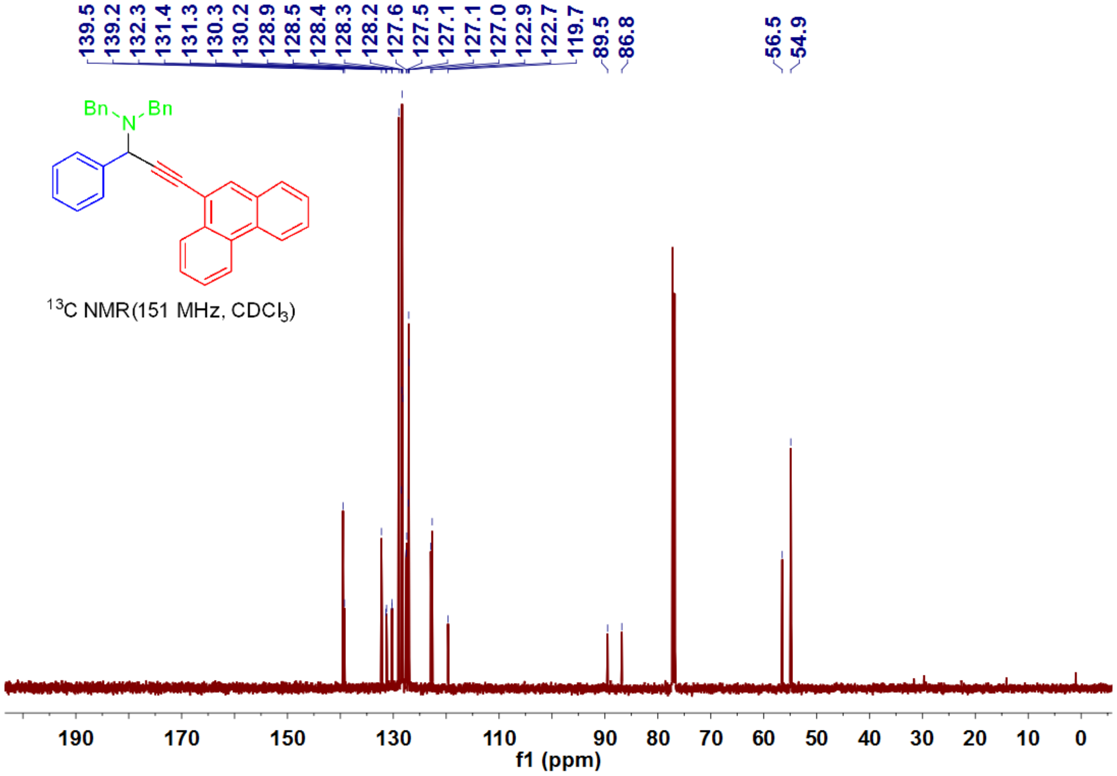

Supplementary Figure 153. ^{13}**C NMR** spectrum of compound **4ay**.

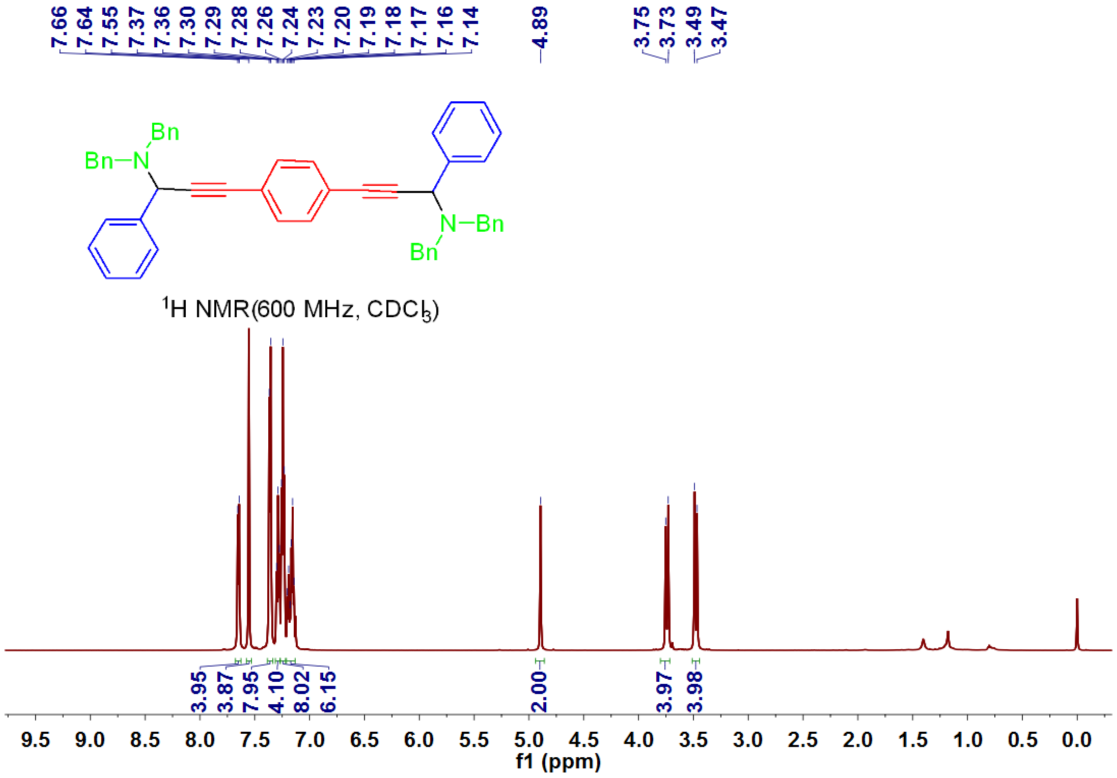

Supplementary Figure 154. ^{1}**H NMR** spectrum of compound **4az**.

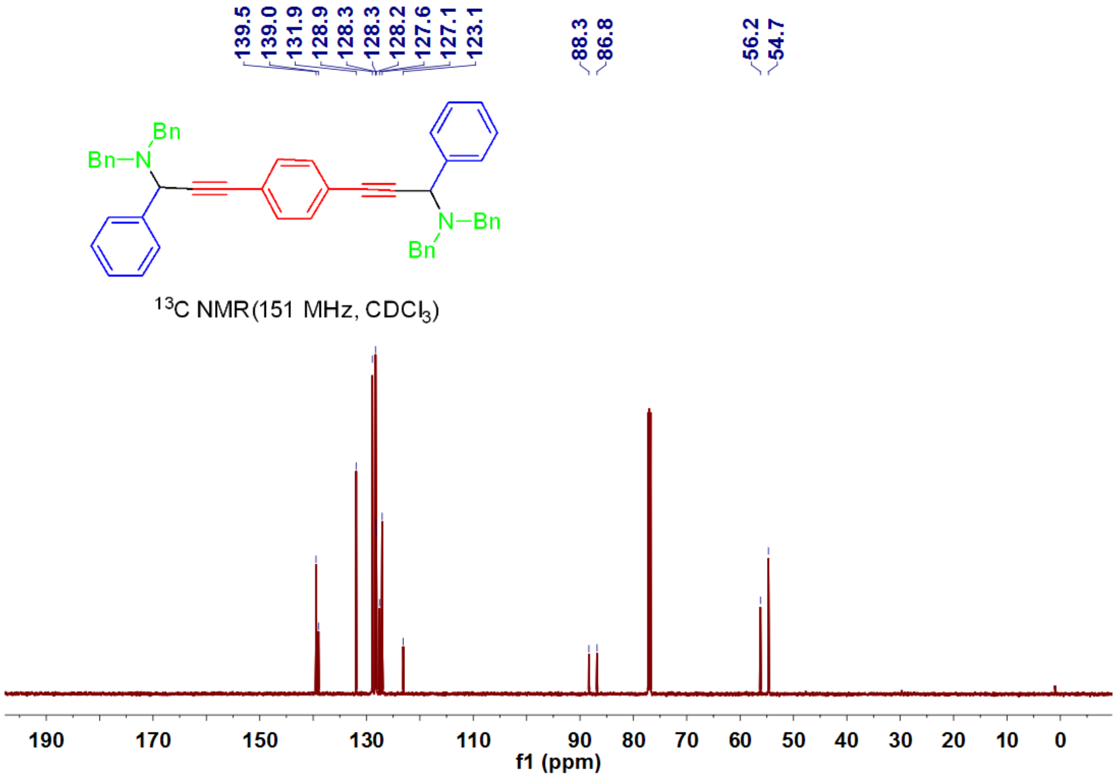

Supplementary Figure 155. ^{13}**C NMR** spectrum of compound **4az**.

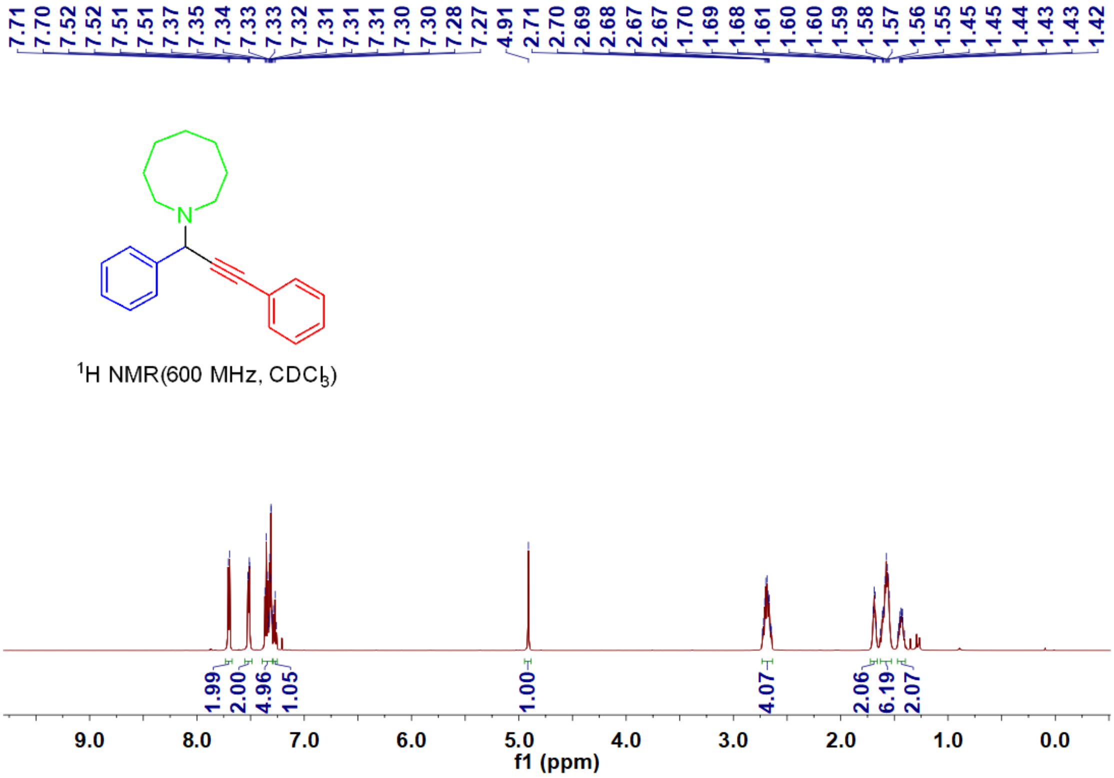

Supplementary Figure 156. ^{1}**H NMR** spectrum of compound **4ba**.

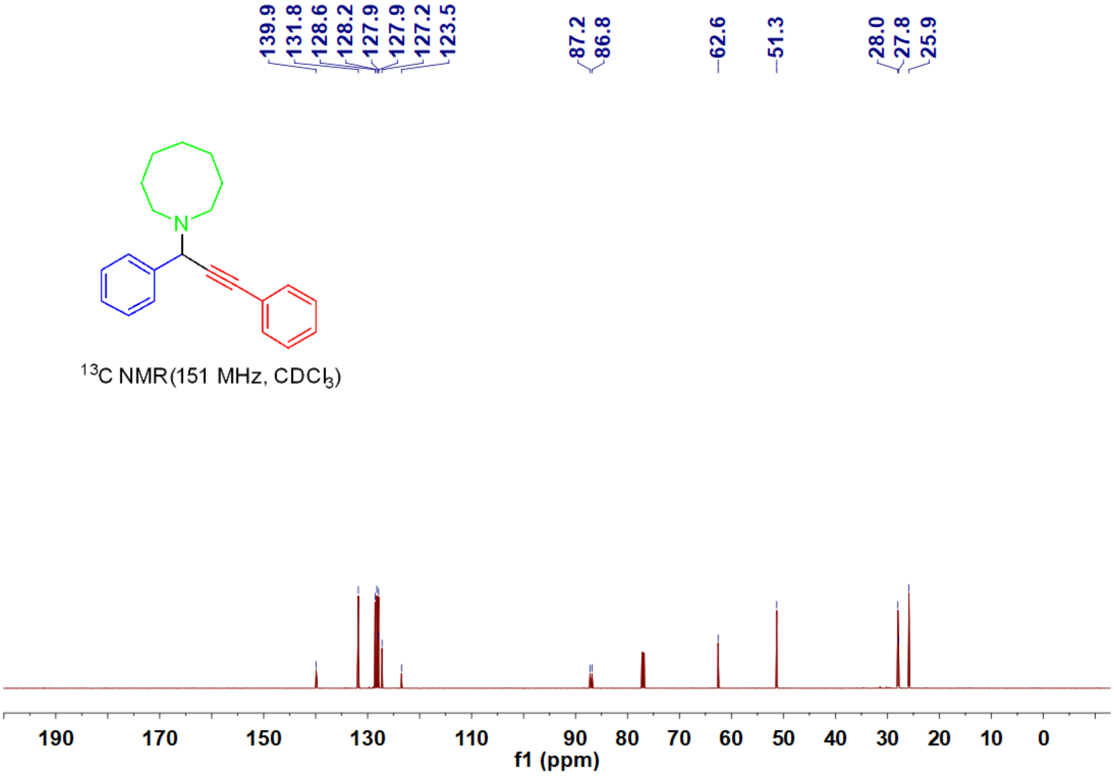

Supplementary Figure 157. ^{13}**C NMR** spectrum of compound **4ba**.

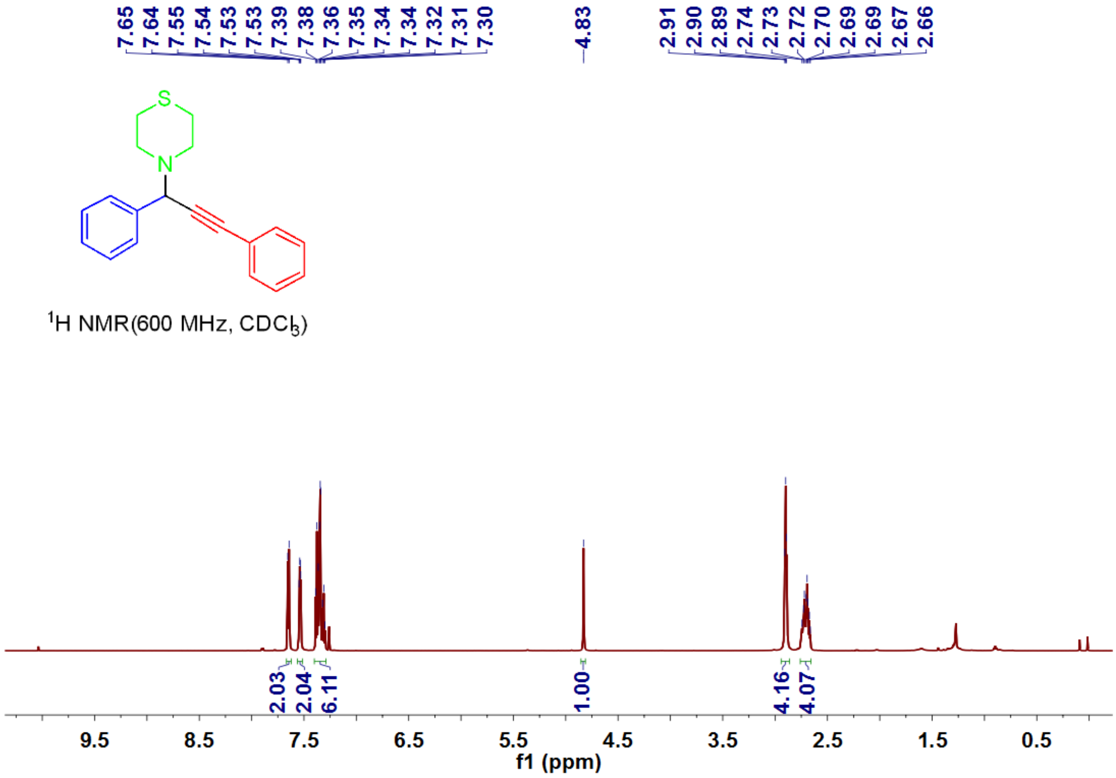

Supplementary Figure 158. ^{1}**H NMR** spectrum of compound **4bb**.

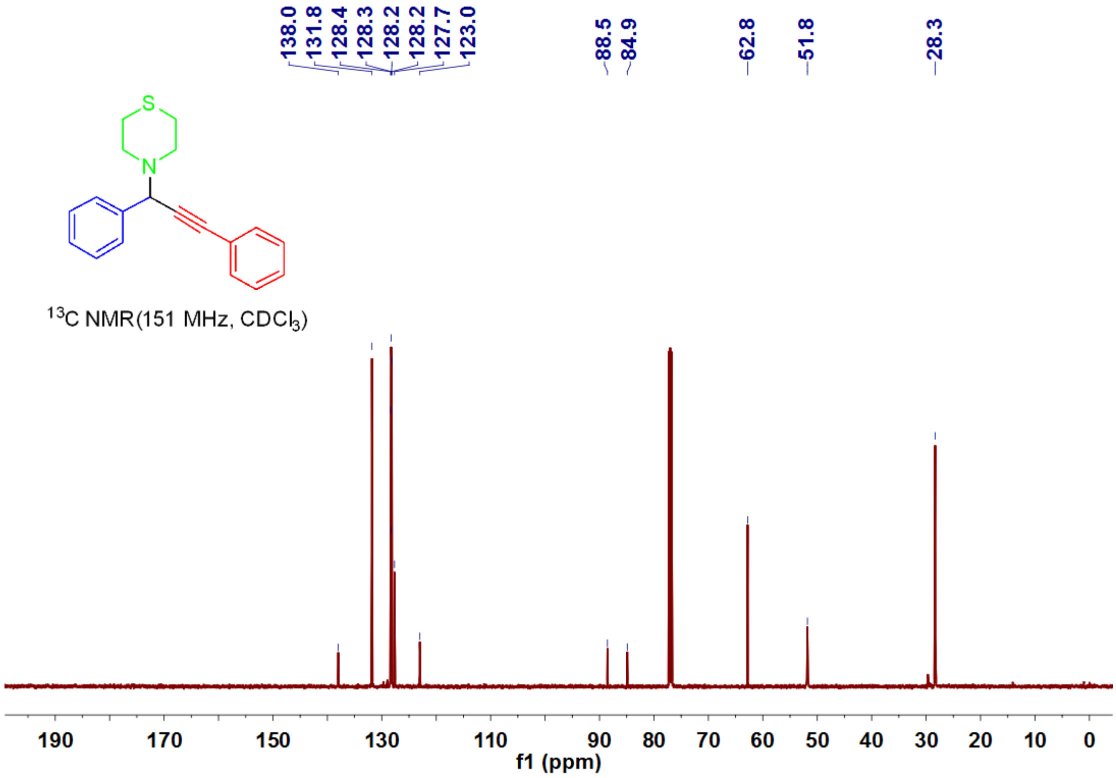

Supplementary Figure 159. ^{13}**C NMR** spectrum of compound **4bb**.

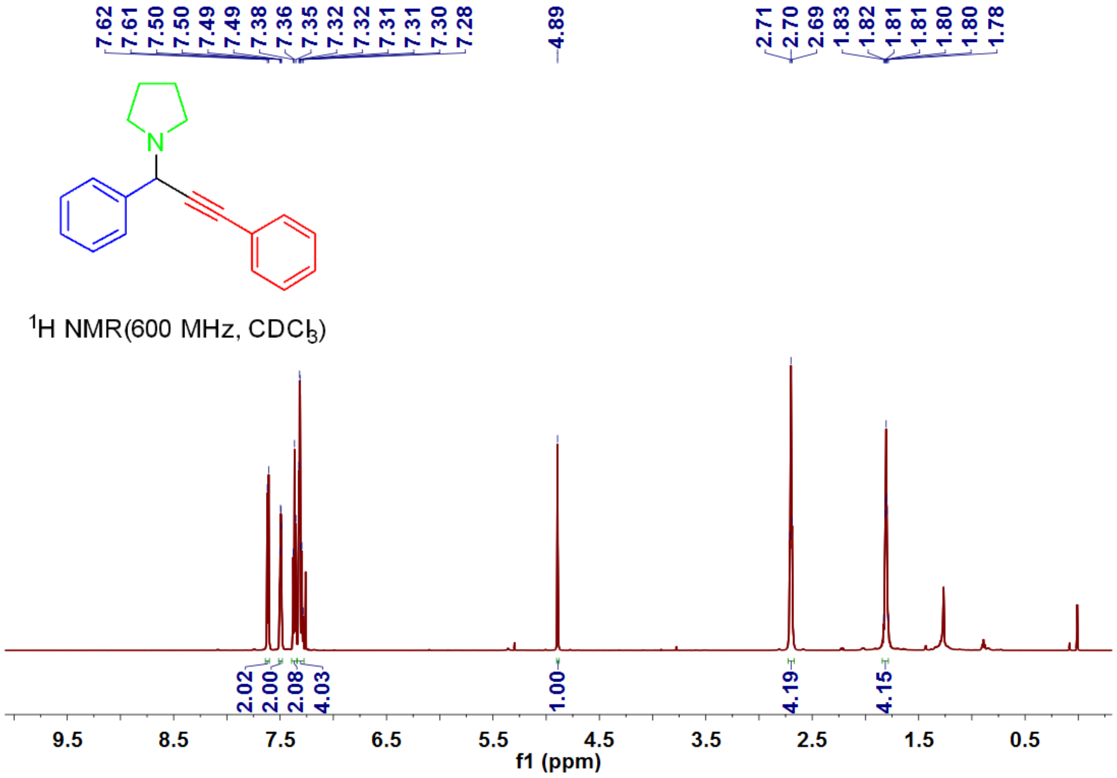

Supplementary Figure 160. ^{1}**H NMR** spectrum of compound **4bc**.

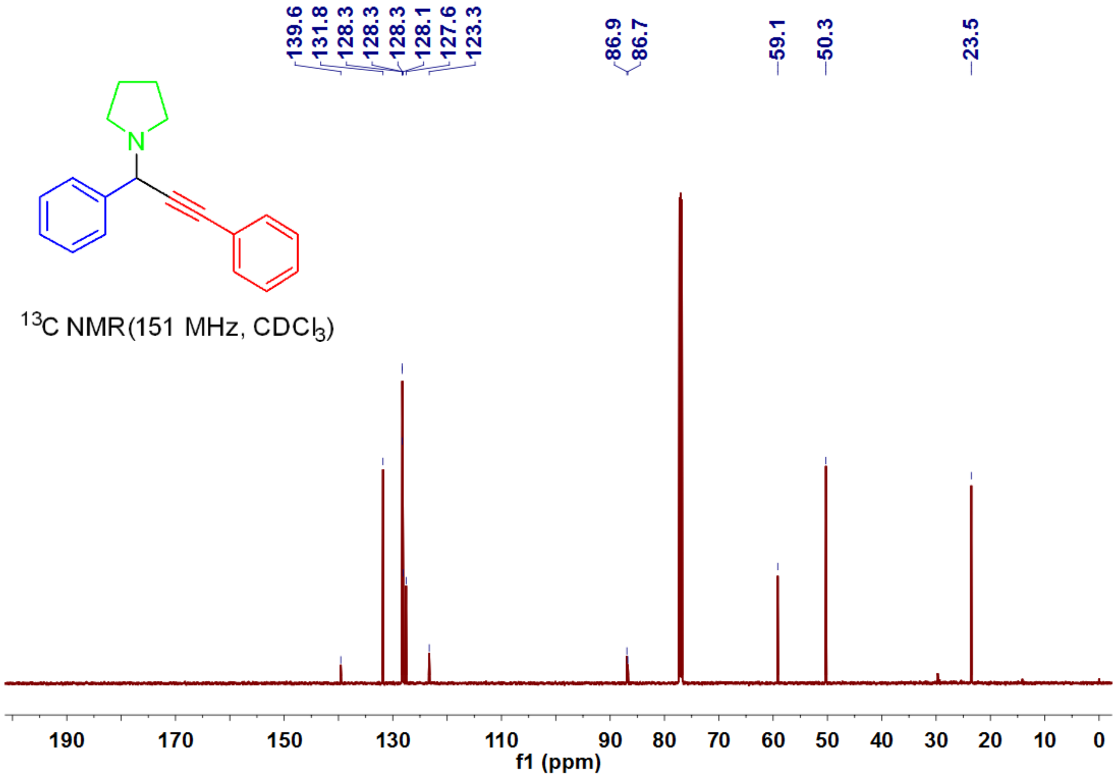

Supplementary Figure 161. ^{13}**C NMR** spectrum of compound **4bc**.

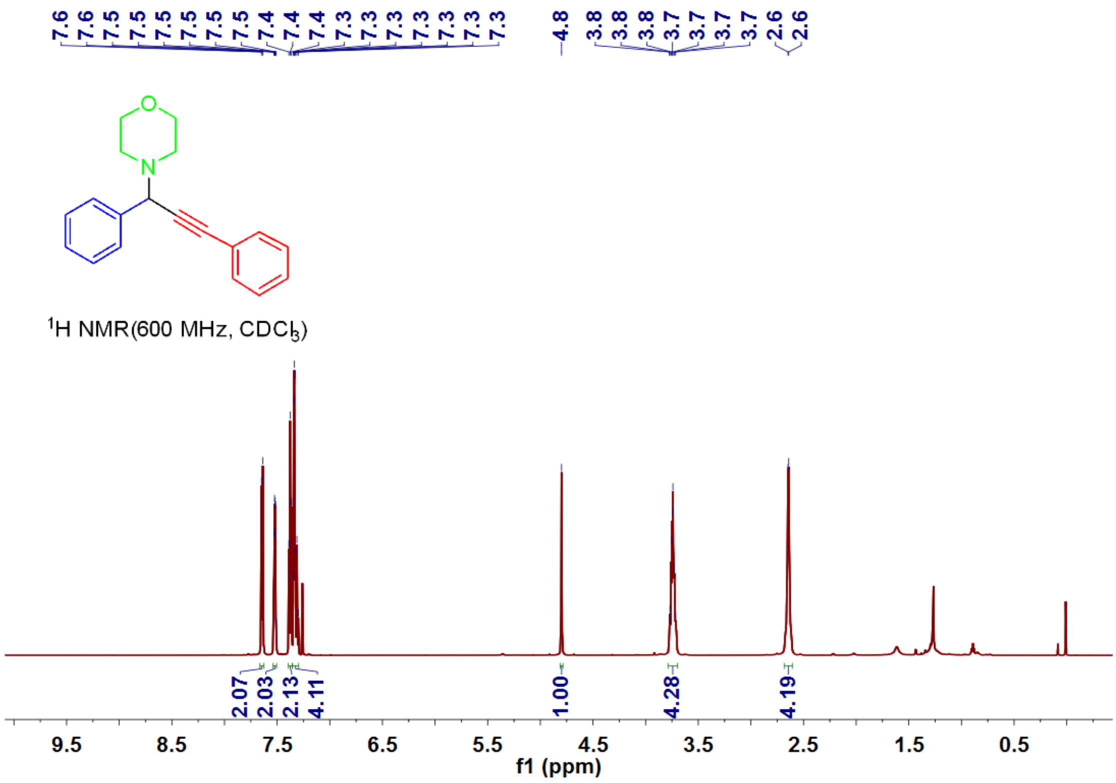

Supplementary Figure 162. **^{1}H NMR** spectrum of compound **4bd**.

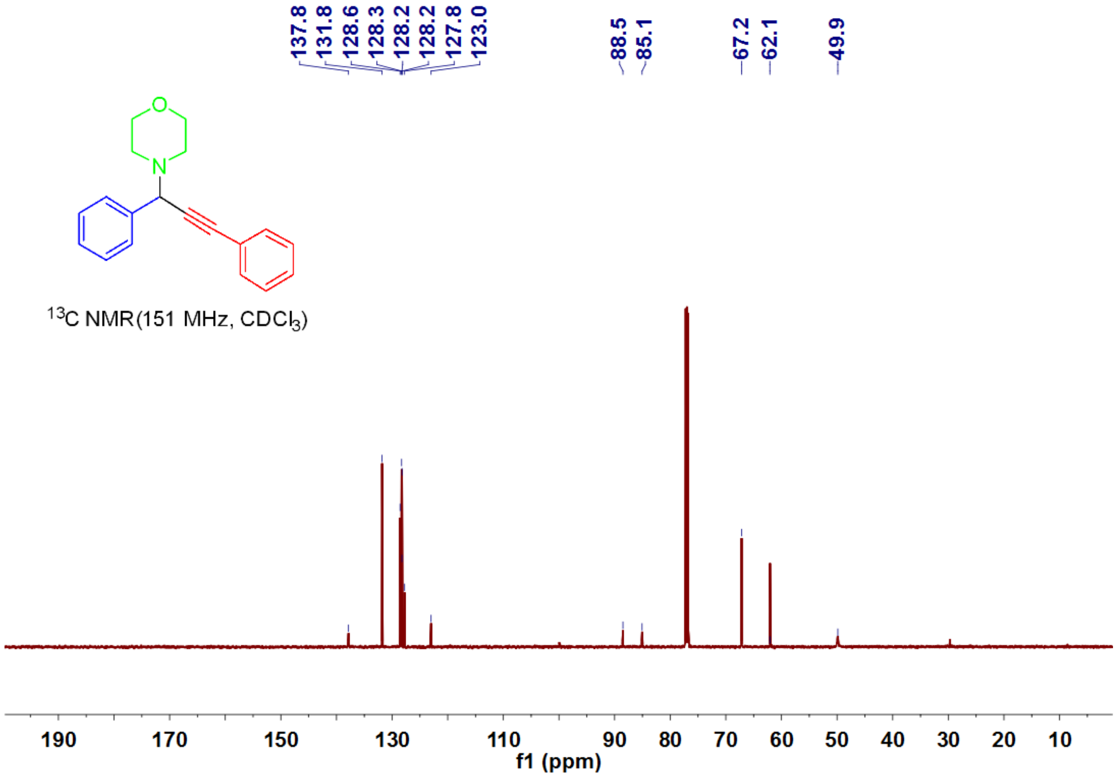

Supplementary Figure 163. **^{13}C NMR** spectrum of compound **4bd**.

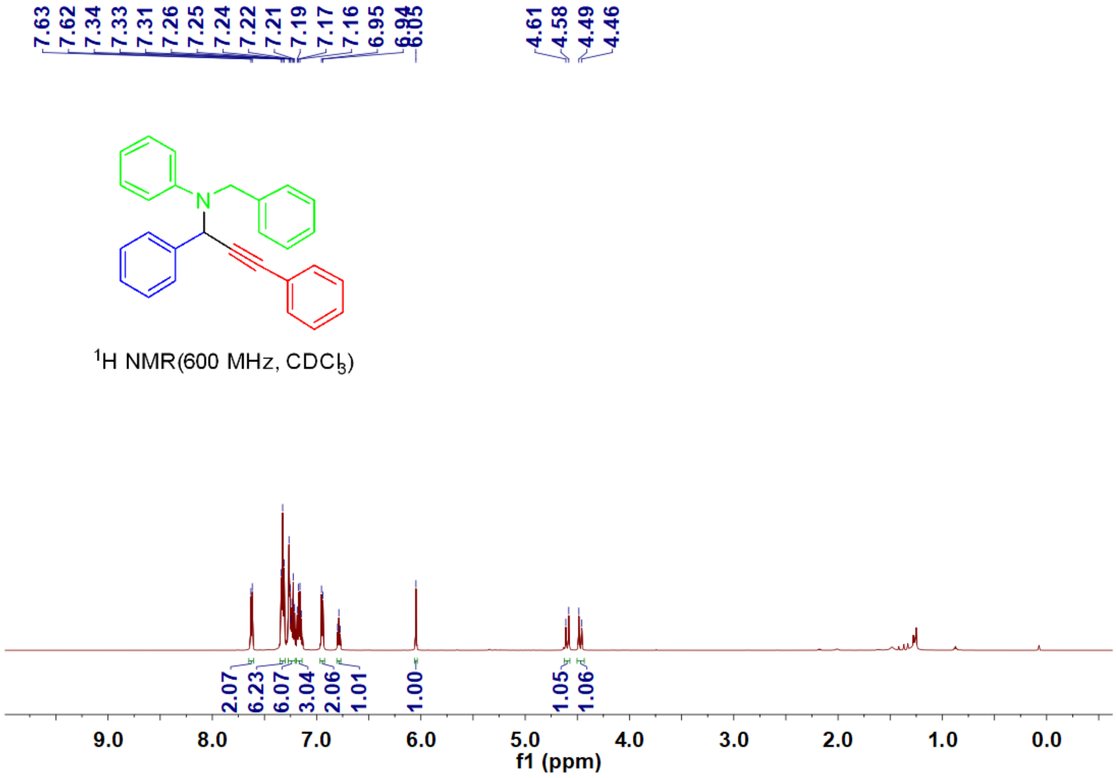

Supplementary Figure 164. **^{1}H NMR** spectrum of compound **4be**.

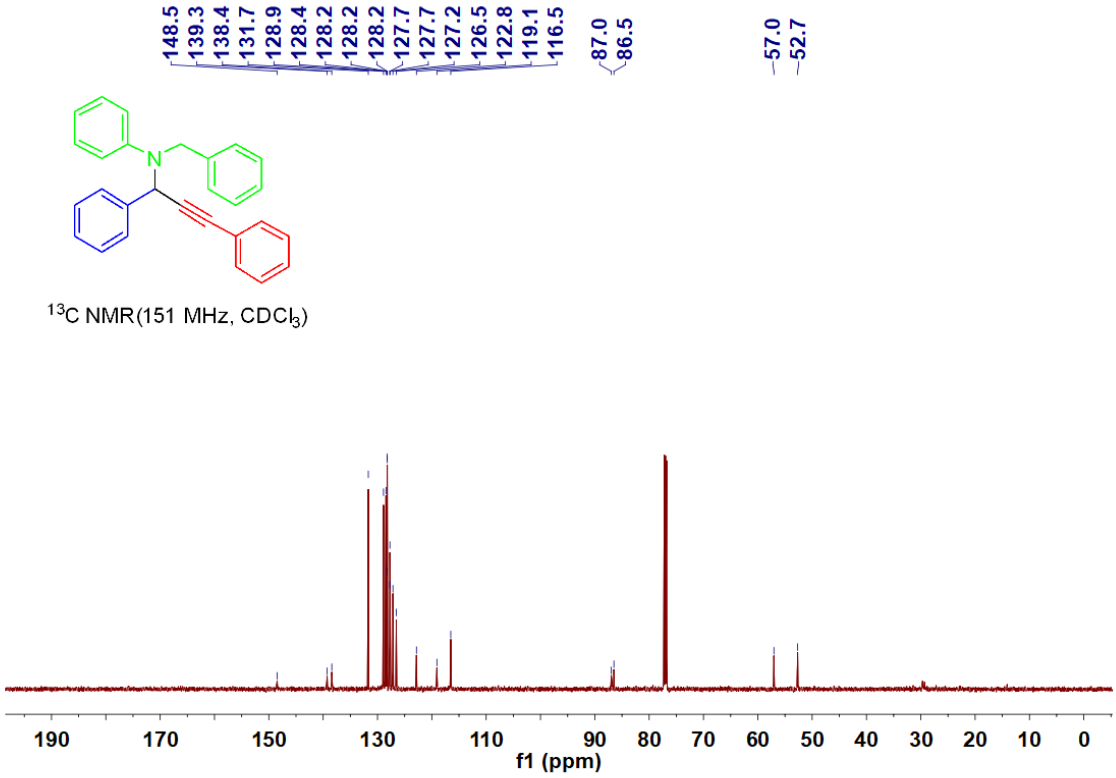

Supplementary Figure 165. **^{13}C NMR** spectrum of compound **4be**.

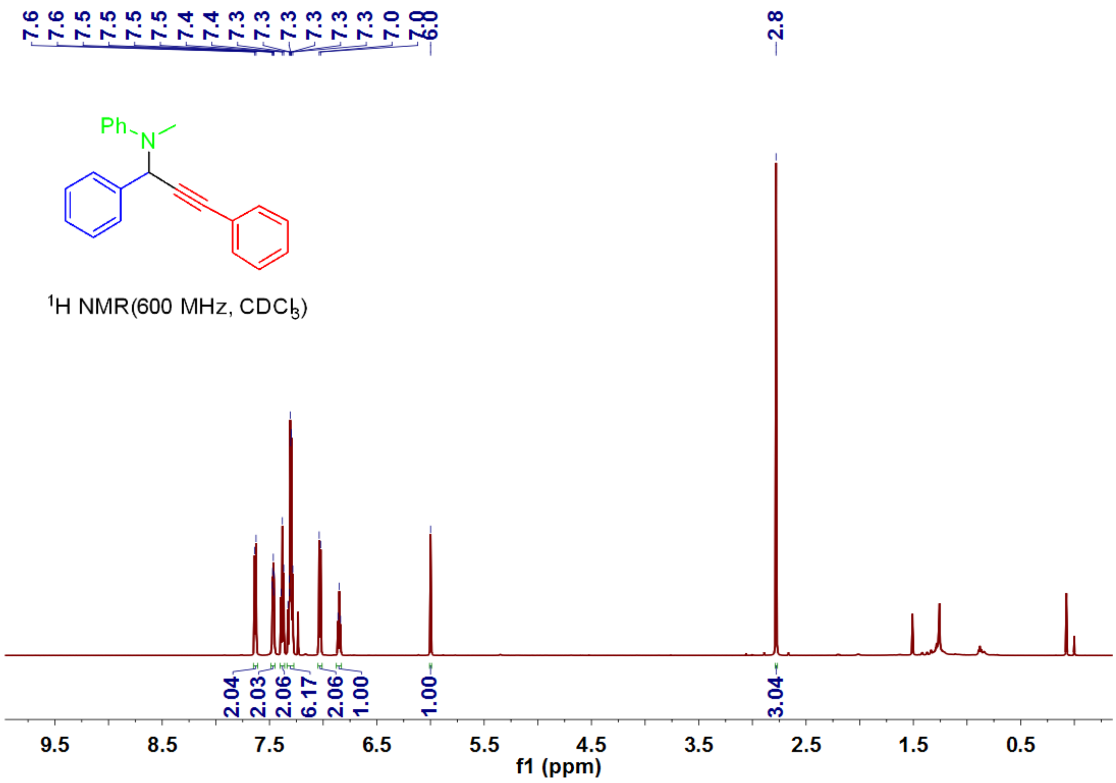

Supplementary Figure 166. ^{1}H NMR spectrum of compound **4bf**.

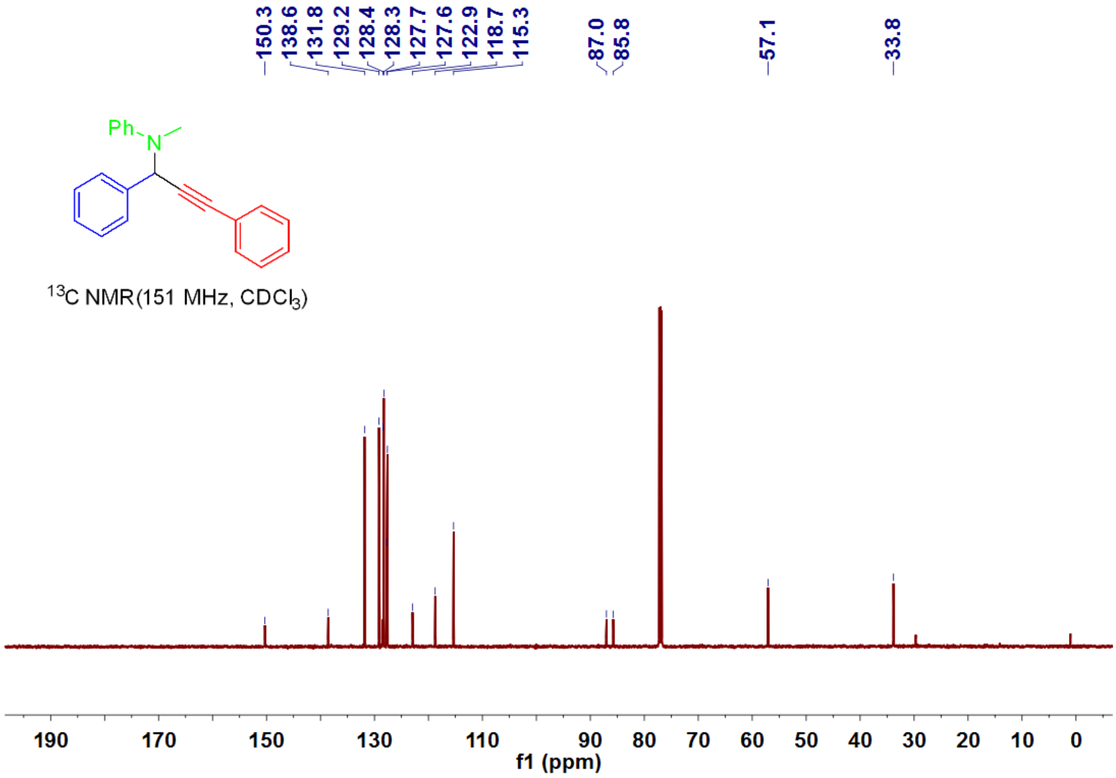

Supplementary Figure 167. ^{13}C NMR spectrum of compound **4bf**.

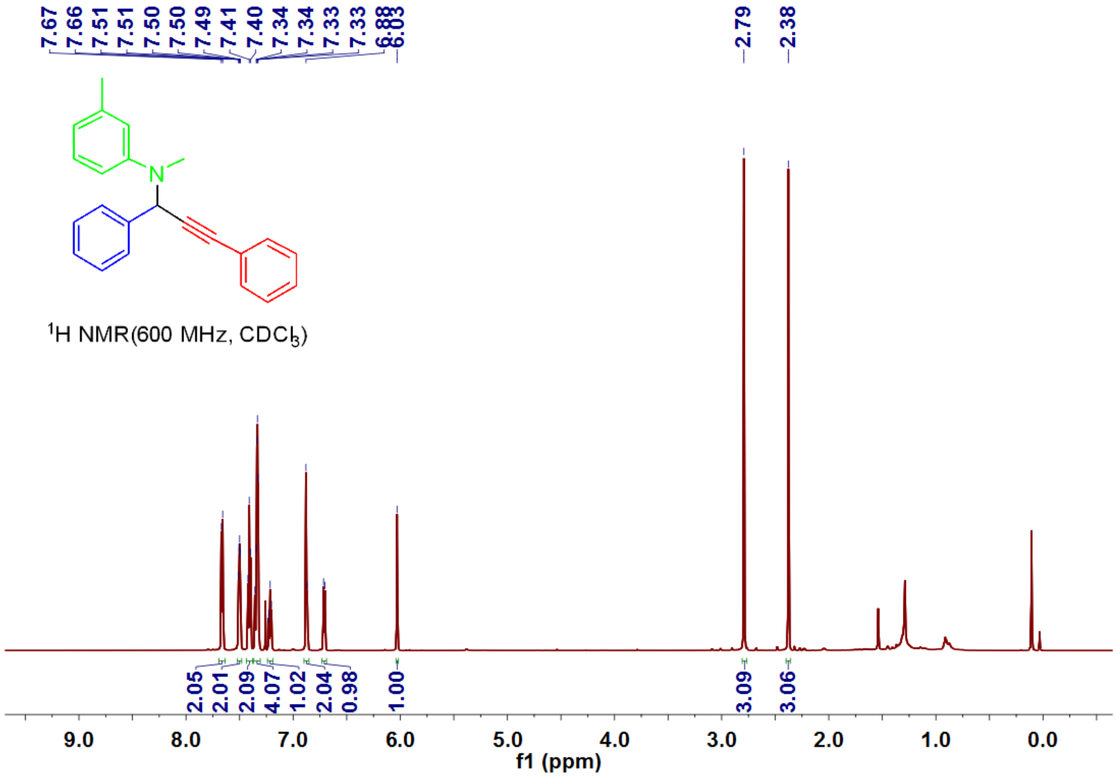

Supplementary Figure 168. ^{1}**H NMR** spectrum of compound **4bg**.

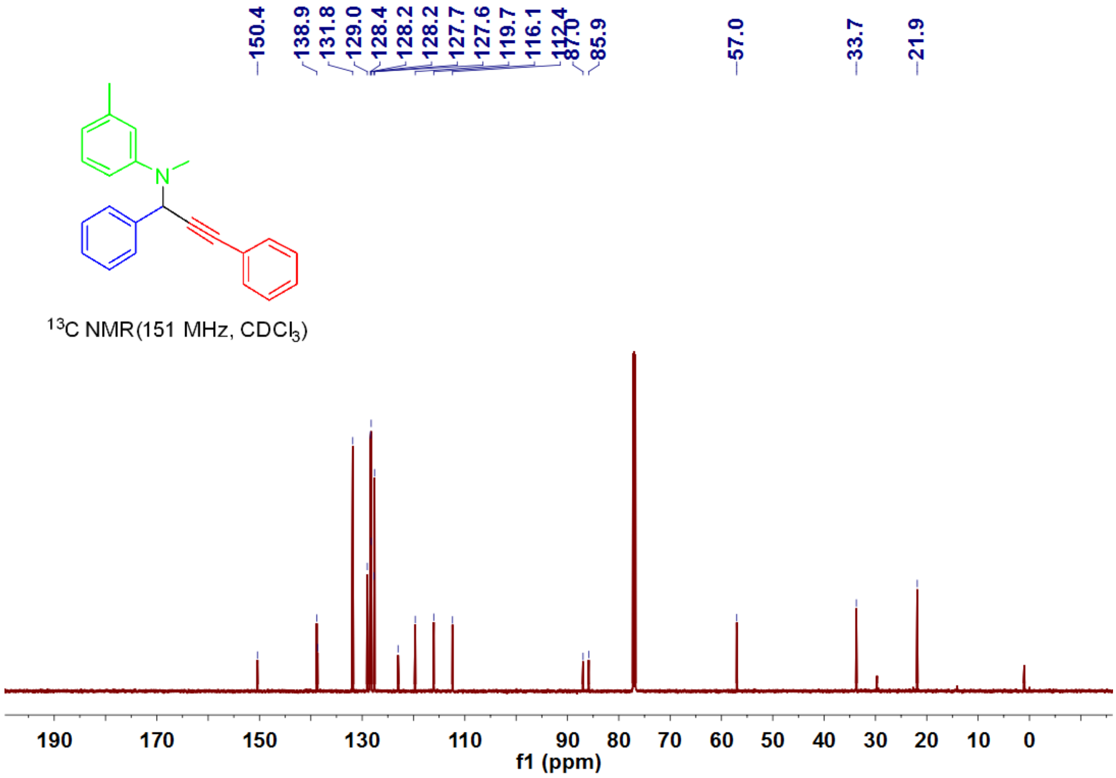

Supplementary Figure 169. ^{13}**C NMR** spectrum of compound **4bg**.

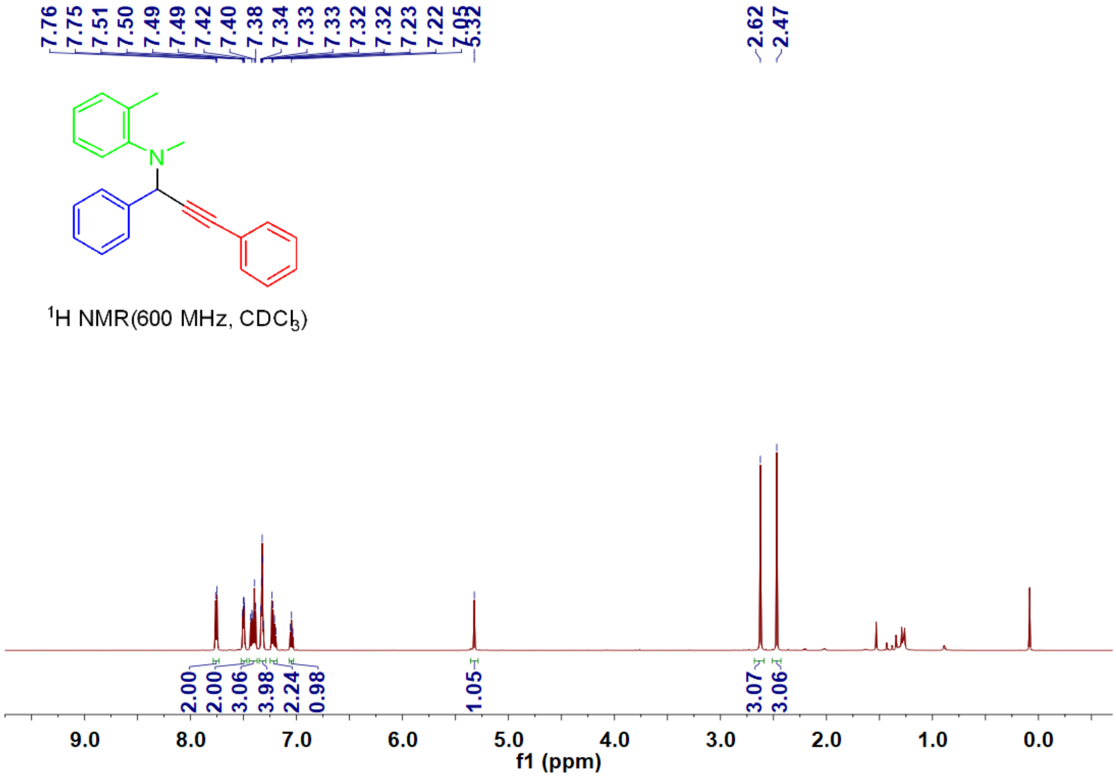

Supplementary Figure 170. ^{1}**H NMR** spectrum of compound **4bh**.

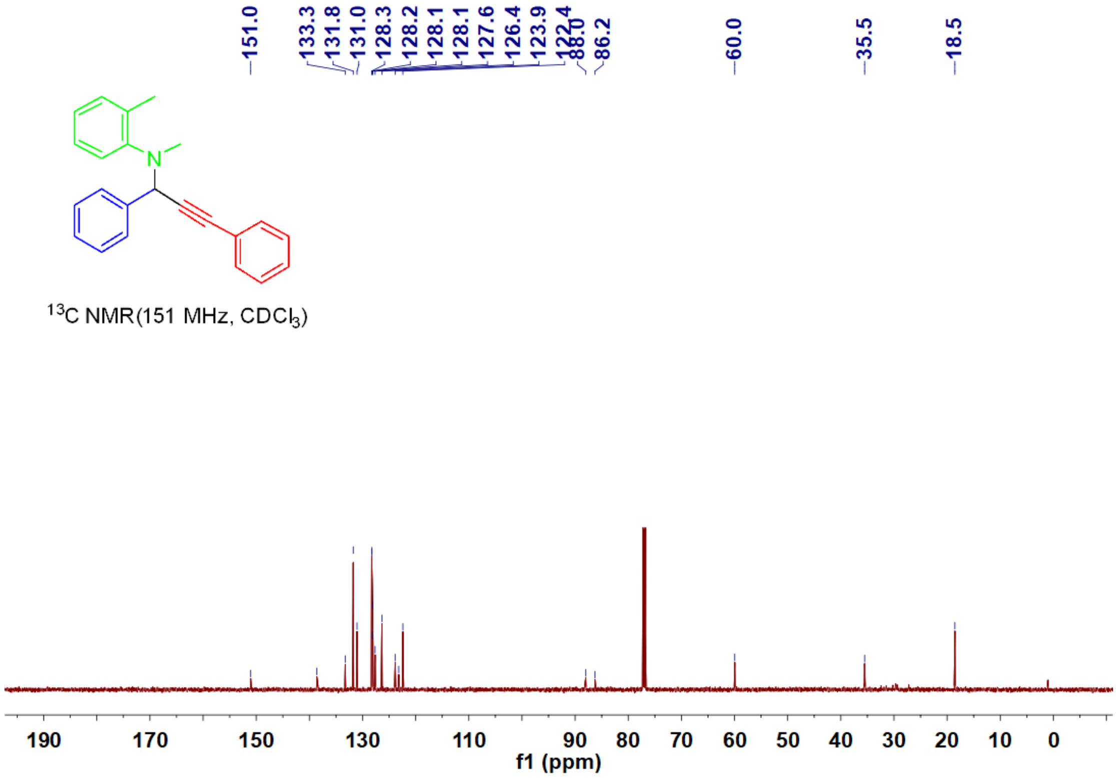

Supplementary Figure 171.^{13}**C NMR** spectrum of compound **4bh**.

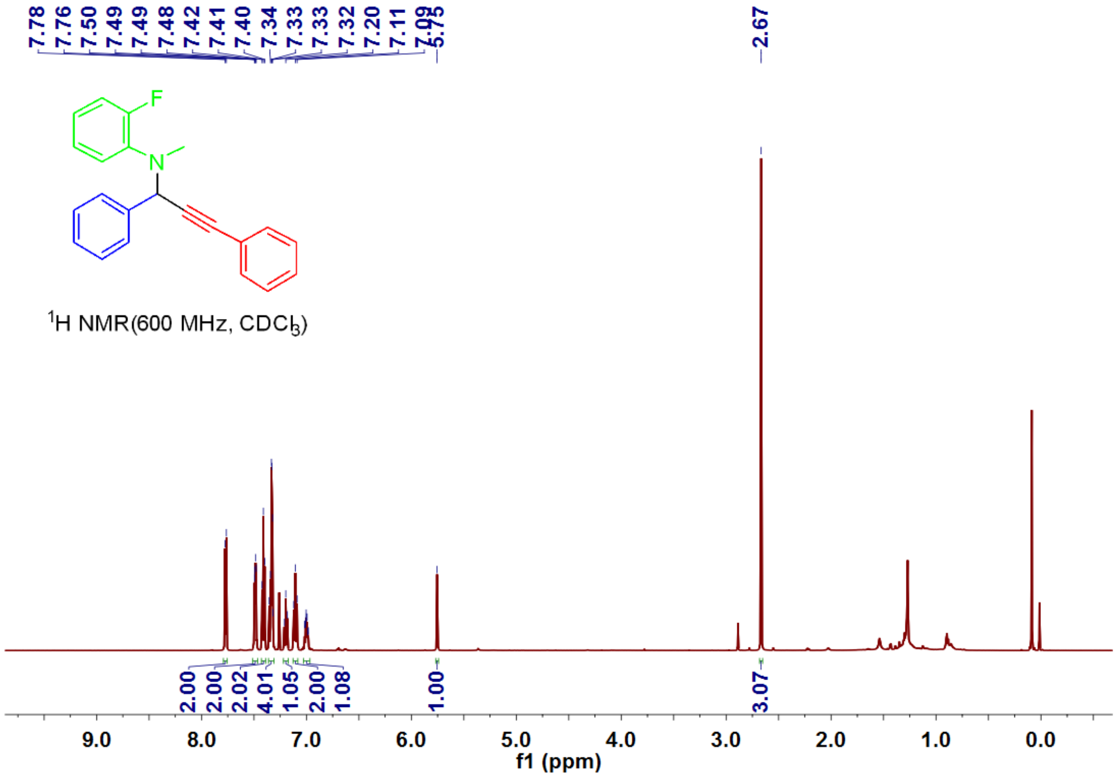

Supplementary Figure 172.^{1}**H NMR** spectrum of compound **4bi**.

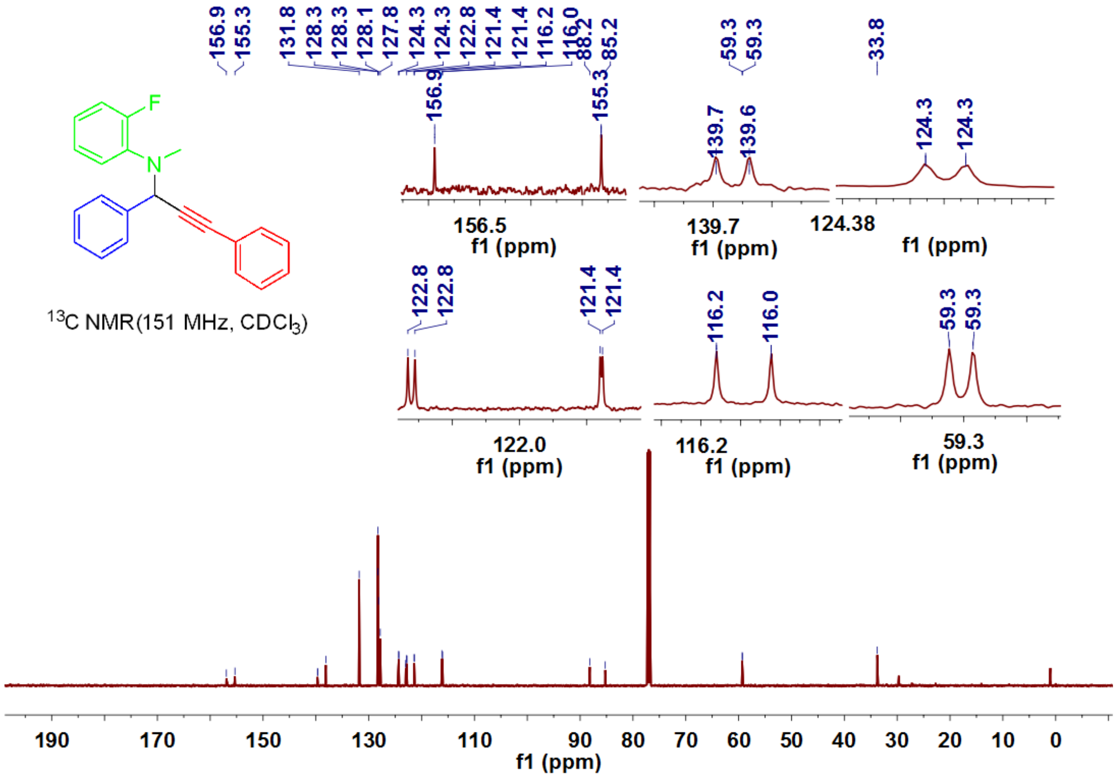

Supplementary Figure 173. ^{13}**C NMR** spectrum of compound **4bi**.

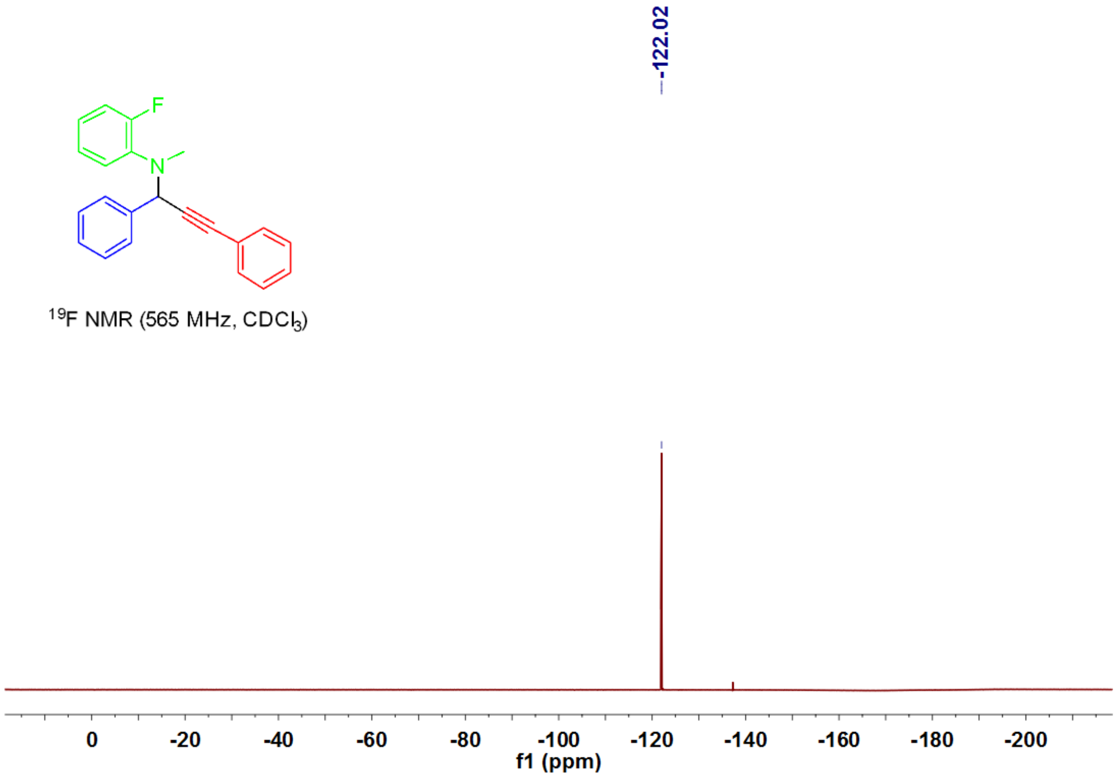

Supplementary Figure 174. ^{19}**F NMR** spectrum of compound **4bi**.

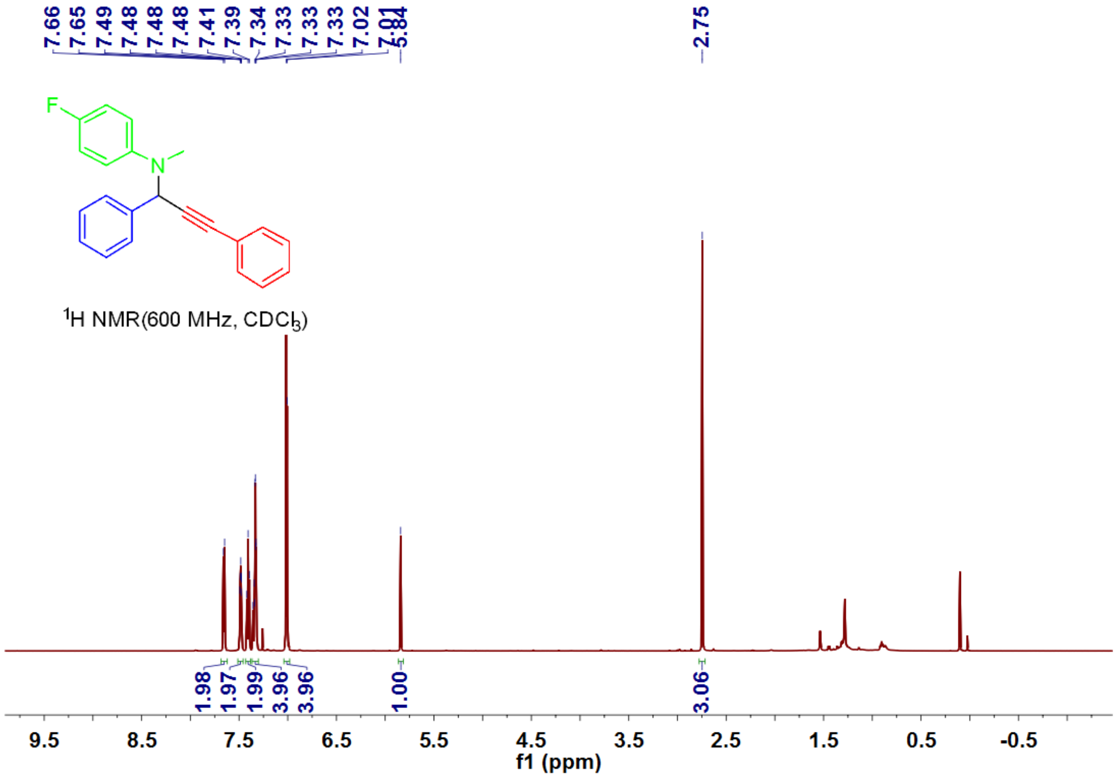

Supplementary Figure 175. ^{1}**H NMR** spectrum of compound **4bj**.

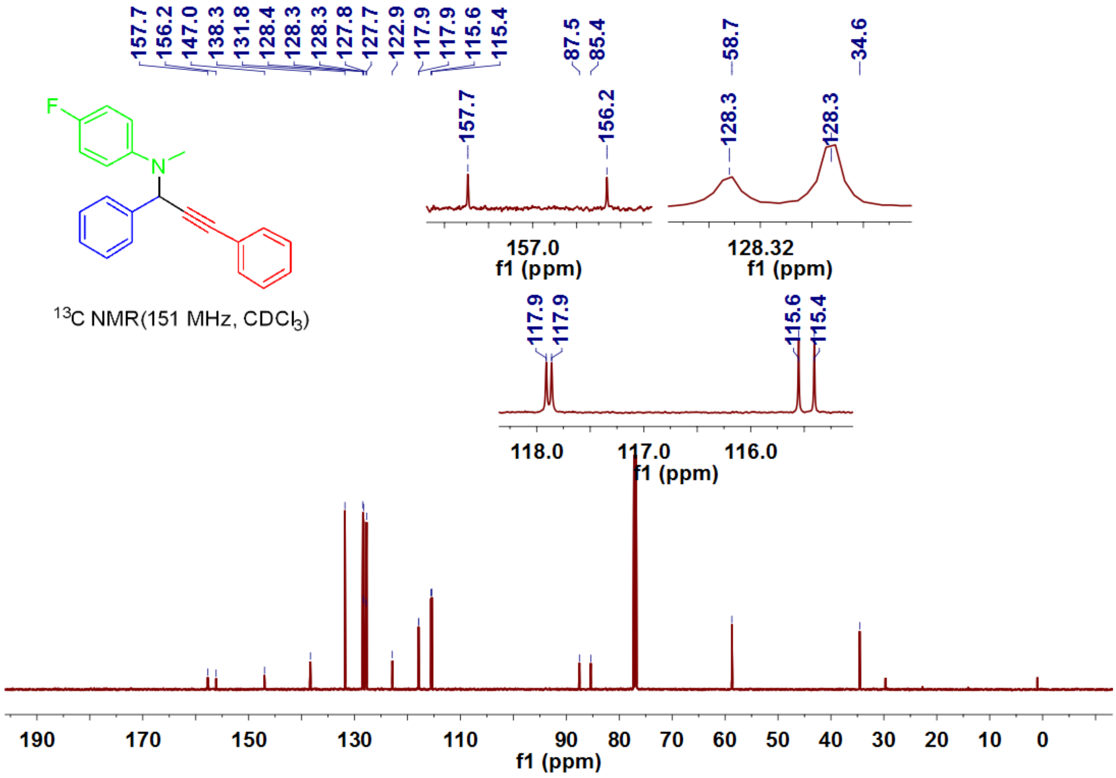

Supplementary Figure 176. ^{13}**C NMR** spectrum of compound **4bj**.

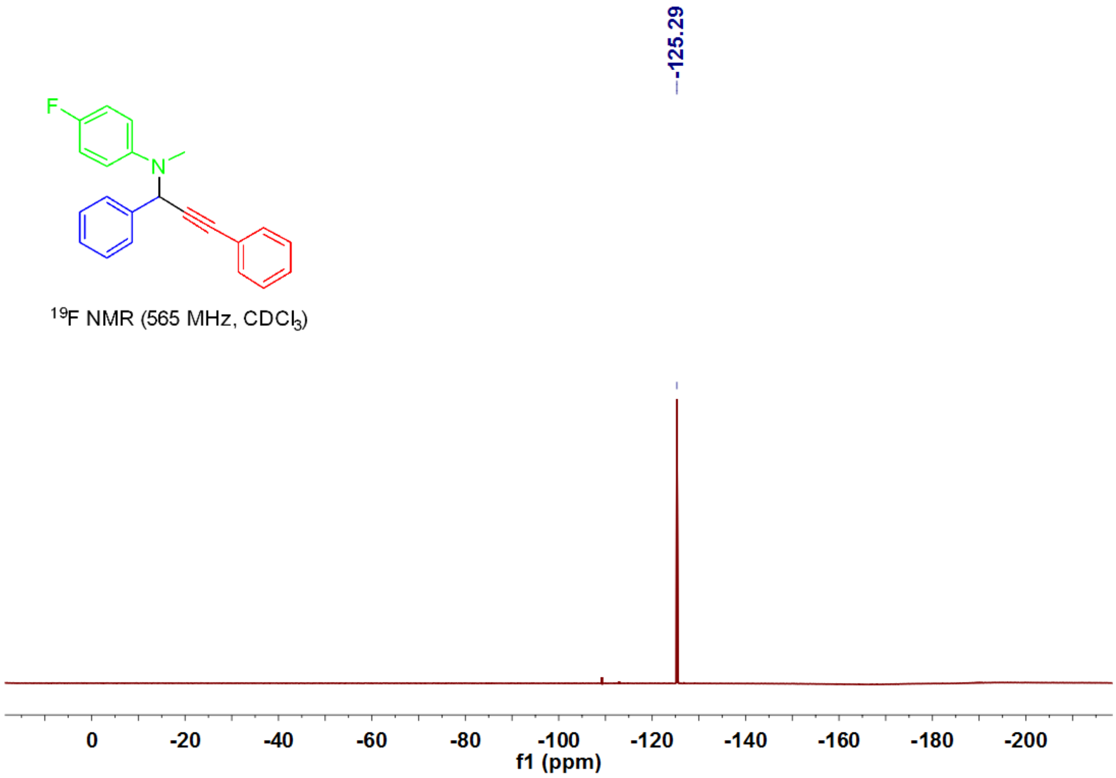

Supplementary Figure 177. ^{19}**F NMR** spectrum of compound **4bj**.

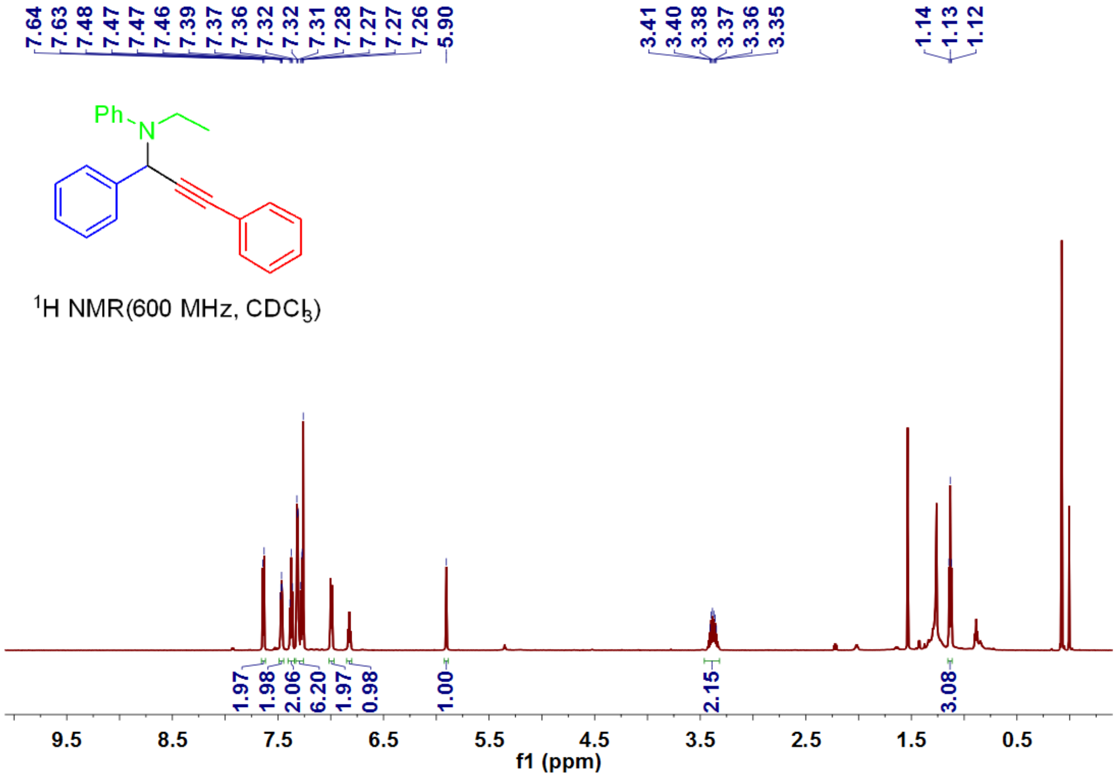

Supplementary Figure 178. ^{1}**H NMR** spectrum of compound **4bk**.

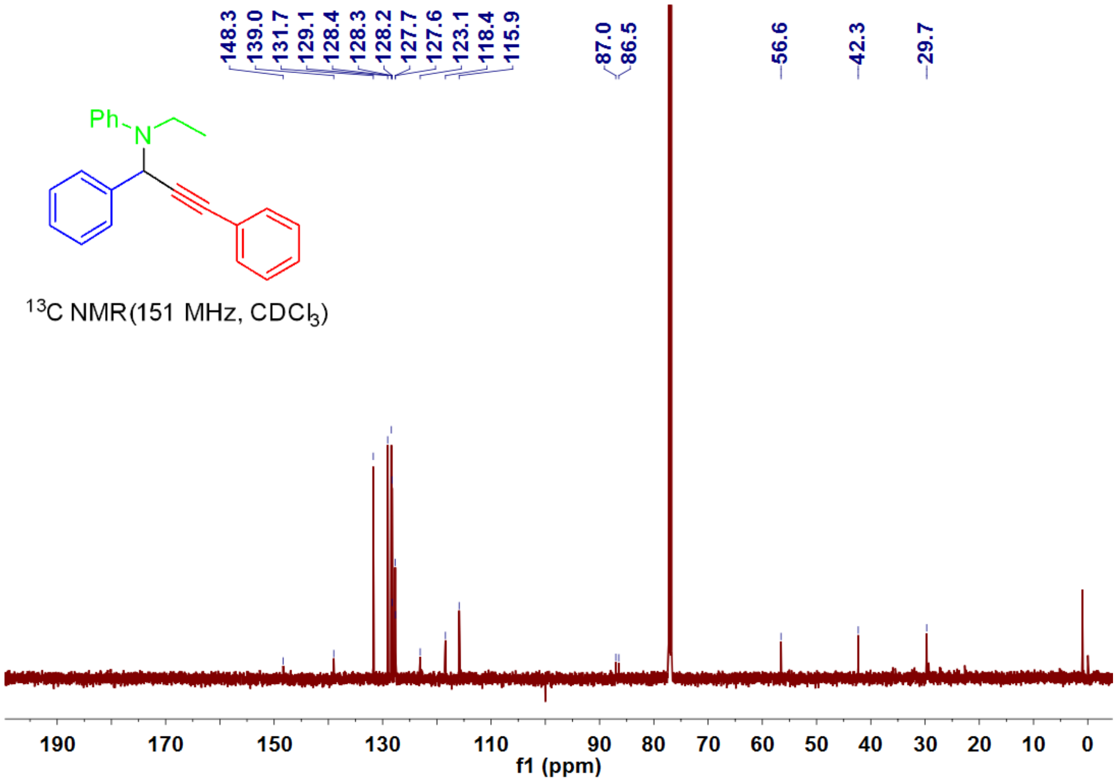

Supplementary Figure 179. ^{13}**C NMR** spectrum of compound **4bk**.

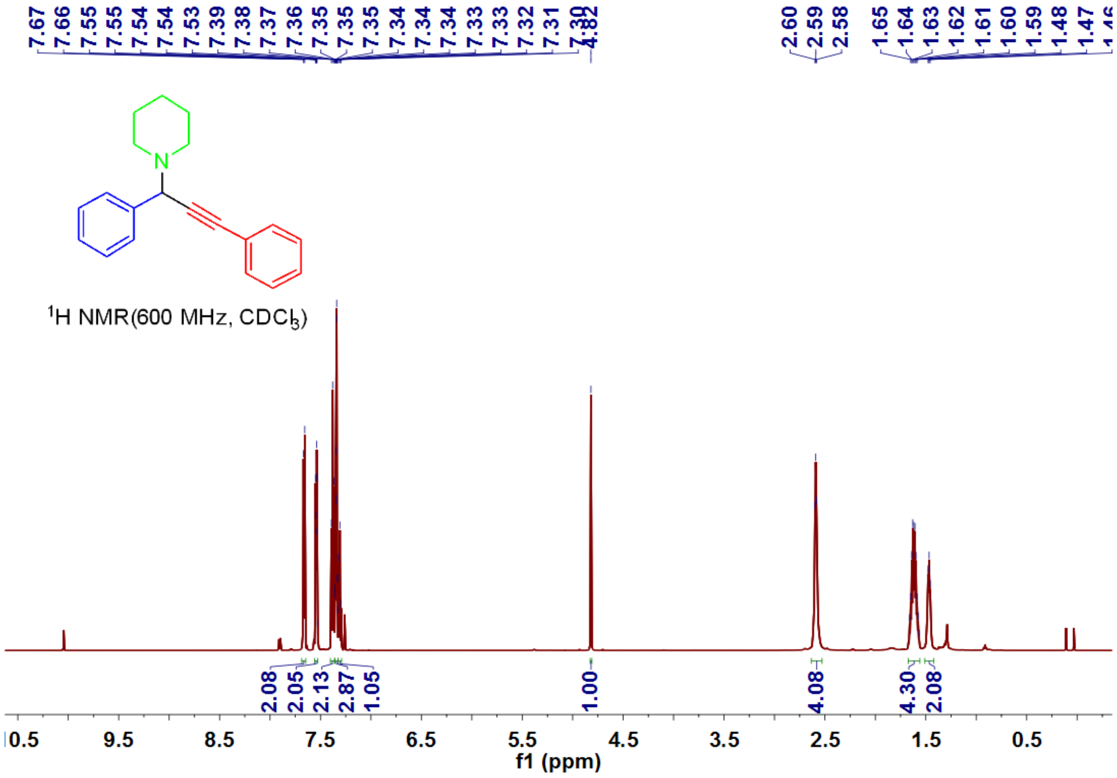

Supplementary Figure 180. ^{1}**H NMR** spectrum of compound **4bl**.

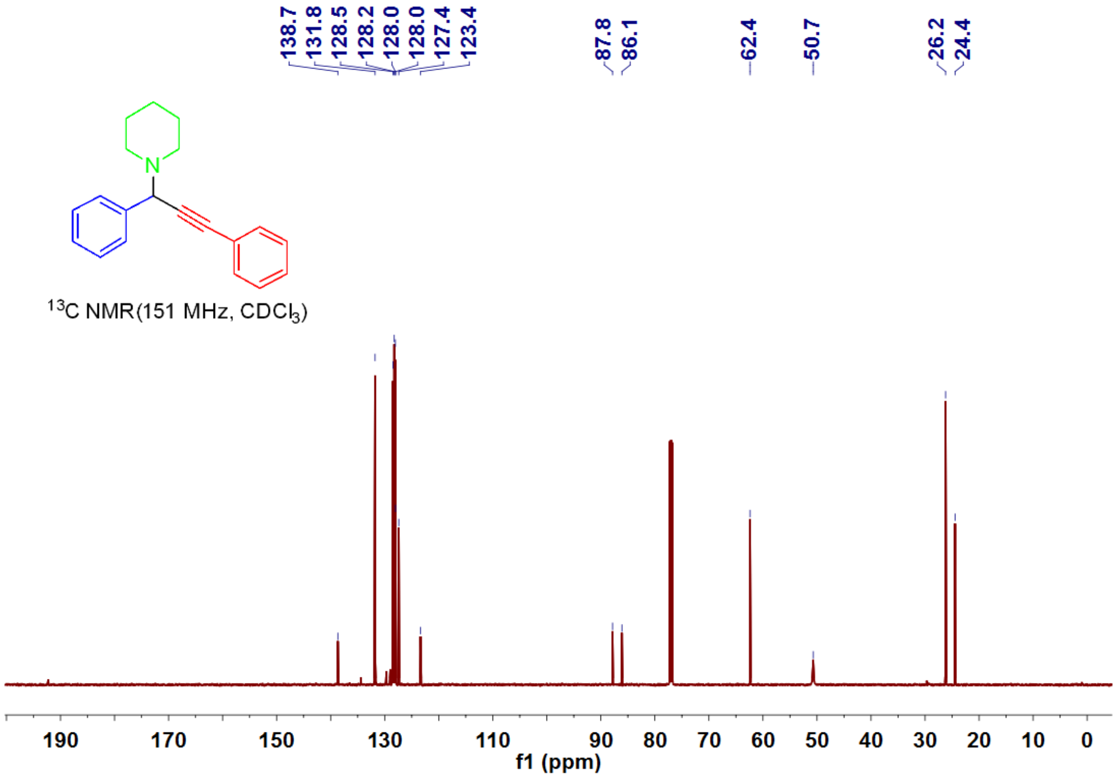

Supplementary Figure 181. ^{13}**C NMR** spectrum of compound **4bl**.

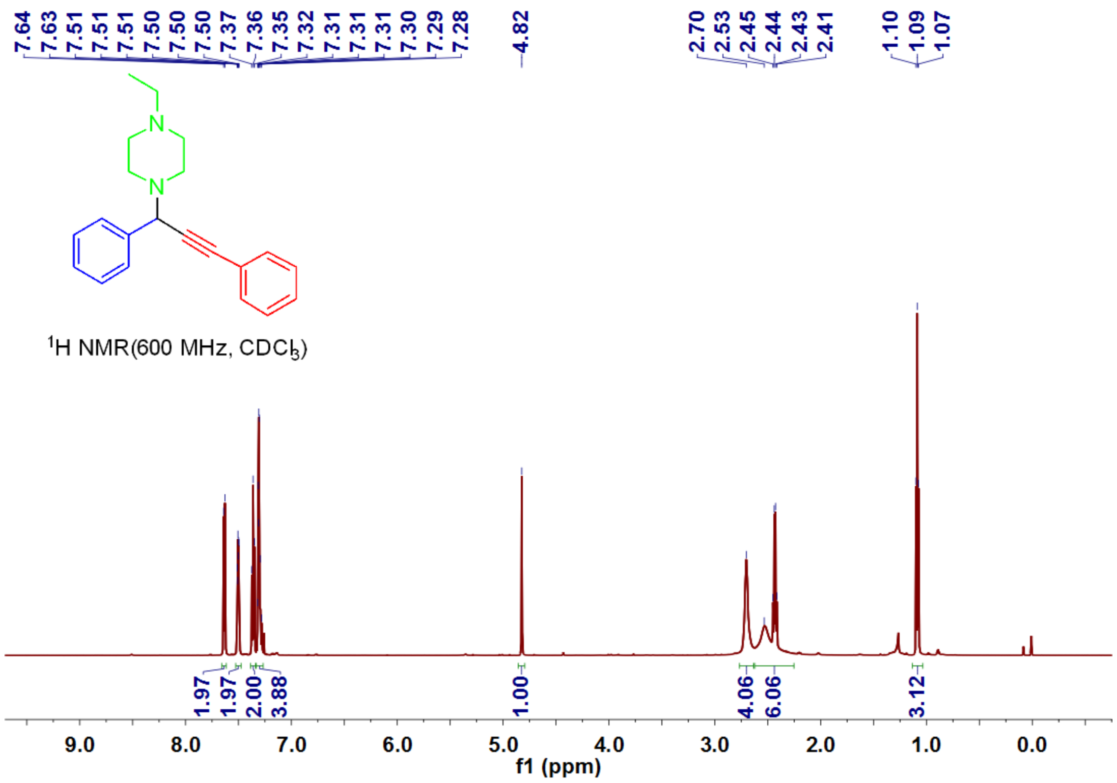

Supplementary Figure 182. **^{1}H NMR** spectrum of compound **4bm**.

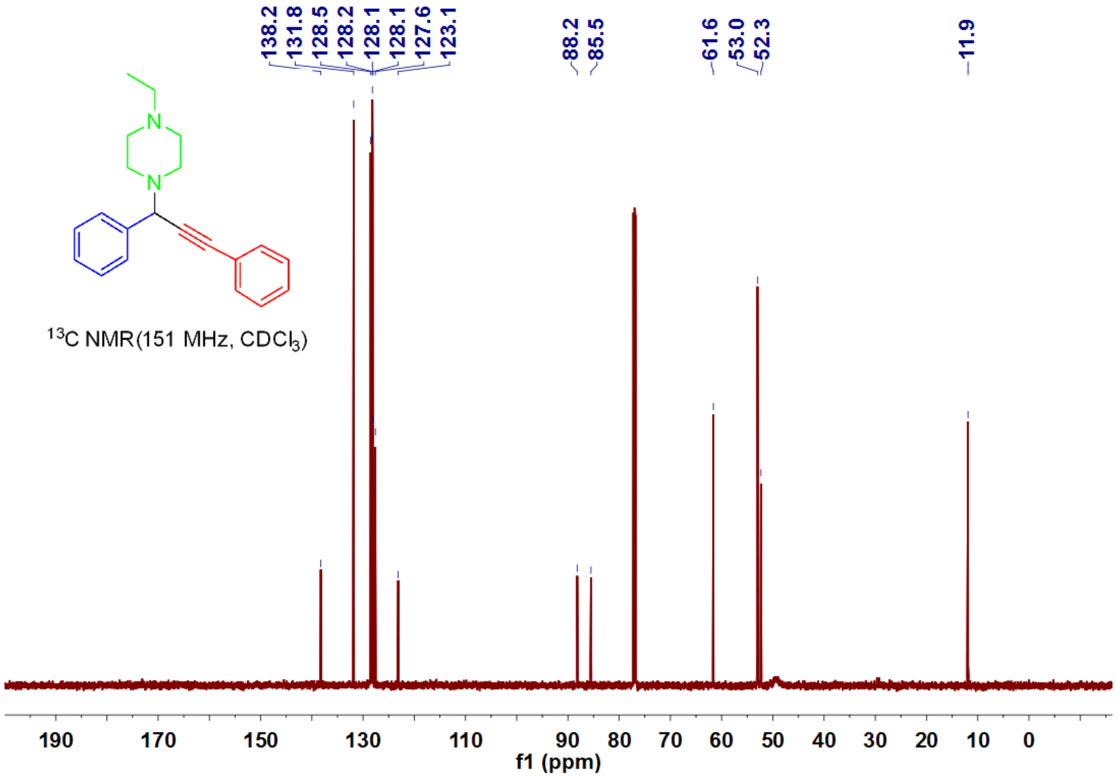

Supplementary Figure 183. **^{13}C NMR** spectrum of compound **4bm**.

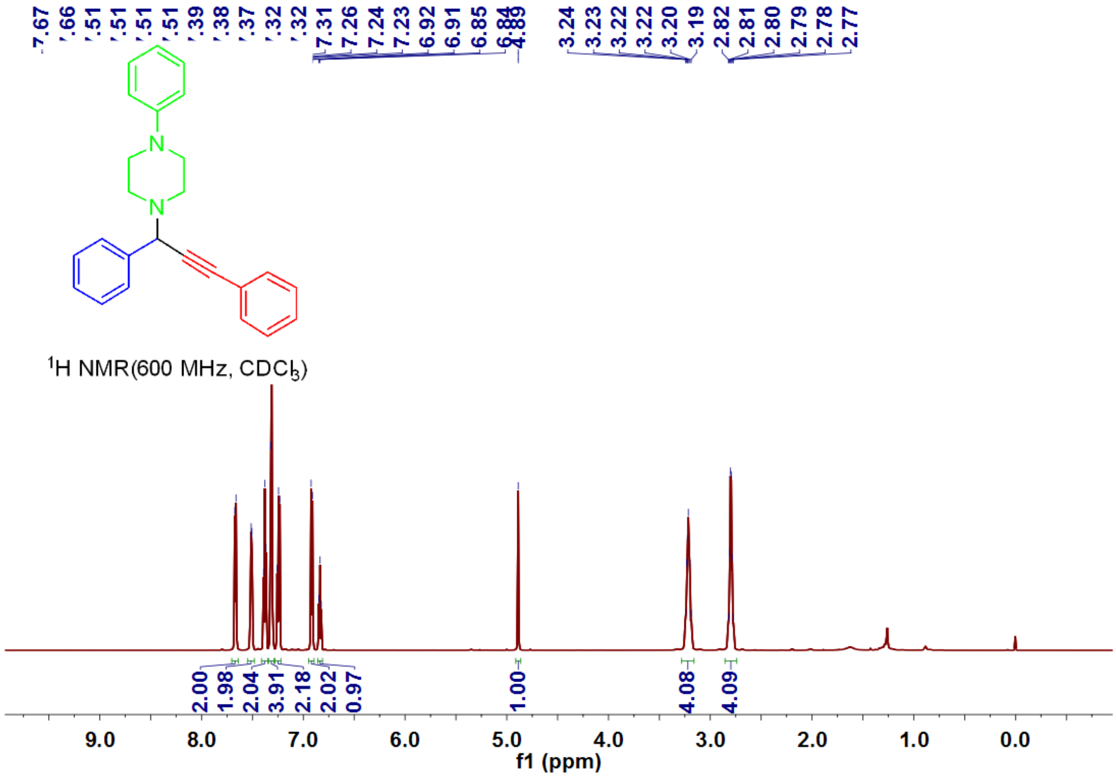

Supplementary Figure 184. ^{1}**H NMR** spectrum of compound **4bn**.

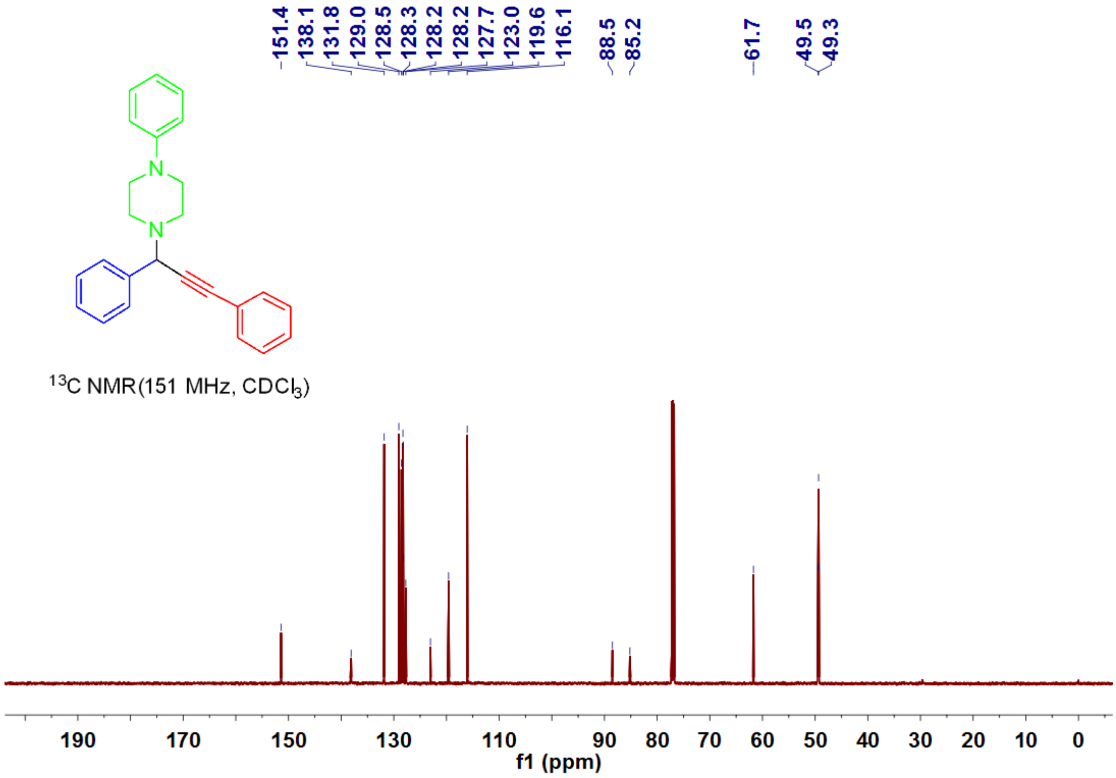

Supplementary Figure 185. ^{13}**C NMR** spectrum of compound **4bn**.

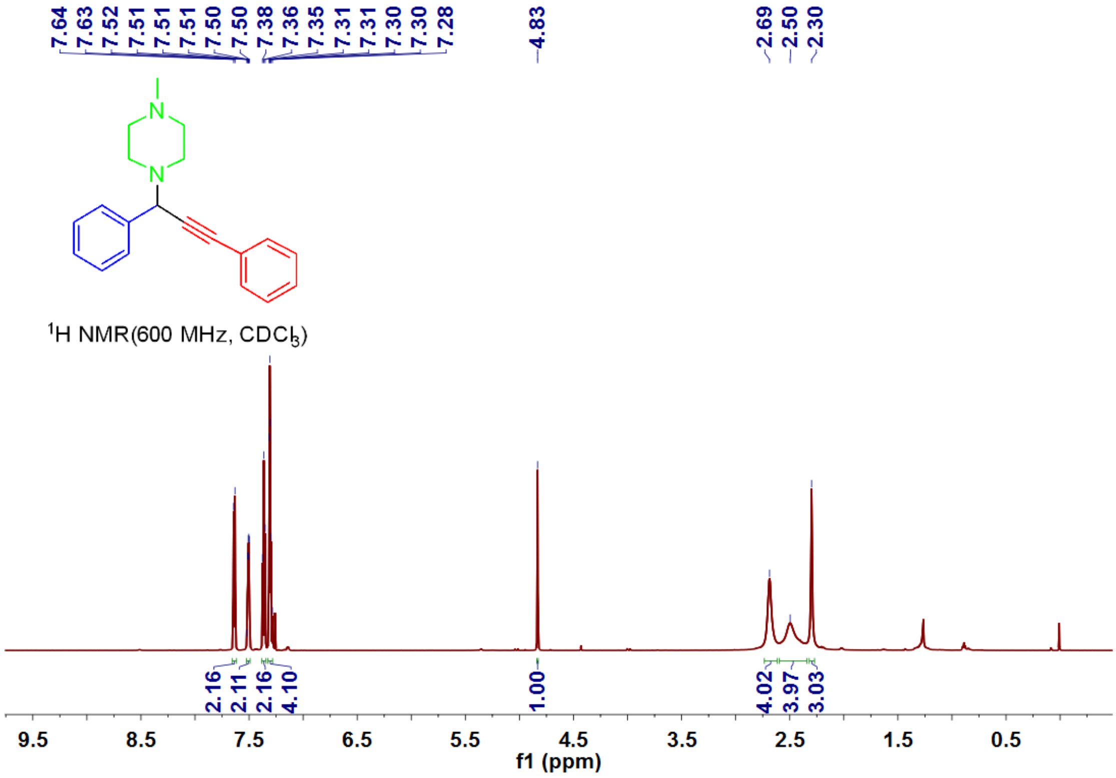

Supplementary Figure 186. ^{1}**H NMR** spectrum of compound **4bo**.

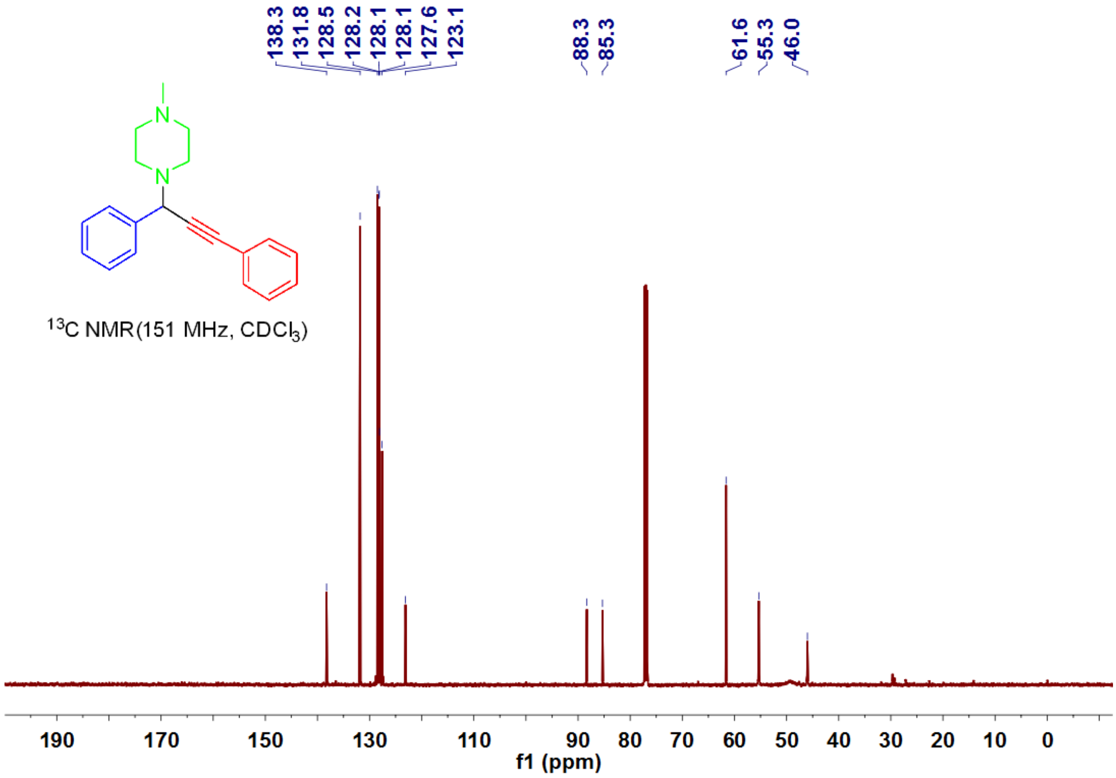

Supplementary Figure 187. ^{13}**C NMR** spectrum of compound **4bo**.

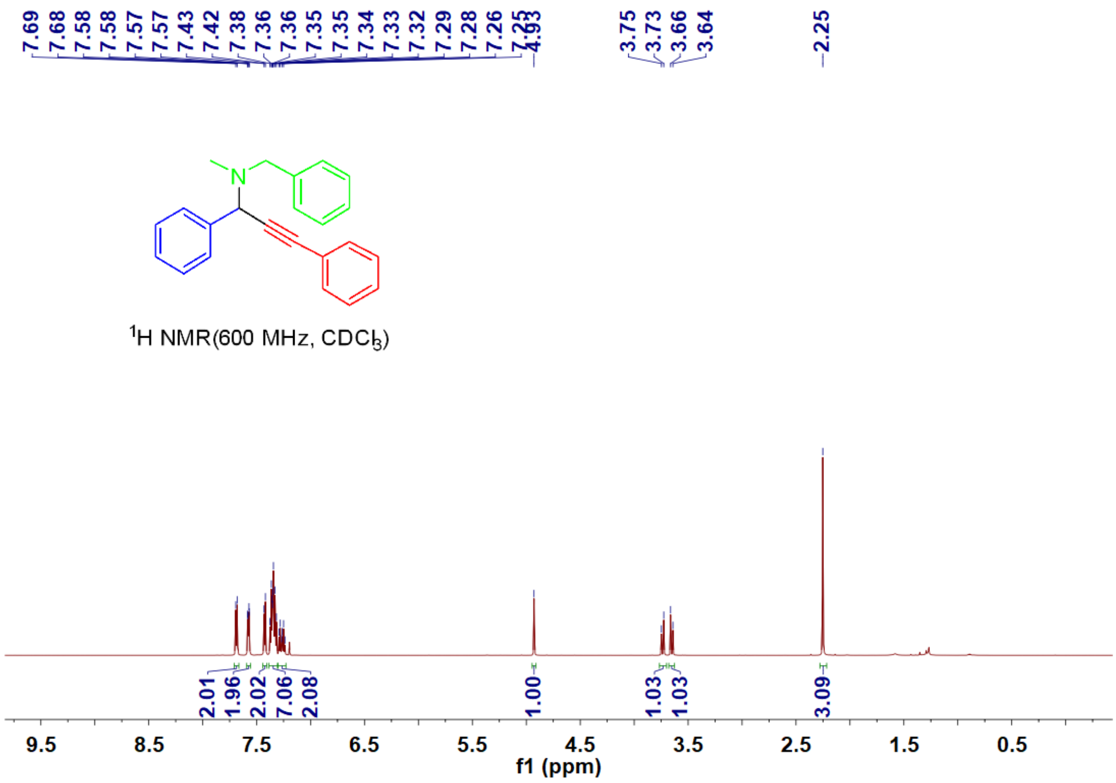

Supplementary Figure 188. ^{1}**H NMR** spectrum of compound **4bp**.

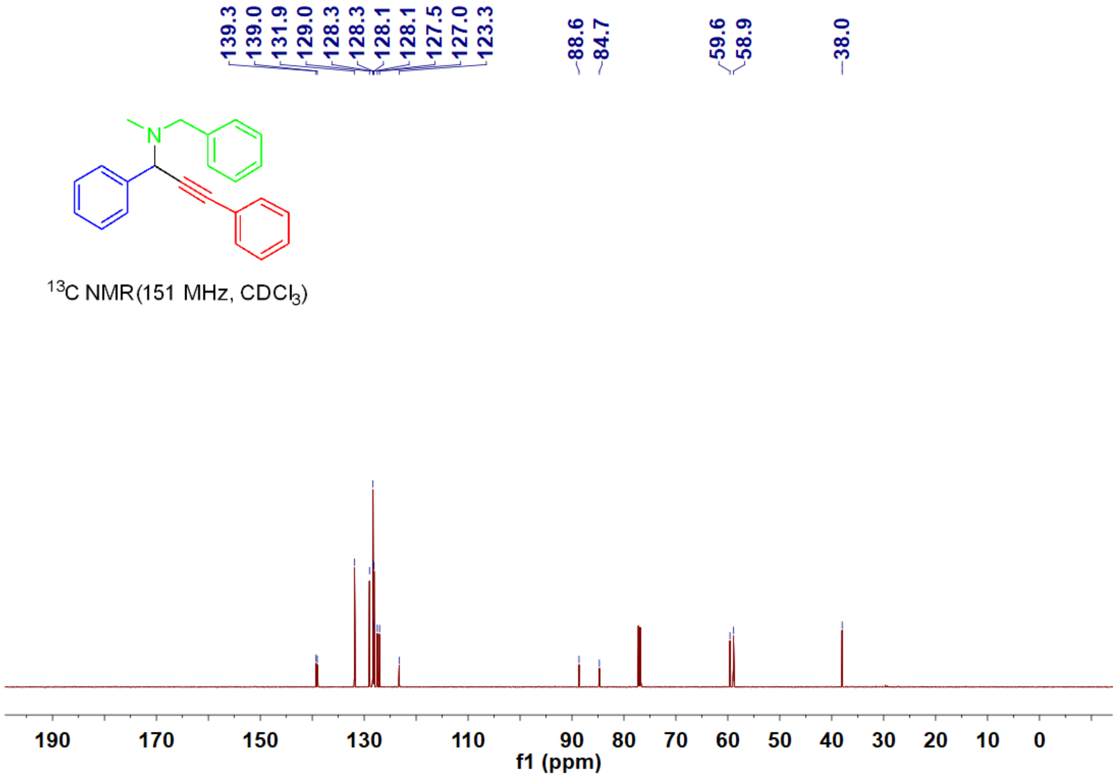

Supplementary Figure 189. ^{13}**C NMR** spectrum of compound **4bp**.

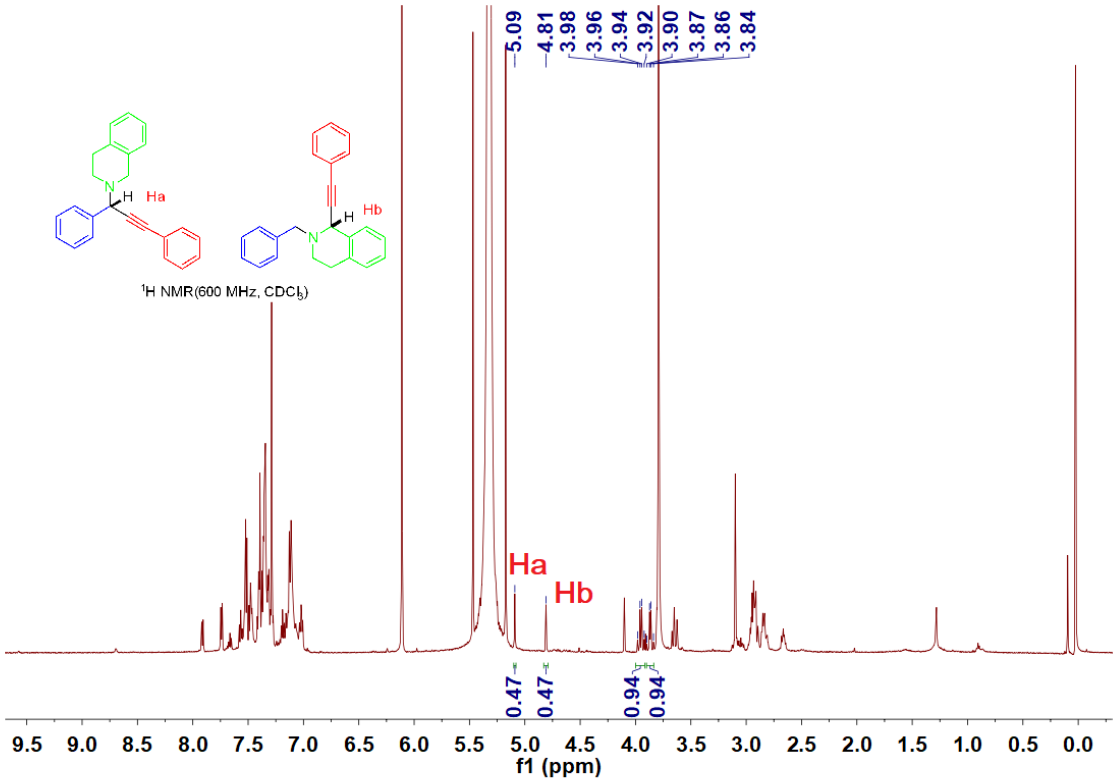

Supplementary Figure 190. ***In situ*** **^{1}H NMR** spectrum of compound **4bq** and **5a**.

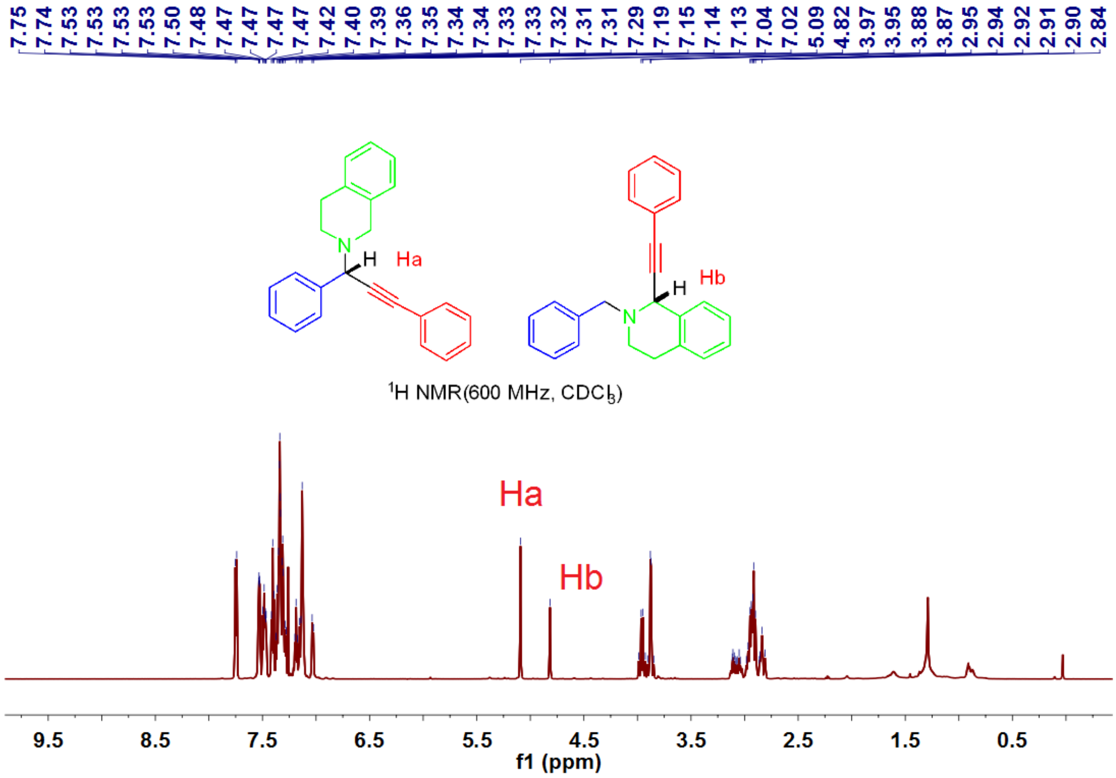

Supplementary Figure 191. **^{1}H NMR** spectrum of compound **4bq** and **5a**.

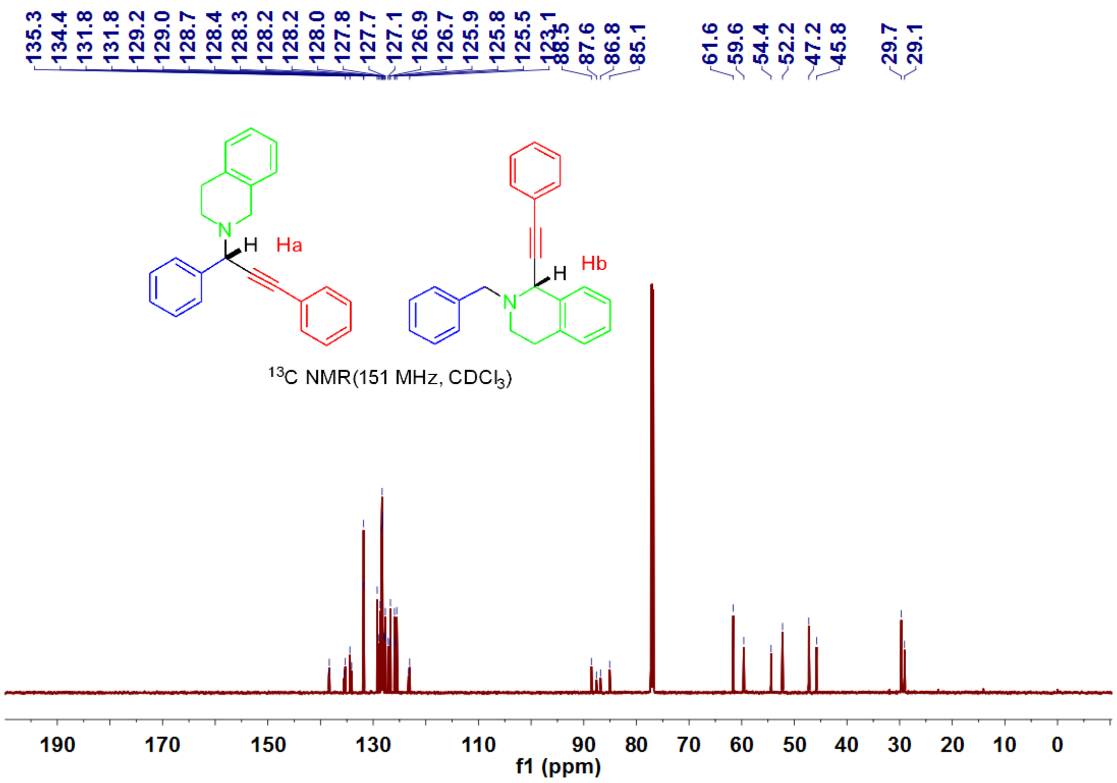

Supplementary Figure 192. ^{13}**C NMR** spectrum of compound **4bq** and **5a**.

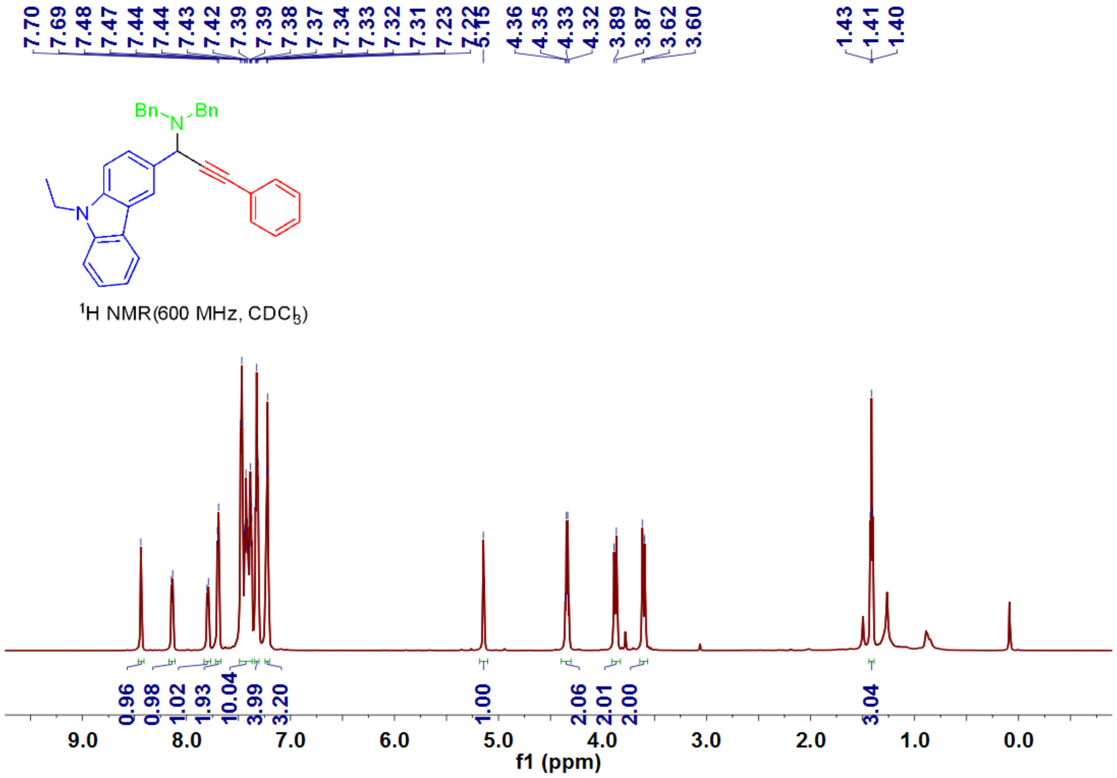

Supplementary Figure 193. ^{1}**H NMR** spectrum of compound **4br**.

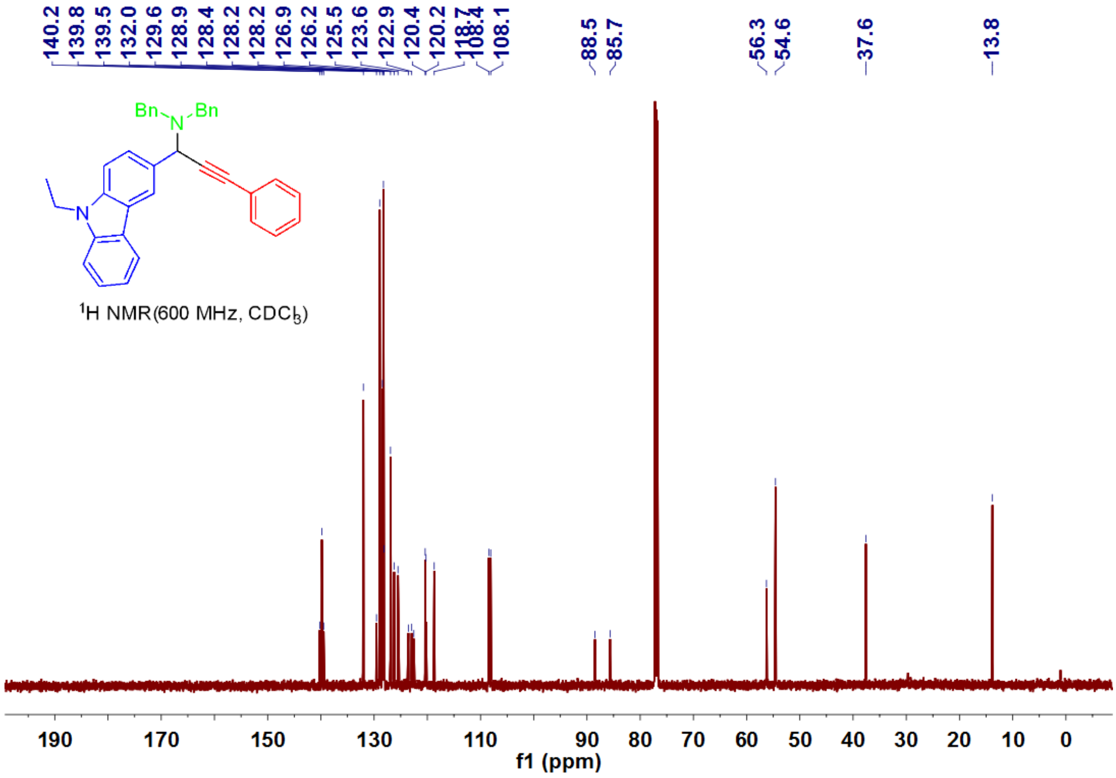

Supplementary Figure 194. ^{13}**C NMR** spectrum of compound **4br**.

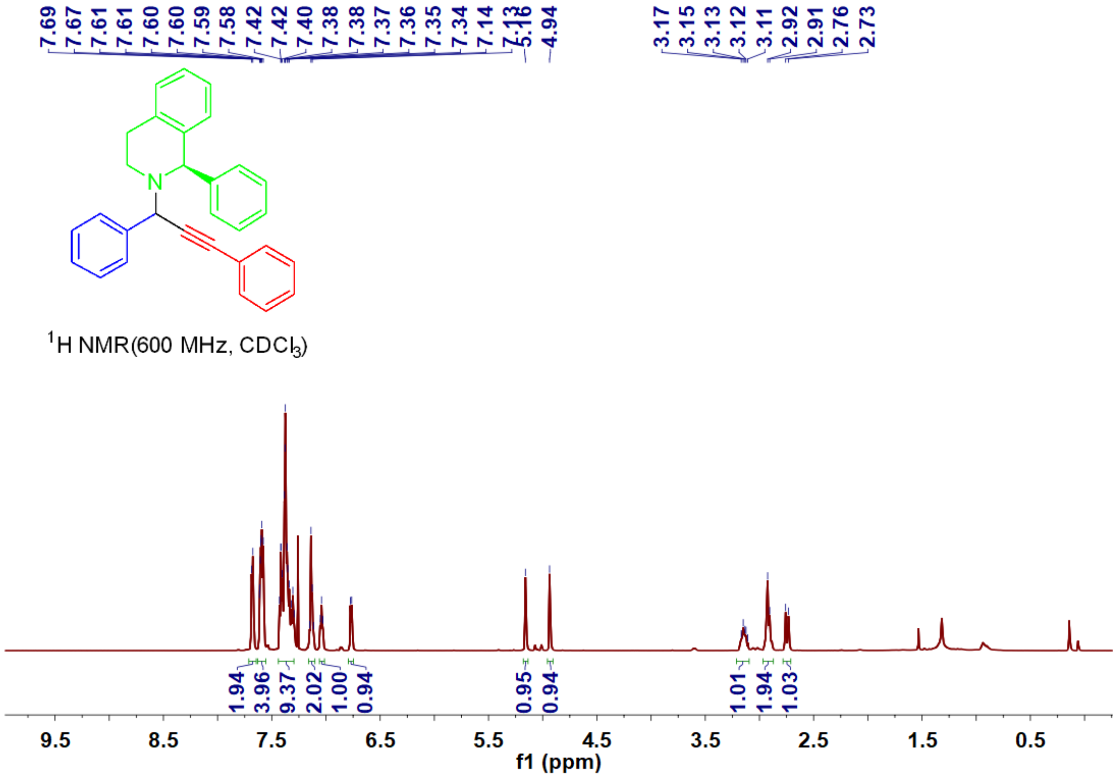

Supplementary Figure 195. ^{1}**H NMR** spectrum of compound **4bs**.

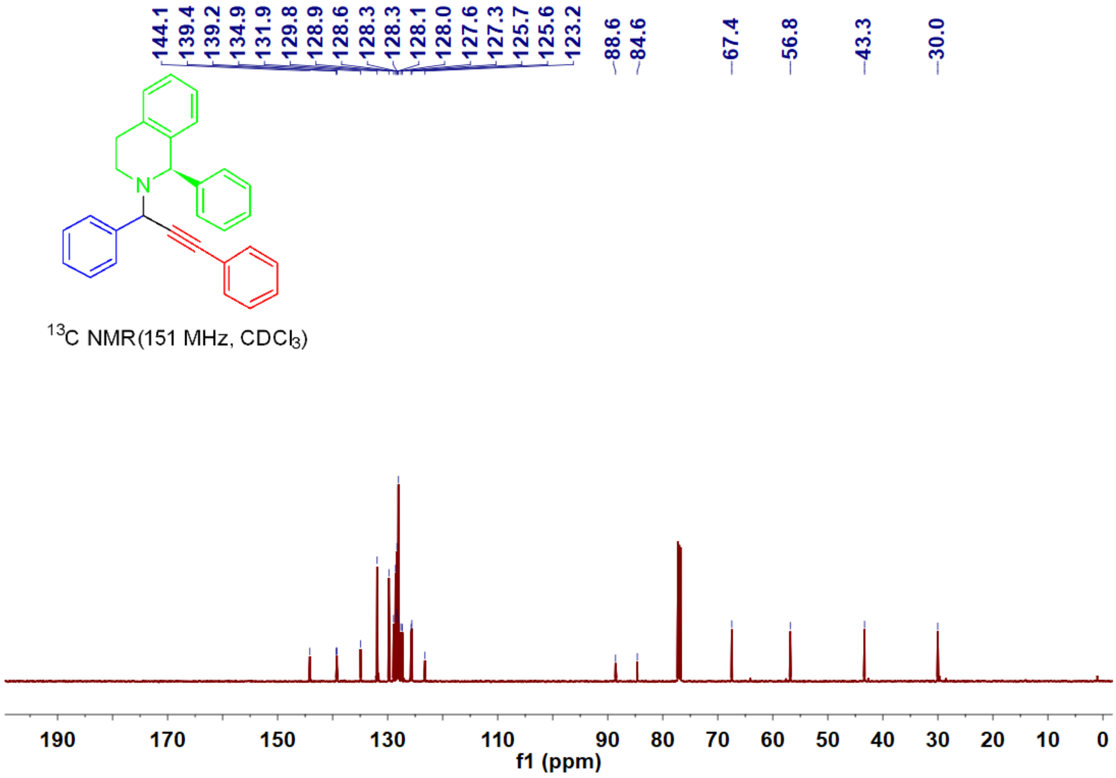

Supplementary Figure 196. ^{13}**C NMR** spectrum of compound **4bs**.

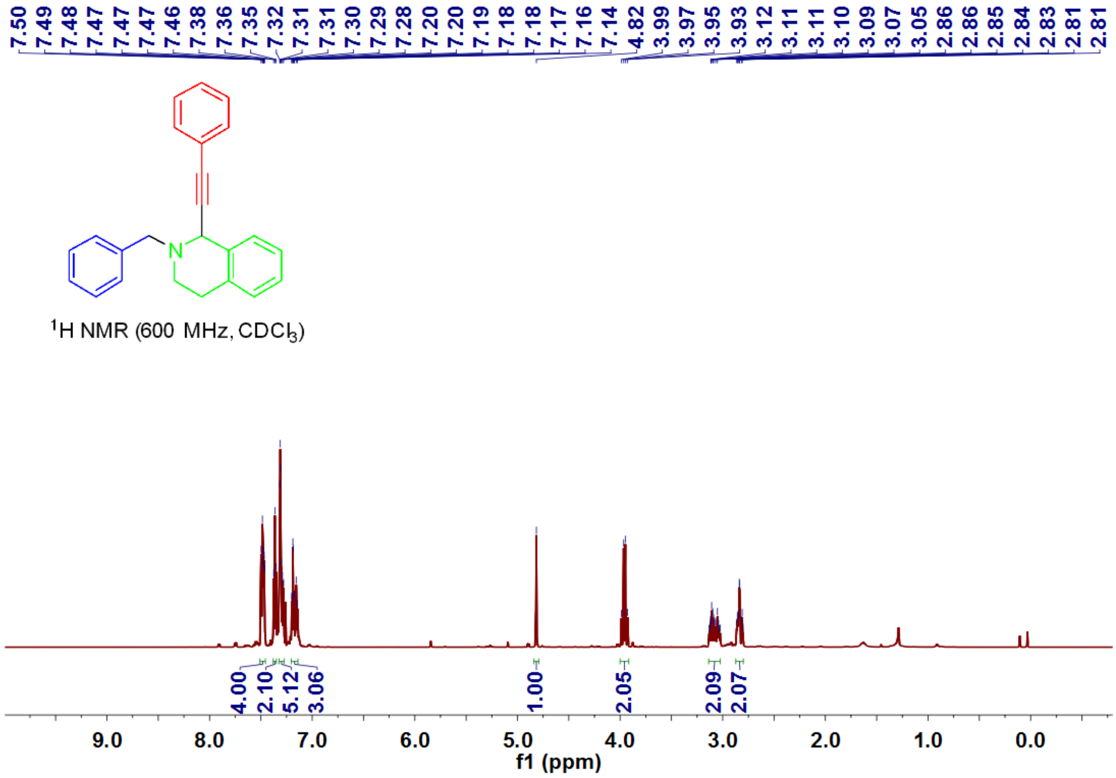

Supplementary Figure 197. ^{1}**H NMR** spectrum of compound **5a**.

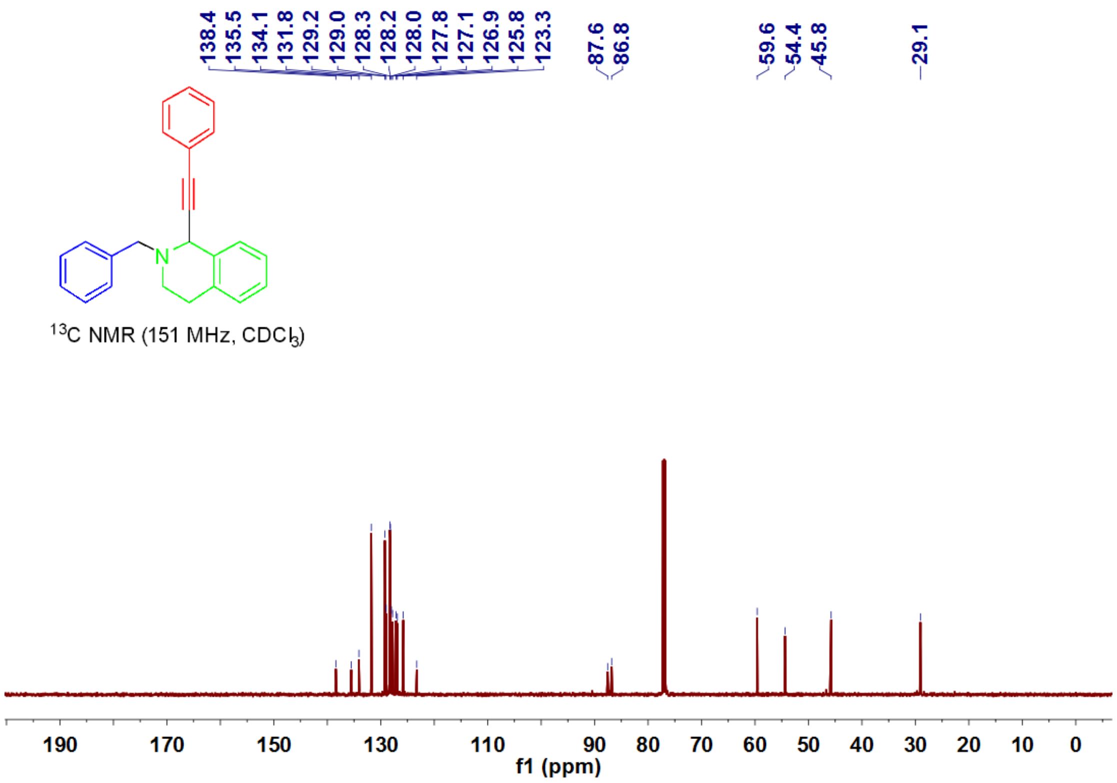

Supplementary Figure 198. ^{13}**C NMR** spectrum of compound **5a**.

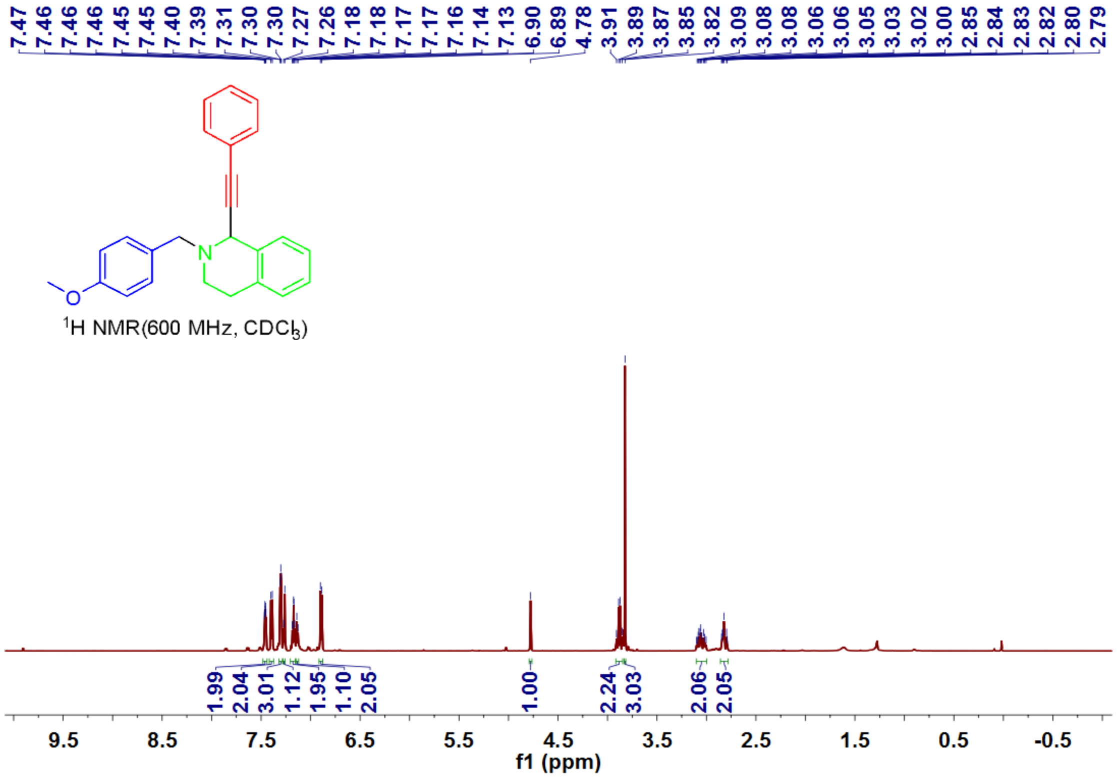

Supplementary Figure 199. ^{1}**H NMR** spectrum of compound **5b**.

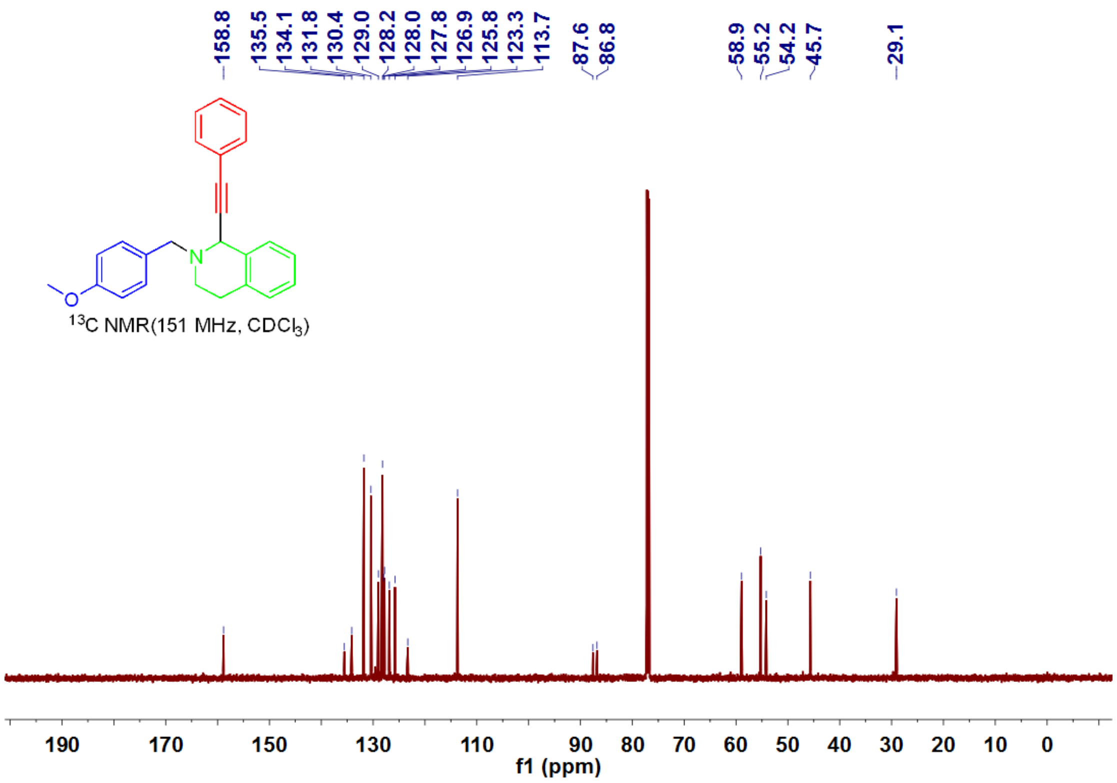

Supplementary Figure 200. ^{13}**C NMR** spectrum of compound **5b**.

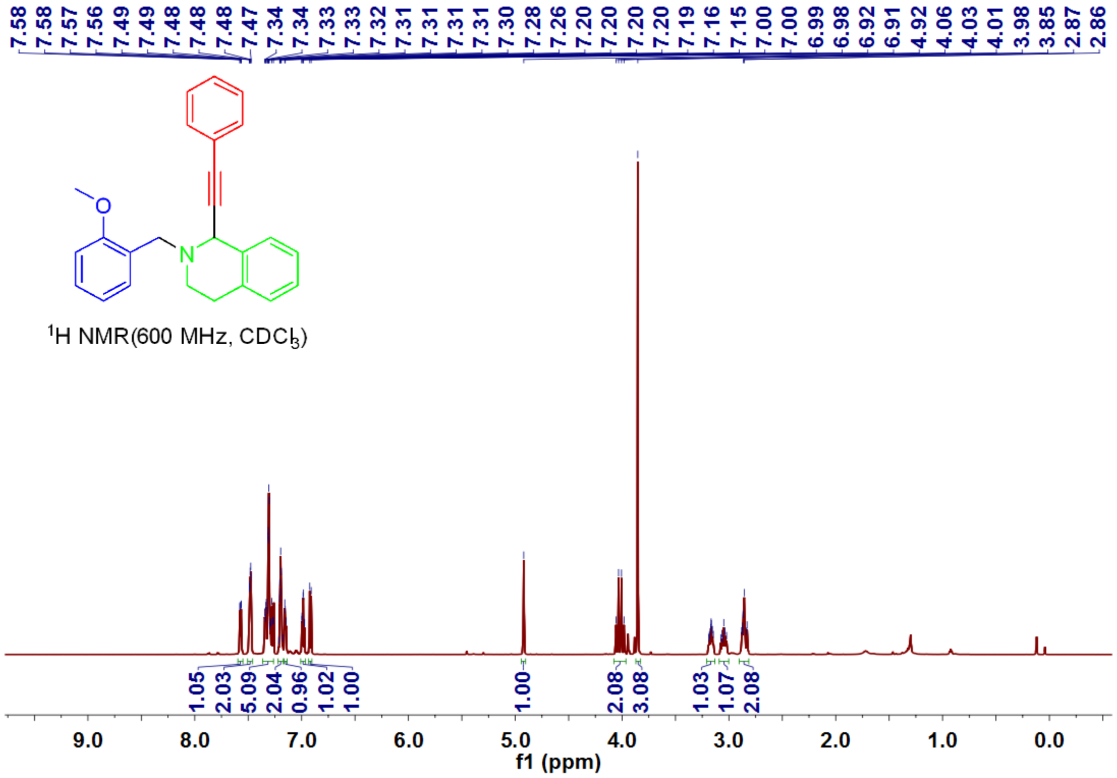

Supplementary Figure 201. ^{1}**H NMR** spectrum of compound **5c**.

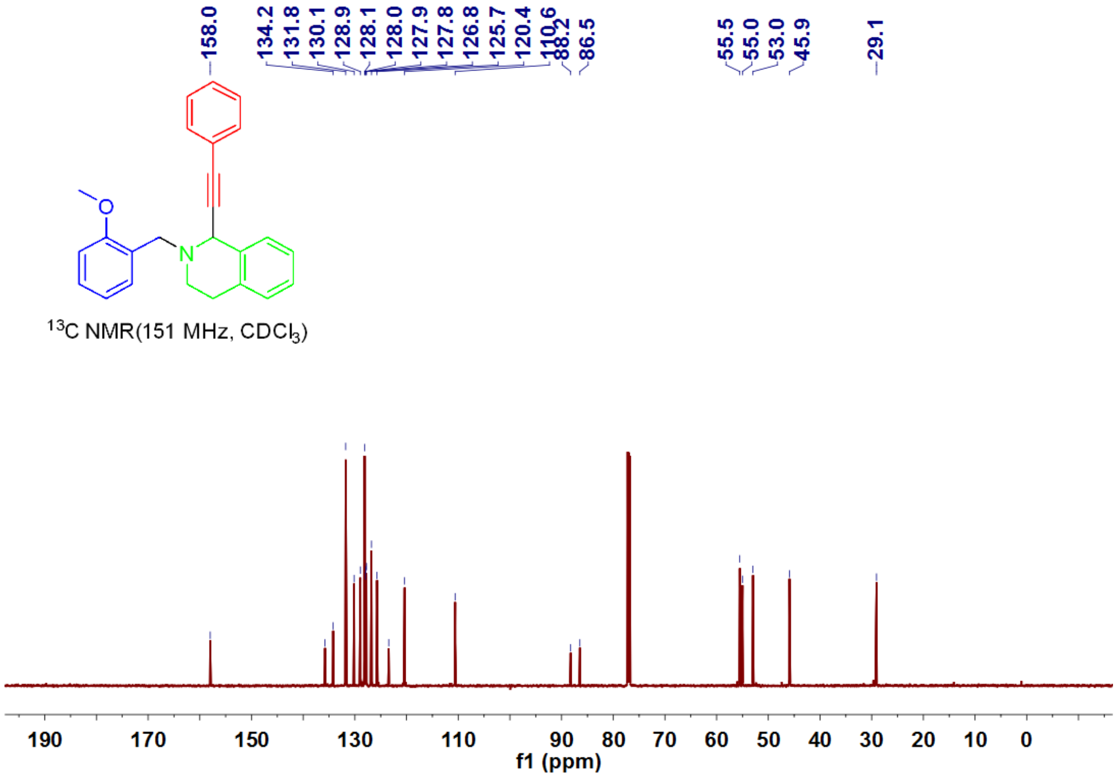

Supplementary Figure 202. ^{13}C NMR spectrum of compound **5c**.

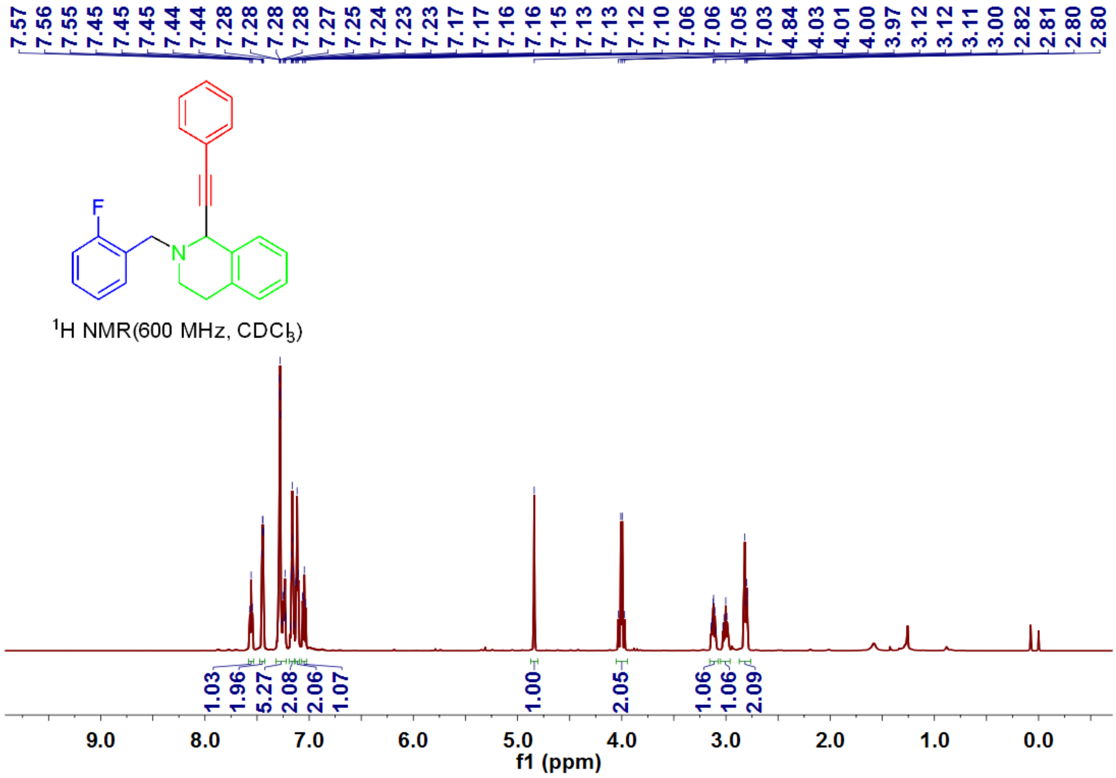

Supplementary Figure 203. ^{1}H NMR spectrum of compound **5d**.

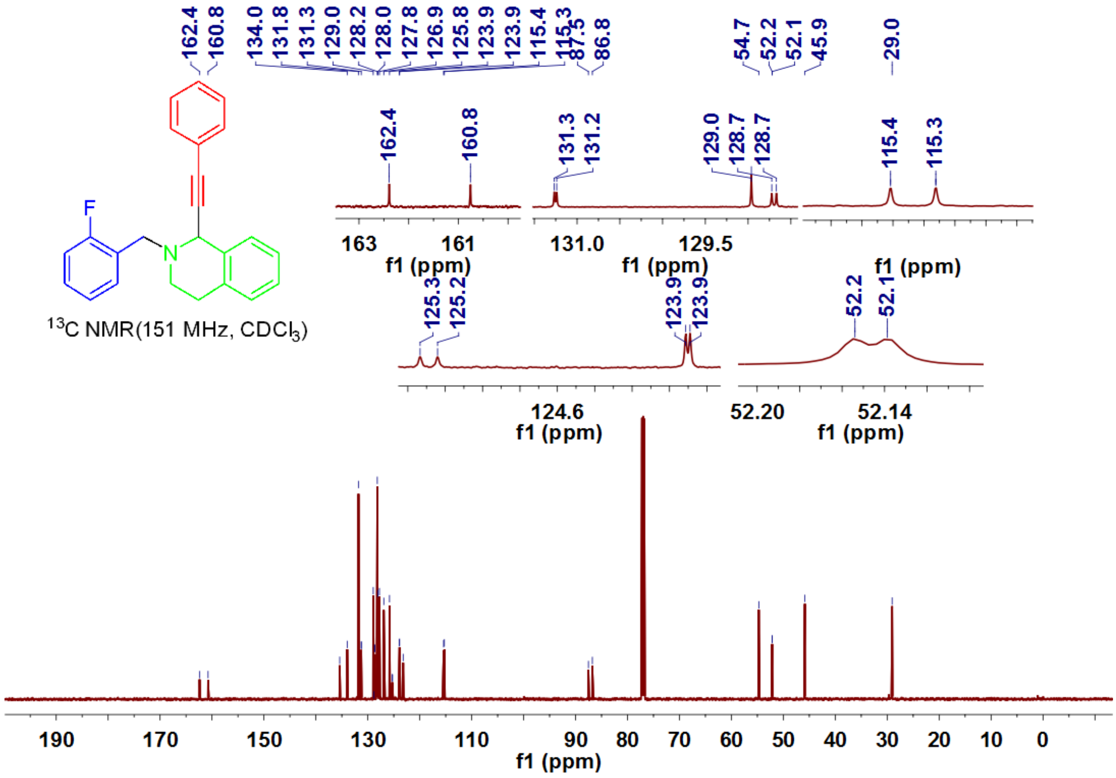

Supplementary Figure 204. ^{13}**C NMR** spectrum of compound **5d**.

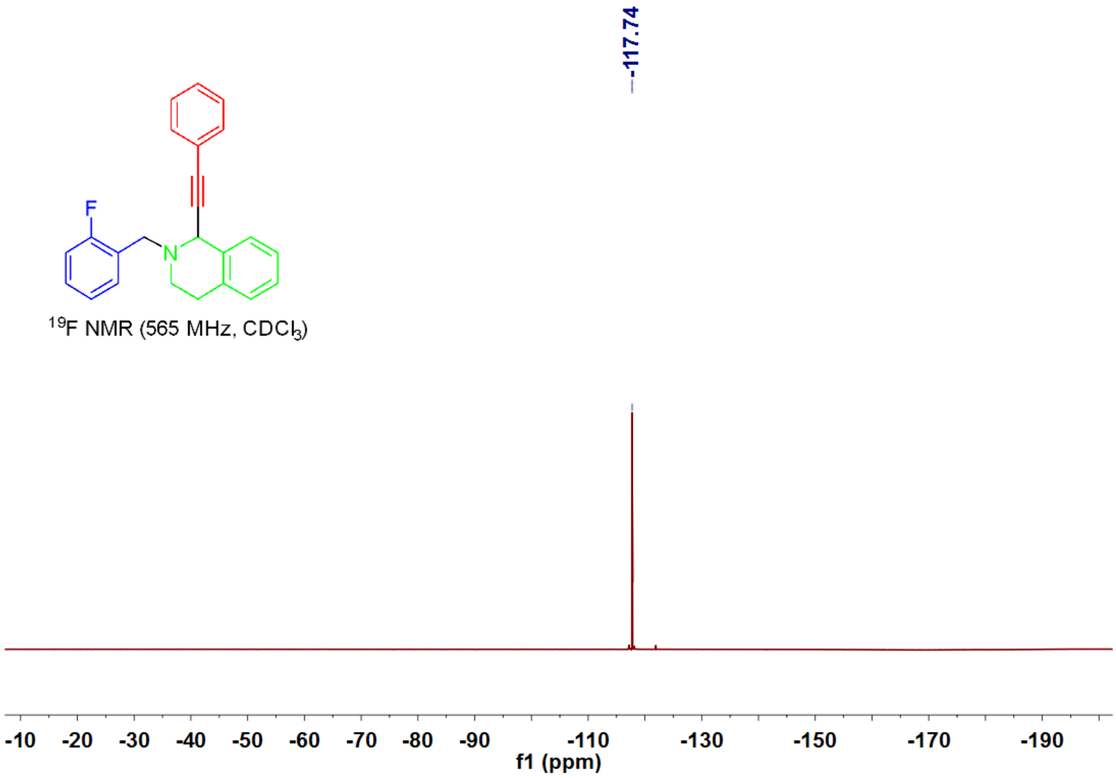

Supplementary Figure 205. ^{19}**F NMR** spectrum of compound **5d**.

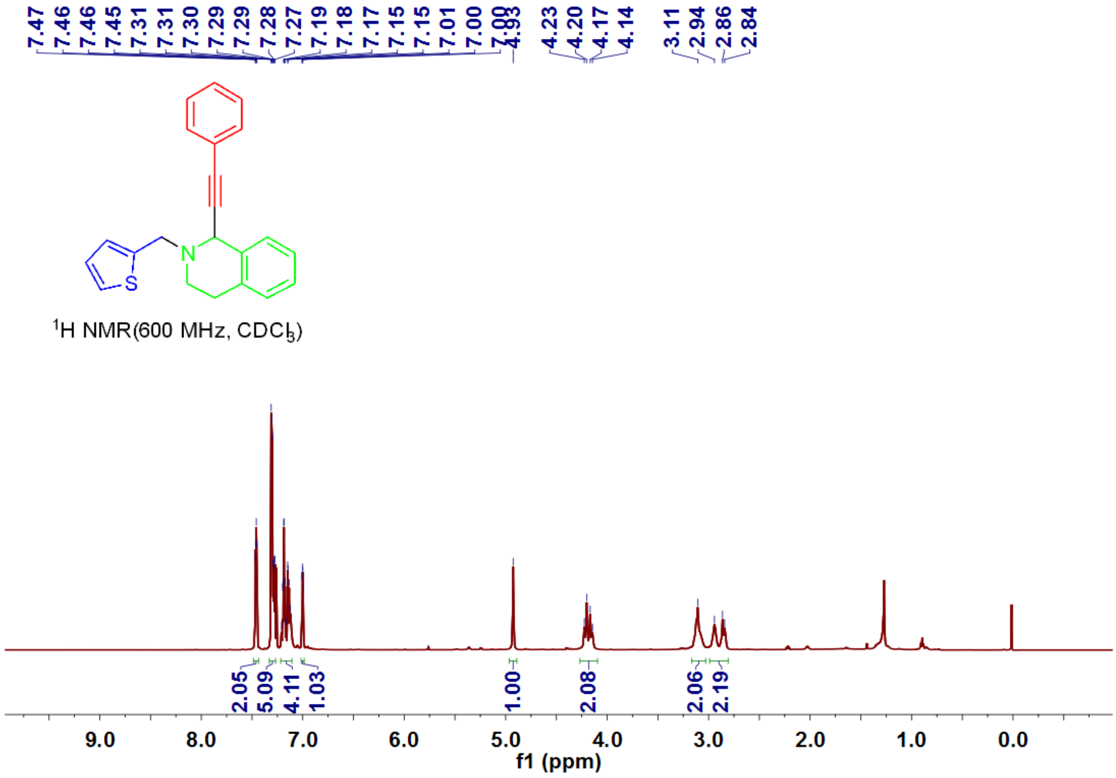

Supplementary Figure 206. ^{1}**H NMR** spectrum of compound **5e**.

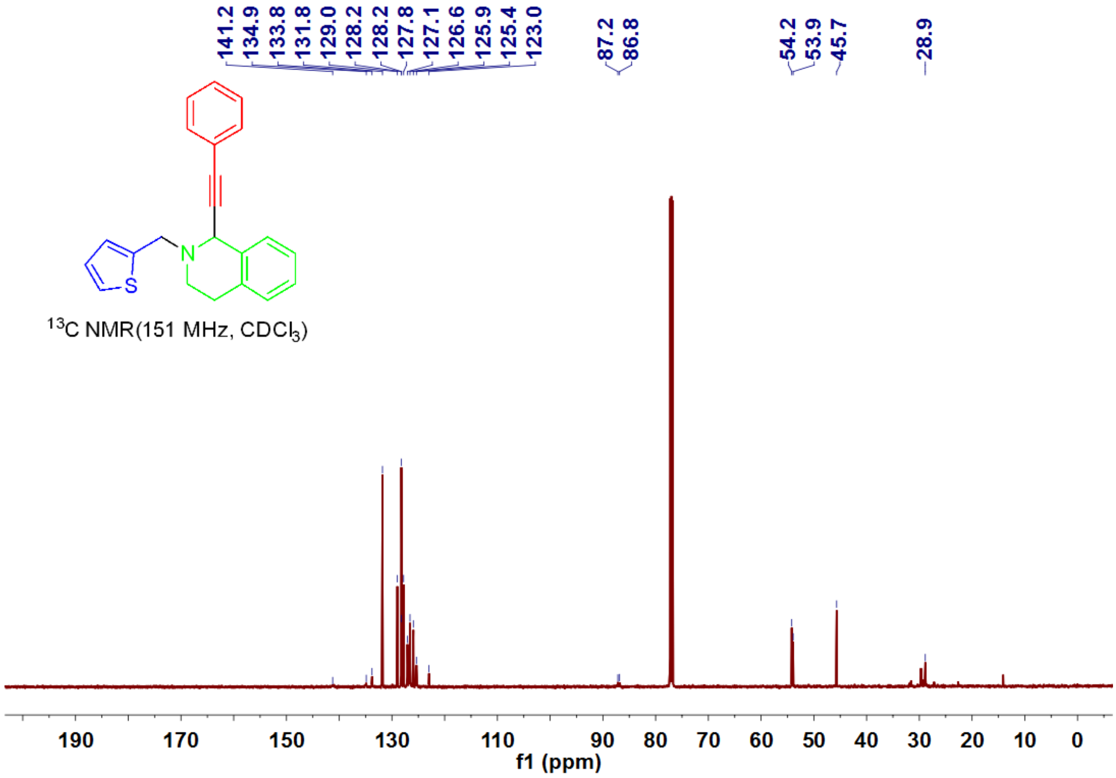

Supplementary Figure 207. ^{13}**C NMR** spectrum of compound **5e**.

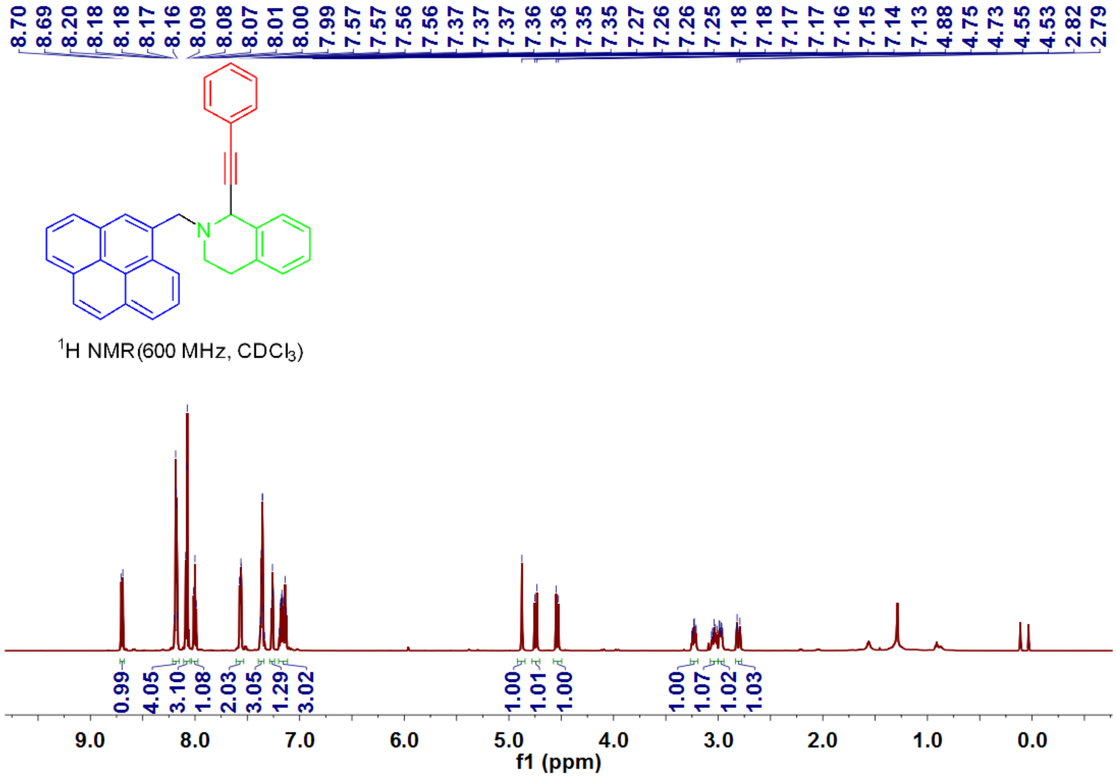

Supplementary Figure 208. ^{1}**H NMR** spectrum of compound **5f**.

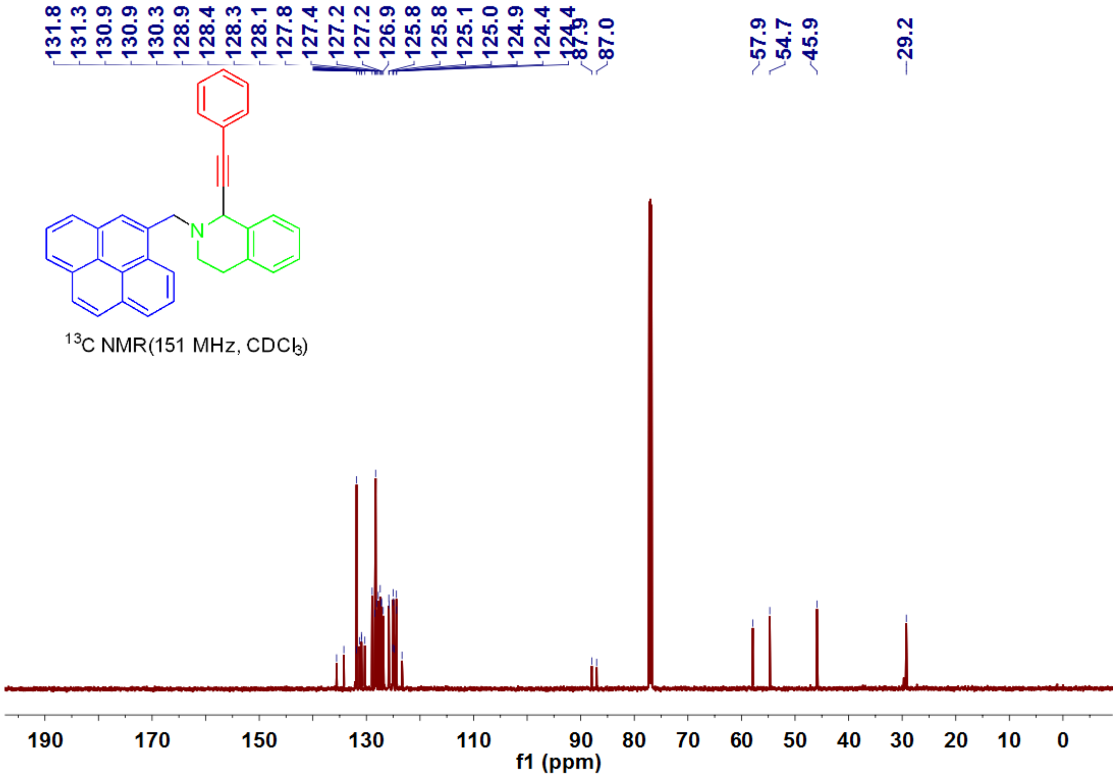

Supplementary Figure 209. ^{13}**C NMR** spectrum of compound **5f**.

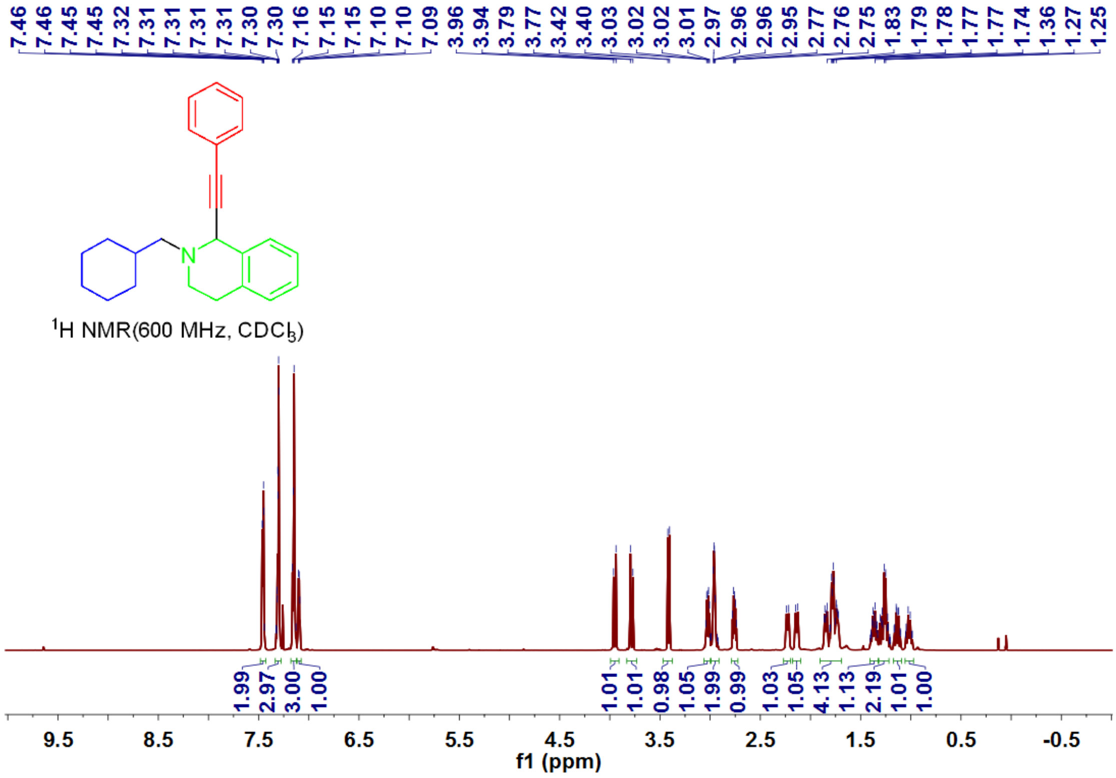

Supplementary Figure 210. ^{1}**H NMR** spectrum of compound **5g**.

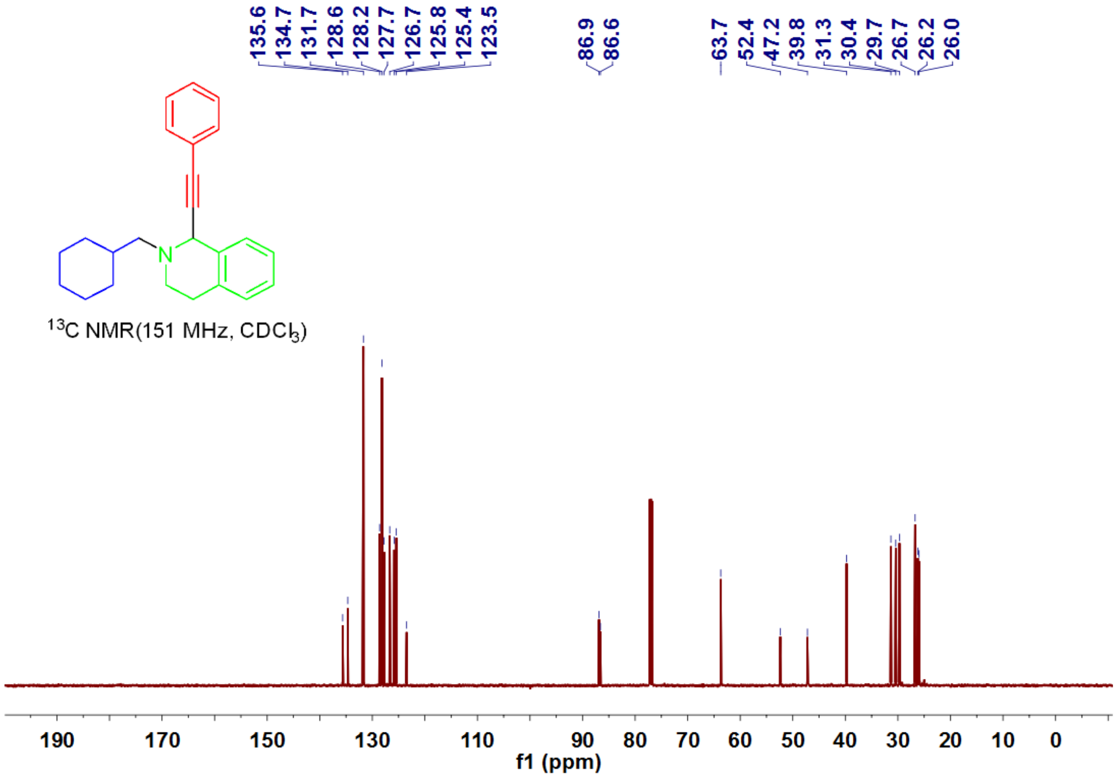

Supplementary Figure 211. ^{13}**C NMR** spectrum of compound **5g**.

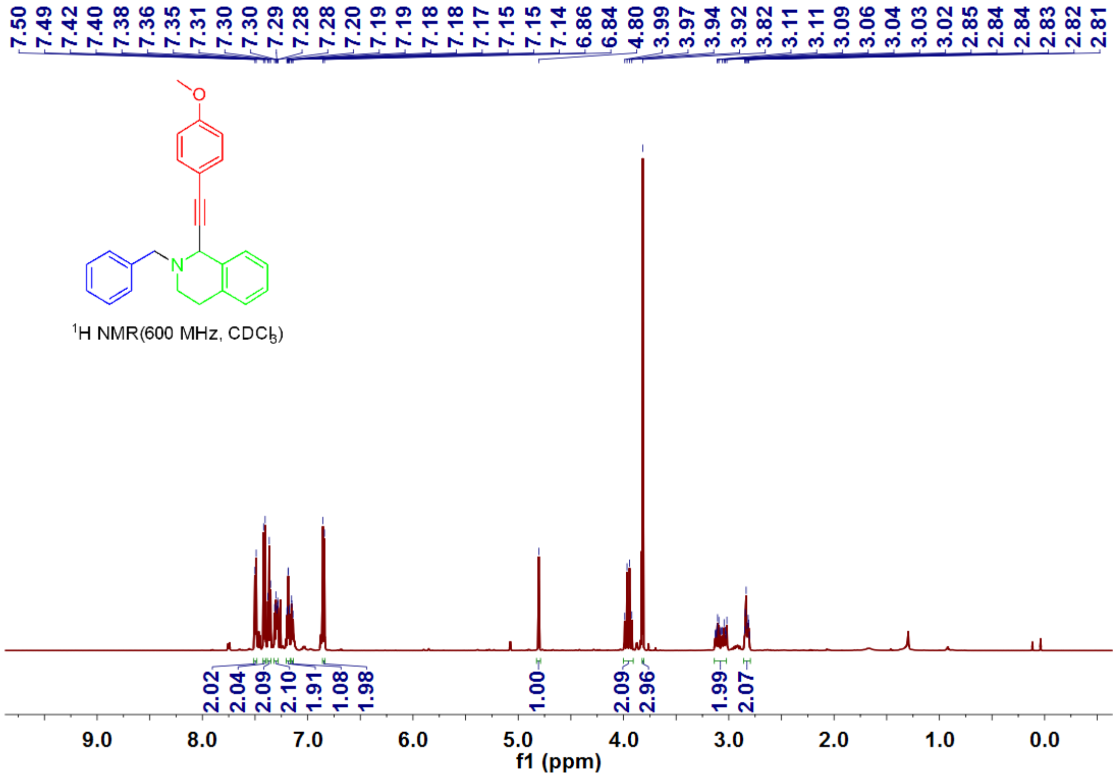

Supplementary Figure 212. ^{1}**H NMR** spectrum of compound **5h**.

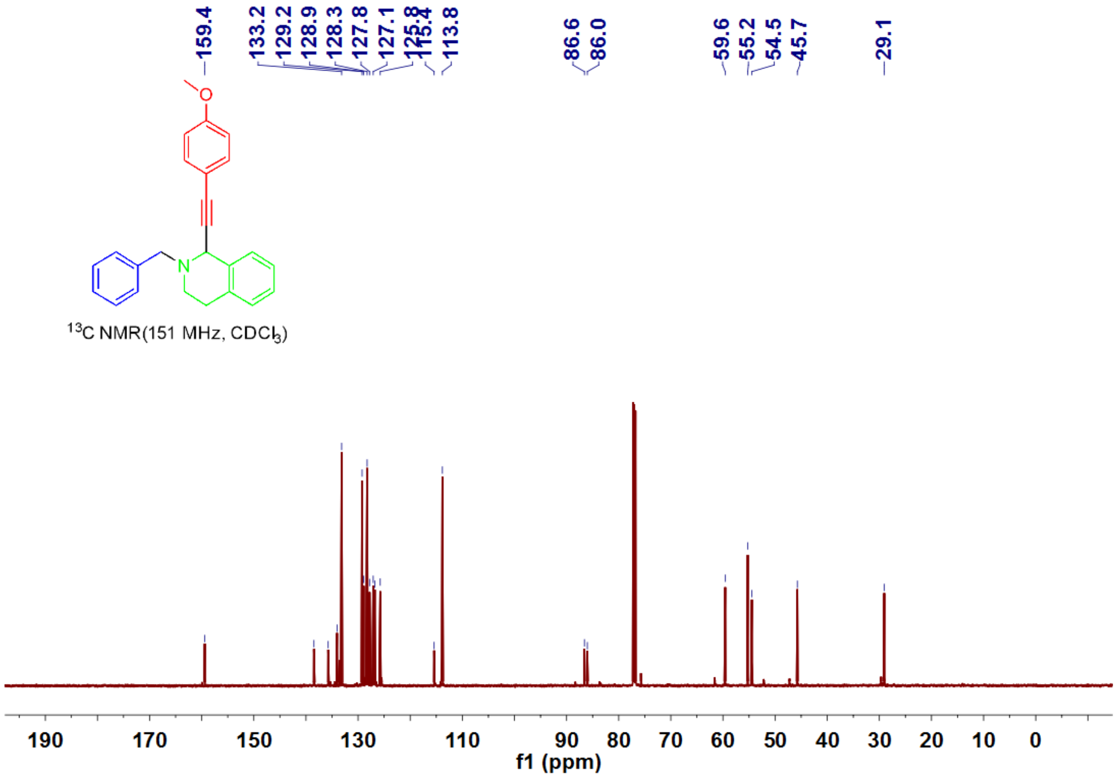

Supplementary Figure 213. ^{13}**C NMR** spectrum of compound **5h**.

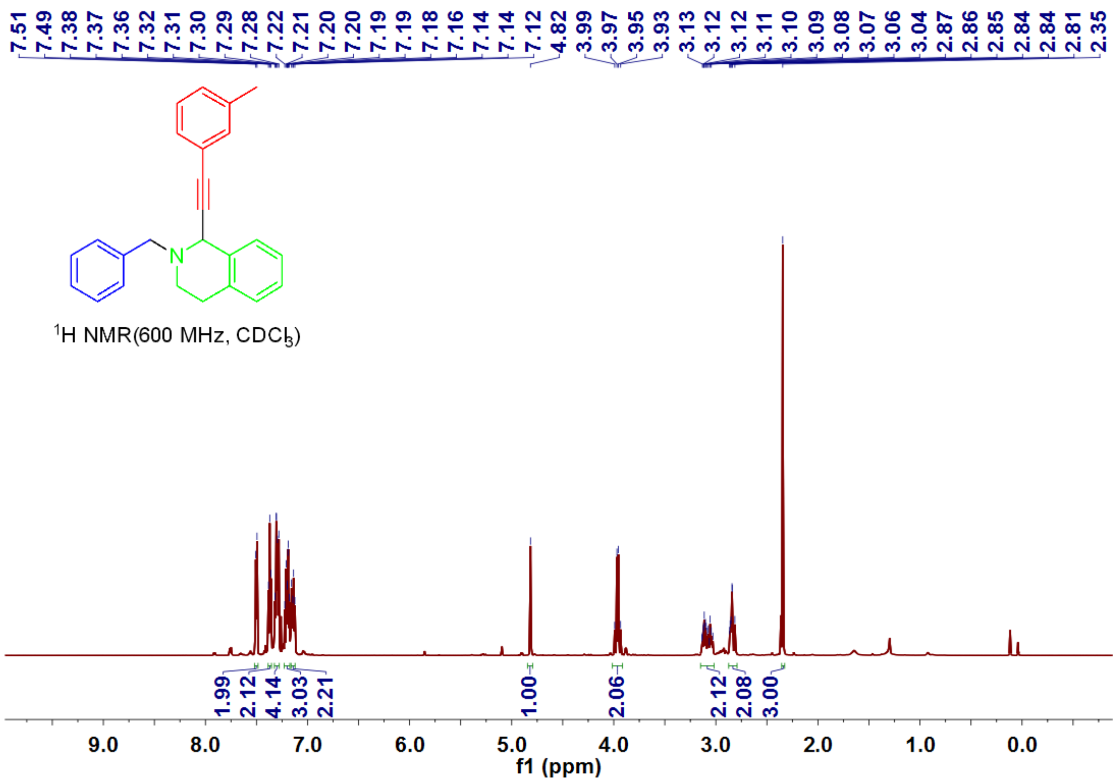

Supplementary Figure 214. **^{1}H NMR** spectrum of compound **5i**.

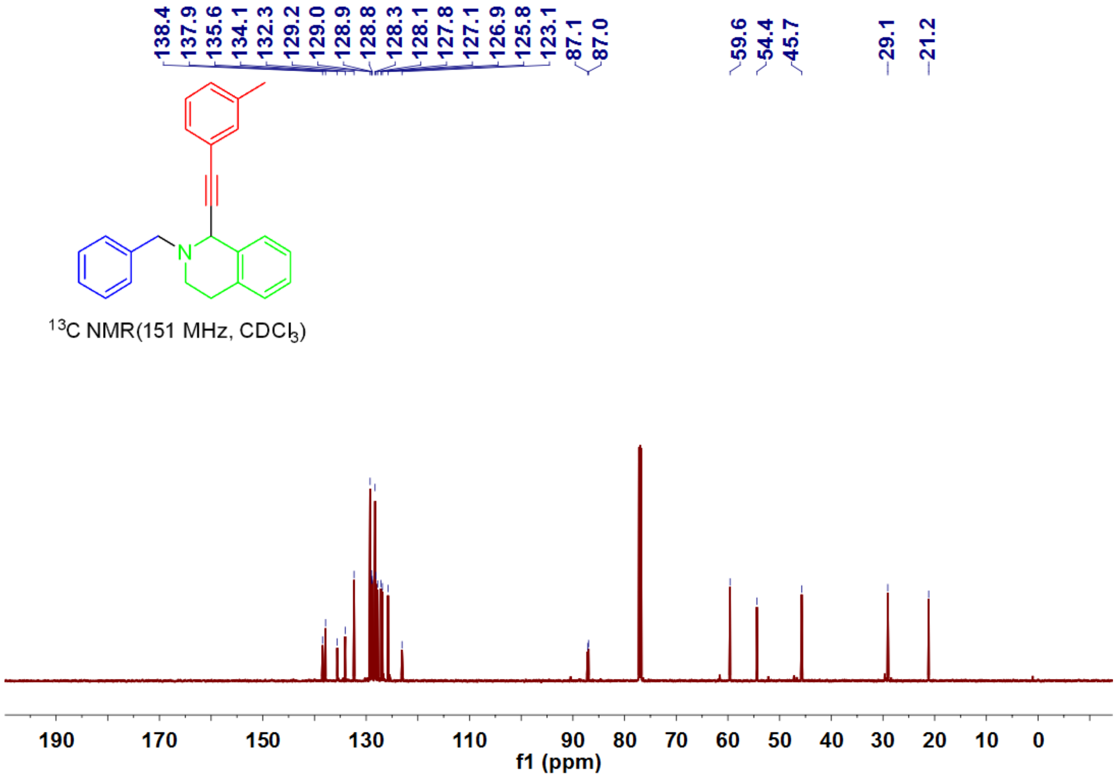

Supplementary Figure 215. **^{13}C NMR** spectrum of compound **5i**.

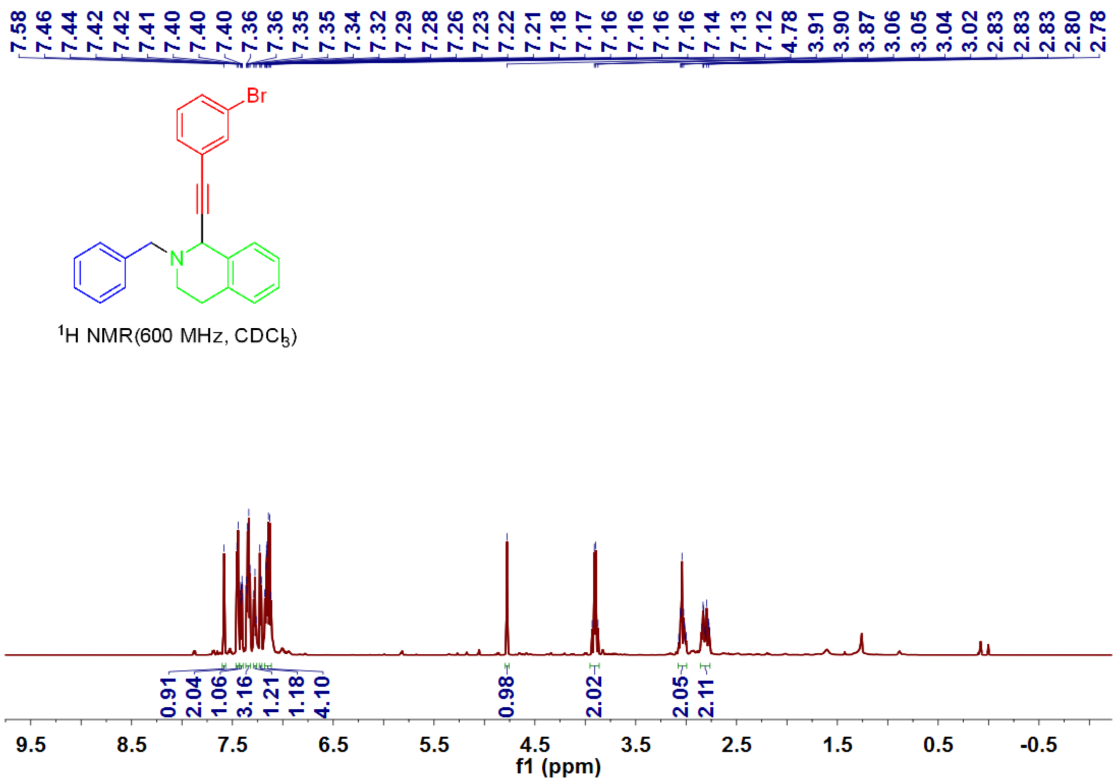

Supplementary Figure 216. ^{1}**H NMR** spectrum of compound **5j**.

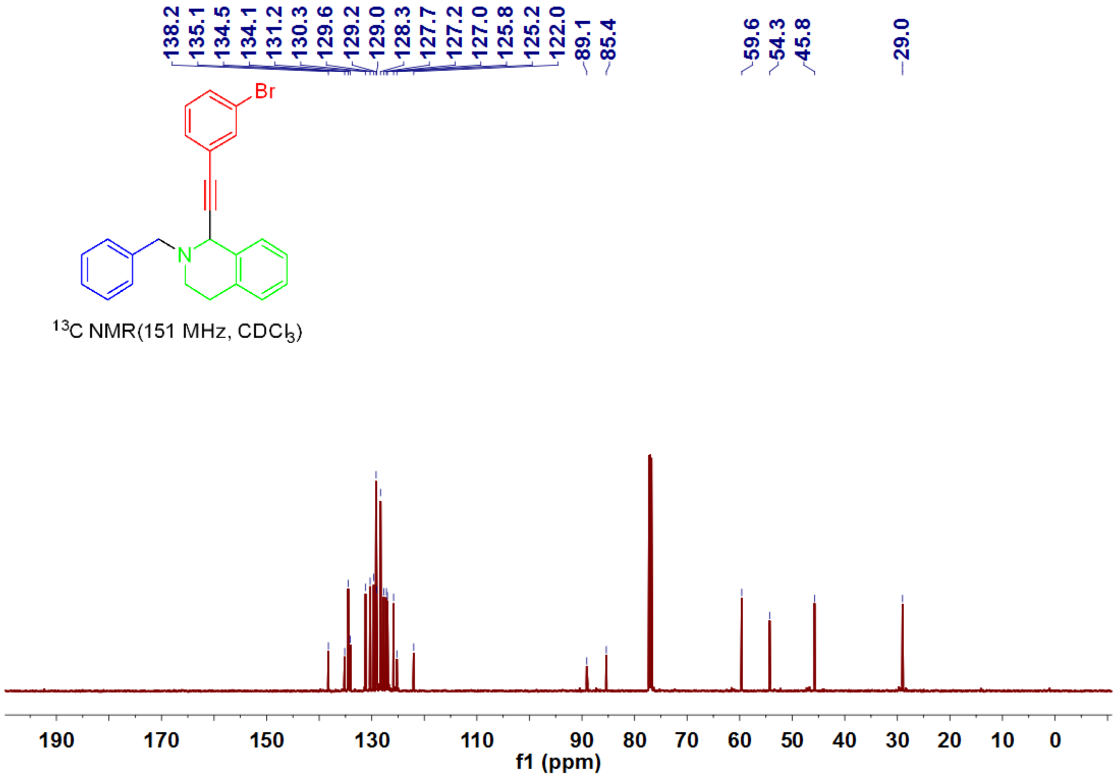

Supplementary Figure 217. ^{13}**C NMR** spectrum of compound **5j**.

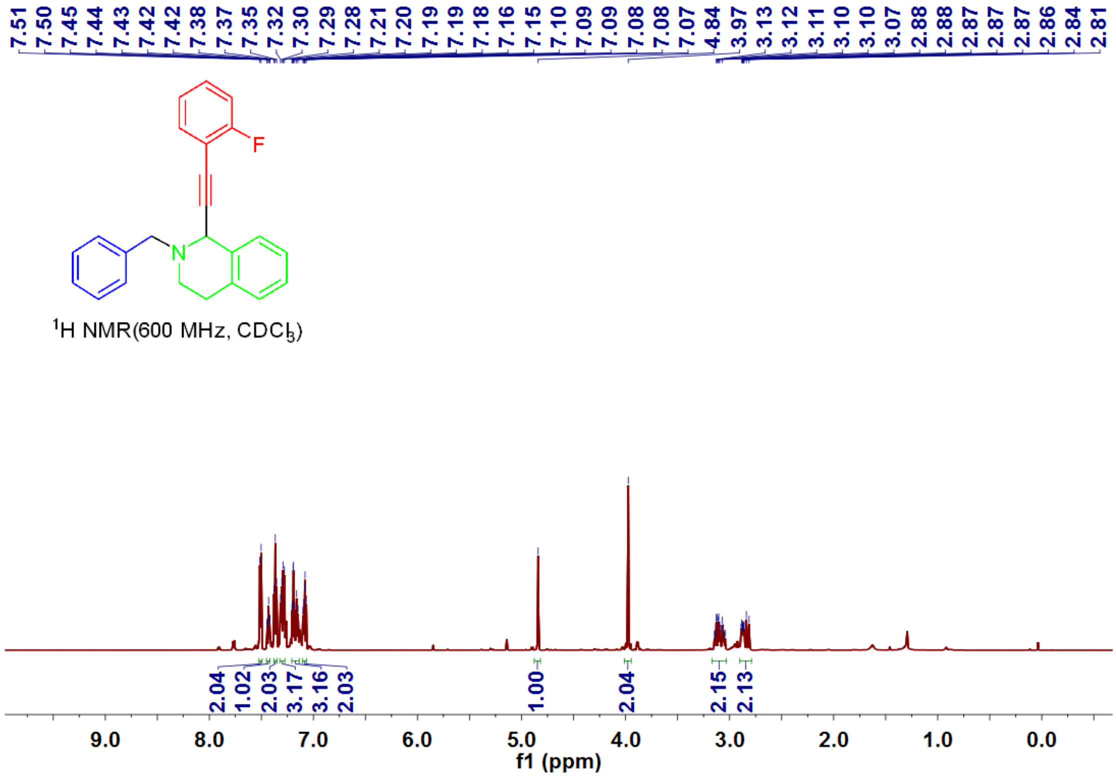

Supplementary Figure 218. ^{1}**H NMR** spectrum of compound **5k**.

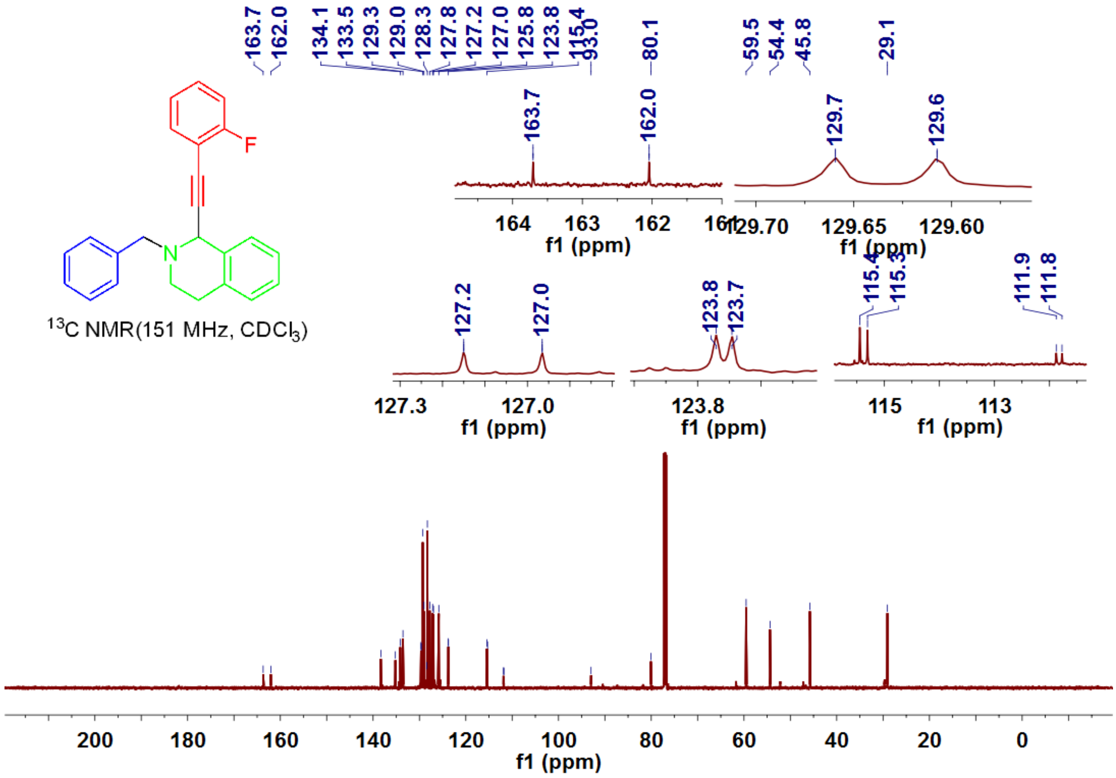

Supplementary Figure 219. ^{13}**C NMR** spectrum of compound **5k**.

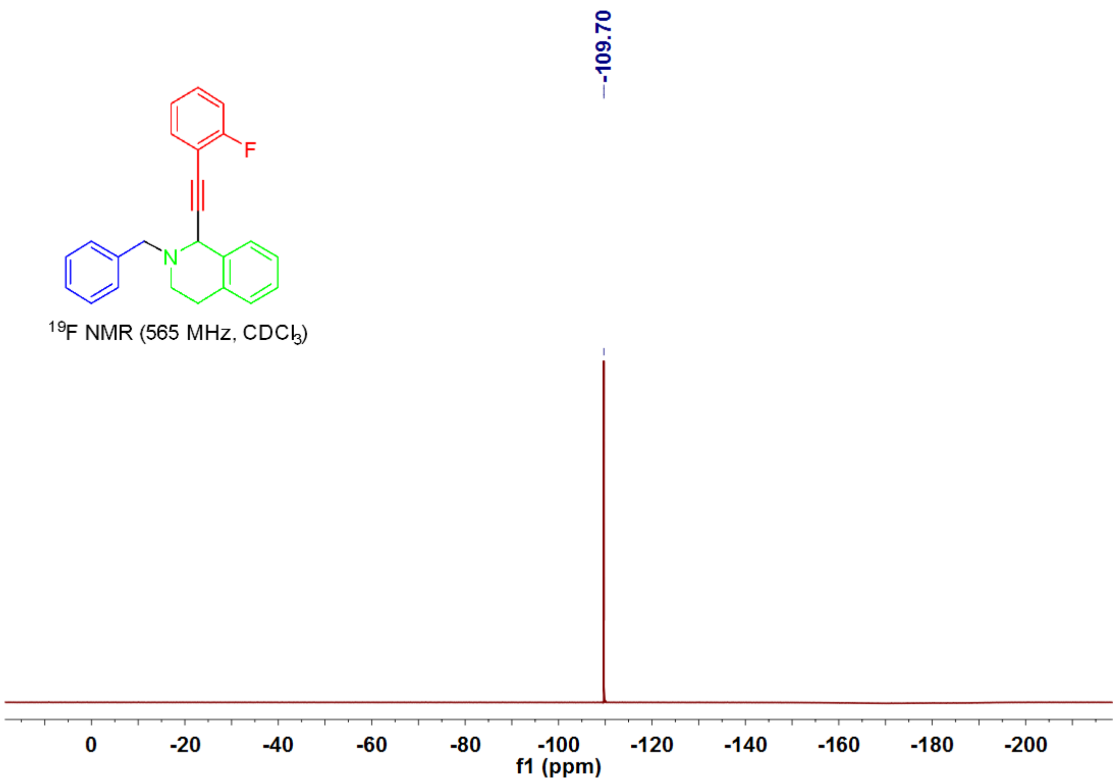

Supplementary Figure 220. ^{19}**F NMR** spectrum of compound **5k**.

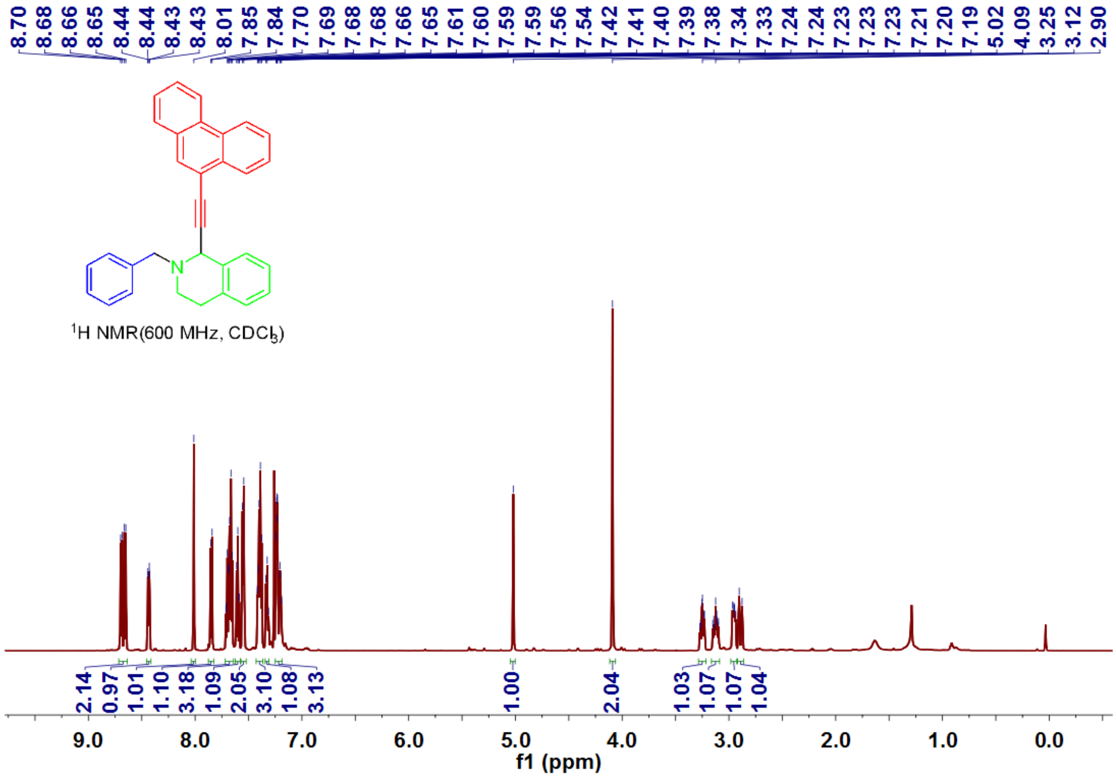

Supplementary Figure 221. ^{1}**H NMR** spectrum of compound **5l**.

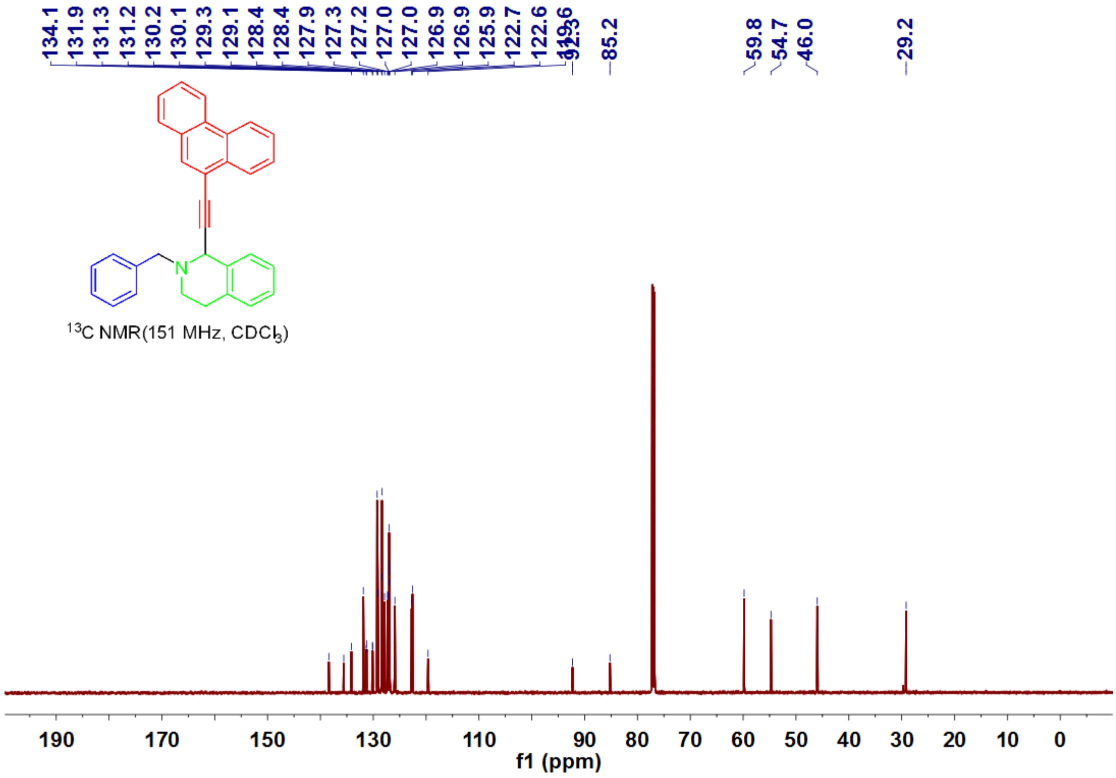

Supplementary Figure 222. ^{13}C NMR spectrum of compound **5l**.

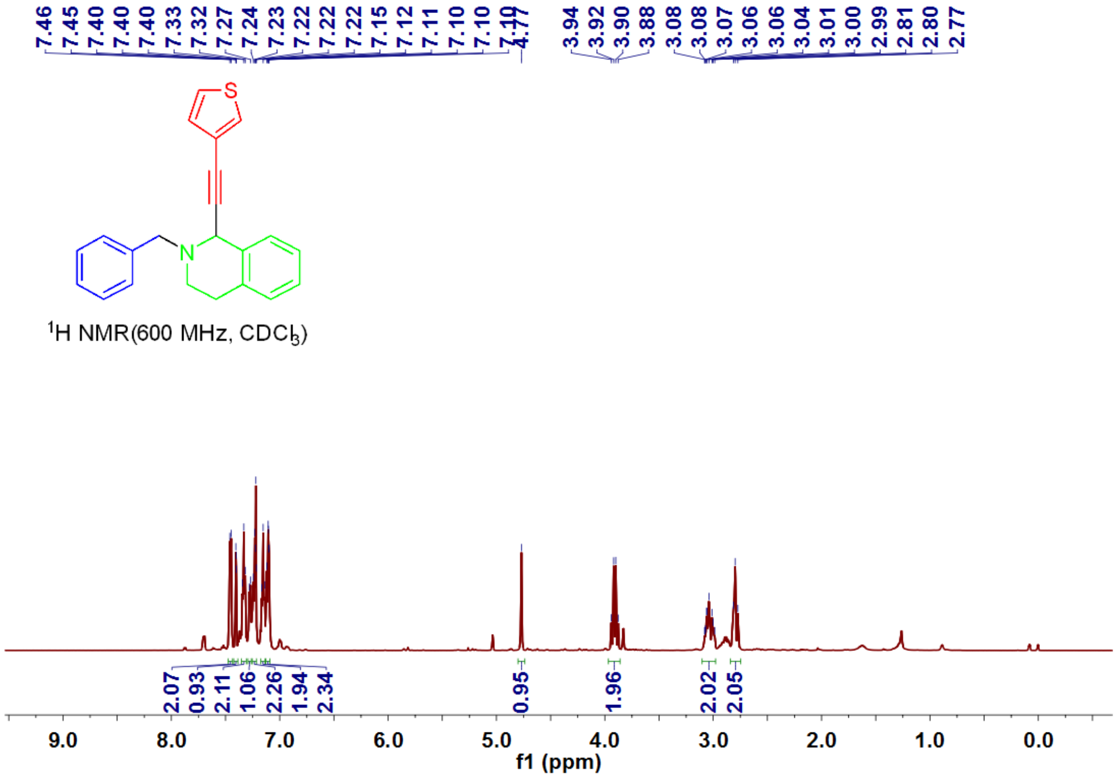

Supplementary Figure 223. ^{1}**H NMR** spectrum of compound **5m**.

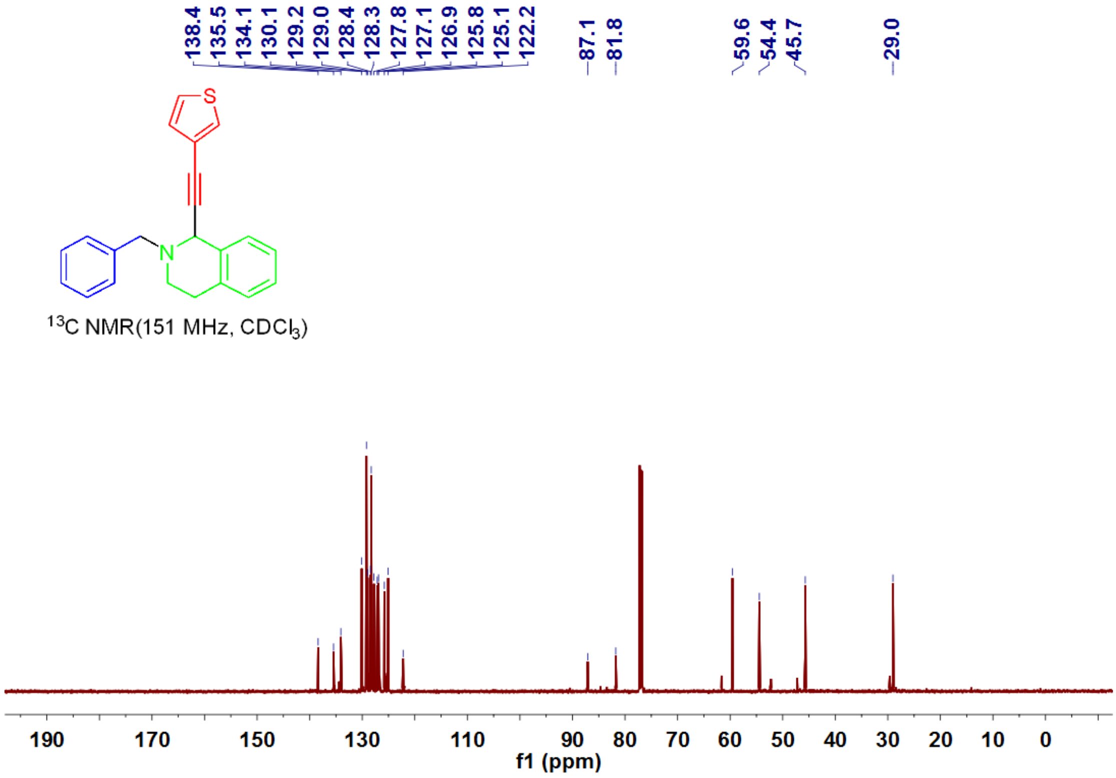

Supplementary Figure 224. ^{13}**C NMR** spectrum of compound **5m**.

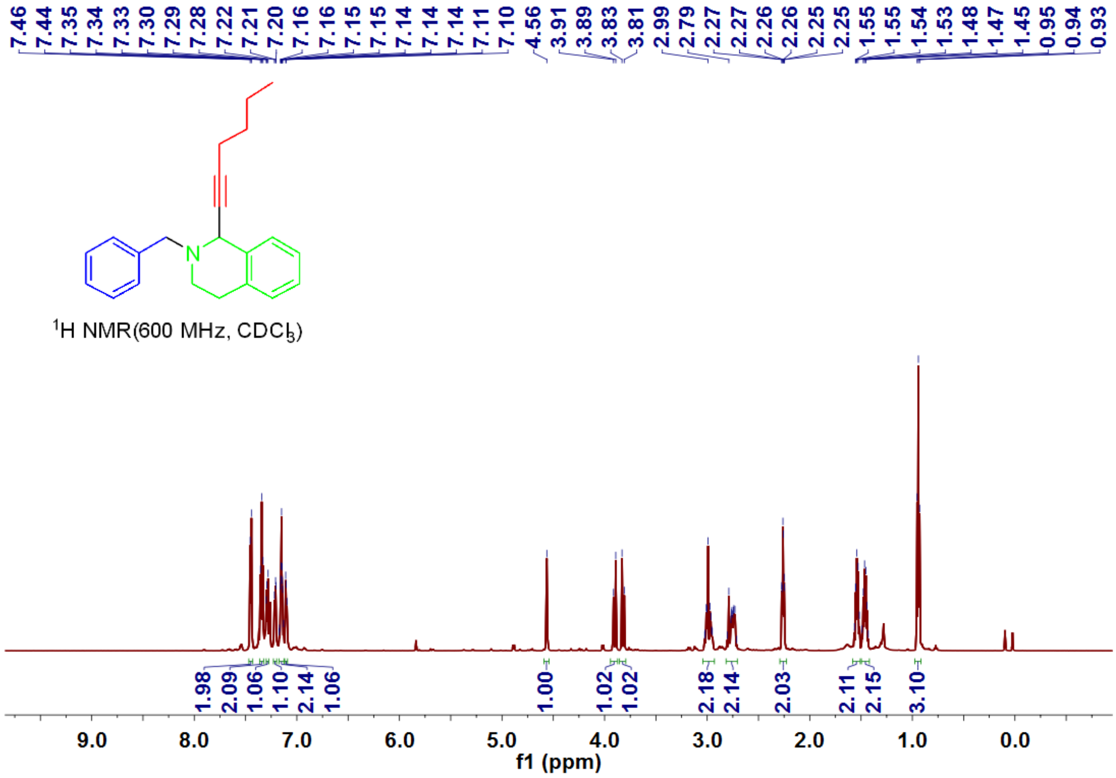

Supplementary Figure 225. ^{1}**H NMR** spectrum of compound **5n**.

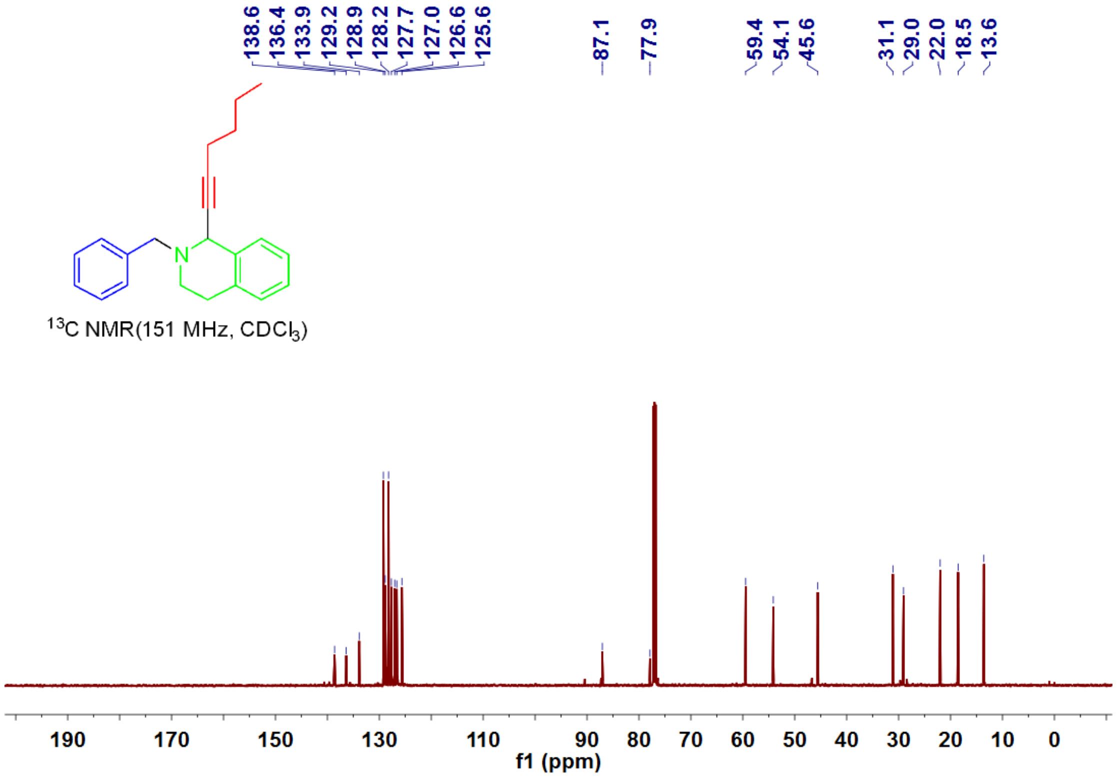

Supplementary Figure 226. ^{13}C NMR spectrum of compound **5n**.

References.

1. CrysAlisPro Version 1.171.36.31. Agilent Technologies Inc. Santa Clara, CA, USA (2012).
2. Sheldrick, G. M. A short history of shelx. *Acta. Cryst.* **A64**, 112–122 (2008).
3. Dolomanov, O. V., Bourhis, L. J., Gildea, R. J., Howard, J. A. -K., Puschmann, H. Olex2: a complete structure solution, refinement and analysis program. *J. Appl. Cryst.* **42**, 339–341 (2009).
4. Sheldrick, G. M. Crystal structure refinement with shelxl. *Acta. Cryst.* **C71**, 3–8 (2015).
5. Liu, L., Wang, Z., Wang, Z., Wang, R., Zang, S. & Mak, T. Mediating CO_2 electroreduction activity and selectivity over atomically precise copper clusters. *Angew. Chem. Int. Ed.* **61**, e202205626 (2022).
6. Murai, k., Morishita, M., Nakatani, R., Fujioka, H. & Kita, Y. Oxidative decarboxylative synthesis of 2-H-imidazolines from glyoxylic acid and 1,2-diaminesw. *Chem. Commun.* **2008**, 4498–4500 (2008).
7. Magill, A. M., McGuinness, D. S., Cavell, K. J., Britovsek, G. J., Gibson, V. C., White, A. J., Williams, G. J., White, A. H. & Skelton, B. W. Palladium(II) complexes containing mono-, bi- and tridentate carbene ligands. synthesis, characterisation and application as catalysts in C-C coupling reactions. *J. Organomet. Chem.* **617–618**, 546–560 (2001).
8. Kong, Y. J., Yan, Z. P., Li, S., Su, H. F., Li, K., Zheng, Y. X. & Zang, S. Q.

Photoresponsive propeller-like chiral AIE copper(I) clusters. *Angew. Chem. Int. Ed.* **59**, 5336–5340 (2020).
9. Mezei, G. & Raptis, R. G. Effect of pyrazole-substitution on the structure and nuclearity of Cu(II)-pyrazolato complexes. *Inorganica Chimica Acta.* **357**, 3279–3288 (2004).
10. Stephens, P., Devlin, F., Chabalowski, C. & Frisch, M. J. Ab initio calculation of vibrational absorption and circular dichroism spectra using density functional force fields. *J. Phys. Chem.* **98**, 11623–11627 (1994).
11. Becke, A. D. Density-functional thermochemistry. III. the role of exact exchange. *J. Chem. Phys.* **98**, 5648–5652 (1993).
12. Lee, C., Yang, W. & Parr, R. G. Development of the colle-salvetti correlation-energy formula into a functional of the electron density. *Phys. Rev. B* **37**, 785–789 (1988).
13. Miehlich, B., Savin, A., Stoll, H. & Preuss, H. Results obtained with the correlation energy density functionals of becke and Lee, Yang and Parr. *Chem. Phys. Lett.* **157**, 200–206 (1889).
14. Grimme, S. Semiempirical GGA-type density functional constructed with a long-range dispersion correction. *J. Comput. Chem.* **27**, 1787–1799 (2006).
15. Grimme, S. Semiempirical hybrid density functional with perturbative second-order correlation. *J. Chem. Phys.* **124**, 034108 (2006).
16. Hariharan, P. C. & Pople, J. A. The influence of polarization functions on molecular orbital hydrogenation energies. *Theor. Chim. Acta*. **28**, 213–222 (1973).
17. Hehre, W. J., Ditchfield, R. & Pople, J. A. Self-consistent molecular orbital methods. XII. further extensions of gaussian-type basis sets for use in molecular orbital studies of organic molecules. *J. Chem. Phys.* **56**, 2257–2261 (1972).
18. Marenich, A. V., Cramer, C. J. & Truhlar, D. G. Universal solvation model based on solute electron density and on a continuum model of the solvent defined by the bulk dielectric constant and atomic surface tensions. *The Journal of Physical Chemistry B* **113**, 6378–6396 (2009).
19. Fukui, K. Formulation of the reaction coordinate. *J. Phys. Chem.* **74**, 4161–4163 (1970).
20. Maeda, S., Harabuchi, Y., Ono, Y., Taketsugu, T. & Morokuma, K. Intrinsic reaction coordinate: calculation, bifurcation, and automated search. *Int. J. Quantum Chem.* **115**, 258–269 (2015).
21. Fukui, K. The path of chemical reactions the IRC approach. *Acc. Chem. Res.* **14**, 363–368 (1981).
22. Wang, M., Fan, T. & Lin, Z. DFT Studies on copper-catalyzed arylation of aromatic C-H bonds. *Organometallics* **31**, 560–569 (2012).
23. Fan, T., Sheong, F. K. & Lin, Z. DFT studies on copper-catalyzed hydrocarboxylation of alkynes using CO_2 and hydrosilanes. *Organometallics* **32**, 5224–5230 (2013).
24. Ariafard, A., Brookes, N. J., Stanger, R. & Yates, B. F. DFT study on the mechanism of the activation and cleavage of CO_2 by (NHC)CuEPh$_3$ (E = Si, Ge, Sn). *Organometallics* **30**, 1340–1349 (2011).
25. Eremin, E. N. The foundations of chemical kinetics. (Mir Pub. **1982**).

26. Schoenebeck, F. & Houk, K. N. Ligand-controlled regioselectivity in palladium-catalyzed cross coupling reactions. *J. Am. Chem. Soc.* **132**, 2496–2497 (2010).
27. Yu, H., Lu, Q., Dang, Z. & Fu, Y. Mechanistic study of the rhodium-catalyzed [3+2+2] carbocyclization of alkenylidenecyclopropanes with alkynes. *Chem. Asian J.* **8**, 2262–2273 (2013).
28. Ardura, D., López, R. & Sordo, T. L. Relative Gibbs energies in solution through continuum models: effect of the loss of translational degrees of freedom in bimolecular reactions on Gibbs energy barriers. *J. Phys. Chem. B* **109**, 23618–23623 (2005).
29. Liu, Q., Lan, Y., Liu, J., Li, G., Wu, Y. D. & Lei, A. Revealing a second transmetalation step in the negishi coupling and its competition with reductive elimination: improvement in the interpretation of the mechanism of biaryl syntheses. *J. Am. Chem. Soc.* **131**, 10201–10210 (2009).
30. Ariafard, A., Ghohe, N. M., Abbasi, K. K., Canty, A. J. & Yates, B. F. Theoretical investigation into the mechanism of 3-dGMP oxidation by [$Pt^{IV}Cl_4$(dach)]. *Inorg. Chem.* **52**, 707–717 (2013).
